# Characteristics of Randomized Clinical Trials in Surgery From 2008 to 2020: A Systematic Review

**DOI:** 10.1001/jamanetworkopen.2021.14494

**Published:** 2021-06-30

**Authors:** N. Bryce Robinson, Stephen Fremes, Irbaz Hameed, Mohamed Rahouma, Viola Weidenmann, Michelle Demetres, Mahmoud Morsi, Giovanni Soletti, Antonino Di Franco, Marco A. Zenati, Shahzad G. Raja, David Moher, Faisal Bakaeen, Joanna Chikwe, Deepak L. Bhatt, Paul Kurlansky, Leonard N. Girardi, Mario Gaudino

**Affiliations:** 1 Department of Cardiothoracic Surgery, Weill Cornell Medicine, New York, New York; 2 Schulich Heart Centre, Sunnybrook Health Sciences Centre, University of Toronto, Toronto, ON, Canada; 3 Division of Cardiothoracic Surgery, Yale University School of Medicine, New Haven, Connecticut; 4 Samuel J. Wood Library and C.V. Starr Biomedical Information Centre, Weill Cornell Medicine, New York, New York; 5 BHS Department of Cardiothoracic Surgery, West Roxbury, Massachusetts; 6 Department of Cardiac Surgery, Harefield Hospital, London, United Kingdom; 7 Ottawa Methods Centre, Ottawa Hospital Research Institute, Ottawa, Ontario, Canada; 8 Department of Thoracic Surgery, Heart and Vascular Institute, Cleveland Clinic, Cleveland, Ohio; 9 Department of Cardiovascular Surgery, Heart and Vascular Institute, Cleveland Clinic, Cleveland, Ohio; 10 Department of Cardiac Surgery, Smidt Heart Institute, Cedars-Sinai Medical Center, Los Angeles, California; 11 Division of Cardiovascular Medicine, Brigham and Women’s Hospital, Boston, Massachusetts; 12 Department of Surgery, Columbia University Medical Center, New York, New York

## Abstract

**Importance:**

Randomized clinical trials (RCTs) provide the highest level of evidence to evaluate 2 or more surgical interventions. Surgical RCTs, however, face unique challenges in design and implementation.

**Objective:**

To evaluate the design, conduct, and reporting of contemporary surgical RCTs.

**Evidence Review:**

A literature search performed in the 2 journals with the highest impact factor in general medicine as well as 6 key surgical specialties was conducted to identify RCTs published between 2008 and 2020. All RCTs describing a surgical intervention in both experimental and control arms were included. The quality of included data was assessed by establishing an a priori protocol containing all the details to extract. Trial characteristics, fragility index, risk of bias (Cochrane Risk of Bias 2 Tool), pragmatism (Pragmatic Explanatory Continuum Indicator Summary 2 [PRECIS-2]), and reporting bias were assessed.

**Findings:**

A total of 388 trials were identified. Of them, 242 (62.4%) were registered; discrepancies with the published protocol were identified in 81 (33.5%). Most trials used superiority design (329 [84.8%]), and intention-to-treat as primary analysis (221 [56.9%]) and were designed to detect a large treatment effect (50.0%; interquartile range [IQR], 24.7%-63.3%). Only 123 trials (31.7%) used major clinical events as the primary outcome. Most trials (303 [78.1%]) did not control for surgeon experience; only 17 trials (4.4%) assessed the quality of the intervention. The median sample size was 122 patients (IQR, 70-245 patients). The median follow-up was 24 months (IQR, 12.0-32.0 months). Most trials (211 [54.4%]) had some concern of bias and 91 (23.5%) had high risk of bias. The mean (SD) PRECIS-2 score was 3.52 (0.65) and increased significantly over the study period. Most trials (212 [54.6%]) reported a neutral result; reporting bias was identified in 109 of 211 (51.7%). The median fragility index was 3.0 (IQR, 1.0-6.0). Multiplicity was detected in 175 trials (45.1%), and only 35 (20.0%) adjusted for multiple comparisons.

**Conclusions and Relevance:**

In this systematic review, the size of contemporary surgical trials was small and the focus was on minor clinical events. Trial registration remained suboptimal and discrepancies with the published protocol and reporting bias were frequent. Few trials controlled for surgeon experience or assessed the quality of the intervention.

## Key Points

**Question:**

How have the design, conduct, and reporting of surgical randomized clinical trials changed from 2008 to 2020?

**Findings:**

In this systematic review of 388 randomized clinical trials, the sizes of surgical trials were small and the focus was on minor clinical events. Trial registration remained suboptimal and discrepancies with the published protocol and reporting bias were frequent. Few trials controlled for surgeon experience or assessed the quality of the intervention.

**Meaning:**

Results of this study suggest that improvements in the design, implementation, and reporting of future randomized clinical trials in surgery are warranted.

## Teaser

This systematic review describes the characteristics of the design, conduct, and reporting of randomized clinical trials in 6 surgical specialties published between 2008 and 2020.

## Introduction

In surgery, the decision to perform one type of operation instead of another is based on evaluation of the patient by the operating surgeon, and subjectivity inherent to the choice of treatment is unlikely to be neutralized even using complex statistical adjustment.^1^ For this reason, treatment allocation bias and unmeasured confounders may be associated with the treatment effect seen in comparative observational surgical studies more than in other medical fields. Only randomized clinical trials (RCTs) can reliably evaluate the true effects of different surgical interventions.^2,3^

Previous analyses of surgical RCTs have been limited to 1 or few specialties, few trial characteristics, and short time spans.^4,5^ We describe the characteristics of the design, conduct, and reporting of RCTs in 6 key surgical specialties published between 2008 and 2020 to evaluate surgical trials in the current era.

## Methods

### Search Strategy and Definitions

A literature search was performed by a medical librarian (M.D.) to identify all adult surgical RCTs published between January 1, 2008, and January 1, 2020, in the 2 journals with the highest impact factor in general medicine and in each of the following surgical specialties: cardiothoracic, general, neurosurgery, orthopedic, transplant, and vascular. The specialties were selected from those recognized by the American College of Surgeons^6^ with the aim of providing an overview of surgical specialties. The full search strategy is available in eTable 1 in the Supplement.

A surgical RCT was defined as a trial that involved a surgical intervention in both the experimental and control arms. A surgical intervention was defined as any procedure performed by a trained surgical specialist with the goal of correcting deformities or defects, repairing injuries, or for the cure of certain diseases, as specified by the National Center for Biotechnology Information.^7^ Trials evaluating nonsurgical interventions or medical treatments and trials with at least 1 nonsurgical arm were excluded because they are generally designed by nonsurgical trialists. Endovascular and percutaneous procedures were also excluded. In case of multiple reports from the same trial, the report of the primary analysis was selected.

This study was prospectively registered on the international prospective register of systematic reviews (CRD42020162797) and followed Preferred Reporting Items for Systematic Reviews and Meta-analyses (PRISMA) reporting guideline.^8^ The protocol was previously published.^9^

### Extraction of Trial Data

Two reviewers (N.B.R. and I.H.) independently screened the citations retrieved from the literature search and extracted all data following previously described methods and using a predefined data collection form.^10,11,12^ A third reviewer (M.G.) resolved any discrepancy. Additional details of trial data extraction are available in the eMethods in the Supplement.

Primary trial outcomes were classified as major or minor clinical end points based on a published classification scheme (eTable 2 in the Supplement).^13^

Trials were evaluated for pragmatism using the Pragmatic Explanatory Continuum Index Summary (PRECIS-2) tool, which uses a 5-point ordinal scale (ranging from very pragmatic to very explanatory) across 9 domains of trial design, including eligibility, recruitment, setting, organization, intervention delivery, intervention adherence, follow-up, primary outcome, and analysis. Trial primary outcomes were assessed for multiplicity and adjustment. When applicable, sponsor details, classification of the outcome as favorable or unfavorable, and identification of a discrepancy in the registered and reported trial outcome were collected. Superiority-design trials with at least 1 significant dichotomous primary outcome were eligible for fragility index (FI) calculation. In trials showing no significant difference in the primary outcome, reporting bias was appraised. Risk of bias was assessed using The Cochrane Risk of Bias Tool Version 2 (RoB 2) tool. Detailed methods are available in the eMethods in the Supplement.

### Statistical Analysis

Categorical variables were reported as counts and percentages. Following assessment of normality by visual inspection and Shapiro-Wilk normality test, continuous variables were reported as mean (SD) when normally distributed or median (interquartile range [IQR]) when not. Based on normality of data, independent samples *t* test or the Mann-Whitney U test was used to compare continuous variables. Categorical variables were compared using χ^2^ or Fisher exact tests. The *P* for trend (linear regression) was used to evaluate variations during the study period. Two-sided significance testing was used and a *P* value <.05 was considered significant without adjustment for multiple testing. All analyses were performed using R (version 3.6.2 R Project for Statistical Computing) within RStudio.

## Results

**Figure 1.**
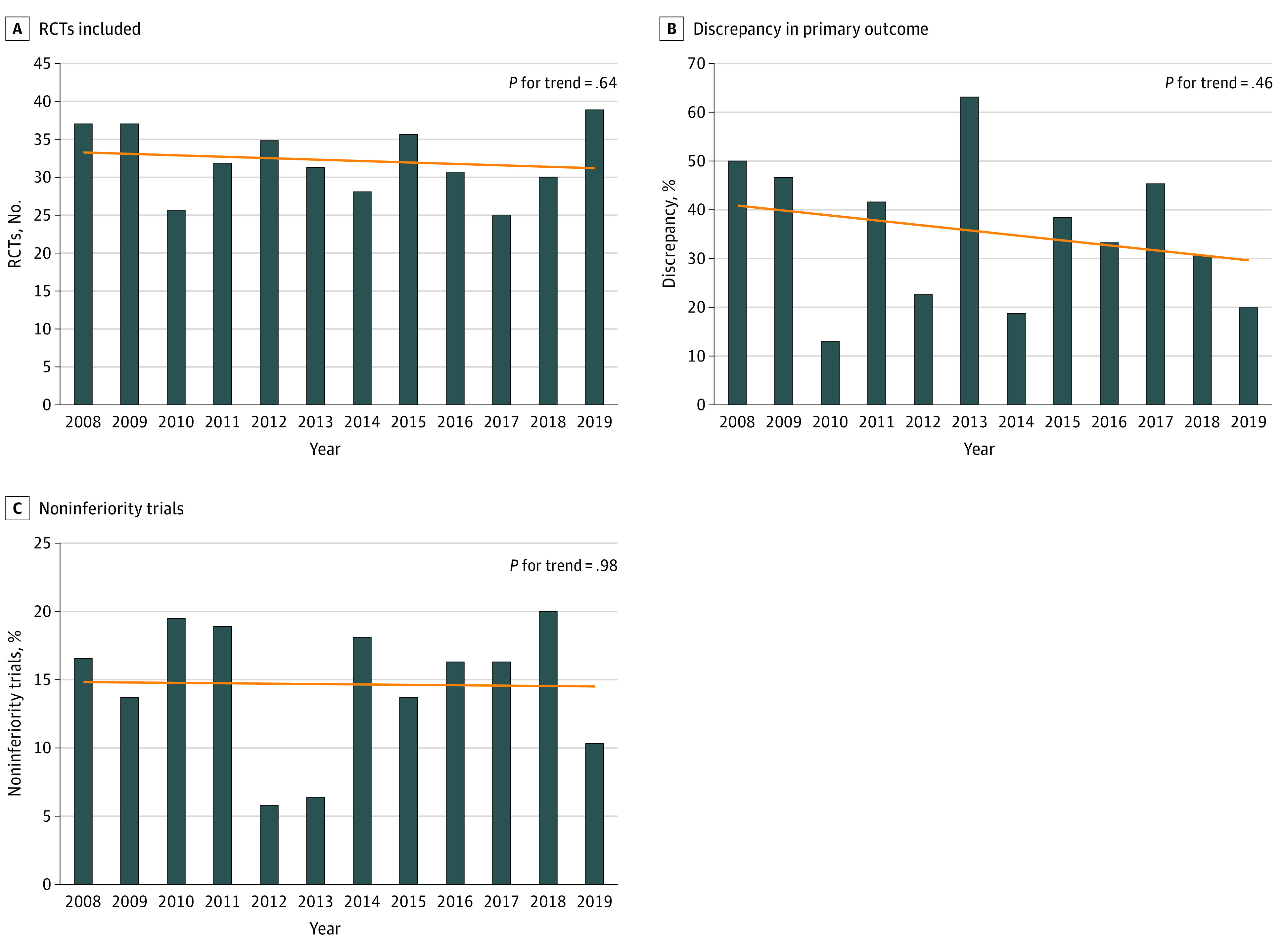
Randomized Clinical Trials During the Study Period A-C, By year, outcome, and study design.

From the 6699 articles screened, a total of 388 trials were included in the analysis (eFigure 1 in the Supplement). The number of published surgical trials did not significantly change during the study period (Figure 1A). Details of the 388 included clinical trials are available in eTable 3 in the Supplement.

### Trial Characteristics

Trial characteristics are summarized in Table 1. One-hundred-twenty-five trials (32.2%) investigated general surgery interventions; 116 (29.9%), orthopedic surgery; 93 (23.9%), cardiothoracic surgery; 21 (5.4%), neurosurgery; 19 (4.9%), vascular surgery; and 1 (0.3%), transplantation. A breakdown of general surgery trials by subspecialty is available in eTable 4 in the Supplement and results by specialty are provided in eTable 5 in the Supplement. Journal characteristics are available in the eResults in the Supplement.

**Table 1.** Trial Characteristics

Characteristic	No. (%) (N = 388)
Journal of publication	
* American Journal of Transplantation*	1 (0.3)
* Annals of Surgery*	103 (26.5)
* Arthroscopy*	47 (12.1)
* European Journal of Vascular and Endovascular Surgery*	10 (2.6)
*JAMA* Surgery	9 (2.3)
* Journal of Bone and Joint Surgery*	63 (16.3)
* Journal of Neurosurgery*	10 (2.6)
* Journal of Vascular Surgery*	9 (2.3)
* Neurosurgery*	10 (2.6)
* The Annals of Thoracic Surgery*	32 (8.2)
* The Journal of Thoracic and Cardiovascular Surgery*	44 (11.3)
* The Lancet*	29 (7.5)
* The New England Journal of Medicine*	21 (5.4)
* Journal of Heart and Lung Transplantation*	0
Location	
Africa	4 (1.0)
Asia	86 (22.2)
Australia	12 (3.1)
Europe	195 (50.3)
North America	65 (16.8)
South America	6 (1.5)
Multiple continents	20 (5.2)
Specialty	
General surgery	125 (32.2)
Orthopedic surgery	116 (29.9)
Cardiothoracic Surgery	93 (23.9)
Neurosurgery	21 (5.4)
Vascular surgery	19 (4.9)
Obstetrics and gynecology	10 (2.6)
Urology	2 (0.5)
Otolaryngology	1 (0.3)
Transplant	1 (0.3)
Multicenter trial	167 (43.0)

One hundred ninety-five (50.3%) trials originated from Europe; 86 (22.2%), from Asia; 65 (16.8%), from North America; 12 (3.1%), from Australia; 6 (1.5%), from South America; 4 (1.0%), from Africa; and 20 (5.2%), from multiple continents. One hundred sixty-seven trials (43.0%) were multicenter.

The median projected sample size was 144 patients (IQR, 85-299 patients) and did not change significantly over the course of the study period (157 patients in 2008 [IQR, 116-361 patients] vs 150 patients in 2019 [IQR, 90-224 patients]; *P* *=* .64 for trend). The largest sample size was 15 935 patients, and 17 studies (4.4%) enrolled more than 1000 patients. Of note, the median enrolled sample size was smaller than the projected sample size (122 patients; IQR, 70-245 patients) and 63 trials (20% of trials that reported a sample size calculation) did not reach their projected sample size.

### Trial Design

The details of trial design are summarized in Table 2. Two hundred forty-two (62.4%) trials were registered a priori; trial registration significantly increased over the study period (16.2% [6 of 37] registered in 2008 vs 89.7% [35 of 39] registered in 2019; *P* < .001) (eFigure 2, eTable 6 in the Supplement). In 81 (33.5%) of the registered trials, 1 or more discrepancies between the registered and published primary outcomes were found. Trials with discrepancies were significantly less likely to use intention-to-treat as the main analysis (59.7% vs 76.5%; *P* = .03) and had significantly lower mean (SD) PRECIS-2 scores (3.38 [0.63] vs 3.63 [0.66]; *P* = .01) (eTable 7 in the Supplement). There was no significant change in the rate of publication of trials with discrepancies during the study period (50.0% [3 of 6] trials with discrepancy in 2008 vs 20% [7 of 35] trials in 2019; *P* = .46 for trend) (Figure 1B).

**Table 2.** Trial Design of 388 Included Trials

Variable	Frequency, No. (%)
Registration in trials registry	242 (62.4)
Discrepancy between registered and primary outcome	81 (33.5)
Superiority design	329 (84.8)
Power, median (IQR), %	80.0 (80.0-90.0)
Estimated relative treatment effect, median (IQR), %	50.0 (24.7-63.3)
Estimated treatment effect of trials with a major clinical end point as primary outcome	50.0 (24.5-67.9)
Estimated treatment effect of trials with a minor clinical end point as primary outcome	46.6 (25.0-57.1)
Intention-to-treat as the primary analysis	221 (56.9)
Noninferiority design	55 (14.2)
Both noninferiority and superiority design	3 (0.8)
Use of composite primary outcome	82 (21.1)
Major clinical event as primary end point	123 (31.7)
No. of patients screened, median (IQR)	204 (105-465)
Sample size, median (IQR)	
Projected	144 (86-299)
Final	122 (70-245)
Duration of follow-up, median (IQR), mo	24.0 (12.0, 32.0)
Type of primary outcome	
Time to event	181 (46.7)
Quality of life	50 (12.8)
Other scales	157 (40.5)
Randomization	
Computer generated	213 (54.9)
Envelope	90 (23.2)
Random number table	36 (9.3)
Telephone call to randomization center	10 (2.6)
Drawing of lots	2 (0.5)
Date of birth	2 (0.5)
Flip of a coin	1 (0.3)
No details given	34 (8.7)
Blinding	
None	74 (19.1)
Outcome assessor only	61 (15.7)
Patient and outcome assessor	60 (15.4)
Patient only	32 (8.3)
Patient, outcome assessor, data analyst	18 (4.6)
Outcome assessor and data analyst	8 (2.1)
Data analyst only	6 (1.5)
Patient, surgeon, outcome assessor, data analyst	1 (0.3)
No details given	128 (33.0)
Control for surgeons’ experience	
None	303 (78.1)
Surgeons’ experience cut-off	60 (15.5)
Pretrial training	25 (6.4)
Monitoring of the intervention	
None	371 (95.6)
Photo	4 (1.0)
Video	9 (2.3)
Site visit	3 (0.8)
Data monitoring of outcomes	1 (0.3)
Details of the experimental procedure	
None	41 (10.6)
Limited	226 (58.2)
Detailed	121 (31.2)
Risk of bias assessment	
Low risk	86 (22.2)
Some concerns	211 (54.4)
High risk	91 (23.5)
Funding	
External	288 (74.2)
Industry	96 (33.3)
Industry sponsor involved in the analysis	51 (53.1)
Conflicts of interest	
First author with study sponsor	34 (35.4)
Last author with study sponsor	29 (30.2)
PRECIS-2 score, mean (SD)^a^	3.52 (0.65)

Abbreviations: IQR, interquartile range; PRECIS-2, Pragmatic Explanatory Continuum Indicator Summary 2.

^a^ PRECIS-2 uses a 5-point ordinal scale (ranging from very pragmatic to very explanatory) across 9 domains of trial design, including eligibility, recruitment, setting, organization, intervention delivery, intervention adherence, follow-up, primary outcome, and analysis.

A total of 329 trials (84.8%) used a superiority design. Trials that used a superiority design were significantly more likely to use intention-to-treat as the main analysis (58.0% [185 of 319] vs 42.3% [22 of 52]; *P* = .004) and had significantly higher PRECIS-2 score (mean [SD] score 3.55 [0.62] vs 3.24 [0.77]; *P* = .002). Trials using superiority design were more likely to have a high risk of bias compared with noninferiority trials (26.3% [84 of 319] vs 11.5% [6 of 52]; *P* = .02) (eTable 8 in the Supplement). There was no significant change in the use of the noninferiority design during the study period (16.2% [6 of 37] noninferiority design in 2008 vs 10.8% [4 of 37] noninferiority design in 2019; *P* = .98 for trend) (Figure 1C).

Trials had 80% power (IQR, 80.0%-90.0% power) and were designed to detect an estimated relative treatment effect of 50% (IQR, 24.7%-63.3%) without significant differences between trials that used major vs minor clinical events in the primary outcome (50.0%; IQR, 24.5%-67.9% vs 46.6%; IQR, 25.0%-57.1%; *P* = .12) (eFigure 3 in the Supplement). Slightly more than half of the trials (22 [56.9%]) used intention-to-treat as the primary analysis.

Most outcomes used (181 [46.7%]) were time to event, 50 (12.8%) were quality-of-life scores, and 157 (40.5%) were based on other ordinal scales. Eighty-two trials (21.1%) used a primary composite outcome. Only 123 trials (31.7%) used major clinical events in the primary outcome.

Most trials (303 [78.1%]) did not control for surgeon experience; 60 (15.5%) used an experience cut-off, and 25 (6.4%) used pretrial training. Most trials (n=371 [95.6%]) did not assess the quality of the intervention. Of the 17 trials that assessed the quality of the intervention, 4 trials (23.5%) used intraoperative images, 9 trials (52.9%) used video recording, 3 trials (17.7%) used site visits, and 1 trial (5.9%) used data monitoring of outcomes. Details of the trial intervention were limited in most reported trials (226 [58.2%]), detailed in 121 trials (31.2%), and not specified in 41 (10.6%).

Most trials (288 [74.2%]) reported an external funding source. Ninety-six trials (33.3%) were industry funded, and of those 50 (53.1%) reported involvement of industry in the analysis. In industry-funded trials, 34 (35.4%) reported that first authors and 29 (30.2%) reported that last authors disclosed a potentially relevant relationship with industry.

Risk of bias was assessed in all trials. Most trials (211 [54.4%]) had some concern for bias, 86 (22.2%) had low risk of bias, and 91 (23.5%) had high risk of bias. Risk of bias by domain is reported in eTable 9 in the Supplement.

**Figure 2.**
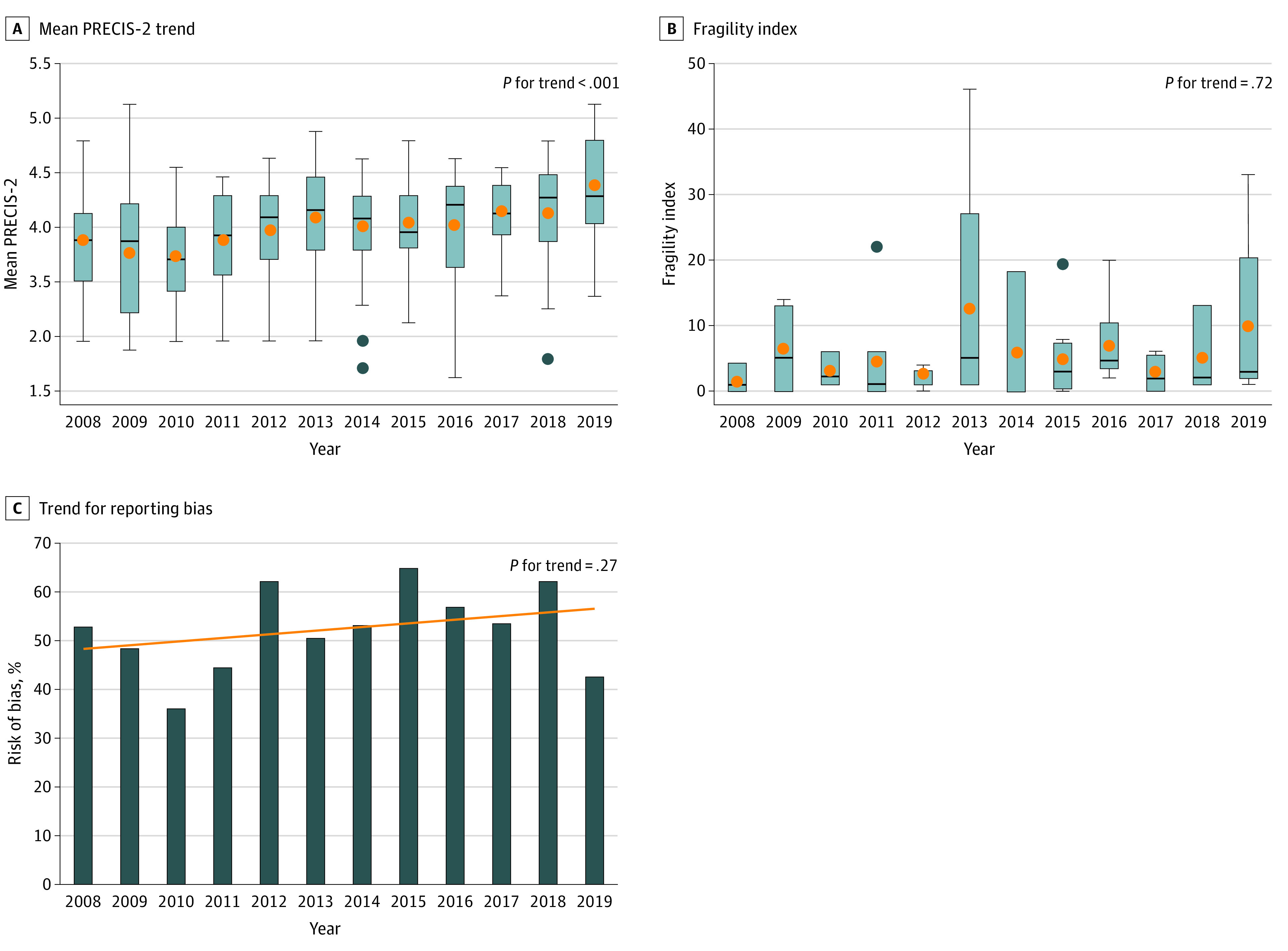
Evaluation of Randomized Clinical Trials A, Evaluation using the Pragmatic Explanatory Continuum Index Summary 2 (PRECIS-2) Tool. B, Evaluation using the Fragility Index. C, Evaluation with reporting bias.

The mean (SD) PRECIS-2 score was 3.52 (0.65) and increased significantly during the study period (mean [SD] PRECIS-2 was 3.34 [0.60] in 2008 vs 4.01 [0.62] in 2019; *P* < .001 for trend) (Figure 2A). The recruitment domain had the highest mean (SD) score (4.41 [0.78]), and flexibility in delivery domain had the lowest (2.90 [1.35]) (eTable 10 in the Supplement). The mean (SD) score varied widely among specialties and was highest in general surgery (3.70, [0.53]), and lowest in vascular surgery (3.21 [0.50]) (eTable 11 in the Supplement).

### Trial Implementation

Data on trial implementation are summarized in Table 3. Two hundred seventy-three (70.4%) trials reported details of the number of patients screened. The median percentage of screened patients who were enrolled was 76.8% (IQR, 45.1%-95.2%).

**Table 3.** Trial Implementation and Reporting

Variable	No. (%)
Screened patients included, median (IQR). %	76.8 (45.1-95.2)
Patients lost to follow up, median (IQR)	4.0 (0.0-17.0)
Sample size lost to follow up, median (IQR), %	3.3 (0.0-10.7)
Fragility index, median (IQR)	3.0 (1.0-6.0)
Fragility Index minus patients lost to follow up, median (IQR)	0.0 (0.0-3.0)
Crossovers, median (IQR), No.	1.0 (0.0-6.5)
Crossover, median (IQR), %	0.5 (0.0-3.0)
Trials	
With a favorable outcome	166 (42.7)
With a neutral outcome	212 (54.6)
Multiplicity	175 (45.1)
Multiple treatment groups	13 (7.4)
Multiple outcomes	66 (37.7)
Multiple analyses of the same outcome	66 (37.7)
Multiple outcomes + multiple analyses of the same outcome	23 (13.2)
Multiple treatment groups + multiple outcomes	4 (2.3)
Multiple treatment groups + multiple analyses of the same outcome	3 (1.7)
Adjusted for multiple comparisons	35 (20.0)
Bonferroni correction	25 (71.4)
Tukey test	7 (20.0)
Dunn test	1 (2.9)
Gatekeeping or hierarchical testing	1 (2.9)
Modified α value	1 (2.9)
Reporting bias present	109/211 (51.7)
Extent of reporting bias	
None	102 (48.3)
In 1 section other than conclusion	11 (5.2)
In conclusion only	34 (16.1)
In 2 sections	33 (15.6)
In all sections	31 (14.7)
Citations, median (IQR), No.	36 (15-91)

Abbreviation: IQR, interquartile range.

Most trials (213 [54.9%]) used a computer-generated randomization sequence. One hundred eighty-six (47.9%) trials used blinding. When blinding was used, the outcome assessors were most often blinded (61 [32.8%]), followed by patients and outcome assessors (60 [32.3%]). Patients only were blinded in 32 trials (17.2%). In 74 (19.1%) trials blinding was explicitly not used, and in 128 (33.0%) trials no details on blinding were given.

Crossover rates were generally low. The median percentage of crossover between treatment arms was 0.5% (IQR, 0.0%-3.0%). The median follow-up time was 24.0 months (IQR, 12.0-32.0 months). The median percentage of patients lost to follow-up was 3.7% (IQR, 0.0%-10.7%).

Sixty-two trials (16.0%) were eligible for calculation of the FI. The median FI was 3.0 (IQR 1.0-6.0 FI) (eFigure 4 in the Supplement). The median FI minus loss to follow-up was 0.0 (IQR, 0.0-3.0 loss to follow-up) (eFigure 5 in the Supplement). In 20 trials (32.2%), the number of patients lost to follow-up was higher than the FI. The FI did not significantly change over time (median FI, 1.0 [IQR, 0.5-2.5] in 2008 vs 3.0 [IQR, 2.0-13.0] in 2019; *P* = .72 for trend) (Figure 2B).

### Trial Reporting and Citations

Data on reporting and citations are summarized in Table 3. Ten trials (2.6%) did not report the results for the primary outcome: 7 trials (70.0%) were interim analyses and 3 trials (30.0%) did not explicitly define a primary outcome. One hundred sixty-six trials (42.7%) were reported as favorable and 212 (54.6%) as neutral. No difference in the rate of favorable results was found for trials at higher vs lower risk of bias (27.1% for favorable trials vs 20.8% for neutral trials; *P* = .15).

Multiplicity was detected in 175 trials (45.1%); there was a nonsignificant decrease during the study period (multiplicity identified in 51.8% [19 of 37] of trials in 2008 vs 30.8% [12 of 39] in 2019; *P* = .06 for trend). Of the trials in which multiplicity was detected, only 20.0% (35) adjusted for multiple comparisons. Details of multiplicity and adjustment are given in Table 3.

Two-hundred-eleven trials were eligible for the analysis of reporting bias. Reporting bias was identified in approximately half of the studies (109 of 211 trials [51.7%]). General surgery trials had the highest rate of reporting bias (65.1% [41 of 63]) and cardiothoracic surgery trials had the lowest (24.3% [9 of 37]) (eTable 12 in the Supplement). No differences in trial design or in study sponsor were found between trials with and without reporting bias. Details of reporting bias appraisal are summarized in eTable 13 in the Supplement. The rate of reporting bias did not significantly change over the course of the study period (reporting bias identified in 52.3% [11 of 21] of eligible trials in 2008 vs 42.8% [9 of 21] in 2019; *P* = .27 for trend) (Figure 2C).

The median number of citations in included trials was 36 (IQR, 15-91). Trials published in *The New England Journal of Medicine* had the highest number of citations (median 268.0; IQR, 168.0-401.0) and trials published in the *Journal of Neurosurgery* had the lowest number of citations (median 14.0; IQR, 3.2-27.2) (eTable 14 in the Supplement). The median number of citations was highest in general surgery trials (50.0; IQR, 20.0-114.0) and lowest in neurosurgery trials (21.0; IQR, 5.0-36.0) (eTable 15 in the Supplement). The median number of citations was similar for trials with a favorable vs neutral outcome (36.0; IQR, 15.2-89.5 for favorable trials vs 32.5; IQR, 15.0-91.0 for neutral trials; *P* = .90).

## Discussion

In this analysis, we systematically evaluated the surgical trials published in the 2 highest impact factor journals in 6 key surgical specialties between 2008 and 2020. We included 388 trials. The average number of published trials per year did not increase during the study period (average 32 per year). Most trials investigated general surgery interventions and were performed in Europe.

Of the trials, only 62.4% were registered a priori, and in 33.5% of the preregistered trials 1 or more discrepancies between the registered and published primary outcome were found. This discrepancy rate is higher than what has been reported in previous analyses limited to medical specialties^14^ but consistent with the rate described in an analysis of orthopedic trials.^15^ Changes in trial primary outcomes have been shown to be associated with larger treatment effects^16^ and, together with the lack of trial registration, may raise concerns of selective reporting.

Most trials (84.8%) used a superiority design and this finding remained constant over the 10 years of the study; this finding is in contrast with the increasing use of the noninferiority design that has been described in the other fields.^17,18^ While most of the trials (74.2%) reported an external funding source, industry sponsorship was limited to 33.3%, a rate much lower than the 53.2% reported, for example, among cardiovascular trials.^13^

The trials were generally small, with a median sample size of 122 patients and the median follow-up was 24 months.

Most trials were designed to evaluate a fairly large treatment effect (50%); only one-third of them (31.7%) used major clinical events as the primary outcome. Most trials (54.6%) were neutral. A prior analysis reported that most (57.0%) cardiovascular RCTs reported a positive outcome.^13^ Other analyses have similarly found that most published trials reported positive findings.^19,20^

Slightly more than one-half of the trials (56.9%) used intention-to-treat as the main analysis. Although intention-to-treat estimates may be biased toward the null in case of high rate of crossover or other protocol deviations, the comparability between groups (the most important strength of RCTs) is assured only when the randomized allocation is preserved.

Most trials did not adopt any method to control for surgeons’ experience (78.1%) or to assess the quality of the intervention performed (95.6%) and provided only limited details of the trial intervention (58.2%). There have been multiple examples of important trials in surgery in which failure to assure adequate delivery and quality of the tested interventions has significantly affected the outcomes.^21,22^

Blinding (in particular blinding of patients) is challenging in surgical trials. We found that approximately half (47.9%) of trials used some form of blinding, and that blinding of the outcome assessors and of the assessors and the patients was the most frequent strategy used. It is notable that 33.0% of trials did not report any information about blinding, and 19.1% explicitly did not use any form of blinding.

Crossover and loss to follow-up rates were low, suggesting excellent trial implementation and high commitment of the investigators to the trial protocol. Trial pragmatism, as measured by the PRECIS-2 score, increased significantly during the study period, similar to what has been reported for cardiovascular trials.^23^ Also, trials enrolled a large proportion of the screened patients (76.8%).

As in previous analyses in other fields,^24,25^ the robustness of the results of surgical trials was relatively low, with the change in condition of only 3 patients needed to switch the statistical significance for most of them. Twenty-three trials (37.1%) had an FI equal to or less than 1 and in 20 trials (32.2%) the number of patients lost to follow-up was higher than the FI, a notable finding because the event rate in patients lost to follow-up has been reported to be higher than in patients who remain in the study.^26^

Multiplicity was found in 45.1% of the trials and of them a small number adjusted for multiple testing (20.0%). This low rate is consistent with the results of a recent analysis limited to trials in the cardiovascular field^27^ and may raise questions because of the potential inflation of the type I error.

In more than half of the 212 trials with neutral results, we found evidence of interpretation bias, a finding consistent with previous analyses.^28,29^ While industry sponsorship has been associated with reporting bias in trials of cardiovascular interventions,^13^ an association was not confirmed among surgical trials.

In addition, the median number of citations of surgical trials during the study period was 36, but with important variations based on the journal of publication and the surgical specialty. Notably, the number of citations was similar for trials with positive or neutral results.

### Limitations

This study has limitations. We could not capture unpublished trials and cannot exclude publication bias. We have included only a limited number of surgical specialties and a limited number of journals, and it is possible that trials in specialties or journals not included in our analysis have different characteristics. The FI, the PRECIS-2 score, and the methods used for calculation of reporting bias have been extensively used in trials analyses, but have been analyzed and found to have several important limitations.^30^ Also, we have used only univariate analysis and have not adjusted for multiplicity, so we cannot exclude the risk of spurious associations and confounders.

## Conclusions

In this systematic review, the number of surgical RCTs published from 2008 to 2020 was relatively small and the average annual number did not increase with the time. Trial sizes were generally small and designed to detect a large effect in outcomes of secondary clinical importance. A substantial proportion of trials was not registered a priori; among those registered, discrepancies between the registered protocol and the published report were found. Blinding and control for surgeons’ experience were adopted in less than half of the trials and the results were relatively fragile. These data suggest that improvements in the design, implementation and reporting of randomized clinical trials in surgery are warranted.

## Notes

Supplement. **eMethods.** Supplementary Methods **eResults.** Supplementary Results **eTable 1.** Search Strategy **eTable 2.** Major and Minor Clinical Endpoints **eTable 3.** Details of the Trials Included in the Analysis (Ordered by Journal) **eTable 4.** General Surgery Trials (n = 125) Included in the Analysis by Subspecialty **eTable 5.** Details of Trials Published in the Analyzed Surgical Specialties **eTable 6.** Trial Registration by Year **eTable 7.** Comparison Between Trials With and Without Discrepancy in the Primary Outcome **eTable 8.** Comparison Between Superiority and Non-inferiority Trials **eTable 9.** Cochrane Risk of Bias 2 Tool Domains **eTable 10.** PRECIS-2 Assessment of Trial Pragmatism Across the Design Domains **eTable 11.** Mean PRECIS-2 Score Among Different Specialties **eTable 12.** Comparison Between Trials With and Without Spin **eTable 13.** Details of Spin Appraisal **eTable 14.** Number of Citations by Journal **eTable 15.** Number of Citations by Specialty **eFigure 1.** CONSORT Diagram **eFigure 2.** Temporal Trend for Percentage of Trials Registered a Priori **eFigure 3.** Hypothesized Treatment Effect Used in Sample Size Calculation **eFigure 4.** Distribution of Fragility Index **eFigure 5.** Distribution of Fragility Index Minus Lost to Follow Up (FI-LTF) eReferences.
