# Supplementary material for: Characteristics of Randomized Clinical Trials in Surgery From 2008 to 2020: A Systematic Review
Source: JAMA Netw Open. 2021 Jun 30;4(6):e2114494. doi: 10.1001/jamanetworkopen.2021.14494 (PMC8246313; doi:10.1001/jamanetworkopen.2021.14494)
Supplement: Supplement. — eMethods. Supplementary Methods eResults. Supplementary Results eTable 1. Search Strategy eTable 2. Major and Minor Clinical Endpoints eTable 3. Details of the Trials Included in the Analysis (Ordered by Journal) eTable 4. General Surgery Trials (n = 125) Included in the Analysis by Subspecialty eTable 5. Details of Trials Published in the Analyzed Surgical Specialties eTable 6. Trial Registration by Year eTable 7. Comparison Between Trials With and Without Discrepancy in the Primary Outcome eTable 8. Comparison Between Superiority and Non-inferiority Trials eTable 9. Cochrane Risk of Bias 2 Tool Domains eTable 10. PRECIS-2 Assessment of Trial Pragmatism Across the Design Domains eTable 11. Mean PRECIS-2 Score Among Different Specialties eTable 12. Comparison Between Trials With and Without Spin eTable 13. Details of Spin Appraisal eTable 14. Number of Citations by Journal eTable 15. Number of Citations by Specialty eFigure 1. CONSORT Diagram eFigure 2. Temporal Trend for Percentage of Trials Registered a Priori eFigure 3. Hypothesized Treatment Effect Used in Sample Size Calculation eFigure 4. Distribution of Fragility Index eFigure 5. Distribution of Fragility Index Minus Lost to Follow Up (FI-LTF) eReferences. [file jamanetwopen-e2114494-s001.pdf]

# Supplemental Online Content

Robinson NB, Fremes S, Hameed I, et al. Characteristics of randomized clinical trials in surgery from 2008 to 2020: a systematic review. *JAMA Netw Open*. 2021;4(6):e2114494. doi:10.1001/jamanetworkopen.2021.14494

**eMethods.** Supplementary Methods

**eResults.** Supplementary Results

**eTable 1.** Search Strategy

**eTable 2.** Major and Minor Clinical Endpoints

**eTable 3.** Details of the Trials Included in the Analysis (Ordered by Journal)

**eTable 4.** General Surgery Trials (n=125) Included in the Analysis by Subspecialty

**eTable 5.** Details of Trials Published in the Analyzed Surgical Specialties

**eTable 6.** Trial Registration by Year

**eTable 7.** Comparison Between Trials With and Without Discrepancy in the Primary Outcome

**eTable 8.** Comparison Between Superiority and Non-inferiority Trials

**eTable 9.** Cochrane Risk of Bias 2 Tool Domains

**eTable 10.** PRECIS-2 Assessment of Trial Pragmatism Across the Design Domains

**eTable 11.** Mean PRECIS-2 Score Among Different Specialties

**eTable 12.** Comparison Between Trials With and Without Spin

**eTable 13.** Details of Spin Appraisal

**eTable 14.** Number of Citations by Journal

**eTable 15.** Number of Citations by Specialty

**eFigure 1.** CONSORT Diagram

**eFigure 2.** Temporal Trend for Percentage of Trials Registered a Priori

© 2021 Robinson NB et al. *JAMA Network Open*.

**eFigure 3.** Hypothesized Treatment Effect Used in Sample Size Calculation

**eFigure 4.** Distribution of Fragility Index

**eFigure 5.** Distribution of Fragility Index Minus Lost to Follow Up (FI-LTF)

**eReferences.**

This supplemental material has been provided by the authors to give readers additional information about their work.

© 2021 Robinson NB et al. *JAMA Network Open*.

## eMethods:

### Extraction of trial data

For each eligible trial, the following data were extracted: journal of publication and 2018 IF (according to Thomson Reuters-Clarivate Analytics),[1] year of publication, details of the tested surgical interventions, single- or multi-center study, geographical locations of the participating centers, details of the primary outcome (definition of the outcome, composite or non-composite endpoint), number of screened patients and percentage of screened patients enrolled, sample size, statistical power, treatment effect estimate used for sample size calculation, blinded or unblinded assessment of outcomes, details of the primary analysis (intention-to-treat, modified intention-to-treat, per-protocol, as treated, superiority, equivalence, or non-inferiority), adjustment for multiple testing, details of trial sponsor, declared conflicts of interest of the first and last author, length of follow-up, number of patients lost to follow-up, number of cross-overs between treatment groups, and number of citations on Scopus/Web of Science.

### Assessment of learning curve effect and deliverability of the intervention

The methods used to control for a possible learning curve effect and assure deliverability of the intervention and to monitor the quality of the intervention were also extracted. Data on the level of details of the interventions provided in the protocol were also collected and categorized using a semi-quantitative scale: none, limited, detailed.

### Extraction of trial primary outcome

The trials' primary outcome was determined according to a published method[2] by sequentially analyzing the methods section of the main trial report, a trial design manuscript if available, and the outcome used in the sample size calculation.

### Determination of author conflict of interest

For industry sponsored trials, the conflicts of interest of the first and last authors were identified from the disclosure statements published in the trials or the supplementary material. For trials listing co-first authors, disclosures of both authors in the list were considered. Authors' conflicts of interest were defined as any report of consulting, advisory, or speaking fees or honoraria, stock ownership, affiliation, or employment by the study sponsor.

### Appraisal of trial pragmatism

Using previously described methodology,[3] the Pragmatic Explanatory Continuum Index Summary (PRECIS-2) tool was used to evaluate trial pragmatism in nine domains: eligibility criteria, recruitment, setting, organization, flexibility of intervention delivery, flexibility of adherence to the intervention, follow-up, primary outcome, and primary analysis. Briefly, the PRECIS-2 tool is used to assess if a trial design is pragmatic or explanatory. Pragmatic trials are designed to test the effects of an intervention under the typical circumstances in which it will be applied in a real-world setting, whereas explanatory trials are designed to test interventions under ideal circumstances.[4] A 5-point Likert scale was used by two reviewers (N.B.R. and I.H.) to independently rate the level of pragmatism in each trial design domain as follows: (1) very explanatory, (2) rather explanatory, (3) equally pragmatic/explanatory, (4) rather pragmatic, and (5) very pragmatic. Disagreements between reviewers were reconciled by a third reviewer (M.G.). For each domain, the mean of the scores was considered. A summary score was then calculated using the mean score over the nine domains.

### Assessment of multiplicity and adjustment

Each trial was assessed for multiplicity for the primary outcome as well as for any multiplicity correction as previously reported.[5] Multiplicity refers to the use of multiple testing, for example across multiple comparison arms, analysis of multiple outcomes, or multiple analyses of the same outcome, which can contribute to increased risk of type I error.[6]

© 2021 Robinson NB et al. *JAMA Network Open*.

**Sponsor**

Trials were classified as commercially-sponsored if they were either industry-initiated and/or sponsored, or investigator-initiated studies that received commercial support. Trials were classified as non-commercially-sponsored if they were investigator-initiated and report local government or federal or hospital or university sponsorship, or no sponsors. For commercially-sponsored trials, the body of the articles, supplementary materials and original trial designs were additionally analyzed for reporting of commercial or sponsor involvement in the trial design, conduct, analysis, or reporting.

**Classification of trial results**

Consistent with previous reports,[7–9] trials were classified as “favorable” if, for at least one primary outcome among those defined in the protocol, the experimental therapy was significantly better than the control therapy ($p < 0.05$ or a 95% confidence interval [CI] which excludes the null value) in superiority trials, the experimental therapy did not exceed the non-inferiority margin in non-inferiority trials, or the effects of the treatments differed by no more than the equivalence margin in equivalence trials.

**Appraisal of Spin**

In studies reporting non-significant difference in the primary outcome, the presence and amount of distortion or misrepresentation of benefit, or “spin” (defined as the use of specific reporting strategies to suggest that the experimental treatment was beneficial or non-inferior despite a statistically non-significant difference for the primary outcome, or to distract the reader from statistically non-significant results[8]) was evaluated. For the purposes of this analysis, we evaluated spin as previously reported by Boutron et. al and others.[10,11] Specifically, spin was defined as a present if a trial, in which the primary outcome was not significantly different, attempted to use language highlighting that the treatment was in fact beneficial to distract the reader from the results.

Following this described method,[10,11] the presence of spin was assessed in the following sections of the manuscript by two independent reviewers (N.B.R. and I.H.) blinded to trial details: title, abstract results, main text results, discussion, abstract conclusions and main text conclusions. The strategies of spin considered were: (1) a focus on secondary statistically significant results (such as within-group comparison, secondary outcomes, subgroup analyses, modified population of analyses); (2) interpreting statistically non-significant results for the primary outcomes as showing treatment equivalence or comparable effectiveness; (3) claiming or emphasizing the beneficial effect of the experimental treatment despite statistically non-significant results; and (4) claiming or emphasizing non-inferiority despite not establishing non-inferiority boundaries or when data are inconclusive. Other spin strategies that are not classified according to this scheme were recorded and classified as “other”. The number of sections with spin was recorded and the extent of spin across a study was defined as the number of sections with spin in the entire article.

**Assessment of discrepancy between the registered and published primary outcomes**

Only trials prospectively registered that clearly describe the primary outcome in the registry were considered in this analysis. For each trial, the registration number in the published articles or clinical trial registries (ClinicalTrials.gov, ISRCTN register, or country-specific registries) was identified. Consistent with previous definitions,[12,13] major discrepancies were defined as: (1) a pre-specified primary outcome in the trial registration protocol reported as a secondary outcome in the final published article; (2) the published primary outcome described as a secondary outcome in the registry; (3) the pre-specified primary outcomes in the trial registration not reported in the published article; (4) a new primary outcome introduced in the published article; and (5) different timing of assessment of the primary outcome in the registered protocol and published article.[13]

**Calculation of the Fragility Index**

Superiority-design trials reporting at least one statistically significant dichotomous primary outcome ($p < 0.05$ or a 95% CI excluding the null value) were eligible for calculation of the FI.

First described by Walsh et al[14] the FI is defined as the number of patients whose status would need to switch from non-event to event to render a statistically significant difference non-significant. It is a method for quatifying the robustness of a trials results. This measurement provides an additional metric to measure the potential weakness and limitations of a trial. While no threshold exists to distinguish fragile from non-fragile trials, a lower FI signifies that a change in just a few patient outcomes could change the direction of the trials conclusion, and caution should be exercised when interpreting the results.

The results for each outcome were entered in a 2x2 contingency table following which the p-value for each outcome was calculated using the two-sided Fisher's exact test. Single participants were then iteratively shifted one at a time in the lower-incidence treatment group from "non-event" to "event" and the p-value for the 2x2 table was re-calculated. The FI for an outcome was reported as the smallest number of patients required to turn the re-calculated p-value non-significant ($\geq$0.05). Lower values indicate less robust results. The difference between the FI and the number of patients lost to follow-up was calculated following the methods used by Mazzinari et al.[15]

**Risk of Bias Assessment**

The Cochrane Risk of Bias Tool Version 2 (RoB 2) was used to assess each RCT for the presence of bias.[16] The RoB 2 tool provides a comprehensive approach to assess for bias introduced to the results of randomized trials across five domains including: bias arising from the randomization process, bias due to deviations from intended interventions, bias due to missing outcome data, bias in measurement of the outcome, and bias in selection of the reported result. For each domain, risk of bias was categorized into one of three categories: low, some concerns, or high. A study was classified as low risk if all domains were categorized as low risk, some concerns if at least one domain was categorized as some concerns but not found to be high risk for any domain, and high risk if at least one domain was categorized as high risk or if the study was judged to have some concern across multiple domains in a way that substantially impacted trial results.

© 2021 Robinson NB et al. *JAMA Network Open*.

## eResults:

### Trial characteristics

One-hundred three (26.5%) trials were published in the *Annals of Surgery*, 63 (16.3%) in the *Journal of Bone and Joint Surgery*, 47 (12.1%) in *Arthroscopy*, 44 (11.3%) in *The Journal of Thoracic and Cardiovascular Surgery*, 32 (8.2%) in *The Annals of Thoracic Surgery,* 29 (7.5%) in *The Lancet*, 21 (5.4%) in *The New England Journal of Medicine,* 10 (2.6%) in the *Journal of Neurosurgery*, 10 (2.6%) in the *European Journal of Vascular and Endovascular Surgery*, 10 (2.6%) in *Neurosurgery*, 9 (2.3%) in the *Journal of Vascular Surgery*, 9 (2.3%) in *JAMA Surgery*, 1 (0.3%) in the *American Journal of Transplantation*, and none in the *Journal of Heart and Lung Transplantation*.

Thirteen trials from surgical specialties not included in the systematic search were published in general medical journals (10 [2.6%] obstetrics and gynecology, 2 [0.5%] urology, 1 [0.3%] otolaryngology). These trials were included in the main analysis but excluded from the analyses by specialty.

© 2021 Robinson NB et al. *JAMA Network Open*.

**eTable 1**: Search Strategy

*Randomized controlled trial search filter used:*
BMJ Publishing Group Limited. BMJ Best Practice Study Design Search Filters 2017 [Available from: https://bestpractice.bmj.com/info/us/toolkit/learn-ebm/study-design-search-filters/]

**Ovid MEDLINE (ALL 1946 to December 10, 2019/August 17, 2020)**
**Searched on Dec 11, 2019; Updated Aug 18, 2020**

| **A. General Medicine** |
|---|
| **The New England Journal of Medicine**<br>Line # \| Search \| # of results<br>1 "randomized controlled trial".pt. 495639<br>2 (random$ or placebo$ or single blind$ or double blind$ or triple blind$).ti,ab. 1186604<br>3 (retraction of publication or retracted publication).pt. 14645<br>4 or/1-3 1298384<br>5 (animals not humans).sh. 4615830<br>6 ((comment or editorial or meta-analysis or practice-guideline or review or letter) not "randomized controlled trial").pt. 4409095<br>7 (random sampl$ or random digit$ or random effect$ or random survey or random regression).ti,ab. not "randomized controlled trial".pt. 84751<br>8 4 not (5 or 6 or 7) 951334<br>9 "new england journal of medicine".jn. 78687<br>10 limit 9 to yr="2008 - 2018" 16267<br>11 8 and 10 1477<br>UPDATE:<br>12 limit 9 to yr="2019" 1497<br>13 8 and 12 173<br>TOTAL: 1650 |

© 2021 Robinson NB et al. *JAMA Network Open*.

**The Lancet**

1 "randomized controlled trial".pt. 495639
2 (random$ or placebo$ or single blind$ or double blind$ or triple blind$).ti,ab. 1186604
3 (retraction of publication or retracted publication).pt. 14645
4 or/1-3 1298384
5 (animals not humans).sh. 4615830
6 ((comment or editorial or meta-analysis or practice-guideline or review or letter) not "randomized controlled trial").pt. 4409095
7 (random sampl$ or random digit$ or random effect$ or random survey or random regression).ti,ab. not "randomized controlled trial".pt. 84751
8 4 not (5 or 6 or 7) 951334
9 lancet.jn. 137172
10 limit 9 to yr="2008 - 2018" 17342
11 8 and 10 1222
UPDATE:
12 limit 9 to yr="2019" 1693
13 8 and 12 128
TOTAL: 1350

**B. General Surgery**

**Annals of Surgery**

1 "randomized controlled trial".pt. 495639
2 (random$ or placebo$ or single blind$ or double blind$ or triple blind$).ti,ab. 1186604
3 (retraction of publication or retracted publication).pt. 14645
4 or/1-3 1298384
5 (animals not humans).sh. 4615830
6 ((comment or editorial or meta-analysis or practice-guideline or review or letter) not "randomized controlled trial").pt. 4409095
7 (random sampl$ or random digit$ or random effect$ or random survey or random regression).ti,ab. not "randomized controlled trial".pt. 84751
8 4 not (5 or 6 or 7) 951334
9 "annals of surgery".jn. 30910
10 limit 9 to yr="2008 - 2018" 4883
11 8 and 10 515
UPDATE:
12 limit 9 to yr="2019" 758
13 8 and 12 79
TOTAL: 594

© 2021 Robinson NB et al. *JAMA Network Open*.

**JAMA Surgery**
1 "randomized controlled trial".pt. 495639
2 (random$ or placebo$ or single blind$ or double blind$ or triple blind$).ti,ab. 1186604
3 (retraction of publication or retracted publication).pt. 14645
4 or/1-3 1298384
5 (animals not humans).sh. 4615830
6 ((comment or editorial or meta-analysis or practice-guideline or review or letter) not "randomized controlled trial").pt. 4409095
7 (random sampl$ or random digit$ or random effect$ or random survey or random regression).ti,ab. not "randomized controlled trial".pt. 84751
8 4 not (5 or 6 or 7) 951334
9 "jama surgery".jn. 2469
10 limit 9 to yr="2008 - 2018" 1973
11 8 and 10 68
UPDATE:
12 limit 9 to yr="2019" 486
13 8 and 12 25
TOTAL: 93

**C. Cardiothoracic surgery Surgery**

**The Journal of Thoracic and Cardiovascular Surgery**
1 "randomized controlled trial".pt. 495639
2 (random$ or placebo$ or single blind$ or double blind$ or triple blind$).ti,ab. 1186604
3 (retraction of publication or retracted publication).pt. 14645
4 or/1-3 1298384
5 (animals not humans).sh. 4615830
6 ((comment or editorial or meta-analysis or practice-guideline or review or letter) not "randomized controlled trial").pt. 4409095
7 (random sampl$ or random digit$ or random effect$ or random survey or random regression).ti,ab. not "randomized controlled trial".pt. 84751
8 4 not (5 or 6 or 7) 951334
9 "journal of thoracic & cardiovascular surgery".jn. 28100
10 limit 9 to yr="2008 - 2018" 9569
11 8 and 10 334
UPDATE:
12 limit 9 to yr="2019" 1639
13 8 and 12 34
TOTAL: 368

© 2021 Robinson NB et al. *JAMA Network Open*.

**Annals of thoracic surgery**
1 "randomized controlled trial".pt. 495639
2 (random$ or placebo$ or single blind$ or double blind$ or triple blind$).ti,ab. 1186604
3 (retraction of publication or retracted publication).pt. 14645
4 or/1-3 1298384
5 (animals not humans).sh. 4615830
6 ((comment or editorial or meta-analysis or practice-guideline or review or letter) not "randomized controlled trial").pt. 4409095
7 (random sampl$ or random digit$ or random effect$ or random survey or random regression).ti,ab. not "randomized controlled trial".pt. 84751
8 4 not (5 or 6 or 7) 951334
9 "annals of thoracic surgery".jn. 35979
10 limit 9 to yr="2008 - 2018" 12309
11 8 and 10 329
UPDATE:
12 limit 9 to yr="2019" 991
13 8 and 12 26
TOTAL: 355

**D. Neurosurgery**

**Neurosurgery**
1 "randomized controlled trial".pt. 495639
2 (random$ or placebo$ or single blind$ or double blind$ or triple blind$).ti,ab. 1186604
3 (retraction of publication or retracted publication).pt. 14645
4 or/1-3 1298384
5 (animals not humans).sh. 4615830
6 ((comment or editorial or meta-analysis or practice-guideline or review or letter) not "randomized controlled trial").pt. 4409095
7 (random sampl$ or random digit$ or random effect$ or random survey or random regression).ti,ab. not "randomized controlled trial".pt. 84751
8 4 not (5 or 6 or 7) 951334
9 neurosurgery.jn. 17748
10 limit 9 to yr="2008 - 2018" 5622
11 8 and 10 178
UPDATE:
12 limit 9 to yr="2019" 659
13 8 and 12 29
TOTAL: 207

© 2021 Robinson NB et al. *JAMA Network Open*.

**Journal of Neurosurgery**
1 "randomized controlled trial".pt. 495639
2 (random$ or placebo$ or single blind$ or double blind$ or triple blind$).ti,ab. 1186604
3 (retraction of publication or retracted publication).pt. 14645
4 or/1-3 1298384
5 (animals not humans).sh. 4615830
6 ((comment or editorial or meta-analysis or practice-guideline or review or letter) not "randomized controlled trial").pt. 4409095
7 (random sampl$ or random digit$ or random effect$ or random survey or random regression).ti,ab. not "randomized controlled trial".pt. 84751
8 4 not (5 or 6 or 7) 951334
9 "journal of neurosurgery".jn. 23817
10 limit 9 to yr="2008 - 2018" 5786
11 8 and 10 232
UPDATE:
12 limit 9 to yr="2019" 589
13 8 and 12 30
TOTAL: 262

**E. Orthopedics**

**Journal of bone and joint surgery - American volume**
1 "randomized controlled trial".pt. 495639
2 (random$ or placebo$ or single blind$ or double blind$ or triple blind$).ti,ab. 1186604
3 (retraction of publication or retracted publication).pt. 14645
4 or/1-3 1298384
5 (animals not humans).sh. 4615830
6 ((comment or editorial or meta-analysis or practice-guideline or review or letter) not "randomized controlled trial").pt. 4409095
7 (random sampl$ or random digit$ or random effect$ or random survey or random regression).ti,ab. not "randomized controlled trial".pt. 84751
8 4 not (5 or 6 or 7) 951334
9 "journal of bone & joint surgery american volume".jn. 19053
10 limit 9 to yr="2008 - 2018" 4988
11 8 and 10 457
UPDATE:
12 limit 9 to yr="2019" 442
13 8 and 12 54
TOTAL: 511

© 2021 Robinson NB et al. *JAMA Network Open*.

| |
|---|
| **Arthroscopy- The journal of Arthroscopic and Related Surgery**<br>1 "randomized controlled trial".pt. 495639<br>2 (random$ or placebo$ or single blind$ or double blind$ or triple blind$).ti,ab. 1186604<br>3 (retraction of publication or retracted publication).pt. 14645<br>4 or/1-3 1298384<br>5 (animals not humans).sh. 4615830<br>6 ((comment or editorial or meta-analysis or practice-guideline or review or letter) not "randomized controlled trial").pt. 4409095<br>7 (random sampl$ or random digit$ or random effect$ or random survey or random regression).ti,ab. not "randomized controlled trial".pt. 84751<br>8 4 not (5 or 6 or 7) 951334<br>9 arthroscopy.jn. 7687<br>10 limit 9 to yr="2008 - 2018" 3595<br>11 8 and 10 228<br>UPDATE:<br>12 limit 9 to yr="2019" 604<br>13 8 and 12 32<br>TOTAL: 260 |
| **F. Transplant surgery** |
| **Journal of Heart and Lung Transplantation**<br>1 "randomized controlled trial".pt. 495639<br>2 (random$ or placebo$ or single blind$ or double blind$ or triple blind$).ti,ab. 1186604<br>3 (retraction of publication or retracted publication).pt. 14645<br>4 or/1-3 1298384<br>5 (animals not humans).sh. 4615830<br>6 ((comment or editorial or meta-analysis or practice-guideline or review or letter) not "randomized controlled trial").pt. 4409095<br>7 (random sampl$ or random digit$ or random effect$ or random survey or random regression).ti,ab. not "randomized controlled trial".pt. 84751<br>8 4 not (5 or 6 or 7) 951334<br>9 "journal of heart & lung transplantation".jn. 6581<br>10 limit 9 to yr="2008 - 2018" 2540<br>11 8 and 10 120<br>UPDATE:<br>12 limit 9 to yr="2019" 206<br>13 8 and 12 13<br>TOTAL: 133 |

© 2021 Robinson NB et al. *JAMA Network Open*.

**American Journal of Transplantation**
1 "randomized controlled trial".pt. 495639
2 (random$ or placebo$ or single blind$ or double blind$ or triple blind$).ti,ab. 1186604
3 (retraction of publication or retracted publication).pt. 14645
4 or/1-3 1298384
5 (animals not humans).sh. 4615830
6 ((comment or editorial or meta-analysis or practice-guideline or review or letter) not "randomized controlled trial").pt. 4409095
7 (random sampl$ or random digit$ or random effect$ or random survey or random regression).ti,ab. not "randomized controlled trial".pt. 84751
8 4 not (5 or 6 or 7) 951334
9 "american journal of transplantation".jn. 7464
10 limit 9 to yr="2008 - 2018" 4796
11 8 and 10 225
UPDATE:
12 limit 9 to yr="2019" 455
13 8 and 12 31
TOTAL: 256

**G. Vascular Surgery**

**European Journal of Vascular and Endovascular Surgery - EJVES**
1 "randomized controlled trial".pt. 495639
2 (random$ or placebo$ or single blind$ or double blind$ or triple blind$).ti,ab. 1186604
3 (retraction of publication or retracted publication).pt. 14645
4 or/1-3 1298384
5 (animals not humans).sh. 4615830
6 ((comment or editorial or meta-analysis or practice-guideline or review or letter) not "randomized controlled trial").pt. 4409095
7 (random sampl$ or random digit$ or random effect$ or random survey or random regression).ti,ab. not "randomized controlled trial".pt. 84751
8 4 not (5 or 6 or 7) 951334
9 "european journal of vascular & endovascular surgery".jn. 6572
10 limit 9 to yr="2008 - 2018" 3173
11 8 and 10 227
UPDATE:
12 limit 9 to yr="2019" 365
13 8 and 12 22
TOTAL: 249

© 2021 Robinson NB et al. *JAMA Network Open*.

**Journal of Vascular Surgery**

| | | |
|---|---|---|
| 1 | "randomized controlled trial".pt. | 495639 |
| 2 | (random$ or placebo$ or single blind$ or double blind$ or triple blind$).ti,ab. | 1186604 |
| 3 | (retraction of publication or retracted publication).pt. | 14645 |
| 4 | or/1-3 | 1298384 |
| 5 | (animals not humans).sh. | 4615830 |
| 6 | ((comment or editorial or meta-analysis or practice-guideline or review or letter) not "randomized controlled trial").pt. | 4409095 |
| 7 | (random sampl$ or random digit$ or random effect$ or random survey or random regression).ti,ab. not "randomized controlled trial".pt. | 84751 |
| 8 | 4 not (5 or 6 or 7) | 951334 |
| 9 | "journal of vascular surgery".jn. | 15822 |
| 10 | limit 9 to yr="2008 - 2018" | 7168 |
| 11 | 8 and 10 | 377 |
| UPDATE: | | |
| 12 | limit 9 to yr="2019" | 765 |
| 13 | 8 and 12 | 34 |
| TOTAL: 411 | | |

Footnote: To ensure the results were comprehensive to January 1, 2020, an update was run on August 18, 2020 for complete 2019 published articles; additional results were also screened.

© 2021 Robinson NB et al. *JAMA Network Open*.

**eTable 2**: Major and minor clinical endpoints

| | Primary endpoint |
|---|---|
| **Major** | • Reopération for meniscus surgery<br>• Atrial fibrillation<br>• Bowel obstruction<br>• Cerebrospinal fluid leak or fistula<br>• Need for device removal<br>• Disease free survival<br>• Need for fasciotomy<br>• Reintervention<br>• Hernia recurrence/occurrence<br>• Brain death<br>• Acute respiratory distress syndrome<br>• Locoregional recurrence<br>• Major surgical morbidity (Accordion grade or Clavien-Dindo Classification)<br>• Mortality<br>• Myocardial infarction<br>• Need for new renal replacement therapy<br>• Chylothorax<br>• Pancreatic fistula<br>• Pneumonia<br>• Pregnancy by natural conception<br>• Recurrence of pneumothorax<br>• Reoperation<br>• Repeat coronary revascularization<br>• Shunt failure<br>• Surgical site complication<br>• Stroke<br>• Surgical site infection |
| **Minor** | • Active knee flexion and extension as measured with a goniometer<br>• American Knee Society (AKS) knee and function scores<br>• American Orthopaedic Foot & Ankle Society (AOFAS) ankle-hindfoot scale<br>• American Shoulder and Elbow Surgeons (ASES) elbow score<br>• Dysesthesia<br>• Assessment of clipping attempts from a sample of video recordings<br>• Athletic shoulder outcome score<br>• Axial, coronal, and sagittal component positioning on computed tomographic scan<br>• Bile duct stenosis<br>• Bishop Score<br>• Blood transfusion<br>• Bone mineral density as measured by dual x-ray absorptiometry (DXA) scan |

© 2021 Robinson NB et al. *JAMA Network Open*.

| | |
|---|---|
| | • Cardiac biomarker levels (Troponin I, CK-MB etc.)<br>• Changes in left ventricular dimensions on echocardiography<br>• Cincinnati knee score<br>• Compensatory hyperhidrosis<br>• Composite evacuation scores<br>• Constant score<br>• Cross clamp and cardiopulmonary bypass times<br>• Cuff integrity assessed by magnetic resonance imaging<br>• Daniels one leg hop test<br>• Delayed gastric emptying<br>• Diarrhea<br>• Disability arm shoulder and hand score<br>• Duration of air leak<br>• Duration of surgery<br>• Echocardiographic indicators of RV global and overall systolic function<br>• Embolic burden based on intraoperative transesophageal echocardiography (TEE) at the time of intramedullary rod insertion and after tourniquet release<br>• Esophageal acid exposure at 3 years after surgery<br>• Excessive BMI loss (EBMIL)<br>• Femoral graft bending angles and femoral tunnel geometry on imaging<br>• Gartland and Werley point system<br>• Gastrointestinal (GI) Symptoms Score<br>• Graft patency as assessed by angiography<br>• Growth of the thyroid remnant as measured by ultrasonography<br>• Harners vertical jump test<br>• Harris hip score<br>• Hemorrhoid recurrence<br>• Hip Outcome Score<br>• Imaging measurement of bony ingrowth<br>• Improvement in dyschesia<br>• Incidence of dislocation of hip<br>• Increase in disc height<br>• Indigestion subscore of the Gastrointestinal Symptom Rating Scale questionnaire (GSRS)<br>• International Knee Documentation Committee score<br>• Japanese Orthopedic Association score<br>• Knee arthrometer testing<br>• Knee Injury and Osteoarthritis Outcome Score (KOOS)<br>• Knee pain after exercise<br>• Korean shoulder score<br>• Kujala score<br>• Left ventricular end systolic volume index<br>• Length of stay<br>• Length of time to drain removal |

© 2021 Robinson NB et al. *JAMA Network Open*.

| | |
|---|---|
| | • Levels of inflammatory markers (IL-6, IL-8, IL-10, TNF-alpha, IFN-gamma etc.)<br>• Lower Extremity Measure (LEM)<br>• Lysholm and Western Ontario Meniscal Evaluation Tool (WOMET) scores<br>• McGowan and Dellon Score<br>• Mean and maximum gradients and indexed effective orifice area (EOAI)<br>• Measurement of the diffusing capacity of the lung for carbon monoxide (DLCO)<br>• Medical Outcomes Study Health Survey Short Form-36 (SF-36) scores<br>• Menorrhagia multi-attribute quality of life scale<br>• Micromotion measured with radiostereometric analysis<br>• Narcotic prescription and consumption<br>• Noncompliance rate of lymph node dissection<br>• Number of high-intensity transient signals (HITS) detected during transcranial doppler (TCD) ultrasonography<br>• Number of microembolic signals recorded by transcranial Doppler<br>• Nurick score<br>• Occurrence of moderate to severe (≥3+) functional tricuspid regurgitation (TR)<br>• Operative cost<br>• Oswestry Disability Index<br>• Oxford knee score<br>• Oxygen saturation and blood flow at the presternal and retrosternal sides in the upper, middle, and lower parts<br>• Oxygenation as assessed by arterial blood gas analysis<br>• Pelvic organ prolapse quantification score<br>• Primary patency<br>• Positive margin status<br>• Quality of life<br>• Quality of surgery (graded by circumferential resection margin, mesorectum grade, and lymph node yield)<br>• Range of motion<br>• Rate of meniscus healing in the 2 groups assessed during a second-look arthroscopy<br>• Reactive oxygen species induction levels in granulocytes<br>• Readmission<br>• Reduction in the number of patient comorbidities<br>• Residual graft volume<br>• Resumption of antisecretory medication<br>• Rotational stability as assessed by the pivot-shift test<br>• Rowe score<br>• Saphenous vein intimal hyperplasia<br>• Semen analysis and scrotal ultrasonography to assess fertility<br>• Short form 12 (SF-12) |

© 2021 Robinson NB et al. *JAMA Network Open*.

| | |
|---|---|
| | • Simple shoulder score<br>• Single leg hop test<br>• Staple height scores<br>• Sternal healing based on computed tomorgraphy evaluation<br>• Success of left atrial appendage closure including no remnant pouch >1 cm in maximum length after closure (stump) and the absence of a color flow jet<br>• Tegner activity score<br>• leakage, defined as cerebrospinal fluid (CSF) collection or any open CSF fistula within 30 days<br>• Time off work<br>• Time to achieve complete liver mobilization at second hepatectomy<br>• Transient postoperative hypoparathyroidism<br>• Tunnel widening as assess by computed tomography or magnetic resonance imaging<br>• Urinary function<br>• Validated cosmesis and body image scoring<br>• Valvular hemodynamics and orifice size on echocardiography<br>• Visual analog score<br>• Zurich Claudication Questionnaire (ZCQ) |

Footnote: In trials using a composite outcome, if any major clinical event was included in the composite the outcome was classified as major.

© 2021 Robinson NB et al. *JAMA Network Open*.

**eTable 3**: Details of the trials included in the analysis (ordered by journal).

| Trial number | Name of trial | Year | Journal | Aim | Primary outcome | Primary results | Number of patients screened | Number of patients included |
|---|---|---|---|---|---|---|---|---|
| **1** | Aull et al[17] | 2014 | American Journal of Transplantation | To assess the benefits of laparoendoscopic single site donor nephrectomy (LESS-DN) over laparoscopic donor nephrectomy (LDN) | Mean/median number of days postsurgery required for each group to return to 100% functioning capacity | Questionnaires revealed that 97.2% of LESS-DN versus 79.5% of LDN (p=0.03) were 100% recovered by 2 months after donation | 105 | 105 |
| **2** | ALCCaS[18] | 2008 | Annals of Surgery | To compare laparoscopic and open colectomy | Disease-free survival and overall survival 3 and 5 years after surgery | Not reported (interim analysis) | 601 | 601 |
| **3** | Fein et al[19] | 2008 | Annals of Surgery | To compare Roux-en-Y reconstruction with and without jejunal pouch in terms of quality of life | Quality of life | In the first postoperative year, there were no benefits of pouch reconstruction in terms of quality of life, independent of the resection status. In the third, fourth, and fifth year after surgery quality of life was significantly improved for patients with a pouch | 138 | 138 |
| **4** | Fernandez-Cruz et al[20] | 2008 | Annals of Surgery | To compare the results of postoperative morbidity rate of a new pancreatogastrostomy technique, pylorus-preserving pancreaticoduodenectomy (PPPD) with gastric partition (PPPD-GP) with the conventional technique of pancreaticojejunostomy (PJ) | Pancreatic fistula rates, and severity of pancreatic fistula | The overall postoperative complications were 23% after PPPD-GP and 44% after PPPD-PJ (P<0.01). The incidence of pancreatic fistula was 4% after PPPD-GP and 18% after PPPD-PJ (P<0.01). The mean + SD hospital stay was 12±2 days after PPPD-GP and 16±3 days after PPPD-PJ | 108 | 108 |
| **5** | GIVOM[21] | 2008 | Annals of Surgery | To assess the efficacy and safety of sentinel lymph node (SLN) biopsy compared with axillary lymph node dissection (ALND) | Disease-free survival (DFS) | At a median follow-up of 56 months, there were more locoregional recurrences in the SLN arm, and the 5-year disease-free survival was 89.9% in the ALND arm and 87.6% in the SLN arm, with a difference of 2.3% (95% confidence interval: -3.1% to 7.6%) | 749 | 749 |
| **6** | Hayashi et al[22] | 2008 | Annals of Surgery | To assess whether the use of a sodium hyluronate-based bioresorbable membrane reduces small bowel obstruction after gastrectomy for gastric cancer | Incidence of bowel obstruction | The overall incidence (Seprafilm group, 5.7% vs. control group, 9.5%; P=0.534) and the cumulative incidence of small bowel obstruction (6.2% vs. 12.2% at 36 months; P=0.3789) were slightly but not significantly lower in the Seprafilm group | 207 | 150 |
| **7** | Karamanakos et al[23] | 2008 | Annals of Surgery | To evaluate and compare the effects of laparoscopic Roux-en-Y gastric bypass (LRYGBP) with laparoscopic sleeve gastrectomy (LSG) on body weight, appetite, fasting, and | Weight loss | Excess weight loss was greater after LSG at 6 months (55.5%±7.6% vs. 50.2%±6.5%, P=0.04) and 12 months (69.7%±14.6% vs. 60.5%±10.7%, (P=0.05)) | 32 | 32 |

© 2021 Robinson NB et al. *JAMA Network Open*.

| | | | | | | | | |
|---|---|---|---|---|---|---|---|---|
| | | | | postprandial ghrelin and peptide-<br>YY (PYY) levels | | | | |
| **8** | Kim et al[24] | 2008 | Annals of Surgery | To evaluate the quality of life (QOL) after laparoscopy-assisted distal gastrectomy (LADG) compared with open distal gastrectomy (ODG) in patients with early gastric cancer | 5-year disease-free survival (DFS) | Not reported (interim analysis) | 183 | 164 |
| **9** | Koh et al[25] | 2008 | Annals of Surgery | To investigate the safety and efficacy of the no-tie (NT) technique using the harmonic scalpel (HS) in terms of the operating time and complications in total thyroidectomy with central neck dissection (CND) | Postoperative complications | No significant difference was observed in the overall perioperative complications, such as postoperative bleeding, temporary recurrent laryngeal nerve palsy, and temporary hypoparathyroidism, between the 2 groups | 65 | 65 |
| **10** | Ikeda et al[26] | 2009 | Annals of Surgery | To verify the new surgical device (the LigaSure vessels sealing system) decrease liver transection time | Liver transection time | The median liver transection time in the VS group was 57 minutes (range: 11–127), similar to that in the CC group (56 (range: 9–269) min, P=0.64), while there was no difference in the transection speed between the 2 groups (1.16 (0.15–2.26) cm2/min vs. 1.10 (0.15–2.66) cm2/min, P=0.95) | 165 | 120 |
| **11** | INSECT[27] | 2009 | Annals of Surgery | To compare: (1) interrupted technique with rapidly absorbable sutures and (2) continuous techniques with different slowly absorbable sutures, focusing on the incidence of incisional hernias within 1 year | The frequency of incisional hernias diagnosed by clinical examination and confirmed by ultrasound | The primary analysis showed an incidence of 28 incisional hernias (15.9%) versus 15 (8.4%) versus 22 (12.5%) for the 3 closure techniques, respectively (P=0.09) | 635 | 625 |
| **12** | Kouhia et al[28] | 2009 | Annals of Surgery | To compare the Lichtenstein hernioplasty with a totally extraperitoneal preperitoneal laparoscopic technique (TEP) in treatment of recurrent inguinal hernias | Hernia recurrence and chronic pain | Recurrence rates were 3 in the Lichtenstein group and none in the TEP group (6.4% versus 0.0%, respectively), but this difference was statistically not significant. Chronic pain was more prevalent in the Lichtenstein group compared with the TEP group (13 (27.7%) versus 4 (8.2%) patients, respectively, P=0.02) | 99 | 96 |
| **13** | Nguyen et al[29] | 2009 | Annals of Surgery | To compare the outcomes, quality of life, and costs of laparoscopic gastric bypass versus laparoscopic gastric banding | Perioperative and late outcomes, weight loss, quality of life, and costs in the 2 groups | Compared with gastric banding, operative blood loss was higher and the mean operative time and length of stay were longer in the gastric bypass group. The 30-day complication rate was higher after gastric bypass (21.6% vs. 7.0% for gastric band); however, there were no life-threatening complications such as leaks or sepsis. The most frequent late complication in the gastric bypass | 250 | 250 |

© 2021 Robinson NB et al. *JAMA Network Open*.

| | | | | | | | | |
|---|---|---|---|---|---|---|---|---|
| | | | | | | group was stricture (14.3%). The 1-year mortality was 0.9% for the gastric bypass group and 0% for the gastric band group. The percent of excess weight loss at 4 years was higher in the gastric bypass group (68±19% vs. 45±28%, respectively, P < 0.05). Treatment failure occurred in 16.7% of the patients who underwent gastric banding and in 0% of those who underwent gastric bypass, with male gender being a predictive factor for poor weight loss after gastric banding. At 1-year postsurgery, quality of life improved in both groups to that of US norms. The total cost was higher for gastric bypass as compared with gastric banding procedure ($12,310 vs. $10,766, respectively, P < 0.01) | | |
| **14** | Peterli et al[30] | 2009 | Annals of Surgery | To evaluate and compare the effects of laparoscopic Roux-en-Y gastric bypass (LRYGB) with those of laparoscopic sleeve gastrectomy (LSG) on fasting, and meal-stimulated insulin, glucose, and glucagon-like peptide-1 (GLP-1) levels | Effectiveness of weight loss, reduction in co morbidies, quality of life at 5 years | Excess BMI loss was similar at 3 months (43.3±12.1% vs. 39.4±9.4%, P>0.36) | 27 | 27 |
| **15** | POVATI[31] | 2009 | Annals of Surgery | To compare both midline and transverse abdominal incisions by focusing on postoperative pain, complications, and frequency of incisional hernias | Abdominal pain intensity experienced by the patient and the amount of analgesic drugs used | Both incision types resulted in similar amounts of required analgesics (95% confidence interval (-0.38; -0.33) was included in the equivalence level). For the Visual Analogue Scale, both the 95% and 90% CI (0 –10) were neither within the equivalence levels nor were their differences significant at the 5% level | 1188 | 200 |
| **16** | Serra-Aracil et al[32] | 2009 | Annals of Surgery | To reduce the incidence of PH by implanting a lightweight mesh in the sublay position | Incidence of parastomal hernia | In the clinical follow-up (median: 29 months, range: 13–49), 11/27 (40.7%) hernias were recorded in the control group compared with 4/27 (14.8%) in the study group (P=0.03) | 44 | 44 |
| **17** | SIGMA[33] | 2009 | Annals of Surgery | To test the hypothesis that LSR is associated with decreased postoperative complication rates as compared with OSR | Postoperative mortality, and complications classified as major and minor | Mortality rate was 1%. There were significantly more major complications in OSR patients (9.6% vs. 25.0%; P=0.038). Minor complication rates were similar (LSR 36.5% vs. OSR 38.5%; P=0.839) | 140 | 104 |
| **18** | Weiss et al[34] | 2009 | Annals of Surgery | To assess biliary complications after liver transplantation using a choledochocholedochostomy with or without a temporary T-tube | Incidence of biliary complications after liver transplantation | The overall biliary complication rate was significantly increased in group 2 (P<0.0005). Biliary leaks occurred in 5 patients in group 1 and in 9 patients in group 2 (5.05% vs. 9.47%; P=0.2756 ns). Anastomotic strictures of the bile duct were seen in 7 patients in group 1 and in 8 patients in group 2 (7.07% vs. 8.42%; P=0.7923 ns). Two of the patients in group 1 and 5 patients | 194 | 194 |

© 2021 Robinson NB et al. *JAMA Network Open*.

| | | | | | | | | |
|---|---|---|---|---|---|---|---|---|
| | | | | | | in group 2 developed an ischemic type biliary lesion (2.02% vs. 5.26%; P=0.2716) | | |
| **19** | Aboulian et al[35] | 2010 | Annals of Surgery | To investigate if laparoscopic cholecystectomy performed within 48 hours of admission for mild gallstone pancreatitis, regardless of resolution of abdominal pain or abnormal laboratory values, would result in a shorter hospital stay | Hospital length of stay | The hospital length of stay was shorter for the early cholecystectomy group (mean: 3.5 (95% CI, 2.7– 4.3), median: 3 (IQR, 2–4)) compared with the control group (mean: 5.8 (95% CI, 3.8 –7.9), median: 4 (IQR, 4–6) (P=0.0016)) | 84 | 50 |
| **20** | Daraï et al[36] | 2010 | Annals of Surgery | To report the first randomized trial of laparoscopically assisted versus open colorectal resection for endometriosis focusing on perioperative complications, improvement in symptoms, quality of life, and fertility | Improvement in dyschesia | No difference in the symptom delta values and quality of life was noted between the groups. A significant improvement was seen in all digestive and gynecologic symptoms in the overall group | 76 | 52 |
| **21** | Gervaz et al[37] | 2010 | Annals of Surgery | To compare open and laparoscopic sigmoid resection for diverticulitis with the patient and the nursing staff blinded to the surgical approach | The primary endpoints for analysis were (1) postoperative pain; (2) duration of postoperative ileus; and (3) duration of hospital stay | The median delay between surgery and first bowel movement was 76 (range, 31–163) hours in the laparoscopy group versus 105 (range, 53–175) hours in the open group (P < 0.0001). The median score for maximal pain (assessed by a visual analog scale) was 4 (range, 1–10) in the laparoscopy group and 5 (range, 1–10) in the open group (P < 0.05). Finally, the median duration of hospital stay was 5 days (range, 4–69) in the laparoscopy group versus 7 days (range, 5–17) in the open group (P < 0.0001) | 150 | 132 |
| **22** | KLASS[38] | 2010 | Annals of Surgery | To evaluate the safety of laparoscopic distal gastrectomy with respect to morbidity and mortality | Overall survival | The postoperative mortality was 1.1% (2/179) and 0% (0/163) in the LADG and ODG groups (P= 0.497) | NR | 342 |
| **23** | LEVEL[39] | 2010 | Annals of Surgery | To compare the most common technique for open mesh repair (Lichtenstein) with the currently preferred minimally invasive technique (total extra peritoneal, TEP) for the surgical correction of inguinal hernia | Postoperative pain, length of hospital stay, period until complete recovery, and quality of life (QOL) | TEP was associated with less postoperative pain until 6 weeks postoperatively (P=0.01). Chronic pain was comparable (25% vs. 29%). Less impairment of inguinal sensibility was seen after TEP (7% vs. 30%, P=0.01). Incidence of adverse events during surgery was higher with TEP (5.8% vs. 1.6%, P=0.004), but postoperative complications (33% vs. 33%), hospital stay and QOL were similar. After TEP, patients had a faster recovery of daily activities (ADL) and less absence from work (P=0.01) | 722 | 660 |
| **24** | Peeters et al[40] | 2010 | Annals of Surgery | To compare quality of life and fertility aspects after laparoscopic | Fertility aspects, assessed preoperatively and at 1-year follow- up by semen | Patients operated on with a VyproII or TiMesh mesh exhibited a decreased sperm motility (vs. preoperatively) compared with Marlex patients, | 351 | 59 |

© 2021 Robinson NB et al. *JAMA Network Open*.

| | | | | | | | | |
|---|---|---|---|---|---|---|---|---|
| | | | | inguinal hernia repair in men using a heavyweight or lightweight mesh | analysis and scrotal ultrasonography | respectively -9.5% and -5.5% versus +2% (P =0.013) | | |
| **25** | Kawai et al[41] | 2011 | Annals of Surgery | To determine whether pylorus-resecting pancreatoduodenectomy (PrPD) with preservation of nearly the entire stomach reduces the incidence of delayed gastric emptying (DGE) compared with pylorus-preserving pancreatoduodenectomy (PpPD) | DGE according to ISGPS definition | The incidence of DGE was 4.5% in PrPD and 17.2% in PpPD, a significant difference | 139 | 130 |
| **26** | Ma et al[42] | 2011 | Annals of Surgery | To compare short-term surgical outcomes and quality of life (QOL) between single-port laparoscopic cholecystectomy (SPLC) and classic 4-port laparoscopic cholecystectomy (CLC) | Postoperative pain scores at hospital discharge and at the initial postoperative follow visit using a standard 10-point visual analog scale (VAS) | Overall and cosmetic satisfaction, QOL as determined by the SF-36 survey, postoperative complications, and postoperative pain scores between discharge and 2-week postoperative visit were not significantly different between groups | NR | 43 |
| **27** | Chen et al[43] | 2012 | Annals of Surgery | To compare the efficacy and safety between apical pleurectomy and pleural abrasion with minocycline in primary spontaneous pneumothorax (PSP) with high recurrence risk | Rate of ipsilateral recurrence after the operation | After a mean follow-up of 26.1 months, recurrent ipsilateral pneumothorax occurred in 3 patients (3.8%) in the pleurectomy group and 3 patients (3.8%) in the abrasion/minocycline group | 369 | 160 |
| **28** | Hamilton et al[44] | 2012 | Annals of Surgery | To compare stapled left pancreatectomy with stapled left pancreatectomy using mesh reinforcement of the staple line with either Seamguard or Peristrips Dry | Clinically significant leak as defined by ISGPF | ISGPF grade B and C leaks were seen in 1.9% (1/53) of patients undergoing resection with mesh reinforcement and 20% (11/45) of patients without mesh reinforcement (P = 0.0007) | 148 | 100 |
| **29** | HASTA[45] | 2012 | Annals of Surgery | To compare hand suture versus stapling loop ileostomy closure in a randomized controlled trial | Bowel obstruction within 1 month after ileostomy closure, was defined as productive vomiting or the need of gastric tube placement or absolute constipation with duration of at least 3 days | The overall rate of postoperative ileus after ileostomy closure was 13.4%. Seventeen of 165 (10.3%) patients in the stapler group and 27 of 163 (16.6%) in the hand suture group developed bowel obstruction within 30 days postoperatively (odds ratio (OR) = 1.72; 95% confidence interval (CI): 0.89–3.31 = 0.10) | 337 | 334 |
| **30** | Oberkofler et al[46] | 2012 | Annals of Surgery | To evaluate the outcome after Hartmann's procedure versus primary anastomosis with diverting ileostomy for | Overall complication rate | The overall complication rate for both resection and stoma reversal operations was comparable (80% vs 84%, P = 0.813) | 83 | 62 |

© 2021 Robinson NB et al. *JAMA Network Open*.

| | | | | | | | | |
|---|---|---|---|---|---|---|---|---|
| | | | | perforated left-sided diverticulitis | | | | |
| **31** | Sancho et al[47] | 2012 | Annals of Surgery | To assess the immediate and long-term clinical results of 2 different surgical procedures for the treatment of asymmetrical multinodular goiter (AMG) | Growth of the thyroid remnant as measured by ultrasonography | Remnant growth (20 vs 0%; P < 0.001), appearance of new nodules (55 vs 14%; P < 0.001), and overall reoperation rate (9.2 vs 1.8%, P = 0.2) were more common in HMT, mostly because of undiagnosed cancer requiring completion thyroidectomy | 142 | 118 |
| **32** | Teoh et al[48] | 2012 | Annals of Surgery | To perform a multicentered prospective double-blinded randomized controlled trial comparing laparoendoscopic single-site access (LESS) versus conventional three-port laparoscopic appendectomy (TPLA) | Overall pain score | There were no significant differences in the morbidity rates, operative time, conversion rates, and postoperative recovery. There were also no differences in the overall pain score and pain score at rest | 278 | 200 |
| **33** | TIMELI[49] | 2012 | Annals of Surgery | To test the hypothesis that fibrin sealant mesh fixation can reduce the incidence of postoperative pain/numbness/groin discomfort by up to 50% compared with sutures for repair of inguinal hernias using the Lichtenstein technique | Composite that evaluated the prevalence of chronic disabling complications (VAS score >30 for pain/numbness/groin discomfort) at 12 months after surgery | Less pain was reported in the fibrin sealant group than in the sutures group at 1 and 6 months (P = 0.0132; P = 0.0052), as reflected by a lower proportion of patients using analgesics in the fibrin group over the study duration (65.2% vs 79.7%; P = 0.0009) | 325 | 319 |
| **34** | Tzovaras et al[50] | 2012 | Annals of Surgery | To compare the LERV 1-stage approach with the standard 2-stage approach consisting of preoperative ERCP followed by laparoscopic cholecystectomy for the treatment of cholecysto-choledocholithiasis | Overall hospital stay | Hospital stay was significantly shorter in the LERV group; median 4 (2–19) days versus 5.5 (3–22) days, P = 0.0004 | 112 | 100 |
| **35** | Uzunoglu et al[51] | 2012 | Annals of Surgery | To assess the potential benefits of ultrasonic energy dissection compared with conventional dissection techniques in pancreatic surgery | Overall duration in operative time | The use of an ultrasonic dissection device did not significantly reduce overall operation time (median 316 minutes in group A and 319 minutes in group B, P = 0.95) and did not significantly increase the costs of surgery | 255 | 255 |
| **36** | ACDC[52] | 2013 | Annals of Surgery | To determine the optimal timing of surgery for acute cholecystitis | Morbidity- defined as the occurrence of any clinically relevant complication on a score sheet | Morbidity rate was significantly lower in group immediate lalaprscopuc cholecystectomy (304 patients) than in group delayed laparoscopic cholecystectomy (314 patients): 11.8% versus 34.4% | 642 | 618 |
| **37** | Boelens et al[53] | 2013 | Annals of Surgery | To clarify the role of a surgical neurectomy on pain in refractory | Proportion of patients achieving a minimal 50% improvement in pain perception measured using a VAS (0 mm (pain | In the neurectomy group, 16 patients reported a successful pain response. In contrast, significant pain reduction was obtained in 4 patients in the sham group (P = 0.001) | 126 | 44 |

© 2021 Robinson NB et al. *JAMA Network Open*.

| | | | | | | | | |
|---|---|---|---|---|---|---|---|---|
| | | | | patients after conservatively treated anterior cutaneous nerve entrapment syndrome | absent) to 100 mm (excruciating pain)), and/or an at least 2-point improvement on a VRS (score 0–4, 0 = no pain; 4 = severe pain) at the 6 weeks time point postoperatively compared with the preoperative VAS and VRS | | | |
| **38** | Frutos et al[54] | 2013 | Annals of Surgery | To compare single incision laparoscopic cholecystectomy with conventional laparoscopic cholecystectomy | Complication rate | There were no significant differences between the 2 groups for early and late complications and lengths of hospital stay measured in postoperative hours | NR | 184 |
| **39** | Lee et al[55] | 2013 | Annals of Surgery | To compare surgical outcomes and quality of life between single port laparoscopic appendectomy (SPLA) and conventional laparoscopic appendectomy (CLA) in patients with acute appendicitis | Overall postoperative complication rates and postoperative pain scores | There was no significant difference in the overall complication rate (P = 0.470). Postoperative pain scores were not significantly different between 2 groups | 657 | 248 |
| **40** | López-Andújar et al[56] | 2013 | Annals of Surgery | To compare the incidence and severity of biliary complications due to liver transplantation after choledochocholedochostomy with or without a T-tube in a single-center, prospective, randomized trial | Biliary complications | The overall biliary complication rate was 22.5% (n = 42), with no difference between groups (P = 0.35) | 233 | 200 |
| **41** | ProLOVE[57] | 2013 | Annals of Surgery | To compare laparoscopic technique with open technique regarding short-term pain, quality of life (QoL), recovery, and complications | Pain at 3 weeks, measured as the bodily pain subscale of Short Form-36 | SF-36 subscales favored the LR group: physical function (P<0.001), role physical (P<0.012), mental health (P<0.022), and physical composite score (P < 0.009). Surgical site infections were 17 in the OR group compared with 1 in the LR group (P < 0.001) | NA | 157 |
| **42** | SEPRAC2T[58] | 2013 | Annals of Surgery | To assess feasibility and efficacy of using a bioresorbable hyaluronic acid/carboxymethylcellulose membrane (HA membrane) to prevent abdominal and perihepatic adhesions in metastatic colorectal cancer patients requiring 2-stage hepatectomy | Time to achieve complete liver mobilization at second hepatectomy calculated as the sum of the time from the start of abdominal incision to the positioning of the retractors and the time from positioning of the retractors to complete | At second hepatectomy, patients in the HA membrane arm required 33% less time than controls to achieve complete liver mobilization (median: 50 vs 75 minutes; primary endpoint) | 60 | 54 |

© 2021 Robinson NB et al. *JAMA Network Open*.

| | | | | | | | | |
|---|---|---|---|---|---|---|---|---|
| | | | | | section of liver ligaments permitting full mobilization of both liver lobes | | | |
| **43** | Shimoda et al[59] | 2013 | Annals of Surgery | To evaluate the superiority of Billroth II (B-II) to Roux-en Y (R-Y) reconstruction on decreasing the incidence of delayed gastric emptying DGE after SSPPD | DGE according to ISGPS definition | DGE occurred in 5.7% of patients in the B-II group and in 20.4% of patients in the R-Y group (P = 0.028) | 115 | 101 |
| **44** | SM-BOSS[60] | 2013 | Annals of Surgery | To assess the effectiveness and safety of laparoscopic sleeve gastrectomy (LSG) and laparoscopic Rouxen-Y gastric bypass (LRYGB) | Weight loss, which was defined by excessive BMI loss (EBMIL), over a period of 5 years | Excessive body mass index loss 1 year after the operation was similar between the 2 groups (72.3% ± 22% for LSG and 76.6% ± 21% for LRYGB; P = 0.2) | NR | 217 |
| **45** | ARCO[61] | 2014 | Annals of Surgery | To investigate the relationship between the route of gastroenteric (GE) reconstruction after pancreatoduodenectomy (PD) and the postoperative incidence of delayed gastric emptying (DGE) | DGE by ISGPS consensus definition | In the retrocolic group, 45 patients (36%) developed clinically relevant DGE compared with 41 (34%) in the antecolic group (absolute risk difference: 2.1%; 95% confidence interval: −9.8% to 14.0%) | 353 | 252 |
| **46** | Barczynski et al[62] | 2014 | Annals of Surgery | To test if posterior retroperitoneoscopic adrenalectomy (PRA) is superior to lateral transperitoneal laparoscopic adrenalectomy (LTLA) | Duration of surgery | The following differences were identified in favor of PRA vs LTLA: shorter duration of surgery (50.8 vs 77.3 minutes), lower intraoperative blood loss (52.7 vs 97.8 mL), diminished pain intensity within 48 hours postoperatively, lower prevalence of shoulder-tip pain (3.0% vs 37.5%), shorter time to oral intake (4.4 vs 7.3 hours), shorter time to ambulation (6.1 vs 11.5 hours), shorter length of hospital stay, and lower cost (1728 € vs 2315 €), respectively (P < 0.001 for all) | 88 | 65 |
| **47** | Denost et al[63] | 2014 | Annals of Surgery | To evaluate if perineal dissection of the distal rectum may improve quality of surgery, compared with the conventional abdominal dissection | Quality of surgery (circumferential resection margin, mesorectum grade, and lymph nodes). | The rate of positive circumferential resection margin decreased significantly after perineal compared with abdominal low rectal dissection, 4% versus 18% (P = 0.025). The mesorectum grade and the number of lymph nodes analyzed did not differ between the 2 groups | 392 | 100 |
| **48** | JCOG 0404[64] | 2014 | Annals of Surgery | To confirm the non-inferiority of laparoscopic surgery to open surgery in terms of overall survival was conducted, and short-term surgical outcomes are demonstrated | Overall survival | Not reported (interim analysis) | NR | 1057 |

© 2021 Robinson NB et al. *JAMA Network Open*.

| | | | | | | | | |
|---|---|---|---|---|---|---|---|---|
| **49** | Melissa et al[65] | 2014 | Annals of Surgery | To compare the clinical outcomes of mesh fixation with fibrin sealant (FS) spray or mechanical stapling (MS) in laparoscopic total extraperitoneal hernioplasty (TEP) | Incidence of chronic pain at 6 months follow up | At 6 months, no significant differences in the incidence of chronic pain were observed (at rest, after coughing or cycling) | 363 | 130 |
| **50** | Nicol et al[66] | 2014 | Annals of Surgery | To determine if stable patients with a hemopericardium detected after penetrating chest trauma can be safely managed with pericardial drainage alone | Survival to discharge from hospital | There was 1 death postoperatively among the 111 patients (0.9%) and this was in the sternotomy group | 191 | 111 |
| **51** | Van Buren et al[67] | 2014 | Annals of Surgery | To test by randomized prospective multicenter trial the hypothesis that pancreaticoduodenectomy (PD) without the use of intraperitoneal drainage does not increase the frequency or severity of complications | 60-day grade II or greater complication rate | PD without intraperitoneal drainage was associated with an increase in the number of complications per patient (1 (0-2) vs 2 (1-4), P = 0.029); an increase in the number of patients who had at least 1 ≥grade 2 complication (35 (52%) vs 47 (68%), P = 0.047); and a higher average complication severity (2 (0-2) vs 2 (1-3), P = 0.027) | 357 | 282 |
| **52** | FinnMesh[68] | 2015 | Annals of Surgery | To find out how to perform a safe and cost-effective open inguinal hernioplasty in day-case setting with the best outcomes with regard to chronic pain | Sensation of pain measured by pain scores and need for analgesics after 1 year | There were no significant differences postoperatively in pain response or need for analgesics between the study groups | 650 | 625 |
| **53** | Garcia-Urena et al[69] | 2015 | Annals of Surgery | To reduce the incidence of incisional hernia (IH) in colorectal surgery by implanting a mesh on the overlay position | Incisional hernia at 2 years | The incidence of IH was 17 of 54 (31.5%) in the control group and 6 of 53 (11.3%) in the study group (P = 0.011) | 134 | 107 |
| **54** | MASH[70] | 2015 | Annals of Surgery | To compare Laparoscopic mesh-augmented hiatoplasty with cardiophrenicopexy (LMAH-C) with laparoscopic Nissen fundoplication (LNF) in patients with GERD | Indigestion subscore of the Gastrointestinal Symptom Rating Scale questionnaire (GSRS) | Indigestion subscore (LMAH-C 2.9±1.5 vs LNF 3.7±1.6; P=0.031) but not dysphagia (2.8±1.9 vs 2.3±1.7; P=0.302) and quality of life (106.9±25.5 vs 105.8±24.9; P=0.838) differed between the groups at 36 months postoperatively | 431 | 90 |
| **55** | NOTES[71] | 2015 | Annals of Surgery | To compare 3 trocar laparoscopic cholecystectomy with umbilical-assisted transvaginal cholecystectomy | Intensity of pain until the morning of postoperative day (POD) 2 | Significant advantages were found for the transvaginal access regarding pain until POD 2, but also until POD 10 (P = 0.043 vs P = 0.010) despite significantly less use of peripheral analgesics (P = 0.019) | NR | 40 |
| **56** | PRIMA[72] | 2015 | Annals of Surgery | To evaluate primary mesh augmentation to reduce incisional | Incisional hernia at 2 years | Not reported | 498 | 480 |

© 2021 Robinson NB et al. *JAMA Network Open*.

| | | | | | | | | |
|---|---|---|---|---|---|---|---|---|
| | | | | hernia incidence | | | | |
| **57** | SPOCC[73] | 2015 | Annals of Surgery | To evaluate cosmesis, body image, pain, and quality of life (QoL) after single-port laparoscopic cholecystectomy (SPLC) versus conventional 4-port laparoscopic cholecystectomy (4PLC) | Validated cosmesis and body image | The SPLC-group showed superior mean cosmesis and body image compared with the 4PLC-group at 12-weeks (21 vs 16, P<0.001 and 5 vs 6, P=0.013, respectively) and at 1-year (24 vs 16, P<0.001 and 5 vs 6, P<0.017, respectively) | 132 | 110 |
| **58** | Watson et al[74] | 2015 | Annals of Surgery | To determine whether absorbable or nonabsorbable mesh in repair of large hiatus hernias reduces the risk of recurrence, compared with suture repair | Hernia recurrence assessed by barium meal radiology and endoscopy at 6 months | A recurrent hernia (any size) was identified in 23.1% after suture repair, 30.8% after absorbable mesh, and 12.8% after non-absorbable mesh (P = 0.161) | NR | 126 |
| **59** | ANOSEAN[75] | 2016 | Annals of Surgery | To explore the impact of the absence of band fixation on the reoperation rate and to identify other risk factors for long term complications | Reintervention rate for band removal or repositioning at 3 years | At 3 years, the reintervention rate for band retrieval or repositioning was significantly higher in the absence of band fixation (19.4% vs11.3%; P=0.013), partly because of the slippage rate (10.3% vs 3.6%; P=0.005) | NR | 706 |
| **60** | DILALA[76] | 2016 | Annals of Surgery | To compare short-term results of laparoscopic lavage with the Hartmann procedure within a randomized trial | Reoperation within 12 months | There were no statistical differences between the 2 groups and neither did the number of complications per patient differ between the 2 groupsNot reported (iterim analysis) | 267 | 83 |
| **61** | DISCOVER[77] | 2016 | Annals of Surgery | To analyze the impact of teres ligament covering on pancreatic fistula rate after distal pancreatectomy (DP) | Rate of POPF | Clinically relevant POPF rate (grade B/C) was 32.9% (control) versus 22.4% (teres, P=0.20) | 436 | 161 |
| **62** | Han et al[78] | 2016 | Annals of Surgery | To compare the ligation of intersphincteric fistula tract (LIFT) with an additional plug (LIFT-plug) in the treatment of transsphincteric anal fistula | Success rate and healing time at 6 months | The primary healing rate was higher in LIFT-plug group than in LIFT group (94.0% (95% confidence interval 89.7%–98.3%) vs 83.9% (95% confidence interval 77.2%–90.6%), P<0.001) | 270 | 239 |
| **63** | Kawai et al[79] | 2016 | Annals of Surgery | To evaluate in a multicenter randomized controlled trial (RCT) whether pancreaticojejunostomy (PJ) of pancreatic stump decreases the incidence of pancreatic fistula after distal pancreatectomy (DP) compared with stapler closure | Incidence of POPF based on ISGPF grading | Pancreatic fistula occurred in 23 patients (37.7%) in the stapler closure group and 24 (38.7%) in the PJ group (P=0.332) in intention-to-treat analysis. The incidence of clinically relevant pancreatic fistula (grade B or C) was 16.4% for stapler closure and 9.7% for PJ (P=0.201). | 184 | 136 |
| **64** | Kim et al[80] | 2016 | Annals of Surgery | To compare the postoperative quality of life of vagus nerve preserving distal gastrectomy (VPG) vs conventional distal gastrectomy (CG) in patients with early-stage gastric cancer | Diarrhea | Patients assigned to VPG showed less diarrhea 3 and 12 months after surgery (P=0.040 and 0.048, respectively) and less appetite loss at 12 months (P=0.011) compared with those assigned to CG | 371 | 185 |

© 2021 Robinson NB et al. *JAMA Network Open*.

| | | | | | | | | |
|---|---|---|---|---|---|---|---|---|
| **65** | LigaLongo[81] | 2016 | Annals of Surgery | To compare Doppler-guided hemorrhoidal artery ligation (DGHAL) with circular stapled hemorrhoidopexy (SH) in the treatment of grade II/III hemorrhoidal disease (HD) | Morbidity at 90 days | At 90 days, operative-related adverse events occurred after DGHAL and SH, respectively, in 47 (24%) and 50 (26%) patients (P=0.70) | NR | 407 |
| **66** | Lopez-Cano et al[82] | 2016 | Annals of Surgery | To assess the reduction in the incidence of parastomal hernia (PH) after placement of prophylactic synthetic mesh using a modified Sugarbaker technique when a permanent end-colostomy is needed | Parastomal hernia formation as demonstrated by CT scan | After CT examination, 6 of 24 PHs (25%) were observed in the mesh group compared with 18 of 28 (64.3%) in the nonmesh group (odds ratio 0.39, 95% confidence interval 0.18–0.82; P=0.005) | 85 | 56 |
| **67** | PRIMAAT[83] | 2016 | Annals of Surgery | To evaluate the impact of prophylactic mesh placement on the incidence of incisional hernia following abdominal aortic aneurysm repair | Incidence of incisional hernias at 2-year follow-up | The cumulative incidence of incisional hernias at 2-year follow-up after conventional closure was 28% (95% confidence interval (CI), 17%–41%) versus 0% (95% CI, 0%–6%) after mesh-augmented reinforcement (P<0.0001; Fisher exact test) | 369 | 120 |
| **68** | RECOPANC[84] | 2016 | Annals of Surgery | To assess pancreatic fistula rate and secondary endpoints after pancreatogastrostomy (PG) versus pancreatojejunostomy (PJ) for reconstruction in pancreatoduodenectomy | POPF ISGPS grades B or C | There was no significant difference in the rate of grade B/C fistula after PG versus PJ (20% vs 22%, P=0.617). The overall incidence of grade B/C fistula was 21%, and the in-hospital mortality was 6% | 618 | 440 |
| **69** | Roulin et al[85] | 2016 | Annals of Surgery | To compare clinical outcomes of early versus delayed laparoscopic cholecystectomy (LC) in acute cholecystitis with more than 72 hours of symptoms | Morbidity following initial diagnosis | Overall morbidity was lower in ELC (6 (14%) vs 17 (39%) patients, P=0.015) | 375 | 86 |
| **70** | TOPAR[86] | 2016 | Annals of Surgery | To find robust estimates for the rates of recurrence of 2 surgical strategies for secondary hyperparathyroidism (SHPT) within 36 months of follow-up | Rates of recurrence | Recurrent SHPT developed in 4 total parathyroidectomies with autotransplantation and none of the totalparathyroidectomy-only patients | 198 | 100 |
| **71** | Westin et al[87] | 2016 | Annals of Surgery | To compare long-term postoperative pain after inguinal hernia surgery using 2 techniques that have shown favorable long-term outcome in previous randomized studies: Lichtenstein using local anesthesia (LLA) and | Long-term post-operative pain | In the TEP group, 39 (20.7%) patients experienced pain, compared with 62 (33.2%) patients in the LLA group (P=0.007). Severe pain was reported by 4 patients in the TEP group and 6 patients in the LLA group (2.1% and 3.2%, respectively, P=0.543). Pain in the operated groin limited the ability to exercise for 5 TEP patients and 14 LLA patients (2.7% and 7.5%, respectively, P=0.034) | 1998 | 389 |

© 2021 Robinson NB et al. *JAMA Network Open*.

| | | | | | | | | |
|---|---|---|---|---|---|---|---|---|
| | | | | endoscopic total extra-peritoneal repair (TEP) under general anesthesia | | | | |
| **72** | BIOPEX[88] | 2017 | Annals of Surgery | To determine the effect of biological mesh closure on perineal wound healing after extralevator abdominoperineal resection (eAPR) | Southampton wound score <2 at 30 days | Uncomplicated perineal wound healing rate at 30 days was 66% (33/50; 3 not evaluable) after primary closure, which did not significantly differ from 63% (30/48) after biological mesh closure (relative risk 1.056; 95% confidence interval (CI) 0.7854–1.4197; P=0.7177) | 117 | 104 |
| **73** | EASY[89] | 2017 | Annals of Surgery | To study morbidity and mortality associated with early closure (8–13 days) of a temporary stoma compared with standard procedure (closure after > 12 weeks) after rectal resection for cancer | Mean number of complications following index operation | The mean number of complications after index surgery up to 12 months follow up was significantly lower in the intervention group (1.2) compared with the control group (2.9), P < 0.0001 | 418 | 127 |
| **74** | FOREseal[90] | 2017 | A Annals of Surgery | To determine the efficacy of alginate staple-line reinforcement of fissure openings as compared with stapling alone, with or without tissue sealant or glue, in reducing the incidence and duration of air leakage after pulmonary lobectomy for malignancy | Duration of postoperative air leak (in days) | The primary endpoint of air leak duration was not different between the 2 groups: 1 day (range: 0–2 d) in the FOREseal group and 1 day (range: 0–3 d) in the control group (P=0.8357) | 611 | 380 |
| **75** | HIPPO[91] | 2017 | Annals of Surgery | To evaluate the effect of a self-gripping mesh (Progrip) on the incidence of chronic postoperative inguinal pain (CPIP) and recurrence rate after Lichtenstein hernioplasty | Pain as measured by 6-point verbal rating scale lasting at least 3 months postoperatively | The incidence of CPIP was 7.3% at 3 months declining to 4.6% at 24 months and did not differ between both groups | 395 | 339 |
| **76** | Langhans et al[92] | 2017 | Annals of Surgery | To compare the rate of positive resection margins between radioactive seed localization (RSL) and wire-guided localization (WGL) after breast conserving surgery (BCS) | Margin status after BCS | Resection margins were positive in 23 cases (11.8%) in the RSL group compared with 26 cases (13.3%) in the WGL group (P=0.65). The per protocol analysis revealed no difference in margin status (P=0.62) | 444 | 409 |
| **77** | PREVENT[93] | 2017 | Annals of Surgery | To investigate the incidence of parastomal hernias (PSHs) after end-colostomy formation using a polypropylene mesh in a randomized controlled trial versus conventional colostomy formation | Parastomal hernia formation | Three out of 67 patients (4.5%) in the mesh group and 16 out of 66 patients (24.2%) in the nonmesh group developed a PSH (P=0.0011) | NR | 150 |
| **78** | Sano et al[94] | 2017 | Annals of Surgery | To clarify the role of splenectomy in total | Overall survival (OS) | The 5-year survivals were 75.1% and 76.4% in the splenectomy and spleen preservation groups, | NA | 505 |

© 2021 Robinson NB et al. *JAMA Network Open*.

| | | | | | | | | |
|---|---|---|---|---|---|---|---|---|
| | | | | gastrectomy for proximal gastric cancer | | respectively. The hazard ratio was 0.88 (90.7%, confidence interval 0.67–1.16) (<1.21); thus, the noninferiority of spleen preservation was confirmed (P= 0.025). | | |
| **79** | COACT[95] | 2018 | Annals of Surgery | To evaluate the feasibility of laparoscopy-assisted distal gastrectomy (LADG) with D2 lymph node dissection compared with open distal gastrectomy (ODG) for the treatment of advanced gastric cancer | The noncompliance rate of the lymph node dissection | There were no significant differences in the overall noncompliance rate of lymph node dissection between LADG and ODG groups (47.0% and 43.2%, respectively; P=0.648) | 204 | 204 |
| **80** | HIGHLOW[96] | 2018 | Annals of Surgery | To compare the incidence of genitourinary (GU) dysfunction after elective laparoscopic low anterior rectal resection and total mesorectal excision (LAR þ TME) with high or low ligation (LL) of the inferior mesenteric artery (IMA) | Incidence of GU dysfunction when compared with preoperative baseline | GU function was impaired in both groups after surgery. LL group reported better continence and less obstructive urinary symptoms and improved quality of life at 9 months postoperative. Sexual function was better in the LL group compared to HL group at 9 months. Urinated volume, maximum urinary flow, and flow time were significantly (P < 0.05) in favor of the LL group at 1 and 9 months from surgery. The ultrasound measured post void residual volume and average urinary flow were significantly (P < 0.05) better in the LL group at 9 months postoperatively | 265 | 214 |
| **81** | Ielpo et al[97] | 2018 | Annals of Surgery | To compare the clinical and cost-effective outcomes of the open Lichtenstein repair (OL) and laparoscopic transabdominal preperitoneal (TAPP) repair for bilateral inguinal hernias | Operative and hospitalization costs | The overall cost of TAPP procedure was higher compared with the OL cost (1,683.93€ vs 1192.83€, P=0.027) | NR | 163 |
| **82** | Kim et al[98] | 2018 | Annals of Surgery | To compare the outcomes of robot-assisted surgery with those of laparoscopic surgery in the patients with rectal cancer | The quality of total mesorectal excision (TME) specimen | The TME quality did not differ between the robot-assisted and laparoscopic groups (80.3% vs 78.1% complete TME, respectively; 18.2% vs 21.9% nearly complete TME, respectively; P=0.599) | 671 | 163 |
| **83** | Lee et al[99] | 2018 | Annals of Surgery | To compare the incidence of incisional hernia (IH) between midline and transverse specimen extraction site in patients undergoing laparoscopic colectomy | IH incidence at 1 year | On intention-to-treat analysis, there was no difference in the incidence of IH at 1 year (transverse 2% vs midline 8%, P = 0.065) or after mean 30.3 month (standard deviation 9.4) follow-up (6% vs 14%, P=0.121) | 371 | 165 |
| **84** | LIGRO[100] | 2018 | Annals of Surgery | To evaluate if associating liver partition and portal vein ligation for staged hepatectomy (ALPPS) could increase resection rates (RRs) | Resection rate measured as the percentages of patients completing both stages of the treatment | The RR was 92% (95% confidence interval (CI) 84%–100%) (44/48) in the ALPPS arm compared with 57% (95% CI 43%–72%) (28/49) in the TSH arm (rate ratio 8.25 (95% CI 2.6–26.6); P < 0.0001) | 117 | 100 |

© 2021 Robinson NB et al. *JAMA Network Open*.

| | | | | | | | | |
|---|---|---|---|---|---|---|---|---|
| | | | | compared with two-stage hepatectomy (TSH) | | | | |
| **85** | Maggiori et al[101] | 2018 | Annals of Surgery | To compare outcome of single-port laparoscopy (SPL) and multiport laparoscopy (MPL) laparoscopy for colonic surgery | Length of theoretical hospital stay (LHS) | Procedure duration, intraoperative complication rate, postoperative 30-day morbidity, postoperative pain, and time to first bowel movement were similar between the groups, leading to similar theoretical LHS (SPL: 6±3 days; MPL: 6±2; P=0.298) | NR | 128 |
| **86** | OSLO-COMET[102] | 2018 | Annals of Surgery | To compare laparoscopic and open liver resection | Postoperative complications within 30 days (Accordion grade 2 or higher) | The postoperative complication rate was 19% in the laparoscopic surgery group and 31% in the open-surgery group (12 percentage points difference [95% confidence interval 1.67–21.8; P = 0.021]) | 294 | 280 |
| **87** | PADULAP[103] | 2018 | Annals of Surgery | To compare perioperative outcomes of pancreatoduodenectomy (PD) performed through the laparoscopic route or by open surgery | Length of hospital stay (LOS) | Laparoscopic versus open PD was associated with a significantly shorter LOS (median 13.5 vs. 17 d; P=0.024) and longer median operative time (486 vs. 365 min; P=0.0001) | 86 | 66 |
| **88** | PROPP[104] | 2018 | Annals of Surgery | To investigate the effect of pylorus resection on postoperative delayed gastric emptying (DGE) after partial pancreatoduodenectomy (PD) | DGE within 30 days according to the International Study Group of Pancreatic Surgery definition | In the PP group 24 of 95 patients (25.3%) and in the PR group 29 of 93 patients (31.2%) developed DGE (odds ratio 1.534, 95% confidence interval 0.788 to 2.987; P=0.208) | 1408 | 198 |
| **89** | Siribumrungwong et al[105] | 2018 | Annals of Surgery | To compare superficial surgical site infection (SSI) rates between delayed primary wound closure (DPC) and primary wound closure (PC) for complicated appendicitis | Superficial SSI defined by the Center for Disease Control criteria | The superficial SSI rate was lower in the PC than DPC groups (ie, 7.3% (95% confidence interval 4.4, 10.3) vs 10% (95% CI 6.6, 13.3)) with a risk difference (RD) of -2.7% (-7.1%, 1.9%), but this RD was not significant | NR | 607 |
| **90** | So et al[106] | 2018 | Annals of Surgery | To compare the clinical symptoms between Billroth II (B-II) and Roux-en-Y (R-Y) reconstruction after distal subtotal gastrectomy (DG) for gastric cancer | Gastrointestinal (GI) Symptoms Score 1 year after surgery | GI symptoms score did not differ between R-Y versus B-II reconstruction (mean difference -0.45, 95%CI -1.21 to 0.31, P=0.232) | 164 | 162 |
| **91** | TISSEEL[107] | 2018 | Annals of Surgery | To evaluate the effectiveness of the use of fibrin sealant (FS) for preventing the development of staple line complications (SLCs) after sleeve gastrectomy (SG) | Composite incidence of SLCs in each of the 2 groups | In an intention-to-treat analysis, the incidence of SLCs was similar in the FS and control groups (1.3% vs 2%, respectively; P=0.52) | 615 | 586 |
| **92** | Allaix et al[108] | 2019 | Annals of Surgery | To determine whether there are clinically relevant differences in outcomes between laparoscopic right colectomy (LRC) with | Length of hospital stay (LOS) | Median LOS was similar in the 2 groups [6 (IQR 5–7) vs 6 (IQR 5–8) days; P = 0.839] | 145 | 140 |

© 2021 Robinson NB et al. *JAMA Network Open*.

| | | | | | | | | |
|---|---|---|---|---|---|---|---|---|
| | | | | intracorporeal ileocolic anastomosis (IIA) and LRC with extracorporeal IA (EIA) | | | | |
| **93** | Chai et al[109] | 2019 | Annals of Surgery | To compare the surgical outcomes of lateral transperitoneal adrenalectomy (LTA) and posterior retroperitoneoscopic adrenalectomy (PRA) for benign adrenal tumor | Operative time | The mean operative times of LTA and PRA were comparable (59.7±18.6 vs 67.6±28.7 minutes, P = 0.139) | NR | 84 |
| **94** | Dumanian et al[110] | 2019 | Annals of Surgery | To compare targeted muscle reinnervation (TMR) to ''standard treatment'' of neuroma excision and burying into muscle for postamputation pain | Change between pre- and postoperative numerical rating scale (NRS, 0–10) pain scores for residual limb pain and PLP at 1 year | Changes in PLP scores at 1 year were 3.2 versus -0.2 (difference 3.4, adjusted confidence interval (aCI) -0.1 to 6.9, adjusted P=0.06) for TMR and standard treatment, respectively | NR | 28 |
| **95** | Dupont et al[111] | 2019 | Annals of Surgery | To determine if resection of cavity shave margins (CSM) can halve the rate of positive margins and re-excision in breast cancer patients undergoing partial mastectomy (PM) across multiple centers | Positive margin and re-excision rates | Those in the ''shave'' group were significantly less likely than those in the ''no shave'' group to have positive margins (19/196 (9.7%) vs 72/200 (36.0%), P < 0.001), and to require re-excision or mastectomy for margin clearance (17/196 (8.7%) vs. 47/200 (23.5%), P < 0.001) | 422 | 400 |
| **96** | Hirono et al[112] | 2019 | Annals of Surgery | To evaluate whether mattress suture of pancreatic parenchyma and the seromuscular layer of jejunum (modified Blumgart method) during pancreaticojejunostomy (PJ) decreases the incidence of clinically relevant postoperative pancreatic fistula (POPF) after pancreaticoduodenectomy (PD) | Incidence of grade B/C POPF based on the International Study Group on Pancreatic Fistula criteria | Grade B/C POPF occurred in 7 patients (6.8%) in the interrupted suture group and 11 (10.3%) in the mattress suture group (P=0.367) | 239 | 224 |
| **97** | LEOPARD[113] | 2019 | Annals of Surgery | To compare time to functional recovery after minimally invasive and open distal pancreatectomy (ODP) | Time to functional recovery | Time to functional recovery was 4 days [interquartile range (IQR) 3–6) in 51 patients after MIDP versus 6 days (IQR 5–8) in 57 patients after ODP (P < 0.001) | 181 | 111 |
| **98** | Lohmander et al[114] | 2019 | Annals of Surgery | To evaluate clinical outcomes of using acellular dermal matrix (ADM) with implant-based breast reconstructions (IBBRs) | Number of unplanned reoperations at 24 months, and safety expressed as the incidence of adverse events with a 6-month follow-up time for all participants | Four patients (6%) in each group had reconstructive failure with implant loss, but IBBR with ADM exhibited a trend of more overall complications and reoperations (difference 0.16, 95% CI, -0.01 to 0.32, P=0.070), and with higher risk of wound healing problems (P=0.013) | NR | 135 |

© 2021 Robinson NB et al. *JAMA Network Open*.

| | | | | | | | | |
|---|---|---|---|---|---|---|---|---|
| **99** | Parc et al[115] | 2019 | Annals of Surgery | To compare prospectively the complications and functional outcome of patients undergoing a J-Pouch (JP) or a side-to-end anastomosis (SE) for treatment of low rectal cancer at a 2-year time point after resection for rectal cancer | QOL and functional outcomesNo primary outcome explicitly stated | QOL scores using either instrument between the 2 groups at 12 and 24 months were similar (P > 0.05). Bowel movements, clustering, and FISI scores were similarNot reported | 259 | 238 |
| **100** | ROBOT[116] | 2019 | Annals of Surgery | To evaluate if Robot-assisted minimally invasive thoracolaparoscopic esophagectomy (RAMIE) reduces complications | Occurrence of overall surgery-related postoperative complications (modified Clavien-Dindo classification grade 2–5) | Overall, surgery-related postoperative complications occurred less frequently after RAMIE (59%) compared to OTE (80%) [risk ratio with RAMIE (RR) 0.74; 95% confidence interval (CI), 0.57–0.96; P=0.02] | 236 | 138 |
| **101** | Sabater et al[117] | 2019 | Annals of Surgery | To compare the rates of R0 resection in pancreatoduodenectomy (PD) for pancreatic and periampullary malignant tumors by means of standard (ST-PD) versus artery-first approach (AFA-PD) | Rate of tumor-free resection margins (R0) | R0 resection rates were 77.3% (95% CI: 68.4–87.4) with ST-PD and 67.9% (95% CI: 58.3–79.1) with AFA-PD, P=0.194 | 179 | 176 |
| **102** | SAKK 40/04[118] | 2019 | Annals of Surgery | To compare, in a phase 3, prospective, randomized, multi-center clinical trial functional outcome of reconstruction procedures following total mesorectal excision (TME) | Comparison of composite evacuation scores 12 months after TME | Composite evacuation scores of PP and ITT populations did not show statistically significant differences among the 3 groups at any time point | 336 | 336 |
| **103** | STOMAMESH[119] | 2019 | Annals of Surgery | To determine whether parastomal hernia (PSH) rate can be reduced by using synthetic mesh in the sublay position when constructing permanent end colostomy | Parastomal hernia at 1 year | No difference in rate of PSH was revealed in the analyses of clinical (P=0.866) and radiologic (P=0.748) data | 240 | 232 |
| **104** | TONIG[120] | 2019 | Annals of Surgery | To determine the incidence of postoperative hypoparathyroidism after near-total thyroidectomy (NTT) versus TT in GD | The incidence of transient postoperative hypoparathyroidism | Transient hypoparathyroidism occurred in 19% (20/103) patients after NTT and in 21% (21 of 102) patients after TT (P=0.84), which persisted >6 months in 2% and 5% of the NTT and TT groups (P=0.34) | 220 | 207 |
| **105** | Cerfolio et al[121] | 2008 | Annals of Thoracic Surgery | To evaluate if leaving the intercostal muscle flap intact reduces postoperative pain | Numeric pain score at postoperative weeks 3, 4, 8, and 12, survival, Readmit ≤30 days post-op, Prescription pain med use, Receiving adjuvant | Intrahospital pain scores were similar; however, at postoperative weeks 3, 4, 8, and 12, the D group had significantly lower mean numeric pain scores and was using fewer analgesics (p < 0.05 for all). At 12 weeks, patients in the D group were more | 263 | 160 |

© 2021 Robinson NB et al. *JAMA Network Open*.

| | | | | | | | | |
|---|---|---|---|---|---|---|---|---|
| | | | | | treatment, Return to baseline activities, Satisfied with procedure/care | likely to have returned to baseline activity (p=0.002). | | |
| **106** | Li et al[122] | 2008 | Annals of Thoracic Surgery | To measure the impact of limited denervation on compensatory sweating while performing endoscopic thoracic sympthectomy | Symptom resolution, postoperative complication, levels of satisfaction, and severity of compensatory sweating | The postoperative complications were minor, and Horner's syndrome was not detected in either group. The frequency of mild and moderate compensatory sweating was not significantly different between the two groups, but the incidence of severe compensatory sweating was significantly lower after T3 sympathectomy (3% versus 10%). As for satisfaction rate, group T3 was superior to group T2–4 (96.6% versus 89.6%). The rate of symptom resolution was 100%, and no recurrence was found in either group | NR | 232 |
| **107** | Schimmer et al[123] | 2008 | Annals of Thoracic Surgery | To compare two surgical techniques with respect to the occurrence of SWI in patients with an increased risk | The rate of sternal dehiscence as well as the occurrence of superficial sternal wound infections and deep sternal wound infections | The rate of sternal dehiscence, superficial sternal wound infections, and deep sternal wound infections (conventional technique 2.5%, 3.4%, 2.5%; and Robicsek 3.7%, 5.6%, 3.7%) did not differ between the groups | NR | 815 |
| **108** | Krishnamoorthy et al[124] | 2009 | Annals of Thoracic Surgery | To compare results of skin closure using Dermabond and subcuticular sutures after coronary artery bypass grafting (CABG) | Cosmetic outcomes at weeks 1 and 6 | Patients in the Dermabond group also reported superior cosmetic outcome at weeks 1 ($p < 0.001$) and 6 (p=0.001) and improved patient satisfaction ($p < 0.001$) | NR | 106 |
| **109** | Suri et al[125] | 2009 | Annals of Thoracic Surgery | To investigate the hemodynamic performance of two widely used, stented xenograft biologic prostheses and to determine the effects of valve hemodynamics on regression of LV hypertrophy 1 year after surgery | Transprosthetic gradient, aortic valve orifice area, and LV mass | The mean aortic valve gradient at dismissal was 19.4 mm Hg (MM) versus 13.5 mm Hg (EP; $p < 0.0001$), and at 1 year was 20.4 mm Hg versus 13.4 mm Hg ($p < 0.0001$). During the first year after implantation, both groups demonstrated similar regression of LV mass index (MM, 32.4 $g/m^2$ versus EP, 27.0 $g/m^2$; p=0.40) | NR | 152 |
| **110** | Allama[126] | 2010 | Annals of Thoracic Surgery | To evaluate the effect of nondivided intercostal muscle flap and intracostal sutures (protecting the intercostal nerves from trauma) on early postoperative pain were investigated, comparing this technique with traditional pericostal sutures | Pain, which was measured by a blinded trained physician using the numeric rating scale from 0 to 10 (0 = no pain, 10 = extreme pain) postoperatively daily until the seventh day, at 1, 3, and 6 months | Postoperative pain score throughout the first week was significantly lower in the patients in the intercostal muscle flap group, who had also a significantly earlier postoperative ambulation and return to normal daily activities, and received significantly lower doses of postoperative analgesics. After 1 month, patients in the intercostal muscle flap group had a significantly lower pain score and use of analgesics. After 3 months, pain score was not significantly different between both groups, but the use of analgesics was significantly lower in the intercostal muscle flap group. After 6 months, no significant | 168 | 120 |

© 2021 Robinson NB et al. *JAMA Network Open*.

| | | | | | | | | |
|---|---|---|---|---|---|---|---|---|
| | | | | | | difference was present between both groups with regard to pain score or the use of analgesics | | |
| **111** | Yousefnia et al[127] | 2010 | Annals of Thoracic Surgery | To investigate the feasibility of performing papillary muscle repositioning (PMR) for subvalvular-sparing mitral valve replacement procedures in patients with ischemic mitral regurgitation and to determine the early and late effects of this procedure on the clinical outcome and left ventricular mechanics | No primary outcome explicitly stated | Not reported | NR | 50 |
| **112** | Aykut et al[128] | 2011 | Annals of Thoracic Surgery | To compare the incidence of sternal dehiscence after prophylactic sternal weave and figure-of-eight suturing in diabetic obese patients undergoing coronary artery bypass grafting (CABG) | Sternal dehiscence according to clinical examination and chest radiography | There were 8 cases of sternal dehiscence documented: 7 in group A (figure of eight group) and 1 in group B (sternal weave group). Sternal dehiscence was significantly lower in group B ($p < 0.05$) | NR | 150 |
| **113** | Baumgartner et al[129] | 2011 | Annals of Thoracic Surgery | To compare sympathicotomy over the second (R2) vs third (R3) costal head relative to these variables in patients with massive palmar hyperhidrosis | Level of recurrence, dramatic failures, Compensatory Hyperhidrosis severity scale, subjective change in plantar sweating at 6 months and 1 year | Sympathicotomy at R2 failed to cure palmar hyperhidrosis in 5 of 122 (4.1%) extremities, but only 2 (1.6%) were to a truly profound dripping level of recurrence. Sympathicotomy at R3 failed to cure palmar hyperhidrosis in 5 of 120 extremities (4.2%), and all were dramatic failures with dripping recurrent sweating | NR | 121 |
| **114** | Lai et al[130] | 2011 | Annals of Thoracic Surgery | To evaluate the preventive effect of thoracic duct mass ligation on postoperative chylothorax | Occurrence rates of chylothorax | Chylothorax occurred in 8 patients, giving an incidence of 1.2%. In the preservation group, chylothorax occurred in 7 patients (2.1%), and in the prevention group, 1 case of chylothorax was found (0.3%). The incidence of postoperative chylothorax was significantly lower in the prevention group | 746 | 653 |
| **115** | Mannacio et al[131] | 2011 | Annals of Thoracic Surgery | To evaluate the flow outcome of the skeletonized versus pedicled left internal mammary artery | LIMA flow before papaverine application | Skeletonized left internal mammary arteries demonstrated better flow capacity at rest and during adenosine recruitment perioperatively and at all time points of follow-up | NR | 200 |
| **116** | Aye et al[132] | 2012 | Annals of Thoracic Surgery | To compare the effectiveness of Laparoscopic Hill repair LHR against the gold standard laparoscopic Nissen fundoplication | Clinical recurrence as evidenced by reoperation for failure or resumption of antisecretory medication | Two LNF and two LHR required reoperation for failed repair | NR | 111 |
| **117** | Licht et al[133] | 2012 | Annals of Thoracic Surgery | To compare rib-oriented (R2 vs R2–R3) sympathicotomy for isolated facial blushing | QOL, local effect on facial blushing, and side effects | QOL increased significantly in all social and mental domains in both groups. Overall, 85% of the patients had an excellent or satisfactory result, | NR | 100 |

© 2021 Robinson NB et al. *JAMA Network Open*.

| | | | | | | | | |
|---|---|---|---|---|---|---|---|---|
| | | | | | | with no significant difference between the R2 procedure and the R2–R3 procedure. Mild recurrence of facial blushing occurred in 30% of patients within the first year. One patient experienced Horner's syndrome. Compensatory sweating occurred in 93% of patients, gustatory sweating 36%, and dry hands in 66%; 13% of patients regretted the operation despite thorough preoperative selection and information | | |
| **118** | Raman et al[134] | 2012 | Annals of Thoracic Surgery | To evaluate if sternal reconstruction after median sternotomy using rigid fixation with plates may improve bone healing and reduce pain when compared with wire cerclage | Sternal union, pain, and function | Sternal healing was superior in rigid plate fixation patients at both 3 and 6 months. Pain scores and narcotic usage were lower in rigid plate fixation patients. Significant differences in pain scores were observed at 3 weeks for total pain (p=0.020) and pain with coughing (p=0.0084) or sneezing (p=0.030) | NR | 140 |
| **119** | Birla et al[135] | 2013 | Annals of Thoracic Surgery | To compare the clinical performance of 2 stented porcine aortic bioprostheses: the Carpentier-Edwards supraannular aortic valve (CE-SAV) from Edwards Lifesciences and the Mosaic valve from Medtronic Corp | Freedom from SVD echocardiography at 1, 5, and 10 years after implantation | There were no statistically significant differences between the 2 groups in terms of structural valve deterioration (SVD) (p=0.16), paraprosthetic leak (p=0.13), thromboembolism (p=0.25), endocarditis (p=0.68), and freedom from reoperation at 5 years (p=0.27) | NR | 403 |
| **120** | Dreifaldt et al[136] | 2013 | Annals of Thoracic Surgery | To compare the patency of no-touch saphenous vein with that of radial artery grafts | Patency of grafts at follow-up | The patency of grafts for no-touch saphenous vein and radial artery was 94% versus 82% (p=0.01), respectively. The patency of coronary arteries grafted with no-touch saphenous vein and radial artery grafts was 95% versus 84% (p=0.005), respectively | 1678 | 108 |
| **121** | Bolotin et al[137] | 2014 | Annals of Thoracic Surgery | To evaluate the safety and efficacy of a novel aortic cannula producing simultaneous forward flow and backward suction for extracting solid and gaseous emboli from the ascending aorta and aortic arch upon their intraoperative release | The volume of new brain lesions measured by diffusion-weighted magnetic resonance imaging (DW-MRI), performed preoperatively and postoperatively | The volume of new brain lesion for the treatment group was (mean ± standard error of the mean) 44.00 ± 64.00 versus 126.56 ± 28.74 $mm^3$ in the control group (p=0.004). Of the treatment group, 41% demonstrated new postoperative lesions versus 66% in the control group (p=0.03) | NR | 66 |
| **122** | Lee et al[138] | 2014 | Annals of Thoracic Surgery | To clarify whether an additional coverage procedure on the staple line after thoracoscopic bullectomy prevents postoperative recurrence compared with additional pleurodesis | Rates of ipsilateral 1-year recurrence rate and recurrence requiring intervention (RRI), defined as a postoperative recurrent pneumothorax large | After a median follow-up of 19.5 months, the postoperative 1-year recurrence rate was 9.5% in the coverage group and 10.7% in the pleurodesis group. The 1-year recurrence rate requiring intervention was 5.8% in the coverage group and 7.8% in the pleurodesis group | NR | 1414 |

© 2021 Robinson NB et al. *JAMA Network Open*.

| | | | | | | | | |
|---|---|---|---|---|---|---|---|---|
| | | | | | enough to need closed thoracostomy or reoperation | | | |
| **123** | Min et al[139] | 2014 | Annals of Thoracic Surgery | To investigate the effectiveness of mechanical pleurodesis after thoracoscopic treatment of primary spontaneous pneumothorax | The recurrence rate of the thoracoscopic wedge resection and mechanical pleurodesis (WR+MP) group | Postoperative recurrence rate did not significantly differ between groups (log-rank test p=0.791; Breslow test p=0.722) | NR | 305 |
| **124** | CADENCE-MIS[140] | 2015 | Annals of Thoracic Surgery | To compare the outcomes for MIS-RDAVR with those of conventional FS-AVR | Cross-clamp time and CPB time | MIS-RDAVR was associated with significantly reduced aortic crossclamp times compared with FS-AVR (41.3 ± 20.3 vs 54.0 ± 20.3 minutes, p < 0.001), although cardiopulmonary bypass times were similar (68.8 ± 29.0 vs 74.4 ± 28.4 minutes, p=0.21) | NR | 100 |
| **125** | VEST[141] | 2015 | Annals of Thoracic Surgery | To investigate whether external stenting inhibits SVG diffuse intimal hyperplasia 1 year after coronary artery bypass graft surgery | SVG intimal hyperplasia (mean area) assessed by intravascular ultrasonography at 1 year | Overall SVG failure rates did not differ significantly between the two groups (30% stented versus 28.2% nonstented SVG, p=0.55). The SVG mean intimal hyperplasia area, assessed in 43 SVGs, was significantly reduced in the stented group (4.37 ± 1.40 $mm^2$) versus nonstented group (5.12 ± 1.35 $mm^2$, p=0.04) | NR | 30 |
| **126** | Baumbach et al[142] | 2016 | Annals of Thoracic Surgery | To investigate minimally invasive extracorporeal circulation (MECC) in coronary operation | Procedural and postoperative outcomes, including the levels of inflammatory factors (procalcitonin, interleukin (IL)-6, IL-8, and IL-10), tumor necrosis factor-α (TNF-α), and interferon-gamma (IFN-γ)) | Hospital mortality (n=1 versus n= 3; p=0.339) and other complications were similar. Hemoglobin level (111.9 ± 19.0 g/L versus 103.8 ± 14.6 g/L; p=0.001), the number of packed red blood cells (PRBCs) (1.1 ± 1.9 versus 1.7 ± 1.8; p=0.003), the levels of ILs (IL-6, 194.0 ± 131.8 pg/mL versus 289.2 ± 62.5 pg/mL; p=0.020; IL-8, 38.1 ± 27.3 pg/ mL versus 45.8 ± 43.4 pg/mL; p=0.012; IL-10, 29.0 ± 123.9 pg/mL versus 49.9 ± 85.6 pg/mL; p=0.012), TNF-a (3.8 ± 6.7 ng/mL versus 10.8 ± 47.7 ng/mL; p=0.049), and IFN-g (1.9 ± 1.9 pg/mL versus 4.5 ± 2.7 pg/mL; p=0.027) were in favor of patients in the MECC group | NR | 200 |
| **127** | Nguyen et al[143] | 2016 | Annals of Thoracic Surgery | To compare induction of reactive oxygen species (ROS) and activation of nuclear factor (NF)-kB, p38 mitogen-activated protein kinase (MAPK) within leukocytes, and leukocyte accumulation in cantharidin-induced blisters in patients exposed to miniaturized CPB (mCPB) and those who underwent conventional CPB | ROS induction levels in granulocytes | ROS in lymphocytes were elevated in cCPB compared with mCPB (p < 0.01), whereas ROS in granulocytes and monocytes were similar between groups | 50 | 26 |

© 2021 Robinson NB et al. *JAMA Network Open*.

| | | | | | | | | |
|---|---|---|---|---|---|---|---|---|
| | | | | (cCPB) | | | | |
| **128** | Fiorelli et al[144] | 2017 | Annals of Thoracic Surgery | To evaluate whether cardiac autonomic changes could be associated with different extent of sympathetic nerve resection in the management of essential palmar hyperhidrosis | To evaluate whether the changes in HRV could be associated with the extent of sympathetic resection. In each group, HRV was evaluated 7 day before ETS and 24 hours and 1, 3, and 6 months later, using a 24-hour Holter recording | In both groups, we observed a significant increase ($p < 0.05$) of vagal activity measurements as root mean square of the successive differences of heart period; proportion of adjacent normal R-R intervals >50 ms; high frequency; and a significant decrease ($p < 0.05$) of adrenergic activity variables as heart rate, low frequency, and the ratio between low frequency and high frequency during daytime, nighttime, and 24-hour periods. These changes were significantly more evident ($p < 0.05$) in the sympathectomy group than in the sympathicotomy group | NR | 60 |
| **129** | Okami et al[145] | 2017 | Annals of Thoracic Surgery | To evaluate flat face with equal height staples and the stepped face with graduated height staples stapling ability in lobectomy | Scores of the staple formation at the lobar bronchial stump | The case scores were significantly lower in the equal height staples than in the graduated height staples (2.17 versus 2.88, p=0.0003) respectively | 69 | 61 |
| **130** | Pettersen et al[146] | 2017 | Annals of Thoracic Surgery | To evaluate the impact of pedicled veins on duration of operations, leg wound infections, and postoperative bleeding | Vein graft patency 6 months and 5 years | No significant difference was found in intraoperative vein graft flow, postoperative bleeding, or leg wound infections (4% in each group) | 156 | 100 |
| **131** | Etiwy et al[147] | 2018 | Annals of Thoracic Surgery | To assess the time and cost required for suture fixation with the automated device versus conventional hand tying in sternotomy for mitral or tricuspid ring annuloplasty | The time required to affix the annuloplasty device to the valve annulus | The times taken to affix a mitral annuloplasty band or ring were 6.1 ± 0.9 min for manual tying versus 3.1 ± 0.4 min for automated fasteners ($p < 0.0001$); when calculated per annuloplasty stitch, the values were 22 ± 2 s versus 12 ± 1.1 s, respectively ($p < 0.0001$) | 50 | 50 |
| **132** | Long et al[148] | 2018 | Annals of Thoracic Surgery | To confirm that VATS is not inferior or even superior to open operation for early stage NSCLC in terms of short-term and oncologic efficacy | 5-year overall and disease-free survival | Not reported (interim analysis) | 508 | 481 |
| **133** | Salim et al[149] | 2018 | Annals of Thoracic Surgery | To compare T2-4 versus T3-4 sympathectomy for the treatment of hyperhidrosis | Improvement of palm sweating, development of complications, patient satisfaction, and quality of life | Overdry hands were significant in group A (p=0.032). Compensatory sweating was significant in group A after the first month (p=0.016), after 6 months (p=0.022), and after 12 months (p=0.025). In group B, very satisfied patients were significant after 6 and 12 months (p=0.002 and 0.000, respectively) | NR | 120 |
| **134** | Halfwerk et al[150] | 2019 | Annals of Thoracic Surgery | To compare MiECC to an advanced standard system with respect to blood loss | Postoperative blood loss after 12 hours and at drain removal | MiECC patients (n=63) had a significant lower blood loss (230 mL, 95% confidence interval: 203 to 261 mL) than regular patients (n=62) after 12 hours (288 mL, 95% confidence interval: 241 to 344 mL, p=0.04) | NR | 180 |

© 2021 Robinson NB et al. *JAMA Network Open*.

| | | | | | | | | |
|---|---|---|---|---|---|---|---|---|
| **135** | Jeong et al[151] | 2019 | Annals of Thoracic Surgery | To compare the early outcomes of a concomitant maze procedure using N2O-based cryoablation (the N2O group) versus argon gas-based cryoablation (the argon group) in patients with persistent atrial fibrillation (AF) | Normal sinus rhythm at 1 year | Normal sinus rhythm was maintained in 26 patients in the N2O group and 26 patients in the argon group (86.7% versus 86.7%, p=1.000) | 66 | 60 |
| **136** | Leshnower et al[152] | 2019 | Annals of Thoracic Surgery | To compare two different established neuroprotective strategies in patients undergoing elective transverse hemiarch replacement | Composite of stroke, transient ischemic attack, and magnetic resonance imaging–adjudicated injury | The primary end point was achieved in 100% of MHCA+ACP patients compared with 45% of DHCA+RCP patients (p < 0.01). | NR | 20 |
| **137** | Ferretti et al[153] | 2008 | Arthroscopy | To test a new method of harvesting semitendinosus tendon during anterior cruciate ligament reconstruction that would allow an anatomic reinsertion of the regenerated tendon and minimize postoperative internal rotation strength loss | Postoperative recovery of flexion and internal rotation strength as measured by clinical examination, isokinetic tests, and magnetic resonance imaging | Isokinetic tests showed a significant deficit in internal rotation strength at 60°/s in patients in group B (control group) (84.60% v 97.37% in patients in group A). No deficits were found in group A (intervention group). Magnetic resonance imaging evaluations showed a higher percentage of regenerated semitendinosus in group A patients (100%) than that in group B patients (50%) at the joint line level | NR | 35 |
| **138** | Järvelä et al[154] | 2008 | Arthroscopy | To compare tunnel enlargement in patients with double-bundle and single-bundle anterior cruciate ligament (ACL) reconstruction | Tunnel enlargement on oblique coronal / sagittal plane | No significant differences were found between the double-bundle group and the single-bundle group in tunnel enlargement on the femoral side. However, on the tibial side, tunnel enlargement was greater in the single-bundle group than in the double-bundle group in each tunnel (P=0.051) | NR | 60 |
| **139** | Monteiro et al[155] | 2008 | Arthroscopy | To compare the functional results of arthroscopic treatment for traumatic anterior shoulder instability in 2 groups of athletes | Clinical outcome based on Rowe score and the Athletic Shoulder Outcome Scoring System | The mean Rowe score was 83.8 in group A and 79.5 in group B. The mean values for the Athletic Shoulder Outcome Scoring System were 84 and 79.2, respectively. Good or excellent results were found in 90.5% of patients in group A and 87.5% in group B. We had 2 failures (9.5%) in group A and 3 (12.5%) in group B. No statistically significant difference was found in the comparison of the outcomes (P=.05). | NA | 50 |
| **140** | Myers et al[156] | 2008 | Arthroscopy | To prospectively assess the outcome of hamstring autograft anterior cruciate ligament (ACL) reconstruction by use of identically shaped bioabsorbable and titanium interference screws | Clinical outcome based on Lysholm and International Knee Documentation Committee score | There were no differences in clinical outcome by use of Lysholm and International Knee Documentation Committee scores between the 2 groups at any stage of follow-up to 2 years | 114 | 100 |
| **141** | Siebold et al[157] | 2008 | Arthroscopy | To evaluate the clinical results of four-tunnel DB ACL reconstruction | Objective / subjective clinical outcomes including International | The subjective results were similar in groups. The subjective International Knee Documentation Committee (IKDC) 2000 score was 88 P for DB | 165 | 70 |

© 2021 Robinson NB et al. *JAMA Network Open*.

| | | | | | | | | |
|---|---|---|---|---|---|---|---|---|
| | | | | | Knee Documentation Committee (IKDC) score, Cincinnati knee score, Lysholm score, pivot shift test | versus 90 P for SB; the Lysholm score was 90 P for DB versus 93 P for SB; and the Cincinnati knee score was 91 P for DB versus 92 P for SB.The objective IKDC was significantly higher for DB: 78% "A" (P< .000) and 19% "B" compared to 24% "A" and 68% "B" for S. The average KT-1000 side-to-side difference was 1.0 mm for DB and 1.6 mm for SB (P=.054) and the pivot shift test was negative in 97% for DB (P=0.01) and 71% for SB | | |
| **142** | Grasso et al[158] | 2009 | Arthroscopy | To compare the clinical outcome of arthroscopic rotator cuff repair with single-row and double-row techniques | Clinical outcome based on Disabilities of the Arm, Shoulder and Hand (DASH) and Work-DASH self-administered questionnaires, normalized Constant score, and muscle strength measurement | The mean DASH scores were 15.4±15.6 points in group 1 and 12.7±10.1 points in group 2; the mean Work-DASH scores were 16.0±22.0 points and 9.6±13.3 points, respectively; and the mean Constant scores were 100.5±17.8 points and 104.9±21.8 points, respectively. Muscle strength was 12.7±5.7 lb in group 1 and 12.9±7.0 lb in group 2 | NR | 80 |
| **143** | Papp et al[159] | 2009 | Arthroscopy | To compare radiologic changes in the sagittal plane after closing-wedge and combined high tibial osteotomies (HTOs) performed in patients with medial knee arthrosis associated with a varus deformity and requiring a 10° correction | Radiologic changes in the sagittal plane angle | We found that at the end of the study, 12 months after the operation, CO resulted in significantly smaller changes in TS angle (4.7% v 38.2%) and ISI (2.2% v 5.7%) values than CWO | 139 | 100 |
| **144** | Chen et al[160] | 2010 | Arthroscopy | To assess effects of the extent of capsular release and to define the benefit of additional release of the inferior glenohumeral ligament (IGHL) from inferior to posterior in frozen shoulder | Constant functional scores and range of motion following capsular release | There was a significant and rapid reduction in the visual analog scale score in both groups postoperatively. No statistical difference in the visual analog scale score was found between the 2 groups at the corresponding time points. Overall, patients had restored shoulder ROM at the last follow-up without difference between group 1 and group 2 | NR | 74 |
| **145** | Milano et al[161] | 2010 | Arthroscopy | To compare the clinical outcome of arthroscopic rotator cuff repair with metal and biodegradable suture anchors | Clinical outcome based on Disabilities of the Arm, Shoulder and Hand (DASH) score | The mean DASH scores were 17.6±17.2 points in group 1 and 22.8±19.9 points in group 2; the mean Work-DASH scores were 24.9±28.1 points and 22.5±24.1 points, respectively; and the mean Constant scores were 104±20.5 points and 98.6±14.3 points, respectively. Differences between groups 1 and 2 were not significant | NR | 110 |
| **146** | Koh et al[162] | 2011 | Arthroscopy | To compare the clinical outcomes and the retear rates of arthroscopic single-row (SR) and double-row (DR) suture | Clinical outcome based on demographic data, Pain visual analog scale (PVAS) scores, American | No intergroup differences in pain visual analog scale, American Shoulder and Elbow Surgeons, Constant, or University of California, Los Angeles scores were found at final follow-up. Only mean | 184 | 71 |

© 2021 Robinson NB et al. *JAMA Network Open*.

| | | | | | | | | |
|---|---|---|---|---|---|---|---|---|
| | | | | anchor repair in 2- to 4-cm rotator cuff tears | Shoulder and Elbow Surgeons (ASES) scores, mean surgical times, and patient satisfaction | surgical time was significantly different between the 2 groups | | |
| **147** | Sabat et al[163] | 2011 | Arthroscopy | To compare the incidence and properties of tunnel widening in patients undergoing anterior cruciate ligament reconstruction with quadrupled hamstring graft by use of either EndoButton CL or Transfix on the femoral side with a bioabsorbable interference screw in the tibial tunnel | Femoral tunnel widening assessed by computed tomography scan | Femoral tunnel widening at the aperture and at midway was significantly greater in the EndoButton group compared with the Transfix group | 53 | 34 |
| **148** | Wipfler et al[164] | 2011 | Arthroscopy | To analyze the long-term evaluation of clinical, functional, and magnetic resonance imaging (MRI) results after implant-free press-fit anterior cruciate ligament (ACL) reconstruction with bone–patella tendon (BPT) versus quadrupled hamstring tendon (HT) grafts | Clinical outcomes based on KT-1000 test and single-leg hop test | The score on the International Knee Documentation Committee evaluation form was significantly better in the HT group. The clinical examination including range of motion, KT-1000 test, and pivot-shift test showed no significant differences. Kneeling (1.5/1.1, P=.002), knee walking (1.72/1.14, P=.002), and single-leg hop test (95.8%/99.1%, P=.057) were better in the HT group | NA | 62 |
| **149** | Kim et al[165] | 2012 | Arthroscopy | To compare femoral graft bending angles and femoral tunnel geometries between the transportal (TP) and outside-in (OI) techniques after anatomic double-bundle (DB) anterior cruciate ligament (ACL) reconstruction | Femoral graft bending angles and femoral tunnel geometries as assessed by CT scan | The mean anteromedial (AM) and posterolateral (PL) femoral graft bending angles of group II (97.3°±8.3°and 97.4°±8.6°, respectively) were significantly more acute than those of group I (108.2°±8.4° and 109.9°±8.8°, respectively) (P=.001). The mean AM femoral tunnel length of group II (34.3±3.9 mm) was significantly longer than that of group I (31.9±2.7 mm) (P=.02). However, the mean PL femoral tunnel lengths did not differ between groups | 62 | 39 |
| **150** | Lawhorn et al[166] | 2012 | Arthroscopy | To compare the results and outcome of anterior cruciate ligament (ACL) reconstruction using autogenous hamstring tendon versus fresh-frozen allograft anterior tibialis tendon | Knee laxity using KT-1000 measurement, IKDC score, Lysholm score, and Tegner score | There was no difference in stability between the 2 groups (P=.05). The mean IKDC subjective score was 91.0 for the autograft group and 90.9 for the allograft group (P=.05). The functional IKDC scores for the autograft group were normal in 46 patients (85%), nearly normal in 7 patients (13%), and severely abnormal in 1 patient. For the allograft group, the functional IKDC scores were normal in 43 patients (90%) and nearly normal in 5 (10%) (P=.05) | NR | 147 |

© 2021 Robinson NB et al. *JAMA Network Open*.

| | | | | | | | | |
|---|---|---|---|---|---|---|---|---|
| **151** | Lee et al[167] | 2012 | Arthroscopy | To determine the relation between the tunnel positions and the kinematic improvement of the knee joint after single-bundle (SB) and double-bundle (DB) anterior cruciate ligament (ACL) reconstructions | Kinematic changes and tunnel position | The average tunnel location was mostly different between the SB and posterolateral bundle (PLB) tunnels, as well as between the anteromedial bundle (AMB) and PLB tunnels of DB ACL reconstructions, whereas the SB and AMB tunnels were similar in the tibial mediolateral and femoral deep-shallow positions | 82 | 42 |
| **152** | Ma et al[168] | 2012 | Arthroscopy | To compare the clinical and imaging outcomes of single-row and double-row suture anchor fixation in arthroscopic rotator cuff repair with emphasis on analysis of the effect of various tear size on repair integrity | Clinical / imaging outcomes based on UCLA score and the ASES index and assessing muscle strength in abduction and external rotation | The UCLA score; the ASES index; and muscle strength were significantly increased in both groups after surgery, but there was no significant difference between the 2 groups | NR | 64 |
| **153** | Shin et al[169] | 2012 | Arthroscopy | To compare the clinical outcomes of patients who underwent partial-thickness articular-sided rotator cuff repairs by 2 surgical techniques | Clinical outcome based on visual analog scales, American Shoulder and Elbow Surgeons and Constant shoulder scores | Shoulder function and range of shoulder motion recovered faster in group II during the recovery period (American Shoulder and Elbow Surgeons score, 54.9±3.7 in group I v 64.6±3.2 in group II; P=.037) (Constant score, 57.9±2.9 in group I v 70.8±3.3 in group II; P=.019). Furthermore, patients in group I had significantly more pain (5.9±0.4) than patients in group II (2.8±0.5) (P=.001) until 3 months after surgery | 92 | 48 |
| **154** | Shin et al[170] | 2012 | Arthroscopy | To assess the role of acromioplasty in the arthroscopic repair of small- to medium-sized rotator cuff tears | Clinical outcome based on pain and satisfaction visual analog scores and range of motion | There were no significant differences with respect to pain and range of motion between the groups at the final follow-up (1.1±0.9 v 1.3±1.4 on visual analog scale). Functional outcomes also showed no significant differences between the 2 groups (American Shoulder and Elbow Surgeons score, 90.7±13.1 v 87.5±12.0; Constant score, 85.0±11.3 v 83.3±13.0; and University of California, Los Angeles score, 33.4±3.3 v 32.3±3.5) | 150 | 120 |
| **155** | Bourke et al[171] | 2013 | Arthroscopy | To compare the outcome of 2 bioabsorbable screws for tibial interference fixation in anterior cruciate ligament reconstruction with reference to rate of absorption, osteoconductive properties, and clinical outcomes | Measurement of bony ingrowth into the tibial tunnel and area of the screw postoperatively by use of radiologic software | At 24 months, 88% of Calaxo screws showed complete screw resorption compared with 0% of Milagro screws (P < .001). At 24 months, the mean volume of new bone formation for the Calaxo group was 21% of original screw volume. Ossification of the Milagro screw was unable to be accurately assessed as a result of incomplete screw resorption | 374 | 60 |
| **156** | Gudas et al[172] | 2013 | Arthroscopy | To compare the concomitant treatment of articular cartilage damage in the medial femoral condyle with osteochondral autologous transplantation | Clinical outcome based on International Knee Documentation Committee (IKDC) score, | The OAT-ACL group's IKDC subjective knee evaluation was significantly better than that of the MF-ACL group (P=.024) and D-ACL group (P=.018). However, the IKDC subjective score of the IAC-ACL group was significantly better than the OAT-ACL | NR | 102 |

© 2021 Robinson NB et al. *JAMA Network Open*.

| | | | | | | | | |
|---|---|---|---|---|---|---|---|---|
| | | | | (OAT), microfracture, or debridement procedures at the time of anterior cruciate ligament (ACL) reconstruction | Tegner activity score, and clinical assessment | group's IKDC evaluation (P=.043). There was no significant difference between the MF-ACL and D-ACL groups' IKDC subjective scores (P= .058) | | |
| **157** | Hwang et al[173] | 2013 | Arthroscopy | To investigate whether the press-fit technique reduces tunnel volume enlargement (TVE) and improves the clinical outcome after anterior cruciate ligament reconstruction at a minimum follow-up of 1 year compared with conventional technique | TVE and tunnel widening using computed tomography scanning, functional outcome using International Knee Documentation Committee and Lysholm scores, for rotational stability using the pivot-shift test, and for anterior laxity using the KT-2000 arthrometer | There were no significant differences in TVE between the 2 groups. In group A, in which the press-fit technique was used, mean volume enlargement in the femoral tunnel was 65% compared with 71.5% in group B (P=.84). Both groups showed no significant difference for functional outcome (mean Lysholm score P = .73, International Knee Documentation Committee score P = .15), or knee laxity (anterior P = .78, rotational P = .22) at a minimum follow-up of 1 year | 85 | 72 |
| **158** | Krych et al[174] | 2013 | Arthroscopy | To compare the outcomes of arthroscopic labral repair and selective labral debridement in female patients undergoing arthroscopy for the treatment of pincer-type or combined pincer- and cam-type femoroacetabular impingement | Validated Hip Outcome Score (HOS) to determine hip function, and simple subjective outcome measure | The postoperative ADL HOS was significantly better in the repair group (91.2; range, 73 to 100) compared with the debridement group (80.9; range, 42.6 to 100; P < .05). Similarly, the postoperative sports HOS was significantly greater in the repair group (88.7; range, 28.6 to 100) than in the debridement group (76.3; range, 28.6 to 100; P < .05). Additionally, patient subjective outcome was significantly better in the labral repair group (P=.046). | 41 | 36 |
| **159** | Lubowitz et al[175] | 2013 | Arthroscopy | To compare the clinical effectiveness of full-tunnel anterior cruciate ligament (ACL) reconstructive surgery with all-inside ACL reconstruction | Clinical effectiveness based on International Knee Documentation Committee (IKDC) Knee Examination Form, IKDC Subjective Knee Evaluation Form, Knee Society Score (KSS), Short Form 12 (SF-12) score, femoral or tibial tunnel or socket widening, narcotic consumption, and visual analog scale (VAS) pain score | There were no differences between groups with regard to IKDC Knee Examination Form, IKDC Subjective Knee Evaluation Form, KSS score, SF-12 score, or femoral socket or tibial tunnel or socket widening, or narcotic consumption. The VAS pain score compared with baseline was significantly lower for the all-inside technique on day 1, on day 7, at 1.5 weeks, and at 24 months | 150 | 148 |
| **160** | Ma et al[176] | 2013 | Arthroscopy | To evaluate the clinical results of medial retinaculum plasty (MRP) and medial patellofemoral ligament reconstruction (MPFLR) with concomitant lateral retinacular | Clinical outcome based on Kujala score, Tegner activity score, and a subjective questionnaire | The measurement results for the congruence angle, patellar tilt angle, and patellar lateral shift decreased significantly from the pretreatment measurements to the normal range at the latest follow-up, without a statistically significant difference between the 2 groups (P > .05). The median Kujala score had significantly improved | 80 | 70 |

© 2021 Robinson NB et al. *JAMA Network Open*.

| | | | | | | | | |
|---|---|---|---|---|---|---|---|---|
| | | | | release with respect to imaging and functional results | | after surgery. However, no statistically significant difference was found between the 2 groups at the latest follow-up (P > .05) | | |
| **161** | Noh et al[177] | 2013 | Arthroscopy | To compare clinical outcomes after single-bundle anterior cruciate ligament (ACL) reconstruction with a free Achilles tendon allograft using either a transtibial or an anteromedial portal technique and then to quantify the difference in femoral tunnel position between these 2 approaches | Clinical outcome based on Lachman test, pivot shift test, International Knee Documentation Committee (IKDC) classification, Lysholm score, Tegner activity scale, and single leg hop (SLH) test | There were no significant differences between the 2 groups in results from the Lachman test, pivot shift test, IKDC classification, Tegner activity scale, and SLH test. The Lysholm score and SSD results in group 2 were superior to those in group 1 (P < 0.001). The femoral tunnel aperture was positioned more posteriorly in group 2 than in group 1 (P <0.001). Changes in the PCL index were greater in group 1 than in group 2 (P < 0.001) | 135 | 64 |
| **162** | Noh et al[178] | 2013 | Arthroscopy | To prospectively compare the outcomes of anterior cruciate ligament (ACL) reconstruction using bone-tendon Achilles allograft with those of free tendon Achilles allograft in 2 groups of active young men | Clinical outcome based on Lachman test, pivot-shift test, 1-leg hop test, The International Knee Documentation Committee classification, Lysholm score, Tegner activity scale and side-to-side difference | There were no statistically significant differences between the 2 groups for the Lachman test, pivot-shift test, 1-leg hop test, International Knee Documentation Committee classification, Lysholm score, and side-to-side difference (P > .05) | 96 | 72 |
| **163** | Takeda et al[179] | 2013 | Arthroscopy | To compare femoral and tibial tunnel placement, angle, and length between transtibial and anteromedial portal techniques for anatomic double-bundle anterior cruciate ligament (ACL) reconstruction | Femoral and tibial tunnel placement using 3-dimensional CT image reconstruction | Anteromedial bundle (AMB) and posterolateral bundle (PLB) femoral tunnels were placed significantly lower and deeper with the AMP technique (shallow/deep direction: 21% and 30%, high/low direction: 18% and 48%) than with the transtibial technique (25% and 34%, 12% and 43%) | 78 | 50 |
| **164** | Li et al[180] | 2014 | Arthroscopy | To evaluate whether posterior cruciate ligament reconstruction with the doublebundle (DB) technique improved stability of the knee compared with the single-bundle (SB) technique | Posterior stability evaluated with the KT-1000 arthrometer, clinical outcomes assessed with the Lysholm score, Tegner activity score, and International Knee Documentation Committee score | The Lysholm score was not significantly different between the 2 groups (P=0.447). The Tegner activity score improved significantly to 6.2±0.9 (range, 5 to 8) in the SB group and 6.8±1.2 (range, 5 to 9) in the DB group. The side-to-side difference in posterior translation decreased to 4.1±1.3 mm (range, 5.5 to 2.5 mm) in the SB group and 2.2±1.3 mm (range, 4.5 to 2.0 mm) in the DB group, and there was a significant difference between the 2 groups (P < 0.05). According to the International Knee Documentation Committee (both objective and subjective), the DB group had a better grade distribution (P <0.05) and had a statistically higher grade (71.6±6.7; range, 63 to 80) than the SB group (65.5±7.8; range, 56 to 75) (P < 0.05) | 69 | 50 |

© 2021 Robinson NB et al. *JAMA Network Open*.

| | | | | | | | | |
|---|---|---|---|---|---|---|---|---|
| **165** | Matsumoto et al[181] | 2014 | Arthroscopy | To compare the tunnel enlargement of double-bundle (DB) anterior cruciate ligament reconstruction (ACLR) with and without suturing of autologous ruptured tissue to hamstring graft in patients with subacute anterior cruciate ligament injury | Tunnel volume assessment by 3-dimensional multidetector row computed tomography (MDCT) | Tunnel volume enlargement between 3 weeks and 1 year after ACLR as assessed by 3-dimensional MDCT was significantly less for ACLR using ruptured tissue than for conventional ACLR, especially at the femoral site (P<0.05) | NR | 10 |
| **166** | Clement et al[182] | 2015 | Arthroscopy | To assess whether arthroscopic bursectomy and debridement of the calcific deposit, with or without subacromial decompression, influences the functional outcome of patients with calcific tendonitis | Pain visual analog score (VAS), short form (SF-12), disability arm shoulder and hand (DASH), and Constant score (CS) | There were no significant differences demonstrated between the groups for improvement in the pain VAS (P=.57), DASH (P=.93), SF-12 physical component score (P=.58), or CS (P=.27) at 1 year | 86 | 80 |
| **167** | Dong et al[183] | 2015 | Arthroscopy | To evaluate the clinical results of medial collateral ligament (MCL) anatomic ligament repair (ALR) and triangular ligament reconstruction (TLR) in treating acute grade III MCL injury with respect to imaging and functional results | Imaging and functional results of medial collateral ligament (MCL) anatomic ligament repair (ALR) and triangular ligament reconstruction (TLR) in treating acute grade III MCL injury using IKDC scores, ROM and medial opening evaluations in the short-term | The incidence of anteromedial rotatory instability in the TLR group (9.4%) decreased significantly compared with that in the ALR group (34.4%) (P < .05). All patients' IKDC subjective scores significantly improved after surgery. No statistically significant difference was found between the 2 groups at the last follow-up (P > .05) | 84 | 69 |
| **168** | Kim et al[184] | 2015 | Arthroscopy | To compare the clinical outcomes of arthroscopic in situ repair with the tear completion repair technique for partial-thickness rotator cuff tears | Clinical outcomes of arthroscopic in situ repair based on American Shoulder Elbow Society (ASES) score, Constant shoulder (CS) score, Simple shoulder (SS) score, and Korean shoulder (KS) score, and visual analog scale (VAS) | No significant difference in the clinical results was observed at any time between the groups. No difference of re-tear rate on articular-sided PT-RCT was observed between the groups (P=.34). | NA | 100 |
| **169** | Kim et al[185] | 2015 | Arthroscopy | To compare the clinical outcomes of immediate rotator cuff repair with capsular release and those of rotator cuff repair after the stiffness was treated with rehabilitative therapy | Clinical outcomes using American Shoulder and Elbow Surgeons score, Simple Shoulder Test score, Constant score, and visual analog scale score for pain and range of motion (ROM) | After treatment, there was significant improvement in ROM and functional scores in both groups, as measured at the last follow-up (P < .05). No statistical differences were found in clinical scores and ROM at the last followup (P > .05). On assessment of the magnetic resonance or ultrasound images taken 6 to 12 months postoperatively, the retear rate for the repaired | 80 | 72 |

© 2021 Robinson NB et al. *JAMA Network Open*.

| | | | | | | | | |
|---|---|---|---|---|---|---|---|---|
| | | | | | | cuff tendon in each group was 12.1% in group I and 13.4% in group II | | |
| **170** | Koga et al[186] | 2015 | Arthroscopy | To evaluate the mid-to long-term results of a randomized controlled trial of single-bundle (SB) versus doublebundle (DB) anterior cruciate ligament (ACL) reconstruction using a semitendinosus tendon | Clinical outcomes based on examination, KT-1000 arthrometer (MEDmetric, San Diego, CA) measurement, muscle strength, Tegner activity score, Lysholm score, subjective rating scale regarding patient satisfaction and sports performance level, graft retear, contralateral ACL tear, and additional meniscus surgery | The Lachman and pivot-shift test results were better in the DB group (P=.024 and P < .0001, respectively). KT measurements were better in the DB group (mean, 1.4 mm v 2.7 mm; P=.0023). The Tegner score was also better in the DB group (P=.033). There were no significant differences in range of motion, muscle strength, Lysholm score, subjective rating scale, graft retear, and secondary meniscal tear | 84 | 78 |
| **171** | Lubowitz et al[187] | 2015 | Arthroscopy | To compare anterior cruciate ligament (ACL) soft-tissue allograft reconstruction using suspensory versus aperture fixation | Knee anteroposterior (AP) stability measured using the KT-1000 device | The primary outcome measure, instrumented knee AP stability at 25° of knee flexion, showed no difference between groups (P=.61) at 24 months' follow-up | NR | 64 |
| **172** | Akamatsu et al[188] | 2016 | Arthroscopy | To assess whether a combined computed tomography (CT) based and image-free navigation system results in better coronal and sagittal alignment than the conventional method for performing opening-wedge high tibial osteotomy (OWHTO) and whether CT-based navigation results in acquisition of an accurate osteotomy plane | Coronal sagittal alignment using American Knee Society (AKS) knee and function scores | There was a significantly greater proportion of TPS outliers in the conventional (51.6%) than in the navigated group (12.9%) (P=0.001), and a significantly greater proportion within the normal range in both planes in the navigated (74.2%) than in the conventional group (48.4%) (P=0.037) | 73 | 62 |
| **173** | Barber et al[189] | 2016 | Arthroscopy | To compare the structural healing and clinical outcomes of triple-loaded single-row with suture-bridging double-row repairs of full-thickness rotator cuff tendons | Cuff integrity by magnetic resonance imaging (MRI) at 12 months postoperatively | A total of 3 of 20 single-row repairs and 3 of 20 double-row repairs (15%) had tears at follow-up MRI | 43 | 40 |
| **174** | Li et al[190] | 2016 | Arthroscopy | To evaluate posterior stability and proprioception after arthroscopic posterior cruciate ligament reconstruction with autograft, hybrid graft, and g-irradiated allograft | Posterior stability and proprioception | The differences in physical examination findings and subjective evaluations among the 3 groups were not significant (P > .05). However, a significant difference was detected in instrumented anteroposterior measurements, with more laxity shown in the g-irradiated | 116 | 90 |

© 2021 Robinson NB et al. *JAMA Network Open*.

| | | | | | | | | |
|---|---|---|---|---|---|---|---|---|
| | | | | | | allograft group than in the other 2 groups (P=.006). | | |
| **175** | Oh et al[191] | 2016 | Arthroscopy | To compare the clinical outcomes in patients with concomitant superior labrumebiceps complex (SLBC) lesions and rotator cuff tears who underwent arthroscopic rotator cuff repair, according to 3 different treatment methods (simple debridement, biceps tenotomy, or biceps tenodesis) for the SLBC lesions | Clinical outcome based on pain, range of motion, and functional scores - VAS pain scores, ASES scores, and ROM | Pain, range of motion, and functional scores significantly improved postoperatively in all 3 groups, with no significant differences across groups | 210 | 120 |
| **176** | Tian et al[192] | 2016 | Arthroscopy | To compare the clinical outcome of anatomic double-bundle (DB) anterior cruciate ligament (ACL) reconstruction with a hamstring tendon autograft versus fresh-frozen allograft | Clinical outcome based on physical examination, radiograph, functional knee ligament testing, KT-2000 arthrometer testing, Harner's vertical jump and Daniel's one-leg hop tests, Lysholm score, Tegner score, the International Knee Documentation Committee (IKDC) standard evaluation form, and Cincinnati knee score | No significant differences were found between the 2 groups according to the evaluations aforementioned except that patients in the Allo group had shorter operation time compared with the Auto group (P=.001) | 178 | 157 |
| **177** | Gupta et al[193] | 2017 | Arthroscopy | To compare mechanical stability, functional outcome, and level of return to sports activity in patients undergoing anterior cruciate ligament (ACL) reconstruction with a free hamstring graft versus a graft with preserved insertions at 2-year follow-up. | Difference between Activities of Daily Living Function Scale and Sports Function Scale (Cincinnati knee score), knee arthrometer (KT-1000) testing, and the Tegner activity scale | At 24 months, the mean side-to-side difference by KT-1000 testing was 1.4 in group 1 and 2.2 in group 2 (P < .0001); the mean Cincinnati knee score (Activities of Daily Living Function Scale and Sports Function Scale) was 418.5 (median, 420; range, 400-420) and 406.8 (median, 420; range, 350-420), respectively (P < .0001); and the mean difference between the preinjury and post-surgery Tegner level of sports activity was 0.3 and 1.08, respectively (P = .027). | NR | 110 |
| **178** | Krishna et al[194] | 2018 | Arthroscopy | To determine the extent to which a strategy of routinely preparing a 5-strand hamstring autograft would increase graft size in anterior cruciate ligament (ACL) reconstruction | Increase of graft size following anterior cruciate ligament (ACL) reconstruction | The mean increase in graft size achieved with the use of the 5-strand technique was 1.4±0.3 mm. In the 5-strand group, 24 of 32 (75%) patients had graft diameters exceeding 8 mm compared with 9 of 32 (28%) patients in the 4-strand group (P < .001) | 140 | 64 |
| **179** | Nam et al[195] | 2018 | Arthroscopy | To evaluate the effects of extensive bursectomy (EB) and | Clinical outcome based on VAS pain score | Group visual analog scale pain scores were not significantly different at any time (P > .05 for all) | 78 | 78 |

© 2021 Robinson NB et al. *JAMA Network Open*.

| | | | | | | | | |
|---|---|---|---|---|---|---|---|---|
| | | | | limited bursectomy (LB) during arthroscopic rotator cuff repair | | | | |
| **180** | Chiang et al[196] | 2019 | Arthroscopy | To investigate the tunnel enlargement rate and clinical function by comparing double-bundle anterior cruciate ligament reconstruction (ACLR) using different fixation devices | Tunnel enlargement | The BS group showed greater tunnel enlargement than the CB group, as well as a significantly increased rate of tunnel communication (P=.029) | 118 | 60 |
| **181** | Kaminski et al[197] | 2019 | Arthroscopy | To compare the effectiveness and safety of meniscal repair in 2 groups of patients: meniscal repair with biological augmentation using a bone marrow venting procedure (BMVP) of the intercondylar notch versus meniscal repair only | Rate of meniscus healing in the 2 groups assessed during a second-look arthroscopy | After 36 weeks, the meniscus healing rate was significantly higher in the BMVP-treated group than in the control group (100% vs. 76%, P=.0035). | 45 | 40 |
| **182** | Li et al[198] | 2019 | Arthroscopy | To compare the stability and clinical outcomes of 2 medial patellofemoral ligament reconstruction (MPFLR) techniques for the treatment of recurrent lateral patellar dislocation in adults | Recurrent patellar dislocations | No recurrent patellar dislocations occurred, except in 4 patients with instability symptoms in group A; however, no significant difference between the 2 groups was seen ($x^{2=}$2.503, P=.114). | 138 | 91 |
| **183** | Yamakado[199] | 2019 | Arthroscopy | To compare the clinical and imaging outcomes between the suture bridge technique (SB) and the medially based single-row technique (medSR) in patients with 1- to 3-cm tear sizes | Modified University of California, Los Angeles scoring system | No significant differences were found between the 2 groups across all measures at final follow-up | 473 | 106 |
| **184** | Matyas et al[200] | 2008 | EJVES | To compare the safety and efficacy of a bioresorbable paclitaxel-eluting wrap implanted with a synthetic vascular graft (treatment) versus the graft implanted alone (control) | Adverse event rate | The overall incidence of adverse events was similar in both groups | 109 | 109 |
| **185** | Meetwaldt et al[201] | 2008 | EJVES | To evaluate results of CEA with Fluoropassiv versus venous patch | Neurological event rate | Perioperative stroke rate was 2.4% in the Fluoropassiv group and 8.9% in the venous group (p=0.02; 1 regressive, 4 non-regressive strokes) | 88 | 87 |
| **186** | Scharn et al[202] | 2008 | EJVES | To compare long-term patency of Heparin-Bonded Dacron (HBD) and Human Umbilical Vein (HUV) vascular prostheses in above-knee femoro-popliteal bypass surgery | Primary patency | The cumulative primary patency rates were 79%, 66% and 58% at 1, 3 and 5 years postoperatively. Primary patency rates for HUV were 74%, 64% and 58% at 1, 3 and 5 years and 84%, 68% and 58% for HBD, respectively (log-rank test, p=0.745). | 137 | 129 |

| | | | | | | | | |
|---|---|---|---|---|---|---|---|---|
| **187** | Stehr et al[203] | 2008 | EJVES | To demonstrate the comparability of retrojugular access for carotid eversion endarterectomy compared to the conventional ventrojugular procedure | Major complications intra- and postoperatively, cranial nerve injuries as well as patient satisfaction (scale 1 to 10) | The study was stopped because of a significant increase in temporary ipsilateral vocal cord motility dysfunction in the retrojugular access group (31% vs. 6%, p=0.0014). This early postoperative impairment was, however, not statistically significant at the follow-up examination at 6 months (2.4% vs. 0%). No other significant differences concerning major complications (death or stroke), other cranial nerve injuries, wound healing, or patient satisfaction was observed neither in the early postoperative phase nor at follow up | 136 | 101 |
| **188** | REVAS[204] | 2009 | EJVES | To compare supragenicular bypass grafting versus remote endarterectomy (RSFAE) for surgical repair of Trans-Atlantic Inter-Society Consensus (TASC) C and D lesions of the superficial femoral artery (SFA) | Primary patency at 5 years | Primary patency after 1-year follow-up was 61% for RSFAE and 73% for bypass (p=0.094). Secondary patency was 79% for both groups | 357 | 118 |
| **189** | van Det et al[205] | 2009 | EJVES | To compare expanded polytetrafluoroethylene (ePTFE) prosthesis and collagen-impregnated knitted polyester (Dacron) for above-knee (AK) femoro-popliteal bypass grafts | Primary patency of the bypass graft at 2, 5 and 10 years after implantation | After 5 years, the primary, primary assisted and secondary patency rates were 36% (confidence interval (CI): 26-46%), 46% (CI: 36-56%) and 51% (CI: 41-61%) for ePTFE and 52% (CI: 42-62%) (p=0.04), 66% (CI: 56-76%) (p=0.01) and 70% (CI: 60-80%) (p=0.01) for Dacron, respectively. After ten years these rates were respectively 28% (CI:18-38%), 31% (CI:19-43%) and 35% (CI: 23-47%) for ePTFE and 28% (CI: 18-38%), 49% (CI: 37-61%) and 49% (CI: 37-61%) for Dacron | 228 | 228 |
| **190** | SCAMICOS[206] | 2010 | EJVES | To assess the use of a vein collar at the distal anastomosis to improve patency and limb salvage | Primary patency | At 3 years primary patency was 26% (95% CI 18-38) with a vein collar and 43 (33-58) without a vein collar for the femoro-popliteal bypass and 20 (11-38), and 17 (9-33) for femoro-distal bypass, respectively | 353 | 352 |
| **191** | Scandinavian Propaten[207] | 2011 | EJVES | To compare 1-year potencies of heparin-bonded PTFE ((Hb-PTFE) (Propaten)) grafts with those of ordinary polytetraflouroethylene (PTFE) grafts in a blinded, randomized, clinically controlled, multi-centre study | Primary patency | Overall, primary patency after 1 year was 86.4% for Hb-PTFE grafts and 79.9% for PTFE grafts (OR=0.627, 95% CI: 0.398; 0.989, p=0.043). Secondary patency was 88% in Hb-PTFE graftsand 81% in PTFE grafts (OR=0.569 (0.353; 0.917, p=0.020)) | 569 | 569 |
| **192** | Tiek et al[208] | 2012 | EJVES | To investigate differences between open and laparoscopic aortobifemoral bypass surgery for aorto-iliac | Visual analogue pain score | Visual pain scores and visual discomfort scores were both lower after laparoscopic surgery. Also return to normal daily activities was achieved earlier | 125 | 28 |

© 2021 Robinson NB et al. *JAMA Network Open*.

| | | | | | | | | |
|---|---|---|---|---|---|---|---|---|
| | | | | occlusive disease on postoperative morbidity and mortality | | | | |
| **193** | Vriens et al[209] | 2013 | EJVES | To compare externally supported thin wall knitted polyester (P-EXS) and externally unsupported thin wall knitted polyester (P-non-EXS) for above-knee (AK) femoro-popliteal bypass grafting | Primary patency rates at one and two years | The 1-year primary, primary assisted and secondary patency rates were 65%, 70% and 84%, respectively, for P-EXS and 76% (p=0.05), 82% (p=0.03) and 88% (p=0.35), respectively, for P-non-EXS. Two-year primary, primary assisted and secondary patency rates were 45%, 57% and 70%, respectively, for P-EXS and 62% (p=0.003), 75% (p=0.005) and 84% (p=0.02), respectively, for P-non-EXS | 267 | 266 |
| **194** | Eker et al[210] | 2013 | JAMA Surgery | To compare laparoscopic vs open ventral incisional hernia repair with regard to postoperative pain and nausea, operative results, perioperative and postoperative complications, hospital admission, and recurrence rate | Postoperative pain | Visual analog scale scores for pain and nausea, completed before surgery and 3 days and 1 and 4 weeks postoperatively, showed no significant differences between the 2 groups | 206 | 206 |
| **195** | Jang et al[211] | 2013 | JAMA Surgery | To evaluate the efficacy of PGA mesh in preventing POPF after distal pancreatectomy | Development of a clinically relevant POPF (grade B or C by the International Study Group grading system). | The rate of clinically relevant POPF (grade B or C) was significantly lower in the PGA group than in the control group (11.4% vs 28.3%, P = .04) | 107 | 107 |
| **196** | ESVIL[212] | 2015 | JAMA Surgery | To determine whether Ivor-Lewis esophagectomy is associated with increased postoperative complications compared with the Sweet procedure | Operative morbidity (any surgical or nonsurgical complications) | Although there was no significant difference between the 2 groups regarding the incidence of each single complication, a significantly higher morbidity rate was found in the Sweet group (62 of 150 (41.3%)) than in the Ivor-Lewis group (45 of 150 (30%)) (P = .04) | 1224 | 300 |
| **197** | Milone et al[213] | 2015 | JAMA Surgery | To validate the safety and efficacy of video-assisted ablation of pilonidal sinus | Time off work | In the minimally invasive treatment group, mean (SD) time off work was significantly less compared with the conventional treatment group (1.6 (1.7) vs 8.2 (3.9) days; P < .001) | 163 | 145 |
| **198** | Hakanson et al[214] | 2019 | JAMA Surgery | To determine whether partial fundoplication (PF) or total fundoplication (TF) is superior in laparoscopic antireflux surgery | Esophageal acid exposure at 3 years after surgery | At 3 years postoperatively, the median (interquartile range) esophageal acid exposure was reduced from 14.6% (9.8-21.9) to 1.8% (0.7-4.4) after PF and from 16.0% (10.4-22.7) to 2.5% (0.8-6.8) after TF (P = .31) | 1171 | 456 |
| **199** | Li et al[215] | 2019 | JAMA Surgery | To evaluate the short-term outcomes of patients with locally advanced gastric cancer who received either laparoscopic distal gastrectomy or open distal gastrectomy | 3-year recurrence-free survival rate | Not reported (interim analysis) | 125 | 96 |

© 2021 Robinson NB et al. *JAMA Network Open*.

| | | | | | | | | |
|---|---|---|---|---|---|---|---|---|
| **200** | ProphMesh[216] | 2019 | JAMA Surgery | To compare the incidence of incisional hernia among patients after prophylactic intraperitoneal mesh implantation with that among patients after standard abdominal closure | Incidence of incisional hernia 3 years after surgery | The cumulative incidence of incisional hernia was significantly lower in the mesh group compared with the control group (5 of 69 [7.2%] vs 15 of 81 [18.5%], log-rank test P = .03). | 612 | 169 |
| **201** | SEIPLUS[217] | 2019 | JAMA Surgery | To evaluate short-term outcomes of patients with advanced gastric cancer who received combined surgery and EIPL or surgery alone | 3-year overall survival | Not reported (interim analysis) | NR | 662 |
| **202** | Wei et al[218] | 2019 | JAMA Surgery | To compare the outcomes of different sequences of vessel ligation during surgery on the dissemination of tumor cells and survival in patients with non–small cell lung cancer | Changes in folate receptor–positive circulating tumor cells (FR+CTCs) after surgery and 5-year overall, disease-free, and lung cancer–specific survival | After surgery, an incremental change in FR+CTCs was observed in 26 of 40 patients (65.0%) in the artery-first group and 12 of 38 (31.6%) in the vein-first group (P = .003) (median change, 0.73 [interquartile range (IQR), −0.86 to 1.58] FU per 3mL vs −0.50 [IQR, −2.53 to 0.79] FU per 3 mL; P = .006). The vein-first group had significantly better outcomes than the artery-first group for 5-year overall survival (73.6%[95%CI, 64.4%-82.8%] vs 57.6%[95%CI, 48.4%-66.8%]; P = .002), disease-free survival (63.6% [95%CI, 55.4%-73.8%] vs 48.4%[95%CI, 40.0%-56.8%]; P = .001), and lung cancer–specific survival (76.4%[95%CI, 67.6%-85.2%] vs 59.9%[95%CI, 50.5%-69.3%]; P = .002) | 2531 | 86 |
| **203** | Chaudhary et al[219] | 2008 | JBJS | To compare the range of motion of the knee over the first two postoperative years between subjects who had received the ligament-substituting design and those who had received the ligament-retaining design | Active knee flexion and extension, measured with a goniometer | At two years postoperatively, the mean difference between the groups with regard to knee flexion was 0.03° (95% confidence interval, -5.9° to 6.0°) and the mean difference in knee extension was 1.0° (95% confidence interval, -0.36° to 2.4°) | 100 | 100 |
| **204** | Lachiewicz et al[220] | 2008 | JBJS | To compare the survival of two femoral components with similar geometry but substantially different surface finishes | Survival of the device defined as component removal for any reason or definite radiographic loosening | In the entire cohort of 219 hips, there was no significant difference (log rank p = 0.66) in survival, with the end point defined as component removal for any reason or definite radiographic loosening, between the precoated components (96.2%; 95% confidence interval, 90.9% to 100%) and the polished components (97.1%; 95% confidence interval, 93.8% to 100%) | 211 | 201 |
| **205** | Leung et al[221] | 2008 | JBJS | To compare external fixation combined with percutaneous pin fixation and plate fixation for the fixation of an intra-articular distal radial fracture | The Gartland and Werley point system | The results for the plate fixation group were significantly better than those for the external fixation and percutaneous pin fixation group according to the Gartland and Werley point | 137 | 137 |

© 2021 Robinson NB et al. *JAMA Network Open*.

| | | | | | | | | |
|---|---|---|---|---|---|---|---|---|
| | | | | | | system (p = 0.04) and the radiographic arthritis grading system (p = 0.01). | | |
| **206** | Pagnano et al[222] | 2008 | JBJS | To determine if patients recovered faster after two-incision total hip arthroplasty than after mini-posterior incision total hip arthroplasty | Functional outcomes and SF-12 score | The patients in the two-incision group recovered more slowly than did those in the mini-posterior-incision group as measured on the basis of the mean time to discontinue a walker or crutches, to discontinue all walking aids, and to return to normal daily activities. The clinical outcome as measured on the basis of the SF-12 scores was similar at both two months and one year postoperatively | 75 | 72 |
| **207** | Rodkey et al[223] | 2008 | JBJS | To compare clinical outcomes in patients receiving a collagen meniscus implant versus those treated with partial medial meniscectomy alone | New tissue growth | The 141 repeat arthroscopies done at one year showed that the collagen meniscus implants had resulted in significantly (p = 0.001) increased meniscal tissue compared with that seen after the original index partial meniscectomy. No differences were detected between the two treatment groups in the acute arm of the study | 494 | 311 |
| **208** | SPRINT[224] | 2008 | JBJS | To compare reamed and unreamed intramedullary nailing with regard to the rates of reoperations and complications in patients with tibial shaft fractures | Composite outcome including bone-grafting, implant exchange, and dynamization in patients with a fracture gap of <1 cm. Infection and fasciotomy were considered as part of the composite outcome, irrespective of the postoperative gap | 105 in the reamed nailing group and 114 in the unreamed nailing group experienced a primary outcome event (relative risk, 0.90; 95% confidence interval, 0.71 to 1.15) | 2974 | 1339 |
| **209** | Bettinson et al[225] | 2009 | JBJS | To compare the ten-year survivorship of metal-backed versus all-polyethylene stemmed tibial implants | Survisorship of the implant with revision surgery | Ten-year survivorship, with revision for any reason (or the time at which patients were documented as requiring revision but were unfit for surgery) as the end point, was 94.5% (95% confidence interval, 90.4% to 96.8%) for the all-polyethylene design and 96% (95% confidence interval, 92.6% to 97.8%) for the metal-backed design | 510 | 510 |
| **210** | Dai et al[226] | 2009 | JBJS | To define the effect of fusion on lumbar spine and patient-related functional outcomes | Loss of kyphosis correction and pain assessed by visual analog pain score | No significant difference in radiographic or clinical outcomes was noted between the patients managed with the two techniques | 91 | 73 |
| **211** | Dalury et al[227] | 2009 | JBJS | To identify differences in knee preference and clinical outcome measures in a series of patients who had undergone bilateral total knee arthroplasty with each knee randomized to one | Active range of motion, quadriceps strength, and Knee Society scores (pain score, function score, and total score) | There were no significant differences between the two groups with regard to the range of motion, quadriceps strength, or Knee Society scores | 40 | 37 |

© 2021 Robinson NB et al. *JAMA Network Open*.

| | | | | | | | | |
|---|---|---|---|---|---|---|---|---|
| | | | | of two different surgical approaches: patellar eversion and anterior tibial translation, or patellar subluxation and no tibial translation | | | | |
| **212** | Dunbar et al[228] | 2009 | JBJS | To compare an uncemented Trabecular Metal tibial component with a conventional cemented stemmed tibial component of the same design | The maximum total point motion, three translations, three rotations, and the proportion of implants at risk | A subset of the Trabecular Metal components migrated extensively in the postoperative period, but all stabilized by one year and the proportion considered to be at risk for early aseptic loosening was 0.0 (95% confidence interval, 0.0 to 0.12) in the group as a whole. Four cemented components were considered to be at risk for early aseptic loosening (proportion at risk, 0.19; 95% confidence interval, 0.08 to 0.4) | 103 | 70 |
| **213** | Gioe et al[229] | 2009 | JBJS | To compare mobile-bearing and fixed-bearing cruciate-substituting total knee arthroplasties of the same design | Range of motion, Knee Society scores (KSS), Western Ontario and McMaster Universities Osteoarthritis Index (WOMAC) scores, selected Medical Outcomes Study Health Survey Short Form-36 (SF-36) scores, and radiographic measures | There was no significant difference between the groups with regard to the mean postoperative range of motion (110.9° and 109.1°, respectively; p = 0.21), the mean KSS clinical score (90.4 and 88.2 points; p = 0.168), or the mean KSS pain score (44.9 and 43.1 points; p = 0.108) | 651 | 400 |
| **214** | KAT[230] | 2009 | JBJS | To evaluate the effects of several design features, including metal backing of the tibial component, patellar resurfacing, and a mobile bearing between the tibial and femoral components, on the function and survival of the implant | Functional status as measured with the Oxford Knee Score14 (OKS), quality of life as measured with the Short Form-12 (SF-12) and the EuroQol-5D-16,17 (EQ-5D), and intraoperative and postoperative complications including the need for additional surgery | There was no evidence of differences in clinical, functional, or quality-of-life measures between the randomized groups at two years | 4070 | 2374 |
| **215** | Kim et al[231] | 2009 | JBJS | To compare the ranges of motion of the knees of patients treated with a standard posterior cruciate-retaining total knee prosthesis in one knee and a high-flexion posterior cruciate-retaining total knee prosthesis in the other | Change in Knee Society Knee Score | The mean postoperative Knee Society and Hospital for Special Surgery knee scores were 93.7 and 89 points, respectively, for the knees with a standard posterior cruciate-retaining prosthesis, and they were 93.9 and 90 points, respectively, for the knees with a high-flexion posterior cruciate-retaining prosthesis | 59 | 59 |

© 2021 Robinson NB et al. *JAMA Network Open*.

| | | | | | | | | |
|---|---|---|---|---|---|---|---|---|
| **216** | McCalden et al[232] | 2009 | JBJS | To report the clinical and radiographic results, after a minimum of five years of follow-up, of a randomized, blinded, controlled trial comparing a conventional polyethylene with a first-generation highly cross-linked polyethylene | Femoral head penetration | The mean femoral head penetration rate in the first through fifth years was found to be significantly lower in the group treated with the highly cross-linked polyethylene (0.003 mm/yr (95% confidence interval, ±0.027)) than it was in the group treated with conventional polyethylene (0.051 mm/yr (95% confidence interval, ±0.022)) (p = 0.006) | 100 | 100 |
| **217** | Pajala et al[233] | 2009 | JBJS | To evaluate if augmentation with a down-turned gastrocnemius fascia flap would not provide better results than would end-to-end suture repair with use of the Krackow locking loop surgical technique | Clinical scoring method described by Leppilahti | The difference between the two groups with regard to the overall outcome was not significant (p = 0.68) | 83 | 60 |
| **218** | Rahme et al[234] | 2009 | JBJS | To compare the fixation of cemented keeled glenoid components with that of cemented in-line pegged glenoid components | Micromotion measured with radiostereometric analysis | No significant difference was detected between groups with regard to the average micromigration of the glenoid components at any of the time points | 28 | 28 |
| **219** | Baton et al[235] | 2010 | JBJS | To compare the outcome of treatment of unstable fractures of the proximal part of the femur with either a sliding hip screw or a long gamma nail | Reoperation within the first postoperative year | There was no significant difference between the reoperation rates for the two groups | 210 | 210 |
| **220** | Bonutti et al[236] | 2010 | JBJS | To compare the early clinical outcomes of mini-subvastus and the mini-midvastus approaches in total knee arthroplasty | Postoperative Knee Society scores | Comparisons of postoperative Knee Society scores between both approaches at the time of the two-year follow-up did not yield a significant difference in outcome | 91 | 51 |
| **221** | Choi et al[237] | 2010 | JBJS | To compare the outcomes of standard and high-flexion posterior-stabilized rotating-platform mobile-bearing total knee arthroplasties | Range of knee motion, knee scores, patients' abilities to perform deep knee flexion related activities, patient satisfaction and radiographic indices | There were no differences in knee scores, the number of knees able to perform deep-flexion-related activities, and patient satisfaction | 144 | 127 |
| **222** | Demey et al[238] | 2010 | JBJS | To assess the influence of femoral cementing on perioperative blood loss | The hemoglobin and hematocrit levels were recorded preoperatively and five days postoperatively for each patient | No differences between the two groups were identified with regard to hemoglobin and hematocrit levels, total measured blood loss, postoperative drainage amounts, or transfusion rates | 132 | 130 |
| **223** | Kim et al[239] | 2010 | JBJS | To compare clinical and radiographic results as well as | Change in Knee Society Knee Score | The mean postoperative Knee Society scores (95.5 points in the standard implant group, compared | 88 | 88 |

© 2021 Robinson NB et al. *JAMA Network Open*.

| | | | | | | | | |
|---|---|---|---|---|---|---|---|---|
| | | | | femoral component fit in patients receiving either a standard posterior cruciate-substituting LPS-Flex or gender-specific posterior cruciate substituting LPS-Flex total knee prosthesis | | with 96.5 points in the gender-specific implant group) and Hospital for Special Surgery knee scores (90.7 points in the standard implant group, compared with 91.2 points in the gender-specific implant group) were similar in both groups | | |
| **224** | MIS GEN II[240] | 2010 | JBJS | To assess the overall safety and effectiveness of a minimally invasive approach without the use of computer navigation in comparison with conventional knee arthroplasty | Total range of motion. Knee Society total and function scores, and visual analog scores for pain and activities of daily living | No significant difference was detected between the groups in any of the relevant clinical areas assessed: total range of motion, Knee Society total and function scores, and visual analog scores for pain and activities of daily living | 162 | 162 |
| **225** | Pospischill et al[241] | 2010 | JBJS | To compare a minimally invasive total hip arthroplasty approach with a traditional standard approach in terms of the effect on gait kinematics as demonstrated with gait analysis and electromyography | Three-dimensional gait analysis | There were no significant differences between the two groups with regard to the temporospatial variables of velocity, cadence, step length, and stride length at any tested time point | 71 | 40 |
| **226** | Delamarter et al[242] | 2011 | JBJS | To determine the twenty-four-month results of a clinical trial of the ProDisc-L total disc replacement as compared with spinal fusion for the treatment of degenerative disc disease at two contiguous vertebral levels from L3 to S1 | Composite regulatory FDA-guided end point consisting of ten criteria | AT 24 months eighty-seven (58.8%) of 148 patients in the total disc replacement group and thirty-two (47.8%) of sixty-seven patients in the arthrodesis group met all ten criteria and were considered a study success; non-inferiority was demonstrated between the two groups with use of a Blackwelder hypothesis with D = 0.125 ($p = 0.0008$) | 273 | 256 |
| **227** | Kim et al[243] | 2011 | JBJS | To determine whether augmentation of volar locking plate fixation with calcium phosphate bone cement has any benefit over volar locking plate fixation alone in patients older than sixty-five years of age who have an unstable distal radial fracture | Clinical assessments included determinations of grip strength, wrist motion, wrist pain, modified Mayo wrist scores, and Disabilities of the Arm, Shoulder and Hand (DASH) scores | No significant differences were observed between the groups with regard to the clinical outcomes at the three or twelve-month follow-up examination. No significant intergroup differences in radiographic outcomes were observed immediately after surgery or at the one-year follow-up visit | 88 | 50 |
| **228** | Kim et al[244] | 2011 | JBJS | To compare the clinical and radiographic results of posterior cruciate-sacrificing mobile-bearing total knee replacements with those of posterior-stabilized mobile-bearing total knee replacements | Change in Knee Society Knee Score | The mean postoperative Knee Society knee score (96 compared with 97 points) and Hospital for Special Surgery knee score (93 compared with 94 points) were similar between the two groups | 126 | 112 |
| **229** | MacDonald et al[245] | 2011 | JBJS | To compare functional and quality of-life indices and rates | Western Ontario Rotator Cuff (WORC) index | There were no differences in WORC or ASES scores between the groups that had arthroscopic | 102 | 86 |

© 2021 Robinson NB et al. *JAMA Network Open*.

| | | | | | | | | |
|---|---|---|---|---|---|---|---|---|
| | | | | of revision surgery in arthroscopic rotator cuff repair with and without acromioplasty | | cuff repair with or without acromioplasty at any time point | | |
| **230** | TROCHNAIL[246] | 2011 | JBJS | To prospectively compare the functional outcome of intramedullary nailing of the femur performed with use of a trochanteric and a piriformis fossa entry portal | WOMAC hip function score | The WOMAC score at three, six, and twelve months did not differ significantly between the piriformis fossa and trochanteric nailing groups | 114 | 114 |
| **231** | Zhang et al[247] | 2011 | JBJS | To compare the accuracy of prosthetic alignment between computer-assisted-navigation and conventional total knee arthroplasties | Alignment of the knee prosthesis in the coronal and sagittal planes | There was a significant difference between the two groups with regard to the alignment of the knee prosthesis in the coronal and sagittal planes. Nine knee implants (28%) in the conventional group, compared with no knee implants in the computer-navigation group, deviated >3° from the mechanical axis in the coronal plane | 35 | 32 |
| **232** | Zhu et al[248] | 2011 | JBJS | To compare the outcomes between locking intramedullary nails and locking plates in patients with a two-part surgical neck fracture | American Shoulder and Elbow Surgeons (ASES) scores | The average ASES score, median VAS score, and average strength of the supraspinatus were significantly better in the locking plate group (90.8 compared with 83.6 points (p = 0.021), 1.0 compared with 0.5 point (p = 0.042), and 77.4% compared with 64.3% (p = 0.032)) | 243 | 57 |
| **233** | Chammout et al[249] | 2012 | JBJS | To compare the results of total hip replacement with those of internal fixation over a long-term follow-up period of seventeen years | Hip function, evaluated with use of the Harris hip score | The Harris hip score was higher in the total hip arthroplasty group, with a mean difference of 14.7 points (95%confidence interval, 9.2 to 20.1 points; p < 0.001 (analysis of covariance)) during the study period | 1172 | 100 |
| **234** | Grewal et al[250] | 2012 | JBJS | To evaluate outcomes of the single and double-incision techniques for acute distal biceps tendon repair | American Shoulder and Elbow Surgeons (ASES) elbow score | There were also no differences in the final outcomes (at two years) between the two groups (p = 0.4 for ASES pain score, p = 0.10 for ASES function score, p = 0.3 for DASH score, and p = 0.4 for PREE score) | 96 | 91 |
| **235** | Gumina et al[251] | 2012 | JBJS | To evaluate the clinical and magnetic resonance imaging (MRI) results of arthroscopic rotator cuff repair with and without the use of platelet-leukocyte membrane in patients with a large posterosuperior rotator cuff tear | The difference between the preoperative and postoperative Constant scores and the repair integrity assessed by MRI according to the Sugaya classification | The only significant differences between the two groups involved the patient age and the preoperative and postoperative Constant scores; the differences in the Constant score were due to differences in the shoulder pain subscore. At a mean of thirteen months of follow-up, rotator cuff retears were observed only in the group of patients in whom the membrane had not been used, and a thin but intact tendon was observed more frequently in this group as well. The use of the membrane was associated with significantly better repair integrity (p = 0.04). | 87 | 80 |
| **236** | Howie et al[252] | 2012 | JBJS | To determine whether the incidence of dislocation one | Overall incidence of dislocation | Overall, at one year of follow-up, hips with a 36-mm femoral head articulation had a significantly | 2319 | 645 |

© 2021 Robinson NB et al. *JAMA Network Open*.

| | | | | | | | | |
|---|---|---|---|---|---|---|---|---|
| | | | | year after total hip arthroplasty is significantly lower in association with the use of a 36-mm femoral head articulation as compared with a 28-mm articulation | | lower incidence of dislocation than did those with a 28-mm articulation (1.3% (four of 299) compared with 5.4% (seventeen of 316); difference, 4.1% (95% confidence interval, 1.2% to 7.2%)) when controlling for the type of procedure (primary or revision) (p = 0.012) | | |
| **237** | Kim et al[253] | 2012 | JBJS | To determine whether computer-navigated total knee arthroplasty improves the clinical function, alignment, and survivorship of the components | Change in Knee Society Knee Score | Total knee scores, knee function scores, pain scores, WOMAC scores, knee motion, and activity scores did not show statistically significant differences between the two groups preoperatively or at the time of the final follow-up | 536 | 536 |
| **238** | Lapner et al[254] | 2012 | JBJS | To compare the functional outcomes and healing rates after use of single-row and double-row suture techniques for repair of the rotator cuff | Western Ontario Rotator Cuff Index (WORC) score at twenty-four months | The WORC score did not differ significantly between groups at any time point (p = 0.48 at baseline, p = 0.089 at three months, p = 0.52 at six months, p = 0.83 at twelve months, and p = 0.60 at twenty-four months) | 118 | 90 |
| **239** | Taylor et al[255] | 2012 | JBJS | To evaluate the use of cement for hemiarthroplasty to treat a displaced subcapital femoral neck fracture in elderly patients | Pain assessed by visual analog scale | The mean visual analog pain score at rest did not differ significantly between the groups | 301 | 160 |
| **240** | Jayasuriya et al[256] | 2013 | JBJS | To examine whether the geometry of the cemented femoral prosthesis affects the pattern of strain-adaptive bone remodeling in the proximal aspect of the femur after primary total hip arthroplasty | Change in proximal femoral bone mineral density over the first two postoperative years | Decreases in femoral bone mineral density were observed over the first year for all prosthesis designs, with no further loss during the second year. The decreases were similar in regional distribution and magnitude between the composite-beam and sliding-taper designs (p>0.05) | 562 | 120 |
| **241** | Mohammadi et al[257] | 2013 | JBJS | to compare the functional performances of soccer players after ACL reconstruction that was performed with either a bone-patellar tendon-bone or a semitendinosus and gracilis tendon graft | Strength, hop, and jump tests | The limbs with the ACL reconstruction in the STG group had greater values for quadriceps torque as well as on the triple-hop, crossover-hop, and jump-landing test (p < 0.01), but the STG and BPTB groups showed similar results in terms of hamstrings peak torque and the results of two other hop tests (p > 0.05) | 61 | 42 |
| **242** | TRIGEN INTERTAN[258] | 2013 | JBJS | To assess whether use of the TRIGEN INTERTAN nail, as compared with a sliding hip screw, resulted in less postoperative pain, improved functional mobility, and reduced surgical complication rates for patients with an intertrochanteric or subtrochanteric fracture | Pain assess by VAS at 12 months | Patients treated with an INTERTAN nail had slightly less pain at the time of early postoperative mobilization (VAS score, 48 versus 52; p = 0.042), although this did not influence the length of the hospital stay and there was no difference at three or twelve months | 842 | 697 |

© 2021 Robinson NB et al. *JAMA Network Open*.

| | | | | | | | | |
|---|---|---|---|---|---|---|---|---|
| **243** | Bennett et al[259] | 2014 | JBJS | To evaluate an uncemented implant (SR71) composed of a carbon-fiber-composite distal section and a porous-coated titanium-alloy proximal section, designed to improve proximal load transfer and provide good fixation | Bone mineral density as measured by dual x-ray absorptiometry (DXA) scan | 19 patients who had been treated with the SR71 stem and not lost to follow-up showed a significantly greater increase in proximal bone mineral density (Gruen zones 1 (p = 0.003) and 7 (p = 0.0007)) from baseline than did the twenty-two who had been treated with the Stability stem and not lost to follow-up. The Stability group showed a significantly greater increase in distal bone mineral density (Gruen zones 2 (p = 0.0004), 3 (p = 0.0001), and 5 (p = 0.0035)) compared with the SR71 group | 60 | 60 |
| **244** | Duivencoorden et al[260] | 2014 | JBJS | To investigate the clinical and radiographic mid-term results of closing-wedge and opening-wedge high tibial osteotomy when used to treat varus deformity | Hip-knee-ankle angle | Six years postoperatively, the mean hip-knee-ankle (HKA) angle (and standard deviation) was 3.2° ± 4.1° of valgus after a closing-wedge high tibial osteotomy and 1.3° ± 5.0° of valgus after an opening-wedge high tibial osteotomy (p = 0.343) | 92 | 92 |
| **245** | Glazebrook et al[261] | 2014 | JBJS | To compare the proximal opening wedge osteotomy of the first metatarsal with the proximal chevron osteotomy for the treatment of hallux valgus with an increased intermetatarsal angle | Clinical outcome scores of the Short Form-36, the American Orthopaedic Foot & Ankle Society forefoot questionnaire, and the visual analog scale for pain, activity, and patient satisfaction | No significant differences were found for any of the patients' clinical outcome measurements between the two procedures | 75 | 75 |
| **246** | Jenkins et al[262] | 2014 | JBJS | To evaluate if patients with knees surgically exposed using patellar lateral retraction would have comparable outcomes with patients with knees surgically exposed using patellar eversion | Quadriceps strength at 1 year postoperative | At one year postoperatively, quadriceps strength was not different between groups (p = 0.77), and the range of motion significantly improved (p < 0.01) from preoperative values by a mean value (and standard deviation) of 6° ± 17°, with no significant difference (p = 0.60) between groups | 274 | 120 |
| **247** | MIKRO[263] | 2014 | JBJS | A quadriceps-sparing (QS) subvastus technique of total knee arthroplasty was compared with medial parapatellar arthrotomy (MPPA) to determine which surgical technique led to better patient-reported function and less postoperative pain and opioid utilization | Early postoperative pain, opioid utilization, and functional recovery | No differences between groups were seen in opioid utilization, either during the acute hospitalization or in the eight weeks after surgery. The QS group reported significantly less pain at rest on postoperative day one and with activity on day three (p = 0.04 for each) | 311 | 129 |
| **248** | Reid et al[264] | 2014 | JBJS | To investigate if retracting rather than everting the patella results in quicker postoperative recovery and improved function in patients | Knee flexion | There was no significant difference in any of the outcomes at one year | 70 | 68 |

© 2021 Robinson NB et al. *JAMA Network Open*.

| | | | | | | | | |
|---|---|---|---|---|---|---|---|---|
| | | | | undergoing knee arthroplasty through a standard medial parapatellar approach | | | | |
| **249** | Woolson et al[265] | 2014 | JBJS | To compare postoperative component alignment between patients treated with custom instruments and those managed with traditional instruments | Knee component alignment as assessed by CT scan | A detailed analysis of intent-to-treat and per-protocol groups of study and control knees did not show any significant improvement in component alignment, including femoral component rotation in the axial plane, in the patients treated with the custom instruments | 83 | 63 |
| **250** | Zhang et al[266] | 2014 | JBJS | To assess the clinical outcomes of a minimally invasive longitudinal approach compared with the sinus tarsi approach in the surgical treatment of calcaneus fractures | American Orthopaedic Foot & Ankle Society scores | Patients in the STA group had better outcomes on all of the four subjective variables than patients in the minimally invasive longitudinal approach group. However, the two groups were only significantly different with regard to the variable of walking surface in the AOFAS scoring system | 233 | 167 |
| **251** | Andrade-Silva et al[267] | 2015 | JBJS | To compare plate fixation and elastic stable intramedullary nailing in the treatment of midshaft clavicular fractures | The six-month Disabilities of the Arm, Shoulder and Hand (DASH) score | The mean six-month DASH score was 9.9 points in the plate group and 8.5 points in the nail group ($p = 0.329$). Similarly, there were no differences in the twelve-month DASH and Constant-Murley scores | 147 | 59 |
| **252** | Malhotra et al[268] | 2015 | JBJS | To compare the emboli produced during computer assisted versus conventional total knee arthroplasty | Embolic burden based on intraoperative TEE at the time of intramedullary rod insertion and after tourniquet release | The mean embolic score was 6.21 points for the conventional technique group and 5.48 points for the computer-assisted surgery group ($p = 0.0161$) | 72 | 57 |
| **253** | POP[269] | 2015 | JBJS | To compare short and midterm results of open reduction and plate fixation with those of intramedullary nailing for displaced midshaft clavicular fractures | DASH score at 6 months postoperatively | No significant differences in the Disabilities of the Arm, Shoulder and Hand (DASH) or Constant-Murley score (3.0 and 96.0 points for the plate group and 5.6 and 95.5 points for the nailing group) were noted between the two surgical interventions at six months postoperatively | 994 | 120 |
| **254** | Reindl et al[270] | 2015 | JBJS | To compare the clinical and radiographic outcomes of patients who had been treated with a traditional extramedullary hip screw for an unstable (AO/OTA 31-A2) intertrochanteric hip fracture with those of patients who had been treated with the newer intramedullary device for the same injury | Lower Extremity Measure (LEM) | No significant differences were noted between the intramedullary and extramedullary treatment arms with regard to either the primary or the secondary clinical outcome tools. | 204 | 204 |
| **255** | Shareghi et al[271] | 2015 | JBJS | To compare the early-term femoral head penetration of an E-poly | Difference in proximal femoral head penetration | The median proximal penetration in the E-poly group was 0.04mm at three months, which increased to 0.06 mm at two years. Corresponding values for the ArComXL group were 0.03mm and | 456 | 61 |

© 2021 Robinson NB et al. *JAMA Network Open*.

| | | | | | | | | |
|---|---|---|---|---|---|---|---|---|
| | | | | liner with that of a heat-treated polyethylene liner, ArComXL | | 0.10 mm (comparison at two years: p = 0.30, Mann-Whitney test) | | |
| **256** | Bell et al[272] | 2016 | JBJS | To evaluate the accuracy of component positioning in unicompartmental knee arthroplasty comparing roboticassisted and conventional implantation techniques | The accuracy of the axial, coronal, and sagittal component positioning on computed tomographic scan | The accuracy of component positioning was improved with the use of the robotic-assisted surgical procedure, with lower root mean square errors and significantly lower median errors in all component parameters (p < 0.01) | 139 | 139 |
| **257** | Heesterbeek et al[273] | 2016 | JBJS | To compare the stability of cemented TKA implants with the stability of TKA implants fixed with the hybrid technique | Micromotion, measured with radiostereometric analysis using a uniplanar setup with ceiling-mounted x-ray tubes | At 24 months, no difference in median migration or the number of migrating components was found between the cemented and hybrid-fixation groups | 193 | 34 |
| **258** | Howie et al[274] | 2016 | JBJS | To compare the mean wear rates of 36-mm and 28-mm metal-on-XLPE articulations between 1 and 3 years postoperatively | Mean of proximal wear rate | Mean annual proximal wear rates between 1 and 3 years were 0.00 and 0.01 mm/yr for the 36 and 28-mm articulation cohorts, respectively | 622 | 56 |
| **259** | Verburg et al[275] | 2016 | JBJS | To compare the clinical and radiographic outcomes of the mini-midvastus (MMV) approach with the conventional approach for total knee arthroplasty at the 5-year follow-up | Knee Injury and Osteoarthritis Outcome Score (KOOS), the Oxford Knee Score (OKS), the Knee Society Score (KSS), and the Short Form (SF)-12 | No significance differences were detected between the groups with respect to the KOOS, OKS, KSS, and SF-12 scores determined at the 6-week and the 1, 2, and 5-year follow-up evaluations | 257 | 100 |
| **260** | Duckworth et al[276] | 2017 | JBJS | To compare the outcomes of tension-band wire (TBW) and plate fixation for simple isolated, displaced fractures of the olecranon | Disabilities of the Arm, Shoulder and Hand (DASH) score at 1 year | At 1 year, the DASH score for the TBW group (12.8) did not differ significantly from that of the plate group (8.5) (p = 0.315) | 184 | 67 |
| **261** | Andersen et al[277] | 2018 | JBJS | To compare clinical and radiographic results between patients who underwent stabilization of an acutely injured syndesmosis with a suture button (SB) and those treated with 1 quadricortical syndesmotic screw (SS) | The score on the American Orthopaedic Foot & Ankle Society (AOFAS) ankle-hindfoot scale | At 2 years, the median AOFAS score was higher in the SB group than in the SS group (96 (interquartile range, or IQR, 90 to 100) versus 86 (IQR, 80 to 96); p = 0.001), as was the median OMA score (100 (IQR, 95 to 100) versus 90 (IQR, 75 to 100); p < 0.001) | 196 | 97 |
| **262** | Strickland et al[278] | 2018 | JBJS | To evaluate the magnetic resonance imaging (MRI) appearance of the hip capsule in patients with femoroacetabular impingement (FAI) who | MRI Evaluation of the distance of separation across capsular fibers | At 6 weeks postoperatively, a continuous hip capsule (with no apparent capsulotomy defect) was observed in 8 hips treated with capsular repair and 3 hips without such a repair. Of the 19 hips with a discontinuous capsule at 6 weeks, 17 were available for follow-up at 24 weeks | 38 | 30 |

© 2021 Robinson NB et al. *JAMA Network Open*.

| | | | | | | | | |
|---|---|---|---|---|---|---|---|---|
| | | | | underwent simultaneous bilateral hip arthroscopy through an interportal capsulotomy with each hip randomized to undergo capsular repair or not | | postoperatively; all 17 demonstrated progression to healing, with a contiguous appearance without defects and no difference in capsular dimensions between treatment cohorts | | |
| **263** | Dolatowski et al[279] | 2019 | JBJS | To evaluate if hemiarthroplasty would be superior to screw fixation with regard to hip function, mobility, pain, quality of life, and the risk of a reoperation in elderly patients with a nondisplaced femoral neck fracture | Harris hip score (HHS) and timed “Up & Go” (TUG) test | There was no significant difference in hip function between the screw fixation and hemiarthroplasty groups, with a 24-month HHS (and standard deviation) of 74 ± 19 and 76 ± 17, respectively, and an adjusted mean difference of 22 (95% confidence interval [CI] = 26 to 3; p = 0.499) | 1524 | 219 |
| **264** | King et al[280] | 2019 | JBJS | To compare the union rates and functional outcomes of displaced and/or shortened clavicular shaft fractures treated with a flexible locked intramedullary nail or with an anatomically contoured locked plate | Union rate and the functional shoulder capacity, as measured with the use of the DASH and CS scores, at 1.5, 3, 6, and 12 months after the surgical procedure | A union rate of 100% was observed in both groups. DASH scores were similar between groups at 1.5, 3, and 6 months, whereas the nailing group had significantly better DASH scores at 12 months (p = 0.022); however, this difference had only a moderate effect size | 92 | 87 |
| **265** | Nam et al[281] | 2019 | JBJS | To compare the clinical outcomes of cemented and cementless versions of the same TKA design at an average of 2 years postoperatively | Oxford Knee Score | There were no differences in any clinical outcome measure at 4 to 6 weeks, 1 year, or an average of 2 years postoperatively (p = 0.1 to 0.9) between the cemented and cementless cohorts | 237 | 147 |
| **266** | Landford et al[282] | 2009 | Journal of Neurosurgery | To investigate if self-closing plastic scalp clips used for hemostasis on the skin edge might lead to localized microscopic tissue damage and subsequent delayed wound healing | Wound healing, with patients assessed at 3 and 6 weeks postoperatively | The results showed no significant difference in wound healing between the 2 groups at either 3 weeks (OR 0.55, 95% CI 0.27–1.11; p = 0.09) or 6 weeks (OR 0.79, 95% CI 0.39–1.58; p=0.50) | NR | 143 |
| **267** | Xiao et al[283] | 2012 | Journal of Neurosurgery | To determine whether patients who have suffered a massive ICH can benefit from a more urgently performed decompressive procedure | Pupil response improvement/ worsening | A worsening pupil reflex before decompression was observed in no Group A patient and in 9 Group B patients | NR | 36 |
| **268** | Hutter et al[284] | 2014 | Journal of Neurosurgery | To investigate whether the addition of TachoSil on top of the dural suture reduces postoperative CSF leakage compared with dural suturing alone and to assess the frequency and risk factors for | The incidence of CSF leakage, defined as CSF collection or any open CSF fistula within 30 days | Cerebrospinal fluid leakage (9.7% vs 17.2%, OR 0.53 (95% CI 0.23–1.15), p = 0.108) and infection (OR 0.18 (95% CI 0.01–1.18), p = 0.077) occurred less frequently in the study group than in the control group | 289 | 241 |

© 2021 Robinson NB et al. *JAMA Network Open*.

| | | | | | | | | |
|---|---|---|---|---|---|---|---|---|
| | | | | dural leakage and potentially related complications after elective craniotomy | | | | |
| **269** | Schucht et al[285] | 2015 | Journal of Neurosurgery | To compare rates of shunt failure in patients who underwent laparoscopic surgery for peritoneal catheter placement with rates in patients who underwent traditional mini-laparotomy | The rate of overall shunt complication or failure within the first 12 months after surgery | The overall shunt complication/failure rate was 15% (9 of 60 cases) in the laparoscopic group and 18.3% (11 of 60 cases) in the mini-laparotomy group (p = 0.404) | 174 | 120 |
| **270** | Welling et al[286] | 2015 | Journal of Neurosurgery | To compare the clinical, functional, and aesthetic results of 2 surgical techniques, pterional (PT) and minipterional (MPT) craniotomies, for microsurgical clipping of anterior circulation aneurysms | Satisfaction with the aesthetic results | Satisfaction in terms of aesthetic result was observed in 19 patients (79%) in the MPT group and 13 (52%) in the PT group (p = 0.07) | NR | 58 |
| **271** | Fonoff et al[287] | 2016 | Journal of Neurosurgery | To demonstrate the feasibility of simultaneous bilateral procedures during the implantation of deep brain stimulation (DBS) leads | The operating time required for the cranial portion of the bilateral DBS implantation procedure | Overall, a reduction of 38.51% in total operating time for the simultaneous bilateral group (136.4 ± 20.93 minutes) as compared with that for the traditional consecutive approach (220.3 ± 27.58 minutes) was observed | NR | 57 |
| **272** | Chugh et al[288] | 2017 | Journal of Neurosurgery | To determine whether use of a surgical rehearsal platform in aneurysm surgery is helpful in decreasing aneurysm dissection time and clip manipulation of the aneurysm | Assessment of clipping attempts from a sample of neurosurgical video recordings | The mean (± SD) amount of operative time per clip used was 920 ± 770 seconds in the SRP group and 1294 ± 678 seconds in the control group (p = 0.05) | NR | 40 |
| **273** | Honeybul et al[289] | 2017 | Journal of Neurosurgery | To see whether it would be cost-effective to use titanium as a primary reconstructive material | Implant Failure Requiring Reoperation at 12 months | In the titanium group, no patient was considered to have partial or complete cranioplasty failure at 12 months of follow-up (p = 0.002) and none needed revision (p = 0.053). There were 2 deaths unrelated to the cranioplasty, one in each arm of the trial. Among the 31 patients who had an autologous cranioplasty, 7 patients (22%) had complete resorption of the autologous bone such that it was deemed a complete failure | 105 | 64 |
| **274** | Lindner et al[290] | 2017 | Journal of Neurosurgery | To compare local and systemic infections related to custom-made titanium and hydroxyapatite (HA) implants within the first 6 months after implantation | To compare local implant associated infections and/or systemic infections in both study arms within the first 6 months after implantation | The rate of local implant–associated wound infection in the HA group was 2 of 26 (7.7%) patients and 5 of 24 (20.8%) patients in the titanium group (p = 0.407). Systemic inflammation within 6 months after operation affected none of the patients in the HA group and 4 of 24 (37.5%) patients in the titanium group (p = 0.107) | NR | 50 |

© 2021 Robinson NB et al. *JAMA Network Open*.

| | | | | | | | | |
|---|---|---|---|---|---|---|---|---|
| **275** | Vieira et al[291] | 2018 | Journal of Neurosurgery | To conduct a prospective randomized controlled trial comparing 2 techniques for performing DC: with watertight duraplasty and without watertight duraplasty (rapid-closure DC) | Incidence of surgical complications (CSF leak, wound infection, brain abscess, or subgaleal fluid collections) | There were 9 surgical complications (5 in the control group and 4 in the test group), with no significant differences between the groups | NR | 55 |
| **276** | Heyligers et al[292] | 2008 | Journal of Vascular Surgery | To examine the systemic effects of the endoluminal heparin and to address whether graft implantation results in (1) a measurable reduction of systemic markers of hemostasis activation compared with control grafts and (2) antibody formation against heparin, potentially responsible for heparin-induced thrombocytopenia (HIT) | Markers of in vivo activation of platelets and blood coagulation | No statistical differences were observed in any of the markers of in vivo activation of platelets and blood coagulation between patients receiving Propaten or control ePTFE. Moreover, no antibodies against heparin could be demonstrated up to 6 weeks after implantation | 20 | 20 |
| **277** | Keuter et al[293] | 2008 | Journal of Vascular Surgery | To compare brachial-basilic fistula (BBAVF) and prosthetic implantation in patients without the possibility for radial-cephalic AV fistula (RCAVF) or brachial-cephalic AV fistula (BCAVF) | Primary patency | Primary and assisted-primary 1-year patency rates were significantly higher in the BBAVF group: 46%±7.4% vs 22%±6.1% (P=.005) and 87%±5.0% vs 71%±6.7% (P=.045) for the BBAVF and PTFE group, respectively | NR | 105 |
| **278** | AbuRahma et al[294] | 2010 | Journal of Vascular Surgery | To compare the results of routine (RS) vs selective shunting (SS) based on stump pressure (SP) | Perioperative complication rates | The overall perioperative complication rates were 8.3% in RS (2 TIA, 3 hemorrhage, 1 myocardial infarction (MI), and 1 asymptomatic carotid thrombosis) vs 7.8% in SS (2 strokes, 1 TIA, 3 hemorrhage, 1 MI, and 1 congestive heart failure; P=.917) | 200 | 200 |
| **279** | Kennealey et al[295] | 2011 | Journal of Vascular Surgery | To compare the standard cuffed expanded polytetrafluoroethylene (ePTFE) graft with the bovine carotid artery (BCA) graft | Primary, assisted primary, and secondary patency | Although there was no significant difference in secondary patency rates, primary and assisted primary patency rates were significantly higher in BCA than in the ePTFE grafts (60.5% vs 10.1% and 60.5% vs 20.8% at 1 year, respectively) | 110 | 57 |
| **280** | Morosetti et al[296] | 2011 | Journal of Vascular Surgery | To compare the outcomes of BBAVF and AV graft (AVG) in patients undergoing long-term hemodialysis in whom there was no other possibility of creating a VA | Primary patency (PP) and secondary patency (SP) rates | PP and SP rates were higher for BBAVF than for AVG, although this was not statistically significant for SP | 57 | 57 |
| **281** | Stollwerck et al[297] | 2011 | Journal of Vascular Surgery | To validate if ePTFE grafts have less dilation when compared with polyester grafts in a long- | Graft dilatation at discharge and at two follow-up assessments | Mean ± standard deviation dilatation of the midgraft segment was 1%±5% (ePTFE/Gore), 10%±9% (polyester/Braun), and 7%±8% | 199 | 199 |

© 2021 Robinson NB et al. *JAMA Network Open*.

| | | | | | | | | |
|---|---|---|---|---|---|---|---|---|
| | | | | term follow-up setting and to present data on graft dilatation and clinical results | | (polyester/Vascutek) (P < .001) at discharge; 8%±11% (ePTFE/Gore), 24%±7% (polyester/Braun), and 20%±13% (polyester/Vascutek; P < .001) after 12 months; and 19%±21% (ePTFE/Gore), 33%±22% polyester/Braun), and 23%±19% (polyester/Vascutek; (P < .001) after 6 years | | |
| **282** | Aitken et al[298] | 2015 | Journal of Vascular Surgery | To evaluate if a hybrid interrupted-continuous suturing technique (as used in many microsurgical procedures) may improve outcomes in fistulas constructed from small vessels | Primary patency at 6 weeks (assessed by a blinded observer for the presence of thrill and bruit) | Primary patency at 6 weeks was higher in the hybrid interrupted-continuous suturing technique group (71% vs 47%; P=.01) | 93 | 90 |
| **283** | FINEST[299] | 2015 | Journal of Vascular Surgery | To assess the clinical outcome of heparin-coated and standard vascular grafts | Primary patency of the study graft | The primary patency rates at 6 months were 86.4% for the FUSION BIOLINE heparin-coated vascular graft group compared with 70.0% for the standard ePTFE group, a difference of 16.4% (95% confidence interval, 2.7%-29.9%; P=.006), and the respective rates at 12 months were 76.5% and 67.0% (95% confidence interval, L4.8% to 23.0%; P=.05) | 209 | 209 |
| **284** | Shemesh et al[300] | 2015 | Journal of Vascular Surgery | To compare the patency of standard grafts with heparin-bonded grafts | Primary, assisted primary, and secondary patency for heparin bonded grafts vs standard grafts | Primary patency was 35% and 14% for heparin-bonded grafts and 29% and 12% for standard ePTFE grafts at 6 and 12 months, respectively (P=.48). Secondary patency was 83%, 83%, and 81% for heparin-bonded grafts and 81%, 73%, and 68% for standard grafts at 12, 24, and 36 months, respectively (P=.33) | 175 | 160 |
| **285** | Damgaard et al[301] | 2008 | JTCVS | To compare outcomes in patients undergoing total arterial revascularization during coronary artery bypass grafting | Angiographic 1- and 5-year distal anastomotic patency and cardiac event-free survival at 1, 5, and 10 years postoperatively | Three months' follow-up for the arterial versus conventional groups showed the following: deaths: 1 (0.6%) versus 0; stroke: 3 (1.9%) versus 3 (1.8%); myocardial infarction: 6 (3.7%) versus 4 (2.4%); sternal wound reoperation: 4 (2.5%) versus 0 (P=.054); arm and leg wound complications requiring hospitalization: 3 (1.9%) versus 6 (3.5%) (P=.50), respectively | 630 | 331 |
| **286** | Droghetti et al[302] | 2008 | JTCVS | To evaluate 2 different surgical techniques (staple versus collagen patch) for the completion of interlobar fissures during pulmonary lobectomy to establish which is superior in preventing air leakage | To assess the percentage of demonstrated intraoperative alveolar air leak effectively sealed after application of the patch (TachoSil) in ES and to compare the proportion of patients incthe experimental and control groups who were | Statistically significant reductions of air leakage were found in the ES group in the overall incidence of air leaks (50%vs 95%, P=.0001), duration of air leaks (1.7 days vs 4.5 days, P=.003), and procedure costs (425 euros vs 630.5 euros, P=.0001) | NR | 40 |

© 2021 Robinson NB et al. *JAMA Network Open*.

| | | | | | | | | |
|---|---|---|---|---|---|---|---|---|
| | | | | | free of air leaks throughout hospitalization | | | |
| **287** | Falk et al[303] | 2008 | JTCVS | To determine how preservation of leaflet structure in combination with premeasured neochordae compares with the widely adopted technique of leaflet resection | Valvular hemodynamics and orifice size (the postoperative MV orifice area of 0.5 cm2 or more and a difference in leaflet coaptation length of 1.5 mm or more) | Intraoperative transesophageal echocardiography showed a significantly longer line of mitral valve leaflet coaptation after implantation of loops (7.6±3.6 mm) than after resection (5.9±2.6 mm; P=.03). Early and mid-term echocardiographic follow-up revealed excellent valve function in the majority of patients, with no significant difference in mitral orifice area (3.6±1.0 $cm^2$ vs 3.7±1.1 $cm^2$, P=0.4) | 896 | 129 |
| **288** | Glineur et al[304] | 2008 | JTCVS | To compare the clinical, functional, and angiographic evolution of saphenous vein versus right gastroepiploic artery grafts at 6 months and 3 years | Major adverse cerebrocardiovascular events and proportion of grafts patent or functional at follow-up angiography | At follow-up there was no significant difference in major adverse cerebrocardiovascular events between the 2 groups. At the 6-month angiographic follow-up, 91% of the anastomoses in the right gastroepiploic artery group and 95% of the anastomoses in the saphenous vein graft group were controlled patent (P=.92) | 1397 | 244 |
| **289** | Guenzinger et al[305] | 2008 | JTCVS | To evaluate the impact of complete supraannular positioning of mechanical aortic bileaflet valves | Early hemodynamic and clinical performance of 2 mechanical aortic valve prostheses during rest and exercise, to analyze the impact of complete supraannular valve positioning, and to evaluate prosthesis-specific differences in valve sizing and valve size labeling | By grouping the data on the basis of a patient's tissue annulus diameter, no significant difference of either valve was detected with regard to mean pressure gradient and effective orifice area index at rest | NR | 80 |
| **290** | Kamiya et al[306] | 2008 | JTCVS | To investigate the influence of skeletonized internal thoracic artery harvesting on the sternal microcirculation in the perioperative phase | Oxygen saturation and blood flow at the presternal and retrosternal sides in the upper, middle, and lower parts | Skeletonization had no advantage in maintaining presternal microcirculation. Retrosternal microcirculation also deteriorated at all measurement points after internal thoracic artery harvesting in both groups. However, the deterioration of the retrosternal microcirculation was significantly less in group 1 at the middle and lower sternum | NR | 24 |
| **291** | Narang et al[307] | 2008 | JTCVS | To compare stentless and stented bioprostheses | Clinical outcomes, hemodynamic performance, and postoperative left ventricular mass regression | At 18±3 months postoperatively, the effective orifice area was greater in group A versus group B. Left ventricular ejection fraction, left ventricular mass index, functional class, and mean gradient were similar in patients of subgroup I (left ventricular ejection fraction .50%) from both groups | NR | 62 |

© 2021 Robinson NB et al. *JAMA Network Open*.

| | | | | | | | | |
|---|---|---|---|---|---|---|---|---|
| **292** | EPIC[308] | 2009 | JTCVS | To evaluate the efficacy of the PAS-Port device which allows an automated proximal anastomosis to be performed without aortic clamping | Angiographic patency (<50% stenosis) 9 months after surgical intervention | The 9-month graft patency was 82.0% (150/183) for hand-sewn and 80.3% (147/183) for PAS-Port grafts. The patency rate of PAS-Port anastomoses was statistically noninferior to that of hand-sewn anastomoses (95% lower confidence limit for difference, -7.95%) | 589 | 220 |
| **293** | Fattouch et al[309] | 2009 | JTCVS | To evaluate the impact of the off-pump technique on clinical results | The incidence of in-hospital death and outcomes (low cardiac output syndrome, prolonged mechanical and pharmacologic cardiac support, prolonged mechanical ventilation support, and postoperative length of stay in intensive care unit and hospital) | In-hospital mortality was 7.7% (5 patients) in the on-pump group and 1.6% (1 patient) in the off-pump group (P=.04). Statistically significant differences were found between the 2 groups concerning the incidence of low cardiac output syndrome (P=.001), time of inotrope drugs support (P=.001), time of mechanical ventilation (P=.006), reoperation for bleeding (P=.04), intensive care unit stay (P= .01), and in-hospital stay (P=.02) | NR | 128 |
| **294** | Formica et al[310] | 2009 | JTCVS | To verify the systemic inflammatory response, inflammatory myocardial damage, and early clinical outcome in coronary surgery with the miniaturized extracorporeal circulation system or on the beating heart | To analyze 1) the myocardial damage: troponin T (TnT), myoglobin, total creatine kinase (CK), and CK mass 2) the inflammatory response: IL-6, TNF-α, C-reactive protein, leukocyte, neutrophil, and monocyte cell counts; 3) the hemodilution: hematocrit and hemoglobin; and 4) the coagulative disorder: fibrinogen and platelet count | Release of interleukin-6 was higher in the off-pump coronary artery bypass grafting group 24 hours after the operation (P=.03), whereas levels of tumor necrosis factor-α were not different in both groups. Cardiac release of interleukin-6, tumor necrosis factor-a, and blood lactate were not different in both groups. Release of troponin T was not significantly different in both groups. Levels of creatine kinase mass were statistically higher in the miniaturized extracorporeal circulation group than in the off-pump coronary artery bypass grafting group, but only at the end of the operation (P<.0001). Hemoglobin levels were significantly higher in the miniaturized extracorporeal circulation group than in the off-pump coronary artery bypass grafting group after 24 hours (P= .01) | NR | 60 |
| **295** | POINT[311] | 2009 | JTCVS | To evaluate the effect of adding mitral valve repair to coronary artery bypass grafting on clinical outcomes and left ventricular remodeling in patients who underwent coronary artery bypass grafting alone versus coronary artery bypass grafting plus mitral valve repair | To evaluate the effect of adding MVR to CABG on the clinical status of patients measured based on New York Heart Association (NYHA) functional class and on postoperative reversal of left ventricular remodeling measured based on left ventricular end-systolic diameter | A significant difference was found between the 2 groups with regard to mean New York Heart Association class (P<.0001), left ventricular end-diastolic diameter (P<.01), left ventricular end-systolic diameter (P<.01), pulmonary arterial pressure (P<.0001), and left atrial size (P<.01) | 132 | 102 |

© 2021 Robinson NB et al. *JAMA Network Open*.

| | | | | | | | | |
|---|---|---|---|---|---|---|---|---|
| | | | | | (LVESD), left ventricular end-diastolic diameter (LVEDD), and left ventricular ejection fraction (LVEF) | | | |
| **296** | Sakwa et al[312] | 2009 | JTCVS | To determine differences in blood loss and transfusion associated with a minimized cardiopulmonary bypass circuit versus a standard bypass circuit | Differences in blood loss and transfusion | Hematocrit, equivalent at baseline, was higher in minimized circuit cohort at lowest point during cardiopulmonary bypass (31.5%±3.9% vs. 25.5%±3.7%), after protamine (31.6%±3.9% vs 29.2%±3.7%), and on intensive care unit arrival (35.2%±4.1% vs 31.8%±3.5%, P<.001). Fewer red blood cells (148 vs 19 units) were given in minimized circuit group (P<.0001) | NR | 199 |
| **297** | Stand in Y Mammary[313] | 2009 | JTCVS | To determine if the use of 2 arterial conduits rather than a single conduit in multivessel coronary artery bypass grafting significantly improves results despite the concomitant use of saphenous vein grafts and whether any among different configurations of composite grafts (left/right thoracic arteries and radial artery) offers an advantage over the others | In-hospital outcomes (mortality rate and morbidity), 2-year freedom from all-cause death, and adverse cardiac event–free survival (adverse cardiac events included cardiac death, acute myocardial infarction, recurrent angina, graft occlusion at coronary angiography, redo coronary surgery, or percutaneous transluminal coronary angioplasty) | The rate of cerebrovascular complications was not statistically lower among patients receiving 2 arterial grafts. At 2 years, overall survival was not significantly different among groups (P=.59). Cardiac event–free survival was significantly better in patients receiving 2 arterial grafts versus control subjects (P<.0001), even among elderly patients (P=.022). The 3 investigated strategies using 2 arterial conduits were similar concerning early and midterm results | 1769 | 815 |
| **298** | Belcher et al[314] | 2010 | JTCVS | To compare BioGlue and Vivostat in the control of postoperative air leak | Duration of air leak, time to intercostal drain removal, and length of hospital stay | Median duration of air leak was 3 (0–32) days versus 2 (0–33) days for patients who received BioGlue and Vivostat, respectively (P=.677). Time to intercostal drain removal was 5 (1–32) days in the BioGlue group compared with 5 (1–34) days for the Vivostat group (P=.473). Median hospital stay was 8 (3–22) days versus 7 (2–29) days for the BioGlue and Vivostat groups, respectively (P=.382) | 378 | 103 |
| **299** | Markman et al[315] | 2010 | JTCVS | To evaluate the incidence and severity of pain by using skeletonized internal thoracic artery harvesting rather than pedicled harvesting | The area of harvest dysesthesia, expressed as a percentage of the hemithoracic area (7 weeks, 21 weeks) | The incidence of harvest dysesthesia at 7 weeks was 14% in the skeletonized group versus 50% in the pedicled group (P=.02). These differences were not sustained at 21 weeks, as the median area of harvest dysesthesia in both groups was 0% (P=.89) and the incidence was 24% and 25% in the skeletonized and pedicled groups, respectively (P=1.0) | NR | 41 |

© 2021 Robinson NB et al. *JAMA Network Open*.

| | | | | | | | | |
|---|---|---|---|---|---|---|---|---|
| **300** | Ponce Gonzalez et al[316] | 2010 | JTCVS | To compare the long-term effects of conventional and simplified thoracic sympathectomy on cardiopulmonary function | Forced spirometry, body plethysmography, measurement of the diffusing capacity of the lung for carbon monoxide (DLCO), and exercise tests at 1 year | No significant differences were found between the conventional and simplified thoracic sympathectomy groups | NR | 32 |
| **301** | Roshanali et al[317] | 2010 | JTCVS | To lower the failure rate of tricuspid repair (TR) for functional tricuspid regurgitation by reducing leaflet tethering via pericardial patch augmentation when the preoperative probability of recurrence was high | Failure rate of TR (residual tricuspid regurgitation in tricuspid valve augmentation) | Postoperative tricuspid regurgitation was different between the groups (P<.05): 16.0%and 28.0%of patients in the De Vega group, 8.0%and 14.0%of patients in the ring annuloplasty group, 4.0%and 10.0%of patients in the De Vega pericardial patch augmentation group, and 2.0% and 8.0% of patients in the ring annuloplasty pericardial patch augmentation group had postoperative tricuspid regurgitation at 1-month and 1-year follow-up, respectively | NR | 210 |
| **302** | Salehi Omran et al[318] | 2010 | JTCVS | To assess the prophylactic effect of ventral cardiac denervation on reducing atrial fibrillation after coronary artery bypass grafting | Atrial fibrillation incidence | Atrial fibrillation incidence was significantly different between the groups (P=.025), with an incidence of 20.9%in the ventral cardiac denervation group and 10% in the control group. Atrial fibrillation occurred in 34 of the 220 patients, and ventral cardiac denervation was considered as a variable to evaluate its possible role in the prevention of postoperative atrial fibrillation | NR | 220 |
| **303** | Wiklund et al[319] | 2010 | JTCVS | To evaluate the clinical and angiographic outcomes of a second-generation anastomotic device used for saphenous vein grafts | 1-year patency for vein grafts anastomoses | The 1-year patency rate for study grafts constructed with the anastomotic connector was 92.2% (118/128) and for hand-sutured grafts, 91.7% (121/132) | NR | 151 |
| **304** | ACSOG Z0030[320] | 2011 | JTCVS | To determine whether mediastinal lymph node dissection improves survival compared with mediastinal lymph node sampling in patients undergoing resection for N0 or non-hilar N1, T1, or T2 non–small cell lung cancer | Overall survival (5 years) | The median survival is 8.1 years for mediastinal lymph node sampling and 8.5 years for mediastinal lymph node dissection (P=.25). The 5-year disease-free survival was 69% (95%confidence interval, 64–74) in the mediastinal lymph node sampling group and 68% (95% confidence interval, 64–73) years in the mediastinal lymph node dissection group (P=.92) | NR | 1111 |
| **305** | Al-Rashidi et al[321] | 2011 | JTCVS | To compare the effectiveness, time required for de-airing, and safety of a newly developed de-airing technique for open left heart surgery (Lund technique) with a standardized carbon dioxide insufflation technique | The severity of gas emboli observed on transesophageal echocardiography and the number of microembolic signals recorded by transcranial Doppler | The severity of gas emboli observed on transesophageal echocardiography and the number of microembolic signals recorded by transcranial Doppler were significantly lower in the Lund group during the de-airing procedure (P = .00634) and in the first 10 minutes after weaning from cardiopulmonary bypass (P = .000377) | NR | 20 |

© 2021 Robinson NB et al. *JAMA Network Open*.

| | | | | | | | | |
|---|---|---|---|---|---|---|---|---|
| **306** | Bonacchi et al[322] | 2011 | JTCVS | To evaluate the χ-configuration, a new cannulation strategy for VV-ECMO | Efficacy of blood oxygenation was obtained by gas–blood analysis, by blood samples obtained at arterial, central venous, and pulmonary artery lines, and by ECMO inflow and outflow lines | In group NS, on-ECMO time, post-ECMO mechanical ventilation time, and ECMO overall results were significantly better than in group C. During high-flow VV-ECMO, pulmonary and systemic arterial oxygen saturation and arterial oxygen tension were significantly higher in group NS, and blood recirculation fraction was significantly lower | NR | 30 |
| **307** | De Leyn et al[323] | 2011 | JTCVS | To evaluate the efficacy and safety of a synthetic bioresorbable pleural sealant to treat air leaks after pulmonary resection | Percentage of patients remaining air leak free until discharge | The overall success rates for intraoperative air leak sealing were as follows: sealant group, 71.0%; control group, 23.7% (P<.001). For grade 2 and 3 air leaks (n=77), the intraoperative sealing rates were as follows: sealant group, 71.7%; control group, 9.1% (P<.001). More patients with grade 2 and 3 air leaks had their leaks remain sealed in the sealant group (43.5%vs 15.2%, P=.013). The median time from skin closure to last observable air leak was 6 hours (sealant group) versus 42 hours (control group, P=.718) | 136 | 121 |
| **308** | Michaux et al[324] | 2011 | JTCVS | To determine if right ventricular global and overall systolic functions are better preserved 3 months after off-pump surgery than after conventional coronary bypass surgery | The echocardiographic indicators of RV global and overall systolic function | There were no significant intergroup differences in any echocardiographic marker of right ventricular function | NR | 50 |
| **309** | Speziale et al[325] | 2011 | JTCVS | To determine if the results of mitral repair for complex Barlow valves are adequate and support earlier intervention | No primary outcome excplicityly defined | Not reported | 618 | 140 |
| **310** | Benedetto et al[326] | 2012 | JTCVS | To assess if concomitant tricuspid valve annuloplasty in patients with tricuspid annulus dilatation (≥40 mm) prevents tricuspid regurgitation progression after mitral valve surgery | The occurrence of moderate to severe (≥3+) functional TR at 1-year follow-up | At 12 months follow-up, tricuspid regurgitation was absent in 71% (n=15) versus 19% (n=4) of patients in the treatment and control groups, respectively (P=.001) | NR | 44 |
| **311** | Chaudhuri et al[327] | 2012 | JTCVS | To analyze neurocognitive outcomes of patients after open-chamber cardiac surgery to determine whether carbon dioxide pericardial insufflation reduces incidence of neurocognitive decline | Incidence of neurocognitive decline as measured 6 weeks postoperatively | Neurocognitive testing showed no clinically significant differences in z scores between preoperative and postoperative testing | 146 | 125 |

© 2021 Robinson NB et al. *JAMA Network Open*.

| | | | | | | | | |
|---|---|---|---|---|---|---|---|---|
| | | | | (primary end point) as measured 6 weeks postoperatively and to assess the utility of carbon dioxide insufflation in cardiac chamber deairing as assessed by transesophageal echocardiography | | | | |
| **312** | On-off[328] | 2012 | JTCVS | To analyze the risk reduction of cardiopulmonary bypass complications between on-pump and off-pump coronary artery bypass grafting in high-risk patients | Composite of mortality and major complications comprising myocardial infarction (MI), neurologic complications, renal failure, adult respiratory distress syndrome (ARDS), and reoperation for bleeding, occurring within 30 days after surgery | According to the intention to treat analysis, the rate of the composite primary end point was significantly lower (unadjusted P=.009, adjusted P=.010) in the off-pump group (5.8% vs 13.3%). The risk of experiencing the primary end point was significantly greater for the on-pump group (unadjusted odds ratio, 2.51; 95% confidence interval, 1.23–5.10; P=.011; adjusted odds ratio, 3.07; 95% confidence interval, 1.32–7.14; P=.009). | NR | 411 |
| **313** | SAVE RITA[329] | 2012 | JTCVS | To compare early angiographic patency rates and clinical outcomes between the saphenous vein and right interval thoracic artery as a Y-composite graft | The 1-year angiographic patency rate of the distal anastomoses performed with a side-arm composite graft | Early angiography demonstrated an overall patency rate of 99.4% (771 of 776 distal anastomoses). Patency rates of the side-arm Y-composite graft (saphenous vein vs right internal thoracic artery) were 98.8% (245 of 248) and 99.5% (207 of 208) in the saphenous vein and right internal thoracic artery groups, respectively (P=.629) | 496 | 224 |
| **314** | Suri et al[330] | 2012 | JTCVS | To determine whether there are clinically important early differences among the Edwards Magna, Sorin Mitroflow, or St. Jude Epic bioprostheses | Early hemodynamic performance by echocardiography | Postoperative echocardiography showed small but statistically significant differences overall between the Magna, Mitroflow, and Epic valves in mean gradient (14.2 mm Hg, 16.3 mm Hg, 16.5 mm Hg, respectively; P=.011), aortic valve area (2.05 $cm^2$, 1.88 $cm^2$, 1.86 $cm^2$, respectively; P=.012), and indexed aortic valve area (1.05 $cm^2/m^2$, 0.97 $cm^2/m^2$, 0.95 $cm^2/m^2$, respectively; P=.012) | NR | 300 |
| **315** | STET[331] | 2013 | JTCVS | To compare off-pump coronary artery bypass surgery carried out via a left anterolateral thoracotomy (ThoraCAB) or via a conventional median sternotomy (OPCAB) | The time from surgery to fitness for hospital discharge as defined by objective criteria | The median time from surgery to fitness for discharge was 6 days (interquartile range, 4-7) in the ThoraCAB group versus 5 days (interquartile range, 4-7) in the OPCAB group (P=.53) | 465 | 93 |
| **316** | Bouchard et al[332] | 2014 | JTCVS | To assess the effect of treating FIMR with annuloplasty when mitral regurgitation is moderate | The LV dimension changes at 1 year | The left ventricular ejection fraction was significantly better at 3 months in the CABG alone group, although at 12 months, the left ventricular ejection fraction in the 2 groups had improved similarly | NR | 67 |

© 2021 Robinson NB et al. *JAMA Network Open*.

| | | | | | | | | |
|---|---|---|---|---|---|---|---|---|
| **317** | Svensson et al[333] | 2015 | JTCVS | To perform a randomized trial of brain protection during total aortic arch replacement and identify the best way to assess brain injury | Composite of (1) hospital death from neurologic causes, (2) postoperative clinical stroke, (3) brain imaging changes, and (4) reliable neurocognitive decline | The primary study composite neurologic end point, obtained by 10-fold multiple imputation of individual components, occurred in 22 of the 60 patients undergoing RBP and 15 of the 61 patients undergoing ABP (P=.2) | NR | 121 |
| **318** | Lee et al[334] | 2016 | JTCVS | To evaluate 3 surgical techniques for left atrial appendage exclusion with long-term follow up to define effectiveness | Success of closure (assessed in real time in multiple views after cessation of cardiopulmonary bypass by an experienced echocardiographer), including no remnant pouch >1 cm in maximum length after closure (stump) and the absence of a color flow jet between the left atrium (LA) and the LAA (gap) | In late follow-up, 1 of 7 patients in the IL group (14%) had a stump, compared with 2 of 8 (25%) in the StEx group and 3 of 6 (50%) in the SxEx group (P=.35). The overall failure rate was 57%: 5 of 8 (63%) in the IL group, 6 of 10 (60%) in the StEx group, and 5 of 10 (50%) in the SxEx group (P=.85). No patient had a stroke at any time during follow-up | 214 | 28 |
| **319** | Allen et al[335] | 2017 | JTCVS | To evaluate sternal healing, complications, and costs after sternotomy closure with rigid plate fixation or wire cerclage | Sternal healing based on CT evaluation by an independent core laboratory using a validated method at 6 months | Rigid plate fixation resulted in better sternal healing scores at 3 (2.6±1.1 vs 1.8±1.0; P<.0001) and 6 months (3.8±1.0 vs 3.3±1.1; P=.0007) and greater sternal union rates at 3 (41% (42/103) vs 16% (16/102); P<.0001) and 6 months (80% (81/101) vs 67% (67/100); P=.03) compared with wire cerclage | 461 | 236 |
| **320** | Halkos et al[336] | 2017 | JTCVS | To determine the impact of different aortic clamping strategies on the incidence of cerebral embolic events during coronary artery bypass grafting (CABG) | The number of HITS detected during TCD ultrasonography | In the off-pump group, the median number of total HITS were higher in the CFD subgroup (30.0; interquartile range (IQR), 22-43) compared with the partial clamp subgroup (7.0; IQR, 0-16; P<.0001). In the CFD subgroup, the median number of total HITS was significantly lower for patients with 1 CFD compared with patients with>1 CFD (12.5 (IQR, 4-19) vs 36.0 (IQR, 25-47); P=.001) | 1235 | 142 |
| **321** | Ad et al[337] | 2018 | JTCVS | To examine whether expanding del Nido cardioplegia to adult cardiac surgery confers benefits in surgical workflow and clinical outcome compared with blood-based cardioplegia | Myocardial preservation by return to spontaneous rhythm; defibrillation requirement; inotropes; and troponin levels at 4 time points: baseline (at anesthesia induction), 2 hours after termination of CPB, 12 hours after | There was no significant difference on CPB time (97 vs 103 minutes; P=.288) or crossclamp time (70 vs 83 minutes; P=.018). The del Nido group showed higher return to spontaneous rhythm (97.7% vs 81.6%; P=.023) and fewer patients required inotropic support (65.1% vs 84.2%; P=.050), but did not reach statistical significance. For del Nido group patients, troponin levels did not increase as much as for control patients | NR | 89 |

© 2021 Robinson NB et al. *JAMA Network Open*.

| | | | | | | | | |
|---|---|---|---|---|---|---|---|---|
| | | | | | admission to a cardiovascular intensive care unit (CVICU), and 24 hours after admission to a CVICU | (P=.040), but statistical significance was not reached | | |
| **322** | Marasco et al[338] | 2018 | JTCVS | To evaluate if biocompatible plastic cable ties would achieve a more rigid sternal fixation, reducing postoperative pain and analgesia requirements | Pain and analgesia requirements in the early postoperative period | There were no significant differences between groups in postoperative pain, analgesia, or early ventilator requirements. Patients in the ZIPFIX group had significantly more movement in the sternum and manubrium on ultrasound at 4 weeks | NR | 120 |
| **323** | Mini-Stern[339] | 2018 | JTCVS | To establish whether mini-sternotomy leads to quicker postoperative recovery and shorter hospital stay after first-time isolated AVR | Duration of postoperative hospital stay and the time to fitness for discharge from hospital after AVR | Compared with the FS group, the MS group had a longer hospital length of stay (mean, 9.5 days vs 8.6 days) and took longer to achieve fitness for discharge home (mean, 8.5 days vs 7.5 days) | 1024 | 222 |
| **324** | Wang et al[340] | 2018 | JTCVS | To evaluate the safety and efficacy of the addition of the cut-and-sew Maze III procedure (CSM) for mitral valve replacement (MVR) in patients with atrial fibrillation (AF) associated with rheumatic mitral valve disease (RMVD) | Composite of freedom from stroke and death at 1 year | One-year freedom from stroke or death was better in the Maze III group compared with the non-Maze group (P=.0028; hazard ratio, 0.2653; 95% confidence interval, 0.1122 to 0.6270). The risk of AF recurrence in the Maze III group was 0.002-fold that in non-Maze group (P=.000) | 180 | 130 |
| **325** | Braathen et al[341] | 2019 | JTCVS | To compare hemodynamic profiles of the Trifecta to our standard Mosaic Ultra biological valve | Mean and maximum gradients and EOAI measured 6 months postoperatively with transthoracic echocardiography | There were lower transvalvular gradients in the Trifecta compared with the Mosaic Ultra group for the given annulus sizes | NR | 90 |
| **326** | CANON[342] | 2019 | JTCVS | To determine whether using any of the 2 selected modifications of OPCAB could decrease the incidence of neuropsychiatric complications | Incidence of postoperative delirium (PD) and early postoperative cognitive dysfunction (ePOCD) | The incidence of PD was 35.9% in the control (OPCAB) arm, 32.8% in the CO2FF arm, and 12.5%in the ANA arm ($x^2$ [2, N=191] = 10.17; P=.006). Post hoc tests revealed that the incidence of PD in the ANA arm differed from thatcin the OPCAB arm (odds ratio [OR], 0.26; 95% confidence interval [CI], 0.09-0.68; P=.002). The incidence of ePOCD was 34.4% in the OPCAB arm, 28.1% in the CO2FF arm, and 9.5% in the ANA arm ($x^2$ [2, N=191] = 11.58; P=.003) | 269 | 192 |
| **327** | Jiwnani et al[343] | 2019 | JTCVS | To evaluate if a modified technique of posterolateral thoracotomy and closure, preserving the intercostal neurovascular bundle, would reduce acute | Worst postoperative pain score in the first 3 postoperative days | There was no difference seen in the worst (mean) postoperative pain scores (3.71 vs 3.83, difference 0.12; 99% confidence interval [CI], -0.7 to +0.9; P=0.7) | 340 | 90 |

© 2021 Robinson NB et al. *JAMA Network Open*.

| | | | | | | | | |
|---|---|---|---|---|---|---|---|---|
| | | | | and chronic post-thoracotomy pain | | | | |
| **328** | Suzuki et al[344] | 2019 | JTCVS | To confirm the noninferiority of segmentectomy to lobectomy in regard to prognosis | Overall survival | No mortality was noted | 1319 | 1106 |
| **329** | JCOG9501[345] | 2008 | NEJM | To compare D2 lymphadenectomy alone with D2 lymphadenectomy plus PAND in patients undergoing gastrectomy for curable gastric cancer | Overall survival | The 5-year overall survival rate was 69.2% for the group assigned to D2 lymphadenectomy alone and 70.3% for the group assigned to D2 lymphadenectomy plus PAND; the hazard ratio for death was 1.03 (95% confidence interval (CI), 0.77 to 1.37; P = 0.85) | NR | 523 |
| **330** | HeartMate II[346] | 2009 | NEJM | To determine the efficacy of a new continuous-flow left ventricular assist device with a pulsatile device | Composite of survival free from disabling stroke and reoperation to repair or replace the device at 2 years | The primary composite end point was achieved in more patients with continuous-flow devices than with pulsatile-flow devices (62 of 134 (46%) vs. 7 of 66 (11%); P<0.001; hazard ratio, 0.38; 95% confidence interval, 0.27 to 0.54; P<0.001), and patients with continuous- flow devices had superior actuarial survival rates at 2 years (58% vs. 24%, P = 0.008) | NR | 200 |
| **331** | ROOBY[347] | 2009 | NEJM | To determine if coronary artery bypass grafting without cardiopulmonary bypass (off-pump CABG) reduces the number of complications related to the heart–lung machine | Composite of death from any cause, a repeat revascularization procedure, or a nonfatal myocardial infarction within 1 year after surgery | There was no significant difference between off-pump and on-pump CABG in the rate of the 30-day composite outcome (7.0% and 5.6%, respectively; P = 0.19). The rate of the 1-year composite outcome was higher for off-pump than for on-pump CABG (9.9% vs. 7.4%, P = 0.04) | 9663 | 2203 |
| **332** | STICH[348] | 2009 | NEJM | To address whether surgical ventricular reconstruction added to coronary-artery bypass grafting (CABG) would decrease the rate of death or hospitalization for cardiac causes, as compared with CABG alone | Composite of death from any cause and hospitalization for cardiac causes | No significant difference was observed in the primary outcome, which occurred in 292 patients (59%) who were assigned to undergo CABG alone and in 289 patients (58%) who were assigned to undergo CABG with surgical ventricular reconstruction (hazard ratio for the combined approach, 0.99; 95% confidence interval, 0.84 to 1.17; P = 0.90) | NR | 1000 |
| **333** | NTMG[349] | 2011 | NEJM | To compare the use of a trocar-guided, transvaginal polypropylene-mesh repair kit with traditional colporrhaphy in women with prolapse of the anterior vaginal wall (cystocele) | A composite of the objective anatomical designation of stage 0 (no prolapse) or 1 (position of the anterior vaginal wall more than 1 cm above the hymen), according to the Pelvic Organ Prolapse Quantification system, and the subjective absence of symptoms of | At 1 year, the primary outcome was significantly more common in the women treated with transvaginal mesh repair (60.8%) than in those who underwent colporrhaphy (34.5%) (absolute difference, 26.3 percentage points; 95% confidence interval, 15.6 to 37.0) | 1685 | 389 |

© 2021 Robinson NB et al. *JAMA Network Open*.

| | | | | | | | | |
|---|---|---|---|---|---|---|---|---|
| | | | | | vaginal bulging 12 months after the surgery | | | |
| **334** | CORONARY[350] | 2012 | NEJM | To establish the benefits and risk of performing coronary-artery bypass grafting (CABG) with a beating-heart technique (off-pump CABG), as compared with cardiopulmonary bypass (on-pump CABG) | The first coprimary outcome was a composite of death, nonfatal stroke, nonfatal myocardial infarction, or new renal failure requiring dialysis at 30 days after randomization. The second coprimary outcome was the first coprimary outcome plus repeat coronary revascularization at a mean of 5 years | There was no significant difference in the rate of the primary composite outcome between off-pump and on-pump CABG (9.8% vs. 10.3%; hazard ratio for the off pump group, 0.95; 95% confidence interval (CI), 0.79 to 1.14; P = 0.59) or in any of its individual components | NR | 4752 |
| **335** | FIDELITY[351] | 2013 | NEJM | To determine the efficacy of arthroscopic partial meniscectomy on patient outcomes | Changes in the Lysholm and Western Ontario Meniscal Evaluation Tool (WOMET) scores (each ranging from 0 to 100, with lower scores indicating more severe symptoms) and in knee pain after exercise (rated on a scale from 0 to 10, with 0 denoting no pain) at 12 months after the procedure | There were no significant between-group differences in the change from baseline to 12 months in any primary outcome | NR | 146 |
| **336** | GOPCABE[352] | 2013 | NEJM | To investigate the benefits of coronary artery bypass grafting without cardiopulmonary bypass in elderly patients | Composite of death, stroke, myocardial infarction, repeat revascularization, or new renal-replacement therapy at 30 days and at 12 months after surgery | At 30 days after surgery, there was no significant difference between patients who underwent off-pump surgery and those who underwent on-pump surgery in terms of the composite outcome (7.8% vs. 8.2%; odds ratio, 0.95; 95% confidence interval (CI), 0.71 to 1.28; P = 0.74) or four of the components (death, stroke, myocardial infarction, or new renal replacement therapy) | 4355 | 2403 |
| **337** | Acker et al[353] | 2014 | NEJM | To evaluate the efficacy and safety of mitral valve repair versus chordal sparing replacement for the treatment of ischemic mitral regurgitation | Left ventricular end-systolic volume index (LVESVI) at 12 months | At 12 months, the mean LVESVI among surviving patients was 54.6±25.0 ml per square meter of body-surface area in the repair group and 60.7±31.5 ml per square meter in the replacement group (mean change from baseline, −6.6 and −6.8 ml per square meter, respectively). The rate of death was 14.3% in the repair group and 17.6% in the replacement group (hazard ratio with repair, 0.79; 95% confidence interval, 0.42 to 1.47; P = 0.45 by the log-rank test). There was no significant | 3458 | 251 |

© 2021 Robinson NB et al. *JAMA Network Open*.

| | | | | | | | | |
|---|---|---|---|---|---|---|---|---|
| | | | | | | between-group difference in LVESVI after adjustment for death (z score, 1.33; P = 0.18) | | |
| **338** | Smith et al[354] | 2014 | NEJM | To determine the benefits of adding mitral valve repair to coronary artery bypass in patients with ischemic mitral regurgitation | Left ventricular end-systolic volume index (LVESVI), a measure of left ventricular remodeling, at 1 year | The mean LVESVI among surviving patients was 46.1±22.4 ml per square meter of body-surface area in the CABG-alone group and 49.6±31.5 ml per square meter in the combined-procedure group (mean change from baseline, −9.4 and −9.3 ml per square meter, respectively) | 725 | 301 |
| **339** | Chagpar et al[355] | 2015 | NEJM | To assess whether routine resection of cavity shave margins reduce the rate of positive margins and reecision among patients undergoing partial mastectomy for breast cancer | The rate of positive margins | After randomization, patients in the shave group had a significantly lower rate of positive margins than did those in the no-shave group (19% vs. 34%, P = 0.01), as well as a lower rate of second surgery (10% vs. 21%, P = 0.02) | NR | 235 |
| **340** | COLOR II[356] | 2015 | NEJM | To compare 3-year rates of cancer recurrence in the pelvic or perineal area (locoregional recurrence) and survival after laparoscopic and open resection of rectal cancer | Locoregional recurrence 3 years after the index surgery | At 3 years, the locoregional recurrence rate was 5.0% in the two groups (difference, 0 percentage points; 90% confidence interval (CI), −2.6 to 2.6) | NR | 1044 |
| **341** | D'Cruz et al[357] | 2015 | NEJM | To evaluate the effect on survival of elective node dissection ipsilateral neck dissection at the time of the primary surgery versus therapeutic node dissection (watchful waiting followed by neck dissection for nodal relapse) in patients with lateralized stage T1 or T2 oral squamous cell carcinomas | Overall survival, which was defined as the interval between the date of randomization and the date of death from any cause | At 3 years, elective node dissection resulted in an improved rate of overall survival (80.0%; 95% confidence interval (CI), 74.1 to 85.8), as compared with therapeutic dissection (67.5%; 95% CI, 61.0 to 73.9), for a hazard ratio for death of 0.64 in the elective-surgery group (95% CI, 0.45 to 0.92; P = 0.01 by the log-rank test) | 1281 | 596 |
| **342** | Gillinov et al[358] | 2015 | NEJM | To evaluate the safety and effectiveness of surgical ablation of atrial fibrillation in patients undergoing mitral valve surgery | Freedom from atrial fibrillation at both 6 months and 12 months (as assessed by means of 3-day Holter monitoring) | There was no significant difference in the rate of freedom from atrial fibrillation between patients who underwent pulmonary-vein isolation and those who underwent the biatrial maze procedure (61.0% and 66.0%, respectively; P = 0.60) | 3502 | 260 |
| **343** | SSSS[359] | 2016 | NEJM | To investigate the efficacy of fusion surgery in addition to decompression surgery in patients who have lumbar spinal stenosis, with or without degenerative spondylolisthesis | Score on the Oswestry Disability Index (ODI; which ranges from 0 to 100, with higher scores indicating more severe disability) 2 years after surgery | There was no significant difference between the groups in the mean score on the ODI at 2 years (27 in the fusion group and 24 in the decompression-alone group, P = 0.24) or in the results of the 6-minute walk test (397 m in the fusion group and 405 m in the decompression-alone group, P = 0.72) | 358 | 247 |
| **344** | ENDURANCE[360] | 2017 | NEJM | To compare a newer LVAD design (a small intrapericardial | Survival at 2 years free from disabling stroke or | The primary end point was achieved in 164 patients in the study group and 85 patients in the | 559 | 445 |

© 2021 Robinson NB et al. *JAMA Network Open*.

| | | | | | | | | |
|---|---|---|---|---|---|---|---|---|
| | | | | centrifugal-flow device) against existing technology (a commercially available axial-flow device) in patients with advanced heart failure who were ineligible for heart transplantation | device removal for malfunction or failure | control group. The analysis of the primary end point showed noninferiority of the study device relative to the control device (estimated success rates, 55.4% and 59.1%, respectively, calculated by the Weibull model; absolute difference, 3.7 percentage points;95% upper confidence limit, 12.56 percentage points; P = 0.01 for noninferiority). | | |
| **345** | MOMENTUM 3[361] | 2017 | NEJM | To investigate the effects of a new magnetically levitated centrifugal continuous-flow pump that was engineered to avert thrombosis | Composite of survival free of disabling stroke (with disabling stroke indicated by a modified Rankin score >3; scores range from 0 to 6, with higher scores indicating more severe disability) or survival free of reoperation to replace or remove the device at 6 months after implantation | The primary end point occurred in 131 patients (86.2%) in the centrifugal-flow pump group and in 109 (76.8%) in the axial-flow pump group (absolute difference, 9.4 percentage points; 95% lower confidence boundary, −2.1 P<0.001 for noninferiority; hazard ratio, 0.55; 95% confidence interval (CI), 0.32 to 0.95 (two-tailed P = 0.04 for superiority)) | NR | 294 |
| **346** | LACC[362] | 2018 | NEJM | To determine if survival outcomes after laparoscopic or robot-assisted radical hysterectomy (minimally invasive surgery) are equivalent to those after open abdominal radical hysterectomy (open surgery) among women with early-stage cervical cancer | Rate of disease-free survival at 4.5 years | The rate of disease-free survival at 4.5 years was 86.0% with minimally invasive surgery and 96.5% with open surgery, a difference of −10.6 percentage points (95% confidence interval (CI), −16.4 to −4.7) | NR | 631 |
| **347** | FREGAT[363] | 2019 | NEJM | To determine whether hybrid minimally invasive esophagectomy results in lower morbidity than open esophagectomy | Intraoperative or postoperative complication of grade II or higher according to the Clavien–Dindo classification (indicating major complication leading to intervention) within 30 days | A total of 37 patients (36%) in the hybrid procedure group had a major intraoperative or postoperative complication, as compared with 67 (64%) in the open-procedure group (odds ratio, 0.31; 95% confidence interval [CI], 0.18 to 0.55; P<0.001) | 219 | 207 |
| **348** | HEALTH[364] | 2019 | NEJM | To compare total hip arthroplasty with hemiarthroplasty | Secondary hip procedure within 24 months of follow-up | The primary end point occurred in 57 of 718 patients (7.9%) who were randomly assigned to total hip arthroplasty and 60 of 723 patients (8.3%) who were randomly assigned to hemiarthroplasty (hazard ratio, 0.95; 95% confidence interval [CI], 0.64 to 1.40; P = 0.79). | NR | 1495 |

© 2021 Robinson NB et al. *JAMA Network Open*.

| | | | | | | | | |
|---|---|---|---|---|---|---|---|---|
| **349** | LION[365] | 2019 | NEJM | To investigate systematic pelvic and paraaortic lymphadenectomy in the surgical treatment of patients with advanced ovarian cancer | Overall survival | The median overall survival was 69.2 months in the no-lymphadenectomy group and 65.5 months in the lymphadenectomy group (hazard ratio for death in the lymphadenectomy group, 1.06; 95% confidence interval [CI], 0.83 to 1.34; P = 0.65) | 1895 | 650 |
| **350** | Ryang et al[366] | 2008 | Neurosurgery | To compare efficiency, safety, and outcome of standard open microsurgical discectomy (SOMD) for lumbar disc herniation with microsurgical discectomy using an 11.5 mm trocar system for minimal access to the spine | Perioperative parameters and pre- and postoperative clinical findings including sensory or motor deficits and pain according to the visual analog scale, Oswestry Disability Index scores, and Short Form-36 results | Preoperatively, no statistically significant intergroup differences could be detected proving the comparability of both groups. Postoperatively, significant improvement of neurological symptoms and pain as measured by the visual analog scale, Oswestry Disability Index, and Short Form-36 scores could be achieved in both groups | NR | 60 |
| **351** | Rosen et al[367] | 2011 | Neurosurgery | To report 6-month results of a randomized, controlled trial of a biosynthesized cellulose (BSC) composed duraplasty device compared with commercially available dural replacements | The absence of pseudomeningocele and extracerebral fluid collection confirmed radiographically and the absence of cerebrospinal fluid fistula at 6 months | At 6 months, the primary hypothesis, noninferiority of the BSC implant compared with the control group, was confirmed (P = .0206) | NR | 99 |
| **352** | van den Akker et al[368] | 2011 | Neurosurgery | To determine whether a favorable cost-effectiveness for tubular discectomy compared with conventional microdiskectomy is attained | Quality-adjusted life-years at 1 year and societal costs, estimated from patient reported utilities (US and Netherlands EuroQol, Short Form Health Survey-6D, and Visual Analog Scale) and diaries on costs (health care, patient costs, and productivity) | Quality-adjusted life-years during all 4 quarters and according to all utility measures were not statistically different between tubular diskectomy and conventional microdiskectomy (difference for US EuroQol, -0.012; 95% confidence interval, -0.046 to 0.021). From the healthcare perspective, tubular diskectomy resulted in nonsignificantly higher costs (difference US $460; 95% confidence interval, -243 to 1163). From the societal perspective, a nonsignificant difference of US $1491 (95% confidence interval, -1335 to 4318) in favor of conventional microdiskectomy was found | NR | 325 |
| **353** | Villavicencio et al[369] | 2011 | Neurosurgery | To quantitatively assess and compare cervical sagittal alignment and clinical outcome when lordotic or parallel allografts were used for fusion | Clinical outcome scores between the lordotic and parallel graft patient groups | There were no statistically significant differences in clinical outcome scores between the lordotic and parallel graft patient groups | NR | 122 |
| **354** | Manzano et al[370] | 2012 | Neurosurgery | To determine clinical, radiological, and patient satisfaction outcomes between expansile cervical laminoplasty (ECL) and cervical laminectomy and fusion (CLF) | Short Form-36, Neck Disability Index, Visual Analog Scale, modified Japanese Orthopedic Association score, Nurick score, and radiographic measures at 3 months and at 1-year follow-up | Both groups showed improvements in their Nurick grade and Japanese Orthopedic Association score postoperatively, but only the improvement in the Nurick grade for the ECL group was statistically significant (P<.05). The cervical range of motion between C2 and C7 was reduced by 75% in the CLF group and by only 20% in the ECL group in a comparison of preoperative and postoperative | NR | 16 |

© 2021 Robinson NB et al. *JAMA Network Open*.

| | | | | | | | | |
|---|---|---|---|---|---|---|---|---|
| | | | | | | range of motion. The overall increase in canal area was significantly (P<.001) greater in the CLF group, but there was a suggestion that the adjacent level was more narrowed in the CLF group in as little as 1 year postoperatively | | |
| **355** | Schmidt et al[371] | 2015 | Neurosurgery | To compare the long- and short-term results of endoscopic and open decompression in cubital tunnel syndrome | The severity of symptoms was measured by McGowan and Dellon Score, and the clinical outcome by modified Bishop Score | No differences were found regarding clinical or neurophysiological outcome in both early and late follow-up between both groups | 56 | 54 |
| **356** | George et al[372] | 2017 | Neurosurgery | To compare the efficacy and safety of adjunctive TachoSil (Takeda Pharma A/S, Roskilde, Denmark) with current practice for the prevention of postoperative CSF leaks in patients undergoing elective skull base surgery involving dura mater closure | Occurrence of clinically evident verified postoperative CSF leak or clinically evident pseudomeningocele within 7 weeks after surgery or treatment failure (third application of trial treatment or use of other treatment) | The primary endpoint of estimated leak rate favored TachoSil with events in 25 (6.9%) patients vs 30 (8.2%) current practice patients; however, this was not statistically significant (odds ratio: 0.82; 95% confidence interval: 0.47, 1.43; P = .485) | 778 | 726 |
| **357** | Choi et al[373] | 2018 | Neurosurgery | To compare the radiological and clinical outcomes of unilateral minimally invasive transforaminal lumbar interbody fusion (MIS-TLIF) using 2 types of cage | Increase of DH (disk height) postoperatively, at 6 and 12 months | The change in disc height and segmental lordotic angle postoperatively was significantly greater in the banana-shaped cage group | NR | 90 |
| **358** | NICE[374] | 2018 | Neurosurgery | To show that a minimally invasive percutaneous IPD is safe and noninferior to standalone decompressive surgery (SDS) for patients with degenerative lumbar spinal stenosis with NIC | The mean percentage change in physical function from baseline to 1-yr follow-up, obtained by the Zurich Claudication Questionnaire (ZCQ) | There was significant improvement in Zurich Claudication Questionnaire physical function, as mean percentage change from baseline, for both the IPD and the SDS groups at 12 mo (primary endpoint) and 24 mo (−32.3 ± 32.1, −37.5 ± 22.8; and −37.9 ± 21.7%, −35.2 ± 22.8, both P < .001). IPD treatment was not significantly noninferior (margin: 10%) to SDS treatment at 12mo (P=.172) but was significantly noninferior at 24mo (P = .005) | 215 | 163 |
| **359** | cSDH-Drain-Trial[375] | 2019 | Neurosurgery | To investigate whether the recurrence rate after insertion of a SPD is noninferior to the insertion of a more commonly used SDD | Recurrence indicating a reoperation within 12 months | Recurrence rate was lower in the SPD group (8.33%, 95% confidence interval [CI] 4.28-14.72) than in the SDD group (12.00%, 95% CI 6.66-19.73), with the treatment difference (3.67%, 95% CI -12.6-5.3) not meeting predefined noninferiority criteria | 262 | 220 |
| **360** | MRC ASTEC[376] | 2009 | The Lancet | To investigate whether pelvic lymphadenectomy could improve survival of women with endometrial cancer | Overall survival | After a median follow-up of 37 months (IQR 24–58), 191 women (88 standard surgery group, 103 lymphadenectomy group) had died, with a hazard ratio (HR) of 1.16 (95% CI 0.87–1.54; p=0.31) in | NR | 1408 |

© 2021 Robinson NB et al. *JAMA Network Open*.

| | | | | | | | | |
|---|---|---|---|---|---|---|---|---|
| | | | | | | favour of standard surgery and an absolute difference in 5-year overall survival of 1% (95% CI –4 to 6) | | |
| **361** | Santarius et al[377] | 2009 | The Lancet | To investigate the effect of drains on recurrence rates of subdural haematoma and clinical outcomes | Recurrence rate defined as the rate of reoperation to treat recurrent chronic subdural haematoma in patients previously treated with burr-hole evacuation with and without drain | Recurrence occurred in ten of 108 (9.3%) people with a drain, and 26 of 107 (24%) without (p=0.003; 95% CI 0.14–0.70). At 6 months mortality was nine of 105 (8.6%) and 19 of 105 (18.1%), respectively (p=0.042; 95% CI 0.1–0.99). Medical and surgical complications were much the same between the study groups | 269 | 215 |
| **362** | El-Hamamsy et al[378] | 2010 | The Lancet | To compare long term outcomes after autograft aortic root replacement (Ross procedure) versus homograft aortic root replacement in adults | Overall survival at 10 years | At 10 years, four patients died in the autograft group versus 15 in the homograft group. Actuarial survival at 10 years was 97% (SD 2) in the autograft group versus 83% (4) in the homograft group. Hazard ratio for death in the homograft group was 4.61 (95% CI 1.71–16.03; p=0.0060) | NR | 216 |
| **363** | DISPACT[379] | 2011 | The Lancet | To investigate if standardized closure with a stapler device would prevent pancreatic fistula more effectively than would a hand-sewn closure of the remnant | Composite of the development of a postoperative pancreatic fistula, death due to any cause, or both | Pancreatic fistula rate or mortality did not differ between stapler (56 (32%) of 177) and hand-sewn closure (49 (28%) of 175; OR 0.84,95% CI 0.53–1.33; p=0.56) | NR | 352 |
| **364** | Gillgren et al[380] | 2011 | The Lancet | To test whether survival was different for a wide local excision margin of 2 cm compared with a 4-cm excision margin | Overall survival | After a median follow-up of 6.7 years (IQR 4.3–9.5) 181 patients in the 2-cm margin group and 177 in the 4-cm group had died (hazard ratio 1.05, 95% CI 0.85–1.29; p=0.64). 5-year overall survival was 65% (95% CI 60–69) in the 2-cm group and 65% (60–70) in the 4-cm group (p=0.69) | NR | 936 |
| **365** | Biere et al[381] | 2012 | The Lancet | To assess whether minimally invasive oesophagectomy reduces morbidity compared with open esophagectomy | Pulmonary infection within the first 2 weeks after surgery and during the whole stay in hospital | 16 (29%) patients in the open oesophagectomy group had pulmonary infection in the first 2 weeks compared with five (9%) in the minimally invasive group (relative risk (RR) 0.30, 95% CI 0.12–0.76; p=0.005). 19 (34%) patients in the open oesophagectomy group had pulmonary infection in-hospital compared with seven (12%) in the minimally invasive group (0.35, 0.16–0.78; p=0.005) | 144 | 115 |
| **366** | CORONIS[382] | 2013 | The Lancet | To assess whether any cesarean section surgical techniques were associated with improved outcomes for women and babies | Composite of death, maternal infectious morbidity, further operative procedures, or blood transfusion (>1 unit) up to the 6-week follow-up visit | There were no statistically significant differences within any of the intervention pairs for the primary outcome: blunt versus sharp entry risk ratio 1.03 (95% CI 0.91–1.17), exterior versus intra-abdominal repair 0.96 (0.84–1.08), single-layer versus double-layer closure 0.96 (0.85–1.08), closure versus non-closure 1.06 (0.94–1.20), and | NR | 15935 |

© 2021 Robinson NB et al. *JAMA Network Open*.

| | | | | | | | | |
|---|---|---|---|---|---|---|---|---|
| | | | | | | chromic catgut versus polyglactin-910 0.90 (0.78–1.04). | | |
| **367** | Kolle et al[383] | 2013 | The Lancet | To compare the survival of fat grafts enriched with autologous adipose derived stem cells (ASCs) versus non-enriched fat grafts | Residual graft volumes of ASC-enriched grafts with those of control grafts | Compared with the control grafts, the ASC enriched fat grafts had significantly higher residual volumes: 23.00 (95% CI 20.57–25.43) cm³ versus 4.66 (3.16–6.16) cm³ for the controls, corresponding to 80.9% (76.6–85.2) versus 16.3% (11.1–21.4) of the initial volumes, respectively (p<0.0001) | NR | 13 |
| **368** | Tsujinaka et al[384] | 2013 | The Lancet | To assess the differences in the frequency of wound complications, including superficial incisional surgical site infection and hypertrophic scar formation, depending on whether subcuticular sutures or staples are used | Incidence of wound complications within 30 days of surgery | Overall, the rate of wound complications did not differ significantly between the subcuticular sutures and staples groups (odds ratio 0.709, 95% CI 0.474–1.062; p=0.12) | NR | 1080 |
| **369** | ESEP[385] | 2014 | The Lancet | To assess whether salpingotomy would improve rates of ongoing pregnancy by natural conception compared with salpingectomy | Ongoing pregnancy by natural conception | The cumulative ongoing pregnancy rate was 60.7% after salpingotomy and 56.2% after salpingectomy (fecundity rate ratio 1.06, 95% CI 0.81–1.38; log-rank p=0.678) | 450 | 446 |
| **370** | MesoVATS[386] | 2014 | The Lancet | To compare efficacy in terms of overall survival, and cost, of VAT-PP and talc pleurodesis in patients with malignant pleural mesothelioma | Overall survival at 1 year | Overall survival at 1 year was 52% (95% CI 41–62) in the VAT-PP group and 57% (46–66) in the talc pleurodesis group (hazard ratio 1.04 (95% CI 0.76–1.42); p=0.81) | NR | 175 |
| **371** | Slaghekke et al[387] | 2014 | The Lancet | To assess the efficacy and safety of a novel surgery technique that uses laser coagulation of the entire vascular equator (Solomon technique) | Composite of incidence of twin anemia polycythemia sequence, recurrence of twin-to-twin transfusion syndrome, perinatal mortality, or severe neonatal morbidity | The primary outcome occurred in 94 (34%) of 274 fetuses in the Solomon group versus 133 (49%) of 270 in the standard treatment group (OR 0.54; 95% CI 0.35–0.82) | NR | 544 |
| **372** | Ladies trial (LOLA group)[388] | 2015 | The Lancet | To assess the superiority of laparoscopic lavage compared with sigmoidectomy in patients with purulent perforated diverticulitis, with respect to overall long-term morbidity and mortality | Composite endpoint including major morbidity and mortality within 12 months | The primary endpoint occurred in 30 (67%) of 45 patients in the lavage group and 25 (60%) of 42 patients in the sigmoidectomy group (odds ratio 1.28, 95% CI 0.54–3.03, p=0.58) | 563 | 90 |
| **373** | PONCHO[389] | 2015 | The Lancet | To compare same-admission and interval cholecystectomy, with the hypothesis that same-admission cholecystectomy would reduce the risk of | Composite of readmission for recurrent gallstone-related complications (pancreatitis, cholangitis, | The primary endpoint occurred in 23 (17%) of 136 patients in the interval group and in six (5%) of 128 patients in the same-admission group (risk ratio 0.28, 95% CI 0.12–0.66; p=0.002) | 713 | 266 |

© 2021 Robinson NB et al. *JAMA Network Open*.

| | | | | | | | | |
|---|---|---|---|---|---|---|---|---|
| | | | | recurrent gallstone-related complications without increasing the difficulty of surgery | cholecystitis, choledocholithiasis needing endoscopic intervention, or gallstone colic) or mortality within 6 months after randomization | | | |
| **374** | STITCH[390] | 2015 | The Lancet | To compare the large bites suture technique with the small bites technique for fascial closure of midline laparotomy incisions | Incisional hernia occurrence at 1 year | At 1-year follow-up, 57 (21%) of 277 patients in the large bites group and 35 (13%) of 268 patients in the small bites group had incisional hernia (p=0.0220, covariate adjusted odds ratio 0.52, 95% CI 0.31–0.87; p=0.0131) | 609 | 560 |
| **375** | eTHoS[391] | 2016 | The Lancet | To establish the clinical effectiveness and cost-effectiveness of stapled haemorrhoidopexy compared with traditional excisional surgery | The area under the quality of life curve (AUC) over 24 months derived from EQ-5D-3L measurements taken from patient questionnaires distributed at baseline, 1 week, 3 weeks, 6 weeks (postoperative), 12 months, and 24 months post-randomization. The primary trial economic outcome was incremental costs per QALY gained with QALYs based on the responses to the EQ-5D-3L over 24 months. | The EQ-5D-3L AUC score was higher in the traditional excisional surgery group than the stapled haemorrhoidopexy group over 24 months; mean difference –0.073 (95% CI –0.140 to –0.006; p=0.0342). | 1127 | 777 |
| **376** | HubBLe[392] | 2016 | The Lancet | To compare recurrence after hemorrhoid artery ligation (HAL) versus rubber band ligation (RBL) in patients with grade II–III hemorrhoids | Recurrent hemorrhoids at 12 months | At 1-year post-procedure, 87 (49%) of 176 patients in the RBL group and 48 (30%) of 161 patients in the HAL group had hemorrhoid recurrence (adjusted odds ratio (aOR) 2.23, 95% CI 1.42–3.51; p=0.0005). | 969 | 372 |
| **377** | Stenberg et al[393] | 2016 | The Lancet | To compare rates of small bowel obstruction due to internal hernia following laparoscopic gastric bypass in patients with closed versus non-closed mesenteric defects | Surgery for small bowel obstruction within 3 years (efficacy endpoint) and severe complications within 30 days after surgery (safety endpoint) | At 3 years after surgery, the cumulative incidence of reoperation because of small bowel obstruction was significantly reduced in the closure group (cumulative probability 0.055 for closure vs 0.102 for non-closure, hazard ratio 0.56, 95% CI 0.41–0.76, p=0.0002. | 2587 | 2507 |
| **378** | Yaxley et al[394] | 2016 | The Lancet | To compare laparoscopic and open radial prostatectomy in terms of functional and oncological outcomes and report the early | Urinary function (urinary domain of EPIC) and sexual function (sexual domain of EPIC and IIEF) at 6 weeks, 12 weeks, | At 12 weeks, urinary and sexual function scores did not differ significantly. Equivalence testing showed quality between the two techniques based on a 90% CI and a delta of 10%. | 334 | 326 |

© 2021 Robinson NB et al. *JAMA Network Open*.

| | | | | | | | | |
|---|---|---|---|---|---|---|---|---|
| | | | | postoperative outcomes at 12 weeks | and 24 months and oncological outcome (positive surgical margin status and biochemical and imaging evidence of progression at 24 months) | | | |
| **379** | ChroPac[395] | 2017 | The Lancet | To investigate the long-term outcomes of patients with chronic pancreatitis within 24 months after surgery treated with duodenum preserving pancreatic head resection versus partial pancreatoduodenectomy | Mean quality of life at 24 months following surgery | No difference in quality of life was seen between the groups within 24 months after surgery (75.3 (SD 16.4) for partial pancreatoduodenectomy vs 73.0 (16.4) for DPPHR; mean difference –2.3, 95% CI –6.6 to 2.0; p=0.284). | NR | 250 |
| **380** | FAITH[396] | 2017 | The Lancet | To investigate the effect of a sliding hip screw versus cancellous screws on the risk of reoperation and other key outcomes | Hip reoperation within 24 months after initial surgery to promote fracture healing, relieve pain, treat infection, or improve function | Reoperations within 24 months did not differ by type of surgical fixation in those included in the primary analysis: 107 (20%) of 542 patients in the sliding hip screw group versus 117 (22%) of 537 patients in the cancellous screws group (hazard ratio (HR) 0.83, 95% CI 0.63–1.09; p=0.18) | 7306 | 1108 |
| **381** | GRECCAR 2[397] | 2017 | The Lancet | To compare local excision and total mesorectal excision in patients with a good response after chemoradiotherapy for lower rectal cancer | Composite outcome with four components: death, recurrence (local or distal), major surgical morbidity (grades III–V of Dindo's classification), and severe complications (definitive colostomy, anal incontinence, or impotence) at 2 years after randomization | At 2 years in the modified ITT population, one or more events from the composite primary outcome occurred in 41 (56%) of 73 patients in the local excision group and 33 (48%) of 69 in the total mesorectal excision group (odds ratio 1.33, 95% CI 0.62–2.86; p=0.43) | NR | 148 |
| **382** | PROSPECT (Graft trial)[398] | 2017 | The Lancet | To compare the outcomes of prolapse repair involving either synthetic mesh inlays or biological grafts against standard repair in women | Participant-reported prolapse symptoms (i.e. the Pelvic Organ Prolapse Symptom Score (POP-SS)) and condition-specific (i.e. prolapse-related) quality-of-life scores | Mean POP-SS at 1 year did not differ substantially between comparisons (standard 5.5 (SD 5.6) vs graft 5.6 (5.6); mean difference –0.15, –0.93 to 0.63; p=0.71). Mean prolapse-related quality-of-life scores also did not differ between groups at 1 year (standard 2.2 (SD 2.8) vs graft 2.4 (2.9); mean diff erence 0.13, –0.30 to 0.56; p=0.54) | 4083 | 735 |
| **383** | PROSPECT (Mesh trial)[398] | 2017 | The Lancet | To compare the outcomes of prolapse repair involving either synthetic mesh inlays or biological grafts against standard repair in women | Participant-reported prolapse symptoms (i.e. the Pelvic Organ Prolapse Symptom Score (POP-SS)) and condition-specific (i.e. prolapse-related) quality-of-life scores | Mean POP-SS at 1 year did not differ substantially between comparisons (standard 5.4 (SD 5.5) vs mesh 5.5 (5.1), mean difference 0.00, 95% CI –0.70 to 0.71; p=0.99). Mean prolapse-related quality-of-life scores also did not differ between groups at 1 year (standard 2.0 (SD 2.7) vs mesh 2.2 (2.7), mean diff erence 0.13, 95% CI–0.25 to 0.51; p=0.50) | 4083 | 865 |

© 2021 Robinson NB et al. *JAMA Network Open*.

| | | | | | | | | |
|---|---|---|---|---|---|---|---|---|
| **384** | HUMP[399] | 2018 | The Lancet | To determine if mesh is superior to primary closure of small (1-4 cm) umbilical hernias | Hernia recurrence rate at 24 months | Mesh was associated with fewer recurrence when compared with primary repair. 2-year actuarial estimates of recurrence 3.6% (95% CI 1.4–9.4) vs 11.4% (6.8–18.9); p=0.01, hazard ratio 0.31, 95% CI 0.12–0.80, corresponding to a number needed to treat of 12.8 | 403 | 300 |
| **385** | RAZOR[400] | 2018 | The Lancet | To compare progression-free survival in patients with bladder cancer treated with open cystectomy and robot-assisted cystectomy | Progression free survival at 2 years following surgery | 2-year progression-free survival was 72.3% (95% CI 64.3 to 78.8) in the robotic cystectomy group and 71.6% (95% CI 63. to 78.2) in the open cystectomy group (difference 0.7%, 95% CI –9.6% to 10.9%; p for non-inferiority=0.001), indicating non-inferiority of robotic cystectomy | NR | 350 |
| **386** | HEALTH[401] | 2019 | The Lancet | To compare laparoscopic supracervical hysterectomy with endometrial ablation in women seeking surgical treatment for heavy menstrual bleeding | Co-primary clinical outcomes were patient satisfaction and condition-specific quality of life, measured with the menorrhagia multi-attribute quality of life scale (MMAS), assessed at 15 months after randomisation | Women randomly assigned to laparoscopic supracervical hysterectomy were also more likely to have the best possible MMAS score of 100 than women assigned to endometrial ablation (180 [69%] of 262 women vs 146 [54%] of 268 women; adjusted percentage difference 13.3, 95% CI 3.8–22.8; adjusted OR 1.87, 95% CI 1.31–2.67; p=0.00058) | 2552 | 664 |
| **387** | TOPKAT[402] | 2019 | The Lancet | To assess the clinical effectiveness and cost effectiveness of TKR versus partial knee replacement in patients with medial compartment osteoarthritis of the knee, and this represents an analysis of the main endpoints at 5 years | Oxford Knee Score (OKS) 5 years after randomisation | At the 5-year follow-up, we found no difference in OKS between groups (mean difference 1.04, 95% CI –0.42 to 2.50; p=0.159) | 962 | 531 |
| **388** | YOMEGA[403] | 2019 | The Lancet | To compare the outcomes of OAGB versus standard Roux-en-Y gastric bypass (RYGB) | Percentage excess BMI loss at 2 years | After 2 years, mean percentage excess BMI loss was –87.9% (SD 23.6) in the OAGB group and –85.8% (SD 23.1) in the RYGB group, confirming non-inferiority of OAGB (mean difference –3.3%, 95% CI –9.1 to 2.6) | 261 | 253 |

*Abbreviations: ACL= anterior cruciate ligament, AKS= american knee society, AOFAS= american othopaedic foot and ankle society score, ASC= adipose derived stem cells, ASES= american shoulder and elbow surgeons score (ASES), AVR= aortic valve replacement, CABG= coronary artery bypass grafting, CI= confidence interval, CS= constant score, CSF= cerebrospinal fluid, CO= combined osteotomy, CWO= closed wedge osteostomy, DASH= disability arm shoulder and hand score, DGE= delayed gastric emptying, DXA= dula x-ray absorptiometry, EJEVS= European Journal of Vascular and Endovascular Surgery, EPIC= expanded prostate cancer index composite, HAL= hemorrhoid artery ligation, HITS= high-intensity transient signals, HOS= hip outcome score, HR= hazard ratio, HKA= hip-knee-ankle angle, HTO= high tibial osteotomy, IKDC= international knee documentation committee, IGHL= inferior glenohumeral ligament, IIEF= international index of erectile function, IQR= interquartile range, ISGPF=international study group of pancreatic fistula, JAMA= Journal of the American Medical Association, JBJS= Journal of*

© 2021 Robinson NB et al. *JAMA Network Open*.

*Bone and Joint Surgery, JTCVS= Journal of Thoracic and Cardiovascular Surgery, KOOS= knee injury and osteoarthritis outcome score, KSS= knee society score, LVAD= left ventricular assist device, LVEDI= left ventricular end-systolic volume index, MDCT= multidetector row computed tomography, MRI= magnetic resonance imaging, NEJM= New England Journal of Medicine, OAT= osteochondral autologous transplantation, ODI= oswestry disability index, OI= outside in technique, OR= odds ratio, OS= overall survival, OWHTO= opening-wedge high tibial osteotomy, PAND= para-aortic nodal dissection, PD= pancreatoduodenectomy, POPF= post operative pancreatic fistula, POP-SS= pelvic organ prolapse symptom score, PREE= patient related elbow evaluation, PTFS= polytetrafluoroethylene, QALY= quality adjusted life year, RBL= rubber band ligation, SD= standard deviation, SLH= single leg hop test, SSI= surgical site infection, TKA= total knee arthroplasty, TP= transportal technique, TVE= tunnel volume enlargement, VAS= visual analog score, VAT-PP= video assisted thoracic partial pleurectomy, WOMAC= western Ontario and McMaster universities osteoarthiritis index, WOMET= western ontanio meniscal evaluation tool< WORC= western Ontario rotator cuff index*

© 2021 Robinson NB et al. *JAMA Network Open*.

**eTable 4:** General surgery trials included in the analysis by subspecialty

| Subspecialty | No. of trials (n=125) |
|---|---|
| **Acute Care Surgery** | 34 |
| **Colon and Rectal Surgery** | 33 |
| **Surgical Oncology** | 28 |
| **Minimally Invasive – Bariatric Surgery** | 14 |
| **Endocrine** | 6 |
| **Hepatobiliary Surgery** | 4 |
| **Breast Surgery** | 4 |
| **Plastic Surgery** | 2 |

© 2021 Robinson NB et al. *JAMA Network Open*.

**eTable 5:** Details of trials published in the analyzed surgical specialties. All values expressed as N (%) unless otherwise noted.

| | Cardiothoracic surgery | General Surgery | Neurosurgery | Orthopedic Surgery | Transplant | Vascular Surgery |
|---|---|---|---|---|---|---|
| **Number of trials** | 93 | 125 | 21 | 116 | 1 | 19 |
| **Multicenter trial** | 39 (41.9) | 76 (60.8) | 5 (23.8) | 26 (22.4) | 0 | 10 (52.6) |
| **Registered a priori** | 44 (47.3) | 114 (91.2) | 11 (52.4) | 53 (45.7) | 1 (100.0) | 6 (31.6) |
| **Discrepancy in primary outcome** | 14 (31.8) | 37 (32.5) | 4 (36.4) | 21 (39.6) | 0 | 2 (33.3) |
| **Intention-to-treat analysis** | 46 (49.5) | 110 (88.0) | 6 (28.6) | 40 (34.5) | 0 | 7 (36.8) |
| **Superiority design** | 82 (88.1) | 109 (87.2) | 18 (85.7) | 97 (83.6) | 0 | 13 (68.4) |
| **Composite primary outcome** | 11 (11.8) | 45 (36.0) | 0 | 21 (18.1) | 0 | 2 (10.5) |
| **Sample size, median (IQR)** | 129 (84-245) | 169 (107-334) | 90 (55-163) | 80 (60-127) | 105 | 118 (88-204) |
| **Multiplicity** | 53 (56.9) | 42 (33.6) | 9 (42.9) | 59 (50.9) | 0 | 6 (31.6) |
| **Fragility index, median (IQR)** | 2 (0-4) | 3 (1-6) | 3 (1-5) | 3[a] | NA[a] | 0 (0-6) |
| **Positive outcome** | 53 (56.9) | 55 (44.0) | 10 (47.6) | 33 (28.4) | 0 | 10 (52.6) |
| **Follow up (in months), median (IQR)** | 20 (12-36) | 12 (6-28) | 16 (15-21) | 24 (12-33) | 14 | 23 (8-28) |
| **Industry funded** | 21 (22.6) | 20 (16.0) | 6 (28.6) | 39 (33.6) | 0 | 8 (42.1) |
| **Percentage of screened patients enrolled, median (IQR)** | 51.2 (22.7-79.6) | 85.7 (58.5-96.6) | 81.7 (74.1-86.3) | 75.9 (50.9-93.0) | 100.0 | 99.7 (92.1-100.0) |
| **Lost to follow up, median (IQR)** | 1.5 (0.0-11.0) | 1.0 (0.0-11.0) | 3.0 (0.0-14.0) | 5.5 (2.0-18.0) | 5.0 | 7.0 (0.0-12.0) |
| **Percentage of cross-over, median (IQR)** | 1.62 (0.00-3.70) | 1.45 (0.00-3.71) | 0.62 (0.00-2.19) | 0.00 (0.00-0.47) | 1.90 | 0.00 (0.00-0.28) |

Footnote: [a]one trial in orthopedic surgery, and no trials in transplant were eligible for calculation of the fragility index

*Abbreviation: IQR= interquartile range*

© 2021 Robinson NB et al. *JAMA Network Open*.

**eTable 6:** Trial registration by year

| Year | Number of trials | Trials found in registry |
|---|---|---|
| **2008** | 37 | 6 (16.2) |
| **2009** | 37 | 17 (45.9) |
| **2010** | 26 | 15 (57.7) |
| **2011** | 32 | 12 (37.5) |
| **2012** | 35 | 22 (62.9) |
| **2013** | 32 | 19 (59.4) |
| **2014** | 28 | 21 (75.0) |
| **2015** | 36 | 26 (72.2) |
| **2016** | 31 | 21 (67.7) |
| **2017** | 25 | 22 (88.0) |
| **2018** | 30 | 26 (86.7) |
| **2019** | 39 | 35 (89.7) |

© 2021 Robinson NB et al. *JAMA Network Open*.

**eTable 7:** Comparison between trials with and without discrepancy in the primary outcome. All values expressed as N (%) unless otherwise noted.[a]

| | Trials registered a priori | Discrepancy in primary outcome | No discrepancy in primary outcome | P-value |
|---|---|---|---|---|
| **Total no. of trials** | 226 | 77 | 149 | - |
| **Impact Factor, median (IQR)** | 9.20 (4.84- 9.20) | 9.20 (4.84-9.20) | 9.20 (4.84-9.20) | 0.40 |
| **Year, median (IQR)** | 2015 (2012-2017) | 2015 (2012-2017) | 2015 (2012-2018) | 0.24 |
| **Composite primary endpoint** | 65 (28.8) | 28 (36.4) | 37 (24.8) | 0.10 |
| **Months follow up, median (IQR)** | 14.0 (12.0-25.1) | 12.5 (12.0-24.0) | 14.0 (12.0-28.0) | 0.60 |
| **Any funding reported** | 167 (73.9) | 62 (80.5) | 105 (70.5) | 0.14 |
| **Industry funding** | 56 (24.7) | 22 (28.6) | 34 (22.8) | 0.81 |
| **Industry involved in analysis** | 26 (46.4) | 11 (50.0) | 15 (44.1) | 0.87 |
| **Multi-institutional** | 124 (54.9) | 39 (50.6) | 85 (57.0) | 0.44 |
| **Number of patients screened, median (IQR)** | 245 (126-541) | 204 (114-585) | 262 (134-508) | 0.44 |
| **Sample size, median (IQR)** | 150 (96-294) | 139 (97-250) | 160 (95-302) | 0.38 |
| **Intention-to-treat analysis** | 160 (70.8) | 46 (59.7) | 114 (76.5) | 0.03 |
| **Total number of citations, median (IQR)** | 39 (15-103) | 42 (15-99) | 37 (16-108) | 0.90 |
| **Non-inferiority design** | 36 (15.9) | 11 (14.3) | 25 (16.7) | 0.71 |
| **Subspecialty** | - | - | - | - |
| ➢ **Cardiothoracic Surgery** | 44 (19.5) | 14 (18.2) | 30 (20.1) | 0.86 |
| ➢ **General Surgery** | 113 (50.0) | 37 (48.1) | 76 (51.0) | 0.78 |
| ➢ **Neurosurgery** | 11 (4.9) | 4 (5.2) | 7 (4.7) | 1.00 |
| ➢ **Orthopedic Surgery** | 50 (22.1) | 21 (27.3) | 29 (19.5) | 0.24 |
| ➢ **Transplant** | 1 (0.4) | 0 (0.0) | 1 (0.7) | 1.00 |
| ➢ **Vascular Surgery** | 7 (3.1) | 1 (1.3) | 6 (4.0) | 0.47 |
| **PRECIS-2 score, mean (SD)** | 3.54 (0.66) | 3.38 (0.63) | 3.63 (0.66) | 0.008 |
| **High Risk of Bias** | 47 (20.8) | 20 (26.0) | 27 (18.1) | 0.17 |

*Abbreviations: IQR= interquartile range*

Footnote: [a]Only subspecialties included in the systematic search were included in this subgroup analysis

© 2021 Robinson NB et al. *JAMA Network Open*.

**eTable 8**: Comparison between superiority and non-inferiority trials. All values reported as N (%) unless otherwise noted.*

| | Overall | Superiority | Non-Inferiority | P-value |
|---|---|---|---|---|
| **Total no. of trials** | 371 | 319 | 52 | - |
| **Impact Factor, median (IQR)** | 4.88 (4.43, 9.20) | 4.88 (4.43, 9.20) | 4.84 (4.61, 9.20) | 0.27 |
| **Year, median (IQR)** | 2013 (2010, 2016) | 2013 (2010, 2016) | 2013 (2010, 2016) | 0.68 |
| **Composite primary endpoint** | 78 (21.0) | 67 (21.0) | 11 (21.2) | 1.00 |
| **Months follow up, median (IQR)** | 24.00 (12.00, 31.80) | 24.00 (12.00, 31.90) | 19.00 (12.00, 24.00) | 0.59 |
| **Any funding reported** | 271 (73.0) | 233 (73.0) | 38 (73.1) | 1.00 |
| **Industry funding** | 92 (24.8) | 75 (23.5) | 17 (32.7) | 0.18 |
| **Industry involved in analysis** | 48 (52.2) | 38 (50.6) | 10 (58.8) | 0.28 |
| **Multi-intuitional** | 153 (41.2) | 128 (40.1) | 25 (48.1) | 0.35 |
| **Number of patients screened, median (IQR)** | 195 (102, 432) | 180 (96, 418) | 215 (136, 509) | 0.16 |
| **Sample size, median (IQR)** | 120 (68, 223) | 120 (66, 219) | 163 (86, 265) | 0.11 |
| **Intention-to-treat analysis** | 207 (55.8) | 185 (58.0) | 22 (42.3) | 0.004 |
| **Fragility Index, median (IQR)** | 2.50 (1.00, 5.00) | 3.00 (1.00, 5.50) | 0.00 (0.00, 1.00) | 0.09 |
| **Favorable outcome** | 159 (43.9) | 141 (44.9) | 18 (37.5) | 0.42 |
| **Total number of citations, median (IQR)** | 34.00 (14.00, 90.00) | 32.00 (12.50, 84.00) | 43.50 (25.25, 106.00) | 0.02 |
| **Subspecialty** | - | - | - | - |
| ➢ **Cardiothoracic Surgery** | 92 (24.8)[a] | 82 (25.7) | 10 (19.2) | 0.41 |
| ➢ **General Surgery** | 122 (32.9)[a] | 109 (34.2) | 13 (25.0) | 0.25 |
| ➢ **Neurosurgery** | 21 (5.7) | 18 (5.6) | 3 (5.8) | 1.00 |
| ➢ **Orthopedic Surgery** | 116 (31.3) | 97 (30.4) | 19 (36.5) | 0.47 |
| ➢ **Transplant** | 1 (0.3) | 0 (0.0) | 1 (1.9) | 0.30 |
| ➢ **Vascular Surgery** | 19 (5.1) | 13 (4.1) | 6 (11.5) | 0.05 |
| **Presence of spin** | 108 (53.5) | 94 (55.0) | 14 (45.2) | 0.42 |
| **PRECIS-2 score, mean (SD)** | 3.51 (0.65) | 3.55 (0.62) | 3.24 (0.77) | 0.002 |
| **High Risk of Bias** | 90 (24.3) | 84 (26.3) | 6 (11.5) | 0.02 |

Footnote: [a]Trials classified as "Both superiority & non-inferiority" (n=2) or where the trial design was not clear (n=2) were excluded from this comparison. Only subspecialties included in the systematic search were included in this subgroup analysis.

© 2021 Robinson NB et al. *JAMA Network Open*.

**eTable 9:** Cochrane Risk of Bias 2 Tool Domains

| Domain | Overall (n=388) |
|---|---|
| **Domain 1: Randomization process** | - |
| • **High risk of bias** | 8 (2.1) |
| • **Some concerns for bias** | 167 (43.0) |
| • **Low risk of bias** | 213 (54.9) |
| **Domain 2: Deviations from intended interventions** | - |
| • **High risk of bias** | 1 (0.3) |
| • **Some concerns for bias** | 52 (13.4) |
| • **Low risk of bias** | 335 (86.3) |
| **Domain 3: Missing outcome data** | - |
| • **High risk of bias** | 1 (0.3) |
| • **Some concerns for bias** | 10 ( 2.6) |
| • **Low risk of bias** | 373 (97.1) |
| **Domain 4: Measurement of the outcome** | - |
| • **High risk of bias** | 11 (2.8) |
| • **Some concerns for bias** | 120 (30.9) |
| • **Low risk of bias** | 257 (66.2) |
| **Domain 5: Selection of the reported result** | - |
| • **High risk of bias** | 85 (22.0) |
| • **Some concerns for bias** | 133 (34.4) |
| • **Low risk of bias** | 169 (43.7) |
| **Domain 6: Overall Bias** | - |
| • **High risk of bias** | 91 (23.5) |
| • **Some concerns for bias** | 211 (54.4) |
| • **Low risk of bias** | 86 (22.2) |

© 2021 Robinson NB et al. *JAMA Network Open*.

**eTable 10**: PRECIS-2 assessment of trial pragmatism across the design domains.

| | Overall (n=388) |
|---|---|
| **PRECIS-2 overall mean score (±SD)** | **3.52 (0.65)** |
| **Eligibility criteria mean score (±SD)** | **3.50 (0.97)** |
| ➢ Very explanatory | 10 (2.6) |
| ➢ Rather explanatory | 43 (11.1) |
| ➢ Equally pragmatic and explanatory | 129 (33.3) |
| ➢ Rather pragmatic | 152 (39.3) |
| ➢ Very pragmatic | 53 (13.7) |
| **Recruitment mean score (±SD)** | **4.41 (0.78)** |
| ➢ Very explanatory | 0 (0.0) |
| ➢ Rather explanatory | 6 (1.5) |
| ➢ Equally pragmatic and explanatory | 54 (13.9) |
| ➢ Rather pragmatic | 103 (26.5) |
| ➢ Very pragmatic | 225 (58.0) |
| **Setting mean score (±SD)** | **3.90 (1.23)** |
| ➢ Very explanatory | 39 (10.1) |
| ➢ Rather explanatory | 7 (1.8) |
| ➢ Equally pragmatic and explanatory | 59 (15.2) |
| ➢ Rather pragmatic | 131 (33.8) |
| ➢ Very pragmatic | 152 (39.2) |
| **Organization mean score (±SD)** | **3.44 (1.35)** |
| ➢ Very explanatory | 62 (16.0) |
| ➢ Rather explanatory | 24 (6.2) |
| ➢ Equally pragmatic and explanatory | 79 (20.4) |
| ➢ Rather pragmatic | 127 (32.7) |
| ➢ Very pragmatic | 96 (24.7) |
| **Flexibility Delivery mean score (±SD)** | **2.90 (1.35)** |
| ➢ Very explanatory | 90 (23.3) |
| ➢ Rather explanatory | 55 (14.2) |
| ➢ Equally pragmatic and explanatory | 91 (23.5) |
| ➢ Rather pragmatic | 105 (27.1) |
| ➢ Very pragmatic | 46 (11.9) |
| **Flexibility Adherence mean score (±SD)** | **3.12 (1.33)** |
| ➢ Very explanatory | 76 (19.7) |
| ➢ Rather explanatory | 28 (7.3) |
| ➢ Equally pragmatic and explanatory | 113 (29.3) |
| ➢ Rather pragmatic | 112 (29.0) |
| ➢ Very pragmatic | 57 (14.8) |
| **Follow-up mean score (±SD)** | **2.97 (1.43)** |
| ➢ Very explanatory | 77 (20.6) |

© 2021 Robinson NB et al. *JAMA Network Open*.

| | |
|---|---|
| ➢ Rather explanatory | 52 (13.9) |
| ➢ Equally pragmatic and explanatory | 102 (27.3) |
| ➢ Rather pragmatic | 88 (23.6) |
| ➢ Very pragmatic | 54 (14.5) |
| **Primary outcome mean score (±SD)** | **3.63 (1.26)** |
| ➢ Very explanatory | 24 (6.2) |
| ➢ Rather explanatory | 52 (13.5) |
| ➢ Equally pragmatic and explanatory | 93 (24.1) |
| ➢ Rather pragmatic | 90 (23.3) |
| ➢ Very pragmatic | 127 (32.9) |
| **Primary analysis mean score (±SD)** | **3.84 (2.01)** |
| ➢ Very explanatory | 74 (22.1) |
| ➢ Rather explanatory | 6 (1.8) |
| ➢ Equally pragmatic and explanatory | 15 (4.5) |
| ➢ Rather pragmatic | 46 (13.7) |
| ➢ Very pragmatic | 194 (57.9) |

For all domains, the very explanatory, rather explanatory, equally explanatory/pragmatic, rather pragmatic, and very pragmatic categories were defined as the PRECIS-2 score =1, >1 and <3, =3, >3 and <5, and =5, respectively.

© 2021 Robinson NB et al. *JAMA Network Open*.

**eTable 11**: Mean PRECIS-2 score among different specialties.*

| | N | PRECIS-2 score, mean (SD) |
|---|---|---|
| **Cardiothoracic Surgery** | 93 | 3.49 (0.55) |
| **General Surgery** | 125 | 3.70 (0.53) |
| **Neurosurgery** | 21 | 3.58 (0.54) |
| **Orthopedic Surgery** | 116 | 3.36 (0.81) |
| **Transplant** | 1 | 3.22 (NA) |
| **Vascular Surgery** | 19 | 3.21 (0.50) |

*Abbreviations: NA= not available, PRECIS-2=Pragmatic–Explanatory Continuum Indicator Summary -2, SD= standard deviation.*

*Only subspecialties included in the systematic search were included in this subgroup analysis

© 2021 Robinson NB et al. *JAMA Network Open*.

**eTable 12**: Comparison between trials with and without spin.[a]

| | Evaluated for spin | Spin present | No spin present | P-value |
|---|---|---|---|---|
| **Total no. of trials** | 203 | 109 | 94 | - |
| **Impact Factor, median (IQR)** | 4.84 (4.52-9.20) | 4.84 (4.43-9.20) | 4.84 (4.61-9.20) | 0.96 |
| **Year, median (IQR)** | 2013 (2010-2016) | 2014 (2011-2016) | 2012 (2010-2016) | 0.29 |
| **Composite primary endpoint** | 45 (22.2) | 25 (22.9) | 20 (21.3) | 0.91 |
| **Months follow up, median (IQR)** | 24.0 (12.0-31.0) | 23.0 (12.0-28.0) | 24.0 (12.0-36.0) | 0.93 |
| **Any funding reported** | 148 (72.9) | 80 (73.4) | 68 (72.3) | 0.99 |
| **Industry funding** | 54 (36.5) | 30 (37.5) | 24 (35.3) | 0.92 |
| **Industry involved in analysis** | 25 (12.3) | 14 (12.8) | 11 (11.7) | 1.00 |
| **Multi-institutional** | 88 (43.3) | 51 (46.8) | 37 (39.4) | 0.36 |
| **Number of patients screened, median (IQR)** | 204 (102-415) | 194 (99-372) | 214 (121-491) | 0.41 |
| **Sample size, median (IQR)** | 126 (79-226) | 120 (80-200) | 133 (78-289) | 0.23 |
| **Intention-to-treat analysis** | 109 (53.6) | 62 (56.9) | 47 (50.0) | 0.96 |
| **Total number of citations, median (IQR)** | 31.0 (14.5-90.5) | 35.0 (15.00-86.0) | 27.5 (12.2-93.2) | 0.45 |
| **Non-inferiority design** | 31 (15.3) | 14 (12.8) | 17 (18.1) | 0.42 |
| **Subspecialty** | - | - | - | - |
| ➢ **Cardiothoracic Surgery** | 37 (18.2) | 9 (8.3) | 28 (29.8) | <0.001 |
| ➢ **General Surgery** | 63 (31.0) | 41 (37.6) | 22 (23.4) | 0.04 |
| ➢ **Neurosurgery** | 11 (5.4) | 7 (6.4) | 4 (4.3) | 0.71 |
| ➢ **Orthopedic Surgery** | 82 (40.4) | 47 (43.1) | 35 (37.2) | 0.48 |
| ➢ **Transplant** | 1 (0.5) | 1 (0.9) | 0 | 1.00 |
| ➢ **Vascular Surgery** | 9 (4.4) | 4 (3.7) | 5 (5.3) | 0.82 |
| **Mean PRECIS-2 score, mean (SD)** | 3.49 (0.70) | 3.51 (0.71) | 3.46 (0.70) | 0.63 |
| **High Risk of Bias** | 44 (21.7) | 23 (21.1) | 21 (22.3) | 0.83 |

*Abbreviations: IQR= interquartile range, PRECIS-2=Pragmatic–Explanatory Continuum Indicator Summary -2.*

[a]Only subspecialities included in the systematic search were included in this subgroup analysis.

© 2021 Robinson NB et al. *JAMA Network Open*.

**eTable 13**: Details of Spin Appraisal. All values expressed at N (%).[a]

| Variable | Abstract | Main Text |
|---|---|---|
| **Spin present** | 109/211 (51.6) | |
| **Spin in the title** | 10 (4.7) | |
| **Spin in the results** | 47 (22.3) | 40 (19.0) |
| ➢ Focus on statistically significant within group | 8 (3.8) | 4 (1.9) |
| ➢ Focus on statistically significant secondary outcome | 23 (10.9) | 17 (8.1) |
| ➢ Focus on statistically significant subgroup analyses | 8 (3.8) | 8 (3.8) |
| ➢ Focus on statistically significant modified population of analysis | 3 (1.4) | 1 (0.5) |
| ➢ Focus on statistically significant within and between group comparisons for secondary outcomes | 3 (1.4) | 6 (2.8) |
| ➢ Other | 6 (2.9) | 7 (3.3) |
| **Spin in the discussion** | NA | 49 (23.2) |
| ➢ Reporting of statistically non-significant outcome as if the trial were an equivalence trial | NA | 16 (7.6) |
| ➢ Focus on statistically significant secondary outcomes | NA | 21 (10.0) |
| ➢ Focus on statistically significant subgroup analyses | NA | 6 (2.8) |
| ➢ Focus on statistically significant modified population of analyses | NA | 2 (0.9) |
| ➢ Focus on statistically significant modified population of analyses | NA | 1 (0.5) |
| ➢ Focus on overall within group improvement | NA | 4 (1.9) |
| ➢ Ruling out adverse event | NA | 1 (0.5) |
| ➢ Other | NA | 10 (4.7) |
| **Spin in the conclusion** | 83 (39.3) | 81 (38.6) |
| ➢ Focus only on treatment effectiveness | 9 (4.3) | 10 (4.7) |
| ➢ Claiming equivalence for statistically non-significant results | 24 (11.4) | 16 (7.6) |
| ➢ Claiming efficacy with no consideration of the statistically non-significant primary outcome | 20 (9.5) | 18 (8.5) |
| ➢ Focusing only on statistically significant results | 14 (6.6) | 15 (7.1) |
| ➢ Acknowledge statistically non-significant results for the primary outcome but emphasize the beneficial effect of treatment | 16 (7.6) | 19 (9.0) |
| ➢ Acknowledge statistically non-significant results for the primary outcome but emphasize other statistically significant results | 16 (7.6) | 15 (7.1) |
| ➢ Interpreting the negative results of primary outcome as showing equivalence | 6 (2.8) | 3 (1.4) |
| ➢ Claiming or emphasizing the non-inferiority despite not establishing non-inferiority inconclusive | 14 (6.6) | 12 (5.7) |
| ➢ Conclusion ruling out an adverse event on statistically non-significant results | 2 (0.9) | 1 (0.5) |

© 2021 Robinson NB et al. *JAMA Network Open*.

| | | |
|---|---|---|
| ➢ Recommendation to use the treatment | 15 (7.1) | 16 (7.6) |
| ➢ Focus on another objective | 2 (0.9) | 1 (0.5) |
| ➢ Comparison with placebo group of another trial | 0 (0.0) | 0 (0.0) |
| ➢ Other | 2 (0.9) | 2 (0.9) |
| ➢ Statistically non-significant subgroup results reported as beneficial | 3 (1.4) | 3 (1.4) |

[a]*Only trials with primary outcome reported and non-significant were evaluated for spin*

© 2021 Robinson NB et al. *JAMA Network Open*.

**eTable 14**: Number of citations by journal.

| Journal | No. of trials | Median number of citations (interquartile range) |
|---|---|---|
| ***Annals of Surgery*** | 103 | 45.0 (17.5-95.5) |
| ***Journal of Bone and Joint Surgery*** | 63 | 60.0 (26.0-109.0) |
| ***Arthroscopy*** | 47 | 21.0 (9.5-43.0) |
| ***The Journal of Thoracic and Cardiovascular Surgery*** | 44 | 20.0 (9.7-35.2) |
| ***The Annals of Thoracic Surgery*** | 32 | 16.5 (7.0-40.5) |
| ***The Lancet*** | 29 | 78.0 (50.0-152.0) |
| ***The New England Journal of Medicine*** | 21 | 268.0 (168.0-401.0) |
| ***European Journal of Vascular and Endovascular Surgery*** | 10 | 24.5 (21.5-31.7) |
| ***Journal of Neurosurgery*** | 10 | 14.0 (3.2-27.2) |
| ***Neurosurgery*** | 10 | 20.5 (8.7-54.5) |
| ***Journal of Vascular Surgery*** | 9 | 14.0 (7.0-37.0) |
| ***JAMA Surgery*** | 9 | 32.0 (18.0-58.0) |
| ***American Journal of Transplantation*** | 1 | 30.0 (30.0-30.0) |

*Abbreviations: JAMA= Journal of the American Medical Association*

© 2021 Robinson NB et al. *JAMA Network Open*.

**eTable 15**: Number of citations by specialty.[a]

| | N | Median number of citations (interquartile range) |
|---|---|---|
| **Cardiothoracic Surgery** | 93 | 25.0 (10.0-56.0) |
| **General Surgery** | 125 | 50.0 (20.0-114.0) |
| **Neurosurgery** | 21 | 20.0 (5.0-36.0) |
| **Orthopedic Surgery** | 116 | 39.0 (16.0-100.0) |
| **Transplant** | 1 | 30.0 (30.0-30.0) |
| **Vascular Surgery** | 19 | 28.0 (19.5-53.5) |

[a]Only subspecialities included in the systematic search were included in this subgroup analysis.

© 2021 Robinson NB et al. *JAMA Network Open*.

**eFigure 1:** Consolidated Standards of Reporting Trials (CONSORT) flow diagram. RCT= randomized controlled trial.

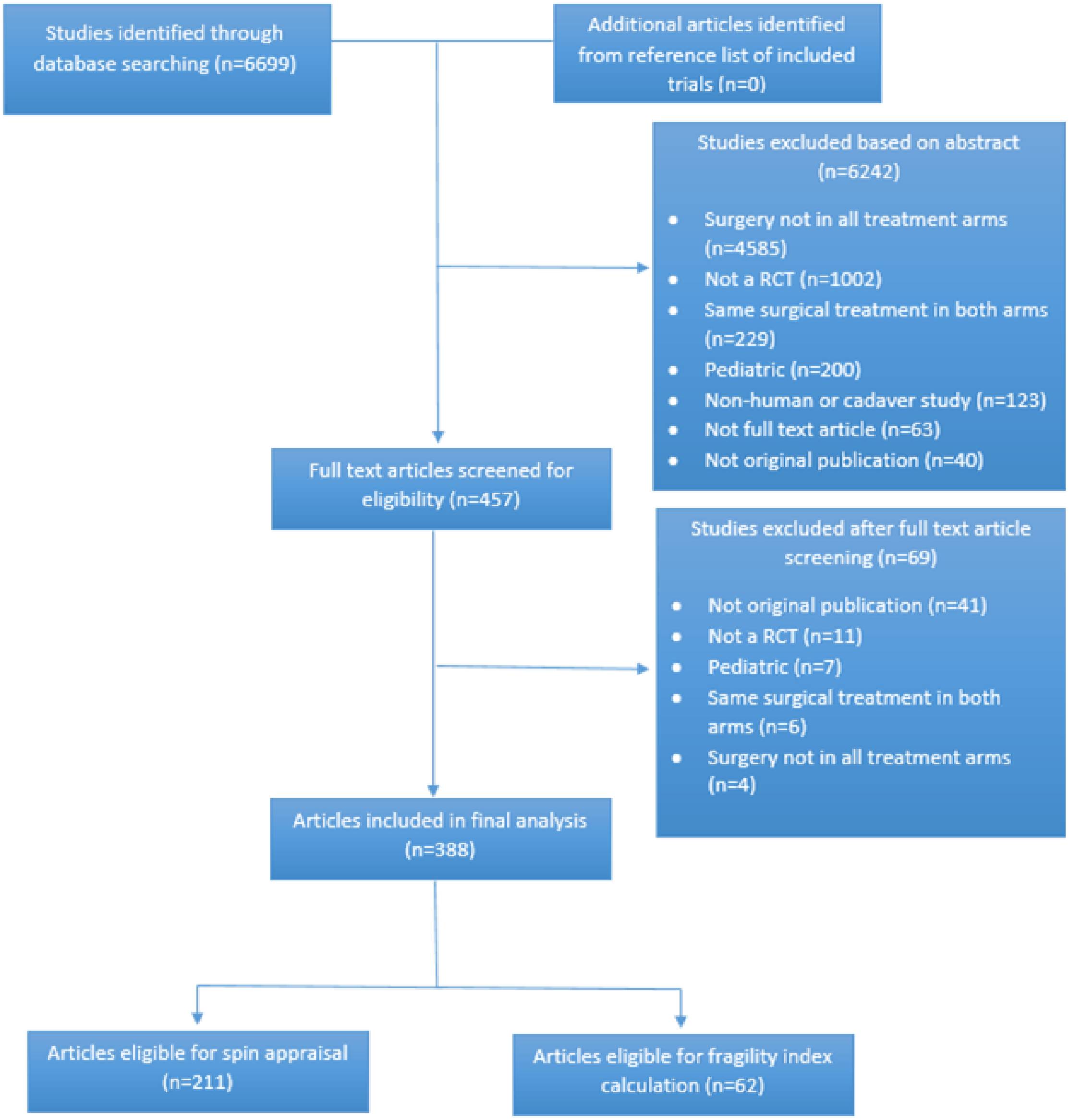


© 2021 Robinson NB et al. *JAMA Network Open*.

**eFigure 2**: Temporal trend for percentage of trials registered a priori. RTC= randomized controlled trial.

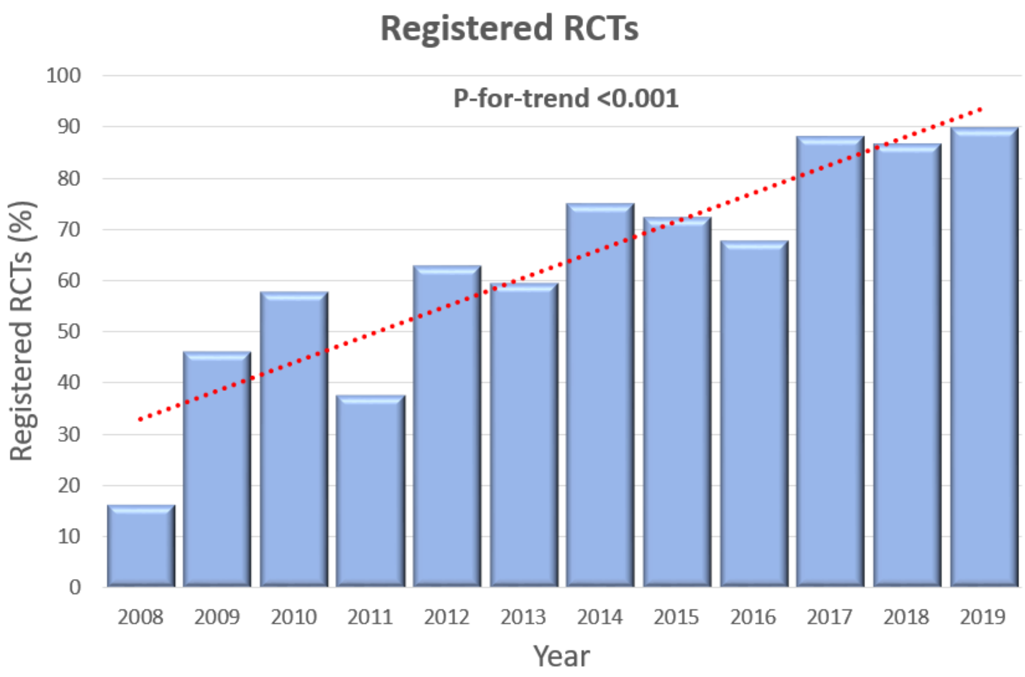


© 2021 Robinson NB et al. *JAMA Network Open*.

**eFigure 3**: Hypothesized treatment effect used in sample size calculation.

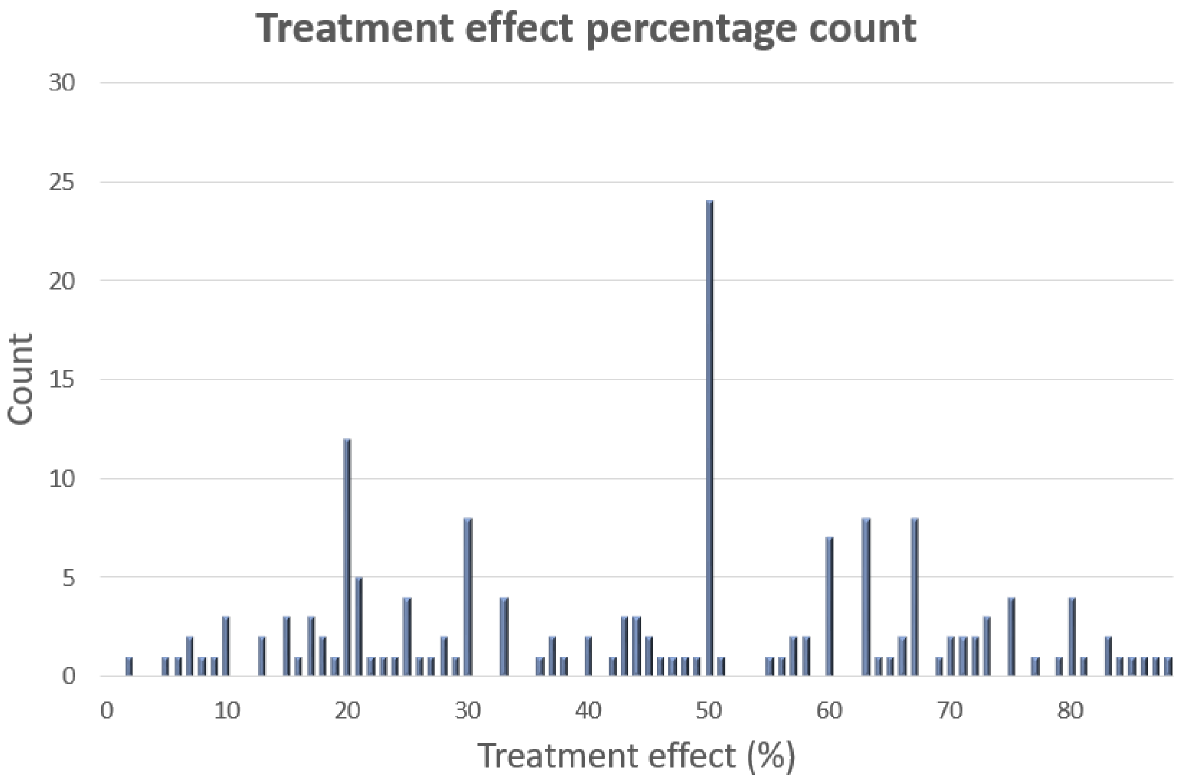


© 2021 Robinson NB et al. *JAMA Network Open*.

**eFigure 4**: Distribution of fragility index.

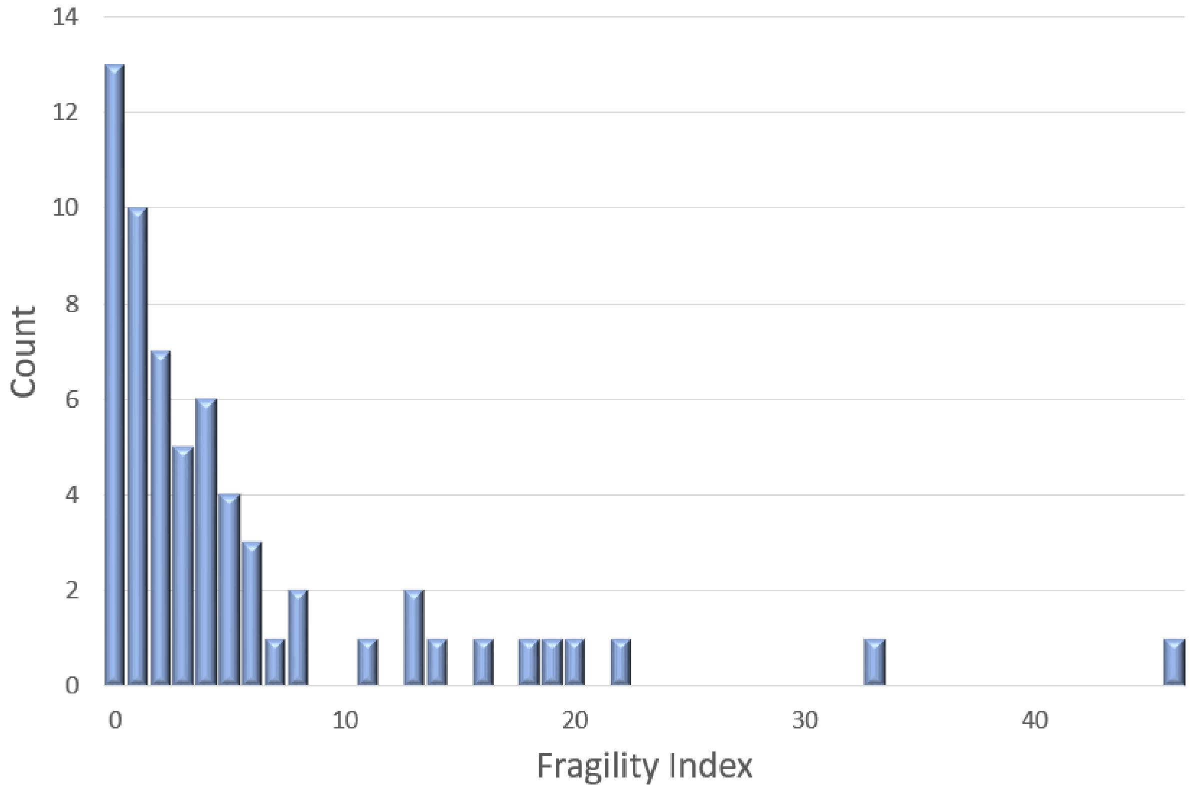


© 2021 Robinson NB et al. *JAMA Network Open*.

**eFigure 5**: Distribution of fragility index minus lost to follow-up (FI-LTF).

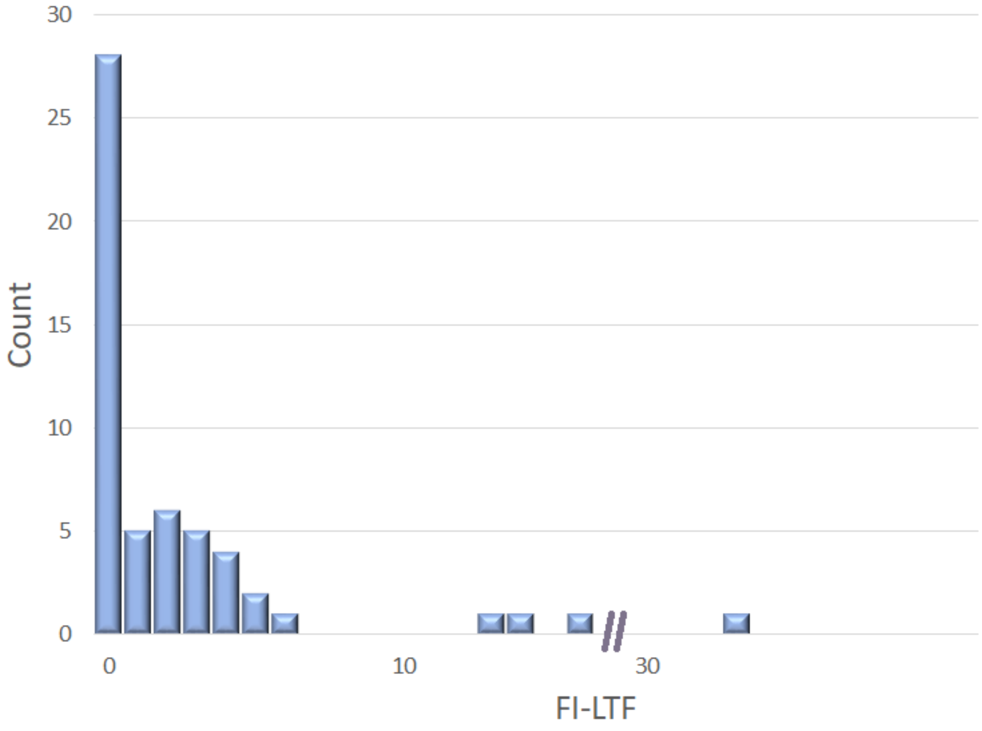


© 2021 Robinson NB et al. *JAMA Network Open*.

**References:**


1 2018 Latest Impact Factors: Clarivate Analytics - Journal Citation Reports. 2019.

2 Gaudino M, Hameed I, Rahouma M, *et al.* Characteristics of Contemporary Randomized Clinical Trials and Their Association With the Trial Funding Source in Invasive Cardiovascular Interventions. *JAMA Intern Med* 2020; **180**: 993.

3 Sepehrvand N, Alemayehu W, Das D, *et al.* Trends in the Explanatory or Pragmatic Nature of Cardiovascular Clinical Trials Over 2 Decades. *JAMA Cardiol* 2019; published online Sept 1. DOI:10.1001/jamacardio.2019.3604.

4 Loudon K, Treweek S, Sullivan F, Donnan P, Thorpe KE, Zwarenstein M. The PRECIS-2 tool: designing trials that are fit for purpose. *BMJ* 2015; **350**: h2147–h2147.

5 Li G, Taljaard M, Van den Heuvel ER, *et al.* An introduction to multiplicity issues in clinical trials: the what, why, when and how. *Int J Epidemiol* 2016; : dyw320.

6 Khan MS, Khan MS, Ansari ZN, *et al.* Prevalence of Multiplicity and Appropriate Adjustments Among Cardiovascular Randomized Clinical Trials Published in Major Medical Journals. *JAMA Netw Open* 2020; **3**: e203082.

7 Flacco ME, Manzoli L, Boccia S, *et al.* Head-to-head randomized trials are mostly industry sponsored and almost always favor the industry sponsor. *J Clin Epidemiol* 2015; **68**: 811–20.

8 Hopewell S, Loudon K, Clarke MJ, Oxman AD, Dickersin K. Publication bias in clinical trials due to statistical significance or direction of trial results. *Cochrane Database Syst Rev* 2009; : MR000006.

9 Ioannidis JP. Effect of the statistical significance of results on the time to completion and publication of randomized efficacy trials. *JAMA* 1998; **279**: 281–6.

10 Boutron I, Dutton S, Ravaud P, Altman DG. Reporting and interpretation of randomized controlled trials with statistically nonsignificant results for primary outcomes. *JAMA* 2010; **303**: 2058–64.

11 Hernandez AV, Pasupuleti V, Deshpande A, Thota P, Collins JA, Vidal JE. Deficient reporting and interpretation of non-inferiority randomized clinical trials in HIV patients: a systematic review. *PLoS ONE* 2013; **8**: e63272.

12 Chan A-W, Hróbjartsson A, Haahr MT, Gøtzsche PC, Altman DG. Empirical evidence for selective reporting of outcomes in randomized trials: comparison of protocols to published articles. *JAMA* 2004; **291**: 2457–65.

13 Chen T, Li C, Qin R, *et al.* Comparison of Clinical Trial Changes in Primary Outcome and Reported Intervention Effect Size Between Trial Registration and Publication. *JAMA Netw Open* 2019; **2**: e197242.

14 Walsh M, Srinathan SK, McAuley DF, *et al.* The statistical significance of randomized controlled trial results is frequently fragile: a case for a Fragility Index. *J Clin Epidemiol* 2014; **67**: 622–8.


© 2021 Robinson NB et al. *JAMA Network Open*.

15 Mazzinari G, Ball L, Serpa Neto A, *et al.* The fragility of statistically significant findings in randomised controlled anaesthesiology trials: systematic review of the medical literature. *British Journal of Anaesthesia* 2018; **120**: 935–41.

16 Sterne JAC, Savović J, Page MJ, *et al.* RoB 2: a revised tool for assessing risk of bias in randomised trials. *BMJ* 2019; : l4898.

17 Aull MJ, Afaneh C, Charlton M, *et al.* A Randomized, Prospective, Parallel Group Study of Laparoscopic Versus Laparoendoscopic Single Site Donor Nephrectomy for Kidney Donation: Laparoendoscopic Single Site Donor Nephrectomy. *American Journal of Transplantation* 2014; **14**: 1630–7.

18 Hewett PJ, Allardyce RA, Bagshaw PF, *et al.* Short-Term Outcomes of the Australasian Randomized Clinical Study Comparing Laparoscopic and Conventional Open Surgical Treatments for Colon Cancer: The ALCCaS Trial. *Annals of Surgery* 2008; **248**: 728–38.

19 Fein M, Fuchs K-H, Thalheimer A, Freys SM, Heimbucher J, Thiede A. Long-Term Benefits of Roux-en-Y Pouch Reconstruction After Total Gastrectomy: A Randomized Trial. *Annals of Surgery* 2008; **247**: 759–65.

20 Fernández-Cruz L, Cosa R, Blanco L, López-Boado MA, Astudillo E. Pancreatogastrostomy With Gastric Partition After Pylorus-Preserving Pancreatoduodenectomy Versus Conventional Pancreatojejunostomy: A Prospective Randomized Study. *Annals of Surgery* 2008; **248**: 930–8.

21 Zavagno G, De Salvo GL, Scalco G, *et al.* A Randomized Clinical Trial on Sentinel Lymph Node Biopsy Versus Axillary Lymph Node Dissection in Breast Cancer: Results of the Sentinella/GIVOM Trial. *Annals of Surgery* 2008; **247**: 207–13.

22 Hayashi S, Takayama T, Masuda H, *et al.* Bioresorbable Membrane to Reduce Postoperative Small Bowel Obstruction in Patients With Gastric Cancer: A Randomized Clinical Trial. *Annals of Surgery* 2008; **247**: 766–70.

23 Karamanakos SN, Vagenas K, Kalfarentzos F, Alexandrides TK. Weight Loss, Appetite Suppression, and Changes in Fasting and Postprandial Ghrelin and Peptide-YY Levels After Roux-en-Y Gastric Bypass and Sleeve Gastrectomy: A Prospective, Double Blind Study. *Annals of Surgery* 2008; **247**: 401–7.

24 Kim Y-W, Baik YH, Yun YH, *et al.* Improved Quality of Life Outcomes After Laparoscopy-Assisted Distal Gastrectomy for Early Gastric Cancer: Results of a Prospective Randomized Clinical Trial. *Annals of Surgery* 2008; **248**: 721–7.

25 Koh YW, Park JH, Lee SW, Choi EC. The Harmonic Scalpel Technique Without Supplementary Ligation in Total Thyroidectomy with Central Neck Dissection: A Prospective Randomized Study. *Annals of Surgery* 2008; **247**: 945–9.

26 Ikeda M, Hasegawa K, Sano K, *et al.* The Vessel Sealing System (LigaSure) in Hepatic Resection: A Randomized Controlled Trial. *Annals of Surgery* 2009; **250**: 199–203.

© 2021 Robinson NB et al. *JAMA Network Open*.

27 Seiler CM, Bruckner T, Diener MK, *et al.* Interrupted or Continuous Slowly Absorbable Sutures For Closure of Primary Elective Midline Abdominal Incisions: A Multicenter Randomized Trial (INSECT: ISRCTN24023541). *Annals of Surgery* 2009; **249**: 576–82.

28 Kouhia STH, Huttunen R, Silvasti SO, *et al.* Lichtenstein Hernioplasty Versus Totally Extraperitoneal Laparoscopic Hernioplasty in Treatment of Recurrent Inguinal Hernia—A Prospective Randomized Trial: *Annals of Surgery* 2009; **249**: 384–7.

29 Nguyen NT, Slone JA, Nguyen X-MT, Hartman JS, Hoyt DB. A Prospective Randomized Trial of Laparoscopic Gastric Bypass Versus Laparoscopic Adjustable Gastric Banding for the Treatment of Morbid Obesity: Outcomes, Quality of Life, and Costs. *Transactions of the . Meeting of the American Surgical Association* 2009; **127**: 262–72.

30 Peterli R, Wölnerhanssen B, Peters T, *et al.* Improvement in Glucose Metabolism After Bariatric Surgery: Comparison of Laparoscopic Roux-en-Y Gastric Bypass and Laparoscopic Sleeve Gastrectomy: A Prospective Randomized Trial. *Annals of Surgery* 2009; **250**: 234–41.

31 Seiler CM, Deckert A, Diener MK, *et al.* Midline Versus Transverse Incision in Major Abdominal Surgery: A Randomized, Double-Blind Equivalence Trial (POVATI: ISRCTN60734227). *Annals of Surgery* 2009; **249**: 913–20.

32 Serra-Aracil X, Bombardo-Junca J, Moreno-Matias J, *et al.* Randomized, Controlled, Prospective Trial of the Use of a Mesh to Prevent Parastomal Hernia: *Annals of Surgery* 2009; **249**: 583–7.

33 Klarenbeek BR, Veenhof AA, Bergamaschi R, *et al.* Laparoscopic Sigmoid Resection for Diverticulitis Decreases Major Morbidity Rates: A Randomized Control Trial: Short-term Results of the Sigma Trial. *Annals of Surgery* 2009; **249**: 39–44.

34 Weiss S, Schmidt S-C, Ulrich F, *et al.* Biliary Reconstruction Using a Side-to-Side Choledochocholedochostomy With or Without T-Tube in Deceased Donor Liver Transplantation: A Prospective Randomized Trial. *Annals of Surgery* 2009; **250**: 766–71.

35 Aboulian A, Chan T, Yaghoubian A, *et al.* Early Cholecystectomy Safely Decreases Hospital Stay in Patients With Mild Gallstone Pancreatitis: A Randomized Prospective Study. *Annals of Surgery* 2010; **251**: 615–9.

36 Daraï E, Dubernard G, Coutant C, Frey C, Rouzier R, Ballester M. Randomized Trial of Laparoscopically Assisted Versus Open Colorectal Resection for Endometriosis: Morbidity, Symptoms, Quality of Life, and Fertility. *Annals of Surgery* 2010; **251**: 1018–23.

37 Gervaz P, Inan I, Perneger T, Schiffer E, Morel P. A Prospective, Randomized, Single-Blind Comparison of Laparoscopic Versus Open Sigmoid Colectomy for Diverticulitis: *Annals of Surgery* 2010; **252**: 3–8.

38 Kim H-H, Hyung WJ, Cho GS, *et al.* Morbidity and Mortality of Laparoscopic Gastrectomy Versus Open Gastrectomy for Gastric Cancer: An Interim Report—A Phase III Multicenter, Prospective, Randomized Trial (KLASS Trial). *Annals of Surgery* 2010; **251**: 417–20.

© 2021 Robinson NB et al. *JAMA Network Open*.

39 Langeveld HR, van't Riet M, Weidema WF, *et al.* Total Extraperitoneal Inguinal Hernia Repair Compared With Lichtenstein (the LEVEL-Trial): A Randomized Controlled Trial. *Annals of Surgery* 2010; **251**: 819–24.

40 Peeters E, Spiessens C, Oyen R, *et al.* Laparoscopic Inguinal Hernia Repair in Men With Lightweight Meshes May Significantly Impair Sperm Motility: A Randomized Controlled Trial. *Annals of Surgery* 2010; **252**: 240–6.

41 Kawai M, Tani M, Hirono S, *et al.* Pylorus Ring Resection Reduces Delayed Gastric Emptying in Patients Undergoing Pancreatoduodenectomy: A Prospective, Randomized, Controlled Trial of Pylorus-Resecting Versus Pylorus-Preserving Pancreatoduodenectomy. *Annals of Surgery* 2011; **253**: 495–501.

42 Ma J, Cassera MA, Spaun GO, Hammill CW, Hansen PD, Aliabadi-Wahle S. Randomized Controlled Trial Comparing Single-Port Laparoscopic Cholecystectomy and Four-Port Laparoscopic Cholecystectomy: *Annals of Surgery* 2011; **254**: 22–7.

43 Chen J-S, Hsu H-H, Huang P-M, *et al.* Thoracoscopic Pleurodesis for Primary Spontaneous Pneumothorax With High Recurrence Risk: A Prospective Randomized Trial. *Annals of Surgery* 2012; **255**: 440–5.

44 Hamilton NA, Porembka MR, Johnston FM, *et al.* Mesh Reinforcement of Pancreatic Transection Decreases Incidence of Pancreatic Occlusion Failure for Left Pancreatectomy: A Single-Blinded, Randomized Controlled Trial. *Annals of Surgery* 2012; **255**: 1037–42.

45 Löffler T, Rossion I, Bruckner T, *et al.* HAnd Suture Versus STApling for Closure of Loop Ileostomy (HASTA Trial): Results of a Multicenter Randomized Trial (DRKS00000040). *Annals of Surgery* 2012; **256**: 828–36.

46 Oberkofler CE, Rickenbacher A, Raptis DA, *et al.* A Multicenter Randomized Clinical Trial of Primary Anastomosis or Hartmann's Procedure for Perforated Left Colonic Diverticulitis With Purulent or Fecal Peritonitis: *Annals of Surgery* 2012; **256**: 819–27.

47 Sancho JJ, Prieto R, Dueñas JP, *et al.* A Randomized Trial of Hemithyroidectomy Versus Dunhill for the Surgical Management of Asymmetrical Multinodular Goiter: *Annals of Surgery* 2012; **256**: 846–52.

48 Teoh AYB, Chiu PWY, Wong TCL, *et al.* A Double-Blinded Randomized Controlled Trial of Laparoendoscopic Single-Site Access Versus Conventional 3-Port Appendectomy: *Annals of Surgery* 2012; **256**: 909–14.

49 Campanelli G, Pascual MH, Hoeferlin A, *et al.* Randomized, Controlled, Blinded Trial of Tisseel/Tissucol for Mesh Fixation in Patients Undergoing Lichtenstein Technique for Primary Inguinal Hernia Repair: Results of the TIMELI Trial. *Annals of Surgery* 2012; **255**: 650–7.

50 Tzovaras G, Baloyiannis I, Zachari E, *et al.* Laparoendoscopic Rendezvous Versus Preoperative ERCP and Laparoscopic Cholecystectomy for the Management of Cholecysto-Choledocholithiasis: Interim Analysis of a Controlled Randomized Trial. *Annals of Surgery* 2012; **255**: 435–9.

© 2021 Robinson NB et al. *JAMA Network Open*.

51 Uzunoglu FG, Stehr A, Fink JA, *et al.* Ultrasonic Dissection Versus Conventional Dissection Techniques in Pancreatic Surgery: A Randomized Multicentre Study. *Annals of Surgery* 2012; **256**: 675–80.

52 Gutt CN, Encke J, Köninger J, *et al.* Acute Cholecystitis: Early Versus Delayed Cholecystectomy, A Multicenter Randomized Trial (ACDC Study, NCT00447304). *Annals of Surgery* 2013; **258**: 385–93.

53 Boelens OB, van Assen T, Houterman S, Scheltinga MR, Roumen RM. A Double-Blind, Randomized, Controlled Trial on Surgery for Chronic Abdominal Pain Due to Anterior Cutaneous Nerve Entrapment Syndrome: *Annals of Surgery* 2013; **257**: 845–9.

54 Frutos MD, Abrisqueta J, Lujan J, Abellan I, Parrilla P. Randomized Prospective Study to Compare Laparoscopic Appendectomy Versus Umbilical Single-Incision Appendectomy: *Annals of Surgery* 2013; **257**: 413–8.

55 Lee W-S, Choi ST, Lee JN, *et al.* Single-Port Laparoscopic Appendectomy Versus Conventional Laparoscopic Appendectomy: A Prospective Randomized Controlled Study. *Annals of Surgery* 2013; **257**: 214–8.

56 López-Andújar R, Orón EM, Carregnato AF, *et al.* T-tube or No T-tube in Cadaveric Orthotopic Liver Transplantation: The Eternal Dilemma. *Annals of Surgery* 2013; **258**: 21–9.

57 Rogmark P, Petersson U, Bringman S, *et al.* Short-term Outcomes for Open and Laparoscopic Midline Incisional Hernia Repair: A Randomized Multicenter Controlled Trial. *Annals of Surgery* 2013; **258**: 37–45.

58 Dupré A, Lefranc A, Buc E, *et al.* Use of Bioresorbable Membranes to Reduce Abdominal and Perihepatic Adhesions in 2-Stage Hepatectomy of Liver Metastases From Colorectal Cancer: Results of a Prospective, Randomized Controlled Phase II Trial. *Annals of Surgery* 2013; **258**: 30–6.

59 Shimoda M, Kubota K, Katoh M, Kita J. Effect of Billroth II or Roux-en-Y Reconstruction for the Gastrojejunostomy on Delayed Gastric Emptying After Pancreaticoduodenectomy: A Randomized Controlled Study. *Annals of Surgery* 2013; **257**: 938–42.

60 Peterli R, Borbély Y, Kern B, *et al.* Early Results of the Swiss Multicentre Bypass or Sleeve Study (SM-BOSS): A Prospective Randomized Trial Comparing Laparoscopic Sleeve Gastrectomy and Roux-en-Y Gastric Bypass. *Annals of Surgery* 2013; **258**: 690–5.

61 Eshuis WJ, van Eijck CHJ, Gerhards MF, *et al.* Antecolic Versus Retrocolic Route of the Gastroenteric Anastomosis After Pancreatoduodenectomy: A Randomized Controlled Trial. *Annals of Surgery* 2014; **259**: 45–51.

62 Barczyński M, Konturek A, Nowak W. Randomized Clinical Trial of Posterior Retroperitoneoscopic Adrenalectomy Versus Lateral Transperitoneal Laparoscopic Adrenalectomy With a 5-Year Follow-up: *Annals of Surgery* 2014; **260**: 740–8.

63 Denost Q, Adam J-P, Rullier A, Buscail E, Laurent C, Rullier E. Perineal Transanal Approach: A New Standard for Laparoscopic Sphincter-Saving Resection in Low Rectal Cancer, a Randomized Trial. *Annals of Surgery* 2014; **260**: 993–9.

© 2021 Robinson NB et al. *JAMA Network Open*.

64 Yamamoto S, Inomata M, Katayama H, *et al.* Short-Term Surgical Outcomes From a Randomized Controlled Trial to Evaluate Laparoscopic and Open D3 Dissection for Stage II/III Colon Cancer: Japan Clinical Oncology Group Study JCOG 0404. *Annals of Surgery* 2014; **260**: 23–30.

65 Melissa CS, Bun TAY, Wing CK, Chung TY, Wai NEK, Tat LH. Randomized Double-Blinded Prospective Trial of Fibrin Sealant Spray Versus Mechanical Stapling in Laparoscopic Total Extraperitoneal Hernioplasty: *Annals of Surgery* 2014; **259**: 432–7.

66 Nicol AJ, Navsaria PH, Hommes M, Ball CG, Edu S, Kahn D. Sternotomy or Drainage for a Hemopericardium After Penetrating Trauma: A Randomized Controlled Trial. *Annals of Surgery* 2014; **259**: 438–42.

67 Van Buren G, Bloomston M, Hughes SJ, *et al.* A Randomized Prospective Multicenter Trial of Pancreaticoduodenectomy With and Without Routine Intraperitoneal Drainage: *Annals of Surgery* 2014; **259**: 605–12.

68 Rönkä K, Vironen J, Kössi J, *et al.* Randomized Multicenter Trial Comparing Glue Fixation, Self-gripping Mesh, and Suture Fixation of Mesh in Lichtenstein Hernia Repair (FinnMesh Study): *Annals of Surgery* 2015; **262**: 714–20.

69 García-Ureña MÁ, López-Monclús J, Hernando LAB, *et al.* Randomized Controlled Trial of the Use of a Large-pore Polypropylene Mesh to Prevent Incisional Hernia in Colorectal Surgery: *Annals of Surgery* 2015; **261**: 876–81.

70 Müller-Stich BP, Linke GR, Senft J, *et al.* Laparoscopic Mesh-augmented Hiatoplasty With Cardiophrenicopexy Versus Laparoscopic Nissen Fundoplication for the Treatment of Gastroesophageal Reflux Disease: A Double-center Randomized Controlled Trial. *Annals of Surgery* 2015; **262**: 721–7.

71 Bulian DR, Knuth J, Cerasani N, Sauerwald A, Lefering R, Heiss MM. Transvaginal/Transumbilical Hybrid—NOTES—Versus 3-Trocar Needlescopic Cholecystectomy: Short-term Results of a Randomized Clinical Trial. *Annals of Surgery* 2015; **261**: 451–8.

72 Timmermans L, Eker HH, Steyerberg EW, *et al.* Short-term Results of a Randomized Controlled Trial Comparing Primary Suture With Primary Glued Mesh Augmentation to Prevent Incisional Hernia: *Annals of Surgery* 2015; **261**: 276–81.

73 Lurje G, Raptis DA, Steinemann DC, *et al.* Cosmesis and Body Image in Patients Undergoing Single-port Versus Conventional Laparoscopic Cholecystectomy: A Multicenter Double-blinded Randomized Controlled Trial (SPOCC-trial). *Annals of Surgery* 2015; **262**: 728–35.

74 Watson DI, Thompson SK, Devitt PG, *et al.* Laparoscopic Repair of Very Large Hiatus Hernia With Sutures Versus Absorbable Mesh Versus Nonabsorbable Mesh: A Randomized Controlled Trial. *Annals of Surgery* 2015; **261**: 282–9.

75 Le Coq B, Frering V, Ghunaim M, *et al.* Impact of Surgical Technique on Long-term Complication Rate After Laparoscopic Adjustable Gastric Banding (LAGB): Results of a Single-blinded Randomized Controlled trial (ANOSEAN Study). *Annals of Surgery* 2016; **264**: 738–44.

© 2021 Robinson NB et al. *JAMA Network Open*.

76 Angenete E, Thornell A, Burcharth J, *et al.* Laparoscopic Lavage Is Feasible and Safe for the Treatment of Perforated Diverticulitis With Purulent Peritonitis: The First Results From the Randomized Controlled Trial DILALA. *Annals of Surgery* 2016; **263**: 117–22.

77 Hassenpflug M, Hinz U, Strobel O, *et al.* Teres Ligament Patch Reduces Relevant Morbidity After Distal Pancreatectomy (the DISCOVER Randomized Controlled Trial): *Annals of Surgery* 2016; **264**: 723–30.

78 Han JG, Wang ZJ, Zheng Y, *et al.* Ligation of Intersphincteric Fistula Tract vs Ligation of the Intersphincteric Fistula Tract Plus a Bioprosthetic Anal Fistula Plug Procedure in Patients With Transsphincteric Anal Fistula: Early Results of a Multicenter Prospective Randomized Trial. *Annals of Surgery* 2016; **264**: 917–22.

79 Kawai M, Hirono S, Okada K, *et al.* Randomized Controlled Trial of Pancreaticojejunostomy versus Stapler Closure of the Pancreatic Stump During Distal Pancreatectomy to Reduce Pancreatic Fistula: *Annals of Surgery* 2016; **264**: 180–7.

80 Kim SM, Cho J, Kang D, *et al.* A Randomized Controlled Trial of Vagus Nerve-preserving Distal Gastrectomy Versus Conventional Distal Gastrectomy for Postoperative Quality of Life in Early Stage Gastric Cancer Patients: *Annals of Surgery* 2016; **263**: 1079–84.

81 Lehur PA, Didnée AS, Faucheron J-L, *et al.* Cost-effectiveness of New Surgical Treatments for Hemorrhoidal Disease: A Multicentre Randomized Controlled Trial Comparing Transanal Doppler-guided Hemorrhoidal Artery Ligation With Mucopexy and Circular Stapled Hemorrhoidopexy. *Annals of Surgery* 2016; **264**: 710–6.

82 López-Cano M, Serra-Aracils X, Mora L, *et al.* Preventing Parastomal Hernia Using a Modified Sugarbaker Technique With Composite Mesh During Laparoscopic Abdominoperineal Resection: A Randomized Controlled Trial. *Annals of Surgery* 2016; **264**: 923–8.

83 Muysoms FE, Detry O, Vierendeels T, *et al.* Prevention of Incisional Hernias by Prophylactic Mesh-augmented Reinforcement of Midline Laparotomies for Abdominal Aortic Aneurysm Treatment: A Randomized Controlled Trial. *Annals of Surgery* 2016; **263**: 638–45.

84 Keck T, Wellner UF, Bahra M, *et al.* Pancreatogastrostomy Versus Pancreatojejunostomy for RECOnstruction After PANCreatoduodenectomy (RECOPANC, DRKS 00000767): Perioperative and Long-term Results of a Multicenter Randomized Controlled Trial. *Annals of Surgery* 2016; **263**: 440–9.

85 Roulin D, Saadi A, Di Mare L, Demartines N, Halkic N. Early Versus Delayed Cholecystectomy for Acute Cholecystitis, Are the 72 hours Still the Rule?: A Randomized Trial. *Annals of Surgery* 2016; **264**: 717–22.

86 Schlosser K, Bartsch DK, Diener MK, *et al.* Total Parathyroidectomy With Routine Thymectomy and Autotransplantation Versus Total Parathyroidectomy Alone for Secondary Hyperparathyroidism: Results of a Nonconfirmatory Multicenter Prospective Randomized Controlled Pilot Trial. *Annals of Surgery* 2016; **264**: 745–53.


© 2021 Robinson NB et al. *JAMA Network Open*.

87 Westin L, Wollert S, Ljungdahl M, Sandblom G, Gunnarsson U, Dahlstrand U. Less Pain 1 Year After Total Extra-peritoneal Repair Compared With Lichtenstein Using Local Anesthesia: Data From a Randomized Controlled Clinical Trial. *Annals of Surgery* 2016; **263**: 240–3.

88 Musters GD, Klaver CEL, Bosker RJI, *et al.* Biological Mesh Closure of the Pelvic Floor After Extralevator Abdominoperineal Resection for Rectal Cancer: A Multicenter Randomized Controlled Trial (the BIOPEX-study). *Annals of Surgery* 2017; **265**: 1074–81.

89 Danielsen AK, Park J, Jansen JE, *et al.* Early Closure of a Temporary Ileostomy in Patients With Rectal Cancer: A Multicenter Randomized Controlled Trial. *Annals of Surgery* 2017; **265**: 284–90.

90 Alifano M, Jayle C, Bertin F, *et al.* Medical and Economic Evaluation of FOREseal Bioabsorbable Reinforcement Sleeves Compared With Current Standard of Care for Reducing Air Leakage Duration After Lung Resection for Malignancy: A Randomized Trial. *Annals of Surgery* 2017; **265**: 45–53.

91 Molegraaf MJ, Grotenhuis B, Torensma B, de Ridder V, Lange JF, Swank DJ. The HIPPO Trial, a Randomized Double-blind Trial Comparing Self-gripping Parietex Progrip Mesh and Sutured Parietex Mesh in Lichtenstein Hernioplasty: A Long-term Follow-up Study. *Annals of Surgery* 2017; **266**: 939–45.

92 Langhans L, Tvedskov TF, Klausen TL, *et al.* Radioactive Seed Localization or Wire-guided Localization of Nonpalpable Invasive and In Situ Breast Cancer: A Randomized, Multicenter, Open-label Trial. *Annals of Surgery* 2017; **266**: 29–35.

93 Brandsma H-T, Hansson BME, Aufenacker TJ, *et al.* Prophylactic Mesh Placement During Formation of an End-colostomy Reduces the Rate of Parastomal Hernia: Short-term Results of the Dutch PREVENT-trial. *Annals of Surgery* 2017; **265**: 663–9.

94 Sano T, Sasako M, Mizusawa J, *et al.* Randomized Controlled Trial to Evaluate Splenectomy in Total Gastrectomy for Proximal Gastric Carcinoma: *Annals of Surgery* 2017; **265**: 277–83.

95 Park YK, Yoon HM, Kim Y-W, *et al.* Laparoscopy-assisted versus Open D2 Distal Gastrectomy for Advanced Gastric Cancer: Results From a Randomized Phase II Multicenter Clinical Trial (COACT 1001). *Annals of Surgery* 2018; **267**: 638–45.

96 Mari GM, Crippa J, Cocozza E, *et al.* Low Ligation of Inferior Mesenteric Artery in Laparoscopic Anterior Resection for Rectal Cancer Reduces Genitourinary Dysfunction: Results From a Randomized Controlled Trial (HIGHLOW Trial). *Annals of Surgery* 2019; **269**: 1018–24.

97 Ielpo B, Nuñez-Alfonsel J, Duran H, *et al.* Cost-effectiveness of Randomized Study of Laparoscopic Versus Open Bilateral Inguinal Hernia Repair: *Annals of Surgery* 2018; **268**: 725–30.

98 Kim MJ, Park SC, Park JW, *et al.* Robot-assisted Versus Laparoscopic Surgery for Rectal Cancer: A Phase II Open Label Prospective Randomized Controlled Trial. *Annals of Surgery* 2018; **267**: 243–51.

99 Lee L, Mata J, Droeser RA, *et al.* Incisional Hernia After Midline Versus Transverse Specimen Extraction Incision: A Randomized Trial in Patients Undergoing Laparoscopic Colectomy. *Annals of Surgery* 2018; **268**: 41–7.

© 2021 Robinson NB et al. *JAMA Network Open*.

100 Sandström P, Røsok BI, Sparrelid E, *et al.* ALPPS Improves Resectability Compared With Conventional Two-stage Hepatectomy in Patients With Advanced Colorectal Liver Metastasis: Results From a Scandinavian Multicenter Randomized Controlled Trial (LIGRO Trial). *Annals of Surgery* 2018; **267**: 833–40.

101 Maggiori L, Tuech JJ, Cotte E, *et al.* Single-incision Laparoscopy Versus Multiport Laparoscopy for Colonic Surgery: A Multicenter, Double-blinded, Randomized Controlled Trial. *Annals of Surgery* 2018; **268**: 740–6.

102 Fretland ÅA, Dagenborg VJ, Bjørnelv GMW, *et al.* Laparoscopic Versus Open Resection for Colorectal Liver Metastases: The OSLO-COMET Randomized Controlled Trial. *Annals of Surgery* 2018; **267**: 199–207.

103 Poves I, Burdío F, Morató O, *et al.* Comparison of Perioperative Outcomes Between Laparoscopic and Open Approach for Pancreatoduodenectomy: The PADULAP Randomized Controlled Trial. *Annals of Surgery* 2018; **268**: 731–9.

104 Hackert T, Probst P, Knebel P, *et al.* Pylorus Resection Does Not Reduce Delayed Gastric Emptying After Partial Pancreatoduodenectomy: A Blinded Randomized Controlled Trial (PROPP Study, DRKS00004191). *Annals of Surgery* 2018; **267**: 1021–7.

105 Siribumrungwong B, Chantip A, Noorit P, *et al.* Comparison of Superficial Surgical Site Infection Between Delayed Primary Versus Primary Wound Closure in Complicated Appendicitis: A Randomized Controlled Trial. *Annals of Surgery* 2018; **267**: 631–7.

106 So JB-Y, Rao J, Wong AS-Y, *et al.* Roux-en-Y or Billroth II Reconstruction After Radical Distal Gastrectomy for Gastric Cancer: A Multicenter Randomized Controlled Trial. *Annals of Surgery* 2018; **267**: 236–42.

107 Rebibo L, Dhahri A, Chati R, Cosse C, Huet E, Regimbeau J-M. Effectiveness of Fibrin Sealant Application on the Development of Staple Line Complications After Sleeve Gastrectomy: A Prospective Randomized Trial. *Annals of Surgery* 2018; **268**: 762–8.

108 Allaix ME, Degiuli M, Bonino MA, *et al.* Intracorporeal or Extracorporeal Ileocolic Anastomosis After Laparoscopic Right Colectomy: A Double-blinded Randomized Controlled Trial. *Annals of Surgery* 2019; **270**: 762–7.

109 Chai YJ, Yu HW, Song R-Y, Kim S, Choi JY, Lee KE. Lateral Transperitoneal Adrenalectomy Versus Posterior Retroperitoneoscopic Adrenalectomy for Benign Adrenal Gland Disease: Randomized Controlled Trial at a Single Tertiary Medical Center. *Annals of Surgery* 2019; **269**: 842–8.

110 Dumanian GA, Potter BK, Mioton LM, *et al.* Targeted Muscle Reinnervation Treats Neuroma and Phantom Pain in Major Limb Amputees: A Randomized Clinical Trial. *Annals of Surgery* 2019; **270**: 238–46.

111 Dupont E, Tsangaris T, Garcia-Cantu C, *et al.* Resection of Cavity Shave Margins in Stage 0–III Breast Cancer Patients Undergoing Breast Conserving Surgery: A Prospective Multicenter Randomized Controlled Trial. *Annals of Surgery* 2019; **Publish Ahead of Print**. DOI:10.1097/SLA.0000000000003449.

© 2021 Robinson NB et al. *JAMA Network Open*.

112 Hirono S, Kawai M, Okada K-I, *et al.* Modified Blumgart Mattress Suture Versus Conventional Interrupted Suture in Pancreaticojejunostomy During Pancreaticoduodenectomy: Randomized Controlled Trial. *Annals of Surgery* 2019; **269**: 243–51.

113 de Rooij T, van Hilst J, van Santvoort H, *et al.* Minimally Invasive Versus Open Distal Pancreatectomy (LEOPARD): A Multicenter Patient-blinded Randomized Controlled Trial. *Annals of Surgery* 2019; **269**: 2–9.

114 Lohmander F, Lagergren J, Roy PG, *et al.* Implant Based Breast Reconstruction With Acellular Dermal Matrix: Safety Data From an Open-label, Multicenter, Randomized, Controlled Trial in the Setting of Breast Cancer Treatment. *Annals of Surgery* 2019; **269**: 836–41.

115 Parc Y, Ruppert R, Fuerst A, *et al.* Better Function With a Colonic J-Pouch or a Side-to-end Anastomosis?: A Randomized Controlled Trial to Compare the Complications, Functional Outcome, and Quality of Life in Patients With Low Rectal Cancer After a J-Pouch or a Side-to-end Anastomosis. *Annals of Surgery* 2019; **269**: 815–26.

116 van der Sluis PC, van der Horst Sylvia, May AM, *et al.* Robot-assisted Minimally Invasive Thoracolaparoscopic Esophagectomy Versus Open Transthoracic Esophagectomy for Resectable Esophageal Cancer: A Randomized Controlled Trial. *Annals of Surgery* 2019; **269**: 621–30.

117 Sabater L, Cugat E, Serrablo A, *et al.* Does the Artery-first Approach Improve the Rate of R0 Resection in Pancreatoduodenectomy?: A Multicenter, Randomized, Controlled Trial. *Annals of Surgery* 2019; **270**: 738–46.

118 Marti WR, Curti G, Wehrli H, *et al.* Clinical Outcome After Rectal Replacement With Side-to-End, Colon-J-Pouch, or Straight Colorectal Anastomosis Following Total Mesorectal Excision: A Swiss Prospective, Randomized, Multicenter Trial (SAKK 40/04). *Annals of Surgery* 2019; **269**: 827–35.

119 Odensten C, Strigård K, Rutegård J, *et al.* Use of Prophylactic Mesh When Creating a Colostomy Does Not Prevent Parastomal Hernia: A Randomized Controlled Trial—STOMAMESH. *Annals of Surgery* 2019; **269**: 427–31.

120 Maurer E, Maschuw K, Reuss A, *et al.* Total Versus Near-total Thyroidectomy in Graves Disease: Results of the Randomized Controlled Multicenter TONIG-trial. *Annals of Surgery* 2019; **270**: 755–61.

121 Cerfolio RJ, Bryant AS, Maniscalco LM. A Nondivided Intercostal Muscle Flap Further Reduces Pain of Thoracotomy: A Prospective Randomized Trial. *The Annals of Thoracic Surgery* 2008; **85**: 1901–7.

122 Li X, Tu Y-R, Lin M, Lai F-C, Chen J-F, Dai Z-J. Endoscopic Thoracic Sympathectomy for Palmar Hyperhidrosis: A Randomized Control Trial Comparing T3 and T2-4 Ablation. *The Annals of Thoracic Surgery* 2008; **85**: 1747–51.

123 Schimmer C, Reents W, Berneder S, *et al.* Prevention of Sternal Dehiscence and Infection in High-Risk Patients: A Prospective Randomized Multicenter Trial. *The Annals of Thoracic Surgery* 2008; **86**: 1897–904.

© 2021 Robinson NB et al. *JAMA Network Open*.

124 Krishnamoorthy B, Najam O, Khan UA, Waterworth P, Fildes JE, Yonan N. Randomized Prospective Study Comparing Conventional Subcuticular Skin Closure With Dermabond Skin Glue After Saphenous Vein Harvesting. *The Annals of Thoracic Surgery* 2009; **88**: 1445–9.

125 Suri RM, Zehr KJ, Sundt TM, *et al.* Left Ventricular Mass Regression After Porcine Versus Bovine Aortic Valve Replacement: A Randomized Comparison. *The Annals of Thoracic Surgery* 2009; **88**: 1232–7.

126 Allama AM. Intercostal Muscle Flap for Decreasing Pain After Thoracotomy: A Prospective Randomized Trial. *The Annals of Thoracic Surgery* 2010; **89**: 195–9.

127 Yousefnia MA, Dehestani A, Saidi B, Roshanali F, Mandegar MH, Alaeddini F. Papillary Muscle Repositioning in Valve Replacement for Left Ventricular Dysfunction: Ischemic Mitral Regurgitation. *The Annals of Thoracic Surgery* 2010; **90**: 497–502.

128 Aykut K, Celik B, Acıkel U. Figure-of-Eight Versus Prophylactic Sternal Weave Closure of Median Sternotomy in Diabetic Obese Patients Undergoing Coronary Artery Bypass Grafting. *The Annals of Thoracic Surgery* 2011; **92**: 638–41.

129 Baumgartner FJ, Reyes M, Sarkisyan GG, Iglesias A, Reyes E. Thoracoscopic Sympathicotomy for Disabling Palmar Hyperhidrosis: A Prospective Randomized Comparison Between Two Levels. *The Annals of Thoracic Surgery* 2011; **92**: 2015–9.

130 Lai F-C, Chen L, Tu Y-R, Lin M, Li X. Prevention of Chylothorax Complicating Extensive Esophageal Resection by Mass Ligation of Thoracic Duct: A Random Control Study. *The Annals of Thoracic Surgery* 2011; **91**: 1770–4.

131 Mannacio V, Di Tommaso L, De Amicis V, Stassano P, Vosa C. Randomized Flow Capacity Comparison of Skeletonized and Pedicled Left Internal Mammary Artery. *The Annals of Thoracic Surgery* 2011; **91**: 24–30.

132 Aye RW, Swanstrom LL, Kapur S, *et al.* A Randomized Multiinstitution Comparison of the Laparoscopic Nissen and Hill Repairs. *The Annals of Thoracic Surgery* 2012; **94**: 951–8.

133 Licht PB, Pilegaard HK, Ladegaard L. Sympathicotomy for Isolated Facial Blushing: A Randomized Clinical Trial. *The Annals of Thoracic Surgery* 2012; **94**: 401–5.

134 Raman J, Lehmann S, Zehr K, *et al.* Sternal Closure With Rigid Plate Fixation Versus Wire Closure: A Randomized Controlled Multicenter Trial. *The Annals of Thoracic Surgery* 2012; **94**: 1854–61.

135 Birla R, Twine G, Unsworth-White J. Randomized Trial of Carpentier-Edwards Supraannular Prosthesis Versus Mosaic Aortic Prosthesis: 6 Year Results. *The Annals of Thoracic Surgery* 2013; **95**: 831–7.

136 Dreifaldt M, Mannion JD, Bodin L, Olsson H, Zagozdzon L, Souza D. The no-touch saphenous vein as the preferred second conduit for coronary artery bypass grafting. *Ann Thorac Surg* 2013; **96**: 105–11.

© 2021 Robinson NB et al. *JAMA Network Open*.

137 Bolotin G, Huber CH, Shani L, *et al.* Novel Emboli Protection System During Cardiac Surgery: A Multi-Center, Randomized, Clinical Trial. *The Annals of Thoracic Surgery* 2014; **98**: 1627–34.

138 Lee S, Kim HR, Cho S, *et al.* Staple Line Coverage After Bullectomy for Primary Spontaneous Pneumothorax: A Randomized Trial. *The Annals of Thoracic Surgery* 2014; **98**: 2005–11.

139 Min X, Huang Y, Yang Y, *et al.* Mechanical Pleurodesis Does Not Reduce Recurrence of Spontaneous Pneumothorax: A Randomized Trial. *The Annals of Thoracic Surgery* 2014; **98**: 1790–6.

140 Borger MA, Moustafine V, Conradi L, *et al.* A Randomized Multicenter Trial of Minimally Invasive Rapid Deployment Versus Conventional Full Sternotomy Aortic Valve Replacement. *The Annals of Thoracic Surgery* 2015; **99**: 17–25.

141 Taggart DP, Ben Gal Y, Lees B, *et al.* A Randomized Trial of External Stenting for Saphenous Vein Grafts in Coronary Artery Bypass Grafting. *The Annals of Thoracic Surgery* 2015; **99**: 2039–45.

142 Baumbach H, Rustenbach CJ, Ahad S, *et al.* Minimally Invasive Extracorporeal Bypass in Minimally Invasive Heart Valve Operations: A Prospective Randomized Trial. *The Annals of Thoracic Surgery* 2016; **102**: 93–100.

143 Nguyen BAV, Fiorentino F, Reeves BC, *et al.* Mini Bypass and Proinflammatory Leukocyte Activation: A Randomized Controlled Trial. *The Annals of Thoracic Surgery* 2016; **101**: 1454–63.

144 Fiorelli A, Messina G, Chiodini P, *et al.* Cardiac Autonomic Changes After Thoracic Sympathectomy: A Prospective, Randomized Study. *The Annals of Thoracic Surgery* 2017; **103**: 216–24.

145 Okami J, Tokunaga T, Kanou T, *et al.* Randomized Study Comparing Equal Height Staples With Graduated Height Staples in Bronchial Closure. *The Annals of Thoracic Surgery* 2017; **104**: 1012–9.

146 Pettersen Ø, Haram PM, Winnerkvist A, *et al.* Pedicled Vein Grafts in Coronary Surgery: Perioperative Data From a Randomized Trial. *Ann Thorac Surg* 2017; **104**: 1313–7.

147 Etiwy M, Javadikasgari H, Houghtaling P, Gillinov M. Automated Titanium Fasteners Versus Hand-Tied Knots: A Randomized Controlled Trial. *The Annals of Thoracic Surgery* 2018; **106**: 1160–3.

148 Long H, Tan Q, Luo Q, *et al.* Thoracoscopic Surgery Versus Thoracotomy for Lung Cancer: Short-Term Outcomes of a Randomized Trial. *The Annals of Thoracic Surgery* 2018; **105**: 386–92.

149 Salim EF, Ali GA. Impact of Thoracoscopic T2 Sympathectomy on Patients With Primary Palmar and Axillary Hyperhidrosis. *The Annals of Thoracic Surgery* 2018; **106**: 1032–7.

150 Halfwerk FR, Knol K, Mariani S, Grandjean JG, Mecozzi G. Randomized Trial of Miniaturized Versus Standard Extracorporeal Circulation in Aortic Valve Surgery. *The Annals of Thoracic Surgery* 2019; **108**: 37–44.

151 Jeong DS, Sung K, Kim WS, Keumhee Cho C, Park PW. Randomized Trial of Concomitant Maze Procedure Using Nitrous Oxide- Versus Argon-Based Cryoablation. *The Annals of Thoracic Surgery* 2019; **108**: 30–6.

© 2021 Robinson NB et al. *JAMA Network Open*.

152 Leshnower BG, Rangaraju S, Allen JW, Stringer AY, Gleason TG, Chen EP. Deep Hypothermia With Retrograde Cerebral Perfusion Versus Moderate Hypothermia With Antegrade Cerebral Perfusion for Arch Surgery. *The Annals of Thoracic Surgery* 2019; **107**: 1104–10.

153 Ferretti A, Vadalà A, De Carli A, Argento G, Conteduca F, Severini G. Minimizing Internal Rotation Strength Deficit After Use of Semitendinosus for Anterior Cruciate Ligament Reconstruction: A Modified Harvesting Technique. *Arthroscopy: The Journal of Arthroscopic & Related Surgery* 2008; **24**: 786–95.

154 Järvelä T, Moisala A-S, Paakkala T, Paakkala A. Tunnel Enlargement After Double-Bundle Anterior Cruciate Ligament Reconstruction: A Prospective, Randomized Study. *Arthroscopy: The Journal of Arthroscopic & Related Surgery* 2008; **24**: 1349–57.

155 Monteiro GC, Ejnisman B, Andreoli CV, Pochini AC, Cohen M. Absorbable Versus Nonabsorbable Sutures for the Arthroscopic Treatment of Anterior Shoulder Instability in Athletes: A Prospective Randomized Study. *Arthroscopy: The Journal of Arthroscopic & Related Surgery* 2008; **24**: 697–703.

156 Myers P, Logan M, Stokes A, Boyd K, Watts M. Bioabsorbable Versus Titanium Interference Screws With Hamstring Autograft in Anterior Cruciate Ligament Reconstruction: A Prospective Randomized Trial With 2-Year Follow-up. *Arthroscopy: The Journal of Arthroscopic & Related Surgery* 2008; **24**: 817–23.

157 Siebold R, Dehler C, Ellert T. Prospective Randomized Comparison of Double-Bundle Versus Single-Bundle Anterior Cruciate Ligament Reconstruction. *Arthroscopy: The Journal of Arthroscopic & Related Surgery* 2008; **24**: 137–45.

158 Grasso A, Milano G, Salvatore M, Falcone G, Deriu L, Fabbriciani C. Single-Row Versus Double-Row Arthroscopic Rotator Cuff Repair: A Prospective Randomized Clinical Study. *Arthroscopy: The Journal of Arthroscopic & Related Surgery* 2009; **25**: 4–12.

159 Papp M, Szabó L, Lázár I, *et al.* Combined High Tibial Osteotomy Decreases Biomechanical Changes Radiologically Detectable in the Sagittal Plane Compared With Closing-Wedge Osteotomy. *Arthroscopy: The Journal of Arthroscopic & Related Surgery* 2009; **25**: 355–64.

160 Chen J, Chen S, Li Y, Hua Y, Li H. Is the Extended Release of the Inferior Glenohumeral Ligament Necessary for Frozen Shoulder? *Arthroscopy: The Journal of Arthroscopic & Related Surgery* 2010; **26**: 529–35.

161 Milano G, Grasso A, Salvatore M, Saccomanno MF, Deriu L, Fabbriciani C. Arthroscopic Rotator Cuff Repair With Metal and Biodegradable Suture Anchors: A Prospective Randomized Study. *Arthroscopy: The Journal of Arthroscopic & Related Surgery* 2010; **26**: S112–9.

162 Koh KH, Kang KC, Lim TK, Shon MS, Yoo JC. Prospective Randomized Clinical Trial of Single- Versus Double-Row Suture Anchor Repair in 2- to 4-cm Rotator Cuff Tears: Clinical and Magnetic Resonance Imaging Results. *Arthroscopy: The Journal of Arthroscopic & Related Surgery* 2011; **27**: 453–62.

163 Sabat D, Kundu K, Arora S, Kumar V. Tunnel widening after anterior cruciate ligament reconstruction: a prospective randomized computed tomography--based study comparing 2 different femoral fixation methods for hamstring graft. *Arthroscopy* 2011; **27**: 776–83.

© 2021 Robinson NB et al. *JAMA Network Open*.

164 Wipfler B, Donner S, Zechmann CM, Springer J, Siebold R, Paessler HH. Anterior Cruciate Ligament Reconstruction Using Patellar Tendon Versus Hamstring Tendon: A Prospective Comparative Study With 9-Year Follow-Up. *Arthroscopy: The Journal of Arthroscopic & Related Surgery* 2011; **27**: 653–65.

165 Kim JG, Wang JH, Lim HC, Ahn JH. Femoral Graft Bending Angle and Femoral Tunnel Geometry of Transportal and Outside-In Techniques in Anterior Cruciate Ligament Reconstruction: An In Vivo 3-Dimensional Computed Tomography Analysis. *Arthroscopy: The Journal of Arthroscopic & Related Surgery* 2012; **28**: 1682–94.

166 Lawhorn KW, Howell SM, Traina SM, Gottlieb JE, Meade TD, Freedberg HI. The Effect of Graft Tissue on Anterior Cruciate Ligament Outcomes: A Multicenter, Prospective, Randomized Controlled Trial Comparing Autograft Hamstrings With Fresh-Frozen Anterior Tibialis Allograft. *Arthroscopy: The Journal of Arthroscopic & Related Surgery* 2012; **28**: 1079–86.

167 Lee S, Kim H, Jang J, Seong SC, Lee MC. Intraoperative Correlation Analysis Between Tunnel Position and Translational and Rotational Stability in Single- and Double-Bundle Anterior Cruciate Ligament Reconstruction. *Arthroscopy: The Journal of Arthroscopic & Related Surgery* 2012; **28**: 1424–36.

168 Ma H-L, Chiang E-R, Wu H-TH, *et al.* Clinical Outcome and Imaging of Arthroscopic Single-Row and Double-Row Rotator Cuff Repair: A Prospective Randomized Trial. *Arthroscopy: The Journal of Arthroscopic & Related Surgery* 2012; **28**: 16–24.

169 Shin S-J. A Comparison of 2 Repair Techniques for Partial-Thickness Articular-Sided Rotator Cuff Tears. *Arthroscopy: The Journal of Arthroscopic & Related Surgery* 2012; **28**: 25–33.

170 Shin S-J, Oh JH, Chung SW, Song MH. The Efficacy of Acromioplasty in the Arthroscopic Repair of Small- to Medium-Sized Rotator Cuff Tears Without Acromial Spur: Prospective Comparative Study. *Arthroscopy: The Journal of Arthroscopic & Related Surgery* 2012; **28**: 628–35.

171 Bourke HE, Salmon LJ, Waller A, *et al.* Randomized Controlled Trial of Osteoconductive Fixation Screws for Anterior Cruciate Ligament Reconstruction: A Comparison of the Calaxo and Milagro Screws. *Arthroscopy: The Journal of Arthroscopic & Related Surgery* 2013; **29**: 74–82.

172 Gudas R, Gudaitė A, Mickevičius T, *et al.* Comparison of Osteochondral Autologous Transplantation, Microfracture, or Debridement Techniques in Articular Cartilage Lesions Associated With Anterior Cruciate Ligament Injury: A Prospective Study With a 3-Year Follow-up. *Arthroscopy: The Journal of Arthroscopic & Related Surgery* 2013; **29**: 89–97.

173 Hwang D-H, Shetty GM, Kim JI, *et al.* Does Press-Fit Technique Reduce Tunnel Volume Enlargement After Anterior Cruciate Ligament Reconstruction With Autologous Hamstring Tendons? A Prospective Randomized Computed Tomography Study. *Arthroscopy: The Journal of Arthroscopic & Related Surgery* 2013; **29**: 83–8.

174 Krych AJ, Thompson M, Knutson Z, Scoon J, Coleman SH. Arthroscopic Labral Repair Versus Selective Labral Debridement in Female Patients With Femoroacetabular Impingement: A Prospective Randomized Study. *Arthroscopy: The Journal of Arthroscopic & Related Surgery* 2013; **29**: 46–53.

© 2021 Robinson NB et al. *JAMA Network Open*.

175 Lubowitz JH, Schwartzberg R, Smith P. Randomized Controlled Trial Comparing All-Inside Anterior Cruciate Ligament Reconstruction Technique With Anterior Cruciate Ligament Reconstruction With a Full Tibial Tunnel. *Arthroscopy: The Journal of Arthroscopic & Related Surgery* 2013; **29**: 1195–200.

176 Ma L-F, Wang F, Chen B-C, Wang C-H, Zhou J-W, Wang H-Y. Medial Retinaculum Plasty Versus Medial Patellofemoral Ligament Reconstruction for Recurrent Patellar Instability in Adults: A Randomized Controlled Trial. *Arthroscopy: The Journal of Arthroscopic & Related Surgery* 2013; **29**: 891–7.

177 Noh JH, Roh YH, Yang BG, Yi SR, Lee SY. Femoral Tunnel Position on Conventional Magnetic Resonance Imaging After Anterior Cruciate Ligament Reconstruction in Young Men: Transtibial Technique Versus Anteromedial Portal Technique. *Arthroscopy: The Journal of Arthroscopic & Related Surgery* 2013; **29**: 882–90.

178 Noh JH, Yang BG, Yi SR, Roh YH, Lee JS. Single-Bundle Anterior Cruciate Ligament Reconstruction in Active Young Men Using Bone-Tendon Achilles Allograft Versus Free Tendon Achilles Allograft. *Arthroscopy: The Journal of Arthroscopic & Related Surgery* 2013; **29**: 507–13.

179 Takeda Y, Iwame T, Takasago T, *et al.* Comparison of Tunnel Orientation Between Transtibial and Anteromedial Portal Techniques for Anatomic Double-Bundle Anterior Cruciate Ligament Reconstruction Using 3-Dimensional Computed Tomography. *Arthroscopy: The Journal of Arthroscopic & Related Surgery* 2013; **29**: 195–204.

180 Li Y, Li J, Wang J, Gao S, Zhang Y. Comparison of Single-Bundle and Double-Bundle Isolated Posterior Cruciate Ligament Reconstruction With Allograft: A Prospective, Randomized Study. *Arthroscopy: The Journal of Arthroscopic & Related Surgery* 2014; **30**: 695–700.

181 Matsumoto T, Kuroda R, Matsushita T, *et al.* Reduction of Tunnel Enlargement With Use of Autologous Ruptured Tissue in Anterior Cruciate Ligament Reconstruction: A Pilot Clinical Trial. *Arthroscopy: The Journal of Arthroscopic & Related Surgery* 2014; **30**: 468–74.

182 Clement ND, Watts AC, Phillips C, McBirnie JM. Short-Term Outcome After Arthroscopic Bursectomy Debridement of Rotator Cuff Calcific Tendonopathy With and Without Subacromial Decompression: A Prospective Randomized Controlled Trial. *Arthroscopy: The Journal of Arthroscopic & Related Surgery* 2015; **31**: 1680–7.

183 Dong J, Wang XF, Men X, *et al.* Surgical Treatment of Acute Grade III Medial Collateral Ligament Injury Combined With Anterior Cruciate Ligament Injury: Anatomic Ligament Repair Versus Triangular Ligament Reconstruction. *Arthroscopy: The Journal of Arthroscopic & Related Surgery* 2015; **31**: 1108–16.

184 Kim Y-S, Lee H-J, Bae S-H, Jin H, Song HS. Outcome Comparison Between in Situ Repair Versus Tear Completion Repair for Partial Thickness Rotator Cuff Tears. *Arthroscopy: The Journal of Arthroscopic & Related Surgery* 2015; **31**: 2191–8.

185 Kim Y-S, Lee H-J, Park I, Im J-H, Park K-S, Lee S-B. Are Delayed Operations Effective for Patients With Rotator Cuff Tears and Concomitant Stiffness? An Analysis of Immediate Versus Delayed Surgery on Outcomes. *Arthroscopy: The Journal of Arthroscopic & Related Surgery* 2015; **31**: 197–204.

© 2021 Robinson NB et al. *JAMA Network Open*.

186 Koga H, Muneta T, Yagishita K, *et al.* Mid- to Long-term Results of Single-Bundle Versus Double-Bundle Anterior Cruciate Ligament Reconstruction: Randomized Controlled Trial. *Arthroscopy: The Journal of Arthroscopic & Related Surgery* 2015; **31**: 69–76.

187 Lubowitz JH, Schwartzberg R, Smith P. Cortical Suspensory Button Versus Aperture Interference Screw Fixation for Knee Anterior Cruciate Ligament Soft-Tissue Allograft: A Prospective, Randomized Controlled Trial. *Arthroscopy: The Journal of Arthroscopic & Related Surgery* 2015; **31**: 1733–9.

188 Akamatsu Y, Kobayashi H, Kusayama Y, Kumagai K, Saito T. Comparative Study of Opening-Wedge High Tibial Osteotomy With and Without a Combined Computed Tomography–Based and Image-Free Navigation System. *Arthroscopy: The Journal of Arthroscopic & Related Surgery* 2016; **32**: 2072–81.

189 Barber FA. Triple-Loaded Single-Row Versus Suture-Bridge Double-Row Rotator Cuff Tendon Repair With Platelet-Rich Plasma Fibrin Membrane: A Randomized Controlled Trial. *Arthroscopy: The Journal of Arthroscopic & Related Surgery* 2016; **32**: 753–61.

190 Li J, Kong F, Gao X, Shen Y, Gao S. Prospective Randomized Comparison of Knee Stability and Proprioception for Posterior Cruciate Ligament Reconstruction With Autograft, Hybrid Graft, and γ-Irradiated Allograft. *Arthroscopy: The Journal of Arthroscopic & Related Surgery* 2016; **32**: 2548–55.

191 Oh JH, Lee YH, Kim SH, *et al.* Comparison of Treatments for Superior Labrum–Biceps Complex Lesions With Concomitant Rotator Cuff Repair: A Prospective, Randomized, Comparative Analysis of Debridement, Biceps Tenotomy, and Biceps Tenodesis. *Arthroscopy: The Journal of Arthroscopic & Related Surgery* 2016; **32**: 958–67.

192 Tian S, Wang Y, Wang B, *et al.* Anatomic Double-Bundle Anterior Cruciate Ligament Reconstruction With a Hamstring Tendon Autograft and Fresh-Frozen Allograft: A Prospective, Randomized, and Controlled Study. *Arthroscopy: The Journal of Arthroscopic & Related Surgery* 2016; **32**: 2521–31.

193 Gupta R, Bahadur R, Malhotra A, *et al.* Outcome of Hamstring Autograft With Preserved Insertions Compared With Free Hamstring Autograft in Anterior Cruciate Ligament Surgery at 2-Year Follow-up. *Arthroscopy: The Journal of Arthroscopic & Related Surgery* 2017; **33**: 2208–16.

194 Krishna L, Panjwani T, Mok YR, Lin Wong FK, Singh A, Toh SJ. Use of the 5-Strand Hamstring Autograft Technique in Increasing Graft Size in Anterior Cruciate Ligament Reconstruction. *Arthroscopy: The Journal of Arthroscopic & Related Surgery* 2018; **34**: 2633–40.

195 Nam J-H, Park S, Lee H-R, Kim SH. Outcomes After Limited or Extensive Bursectomy During Rotator Cuff Repair: Randomized Controlled Trial. *Arthroscopy: The Journal of Arthroscopic & Related Surgery* 2018; **34**: 3167–74.

196 Chiang E-R, Chen K-H, Chih-Chang Lin A, *et al.* Comparison of Tunnel Enlargement and Clinical Outcome Between Bioabsorbable Interference Screws and Cortical Button-Post Fixation in Arthroscopic Double-Bundle Anterior Cruciate Ligament Reconstruction: A Prospective, Randomized Study With a Minimum Follow-Up of 2 Years. *Arthroscopy: The Journal of Arthroscopic & Related Surgery* 2019; **35**: 544–51.

© 2021 Robinson NB et al. *JAMA Network Open*.

197 Kaminski R, Kulinski K, Kozar-Kaminska K, Wasko MK, Langner M, Pomianowski S. Repair Augmentation of Unstable, Complete Vertical Meniscal Tears With Bone Marrow Venting Procedure: A Prospective, Randomized, Double-Blind, Parallel-Group, Placebo-Controlled Study. *Arthroscopy: The Journal of Arthroscopic & Related Surgery* 2019; **35**: 1500-1508.e1.

198 Li J, Li Z, Wang K, Liu C, Wang Y, Wang H. Medial Patellofemoral Ligament Reconstruction: A Comparison of Single-Bundle Transpatellar Tunnel and Double-Anchor Anatomic Techniques for the Treatment of Recurrent Lateral Patellar Dislocation in Adults. *Arthroscopy: The Journal of Arthroscopic & Related Surgery* 2019; **35**: 845-854.e1.

199 Yamakado K. A Prospective Randomized Trial Comparing Suture Bridge and Medially Based Single-Row Rotator Cuff Repair in Medium-Sized Supraspinatus Tears. *Arthroscopy: The Journal of Arthroscopic & Related Surgery* 2019; **35**: 2803–13.

200 Mátyás L, Berry M, Menyhei G, *et al.* The Safety and Efficacy of a Paclitaxel-eluting Wrap for Preventing Peripheral Bypass Graft Stenosis: A 2-Year Controlled Randomized Prospective Clinical Study. *European Journal of Vascular and Endovascular Surgery* 2008; **35**: 715–22.

201 Meerwaldt R, Lansink KWW, Blomme AM, Fritschy WM. Prospective Randomized Study of Carotid Endarterectomy with Fluoropassiv™ Thin Wall Carotid Patch versus Venous Patch. *European Journal of Vascular and Endovascular Surgery* 2008; **36**: 45–52.

202 Scharn DM, Dirven M, Barendregt WB, Boll APM, Roelofs D, van der Vliet JA. Human Umbilical Vein versus Heparin-bonded Polyester for Femoro-popliteal Bypass: 5-year Results of a Prospective Randomized Multicentre Trial. *European Journal of Vascular and Endovascular Surgery* 2008; **35**: 61–7.

203 Stehr A, Scodacek D, Wustrack H, *et al.* Retrojugular versus ventrojugular approach to carotid bifurcation for eversion endarterectomy: a prospective randomized trial. *Eur J Vasc Endovasc Surg* 2008; **35**: 190–5; discussion 196-197.

204 Gisbertz SS, Ramzan M, Tutein Nolthenius RP, *et al.* Short-Term Results of A Randomized Trial Comparing Remote Endarterectomy and Supragenicular Bypass Surgery for Long Occlusions of the Superficial Femoral Artery [The REVAS Trial]. *European Journal of Vascular and Endovascular Surgery* 2009; **37**: 68–76.

205 van Det RJ, Vriens BHR, van der Palen J, Geelkerken RH. Dacron or ePTFE for Femoro-popliteal Above-Knee Bypass Grafting: Short- and Long-term Results of a Multicentre Randomised Trial. *European Journal of Vascular and Endovascular Surgery* 2009; **37**: 457–63.

206 SCAMICOS. PTFE bypass to below-knee arteries: distal vein collar or not? A prospective randomised multicentre study. *Eur J Vasc Endovasc Surg* 2010; **39**: 747–54.

207 Lindholt JS, Gottschalksen B, Johannesen N, *et al.* The Scandinavian Propaten® Trial – 1-Year Patency of PTFE Vascular Prostheses with Heparin-Bonded Luminal Surfaces Compared to Ordinary Pure PTFE Vascular Prostheses – A Randomised Clinical Controlled Multi-centre Trial. *European Journal of Vascular and Endovascular Surgery* 2011; **41**: 668–73.

© 2021 Robinson NB et al. *JAMA Network Open*.

208 Tiek J, Remy P, Sabbe T, *et al.* Laparoscopic versus Open Approach for Aortobifemoral Bypass for Severe Aorto-iliac Occlusive Disease – A Multicentre Randomised Controlled Trial. *European Journal of Vascular and Endovascular Surgery* 2012; **43**: 711–5.

209 Vriens BHR, van Det RJ, Meerwaldt R, *et al.* Superior Two-year Results of Externally Unsupported Polyester Compared to Supported Grafts in Above-knee Bypass Grafting: A Multicenter Randomised Trial. *European Journal of Vascular and Endovascular Surgery* 2013; **45**: 275–81.

210 Eker HH, Hansson BME, Buunen M, *et al.* Laparoscopic vs. open incisional hernia repair: a randomized clinical trial. *JAMA Surg* 2013; **148**: 259–63.

211 Jang J-Y, Shin YC, Han Y, *et al.* Effect of Polyglycolic Acid Mesh for Prevention of Pancreatic Fistula Following Distal Pancreatectomy: A Randomized Clinical Trial. *JAMA Surg* 2017; **152**: 150.

212 Li B, Xiang J, Zhang Y, *et al.* Comparison of Ivor-Lewis vs Sweet Esophagectomy for Esophageal Squamous Cell Carcinoma: A Randomized Clinical Trial. *JAMA Surg* 2015; **150**: 292.

213 Milone M, Fernandez LMS, Musella M, Milone F. Safety and Efficacy of Minimally Invasive Video-Assisted Ablation of Pilonidal Sinus: A Randomized Clinical Trial. *JAMA Surg* 2016; **151**: 547.

214 Håkanson BS, Lundell L, Bylund A, Thorell A. Comparison of Laparoscopic 270° Posterior Partial Fundoplication vs Total Fundoplication for the Treatment of Gastroesophageal Reflux Disease: A Randomized Clinical Trial. *JAMA Surg* 2019; **154**: 479.

215 Li Z, Shan F, Ying X, *et al.* Assessment of Laparoscopic Distal Gastrectomy After Neoadjuvant Chemotherapy for Locally Advanced Gastric Cancer: A Randomized Clinical Trial. *JAMA Surg* 2019; **154**: 1093.

216 Kohler A, Lavanchy JL, Lenoir U, Kurmann A, Candinas D, Beldi G. Effectiveness of Prophylactic Intraperitoneal Mesh Implantation for Prevention of Incisional Hernia in Patients Undergoing Open Abdominal Surgery: A Randomized Clinical Trial. *JAMA Surg* 2019; **154**: 109.

217 Guo J, Xu A, Sun X, *et al.* Combined Surgery and Extensive Intraoperative Peritoneal Lavage vs Surgery Alone for Treatment of Locally Advanced Gastric Cancer: The SEIPLUS Randomized Clinical Trial. *JAMA Surg* 2019; **154**: 610.

218 Wei S, Guo C, He J, *et al.* Effect of Vein-First vs Artery-First Surgical Technique on Circulating Tumor Cells and Survival in Patients With Non–Small Cell Lung Cancer: A Randomized Clinical Trial and Registry-Based Propensity Score Matching Analysis. *JAMA Surg* 2019; **154**: e190972.

219 Chaudhary R, Beaupré L, Johnston D. Knee Range of Motion During the First Two Years After Use of Posterior Cruciate-Stabilizing or Posterior Cruciate-Retaining Total Knee Prostheses: A Randomized Clinical Trial. *The Journal of Bone and Joint Surgery-American Volume* 2008; **90**: 2579–86.

220 Lachiewicz PF, Kelley SS, Soileau ES. Survival of Polished Compared with Precoated Roughened Cemented Femoral Components: A Prospective, Randomized Study. *The Journal of Bone and Joint Surgery-American Volume* 2008; **90**: 1457–63.

© 2021 Robinson NB et al. *JAMA Network Open*.

221 Leung F, Tu Y, Chew WY, Chow S-P. Comparison of External and Percutaneous Pin Fixation with Plate Fixation for Intra-articular Distal Radial Fractures: A Randomized Study. *The Journal of Bone and Joint Surgery-American Volume* 2008; **90**: 16–22.

222 Pagnano MW, Trousdale RT, Meneghini RM, Hanssen AD. Slower Recovery After Two-Incision Than Mini-Posterior-Incision Total Hip Arthroplasty: A Randomized Clinical Trial. *The Journal of Bone and Joint Surgery-American Volume* 2008; **90**: 1000–6.

223 Rodkey WG, DeHaven KE, Montgomery WH, *et al.* Comparison of the Collagen Meniscus Implant with Partial Meniscectomy: A Prospective Randomized Trial. *The Journal of Bone and Joint Surgery-American Volume* 2008; **90**: 1413–26.

224 Bhandari M, Guyatt G, Walter SD, *et al.* Randomized Trial of Reamed and Unreamed Intramedullary Nailing of Tibial Shaft Fractures: By the Study to Prospectively Evaluate Reamed Intramedullary Nails in Patients with Tibial Fractures (SPRINT) Investigators*. *The Journal of Bone and Joint Surgery-American Volume* 2008; **90**: 2567–78.

225 Bettinson KA, Pinder IM, Moran CG, Weir DJ, Lingard EA. All-polyethylene compared with metal-backed tibial components in total knee arthroplasty at ten years. A prospective, randomized controlled trial. *J Bone Joint Surg Am* 2009; **91**: 1587–94.

226 Dai L-Y, Jiang L-S, Jiang S-D. Posterior Short-Segment Fixation with or without Fusion for Thoracolumbar Burst Fractures: A Five to Seven-Year Prospective Randomized Study. *The Journal of Bone and Joint Surgery-American Volume* 2009; **91**: 1033–41.

227 Dalury DF, Mulliken BD, Adams MJ, Lewis C, Sauder RR, Bushey JA. Early Recovery After Total Knee Arthroplasty Performed with and without Patellar Eversion and Tibial Translation: A Prospective Randomized Study. *The Journal of Bone and Joint Surgery-American Volume* 2009; **91**: 1339–43.

228 Dunbar M, Wilson DAJ, Hennigar A, Amirault J, Gross M, Reardon G. Fixation of a Trabecular Metal Knee Arthroplasty Component: A Prospective Randomized Study. *The Journal of Bone and Joint Surgery-American Volume* 2009; **91**: 1578–86.

229 Gioe TJ, Glynn J, Sembrano J, Suthers K, Santos ER, Singh J. Mobile and Fixed-Bearing (All-Polyethylene Tibial Component) Total Knee Arthroplasty Designs: A Prospective Randomized Trial. *The Journal of Bone and Joint Surgery-American Volume* 2009; **91**: 2104–12.

230 KAT Trial Group, Johnston L, MacLennan G, McCormack K, Ramsay C, Walker A. The Knee Arthroplasty Trial (KAT) design features, baseline characteristics, and two-year functional outcomes after alternative approaches to knee replacement. *J Bone Joint Surg Am* 2009; **91**: 134–41.

231 Kim Y-H, Choi Y, Kim J-S. Range of Motion of Standard and High-Flexion Posterior Cruciate-Retaining Total Knee Prostheses*: A Prospective Randomized Study. *The Journal of Bone and Joint Surgery-American Volume* 2009; **91**: 1874–81.

232 McCalden RW, MacDonald SJ, Rorabeck CH, Bourne RB, Chess DG, Charron KD. Wear Rate of Highly Cross-Linked Polyethylene in Total Hip Arthroplasty: A Randomized Controlled Trial. *The Journal of Bone and Joint Surgery-American Volume* 2009; **91**: 773–82.

© 2021 Robinson NB et al. *JAMA Network Open*.

233 Pajala A, Kangas J, Siira P, Ohtonen P, Leppilahti J. Augmented Compared with Nonaugmented Surgical Repair of a Fresh Total Achilles Tendon Rupture: A Prospective Randomized Study. *The Journal of Bone and Joint Surgery-American Volume* 2009; **91**: 1092–100.

234 Rahme H, Mattsson P, Wikblad L, Nowak J, Larsson S. Stability of Cemented in-Line Pegged Glenoid Compared with Keeled Glenoid Components in Total Shoulder Arthroplasty: *The Journal of Bone and Joint Surgery-American Volume* 2009; **91**: 1965–72.

235 Barton TM, Gleeson R, Topliss C, Greenwood R, Harries WJ, Chesser TJ. A Comparison of the Long Gamma Nail with the Sliding Hip Screw for the Treatment of AO/OTA 31-A2 Fractures of the Proximal Part of the Femur: A Prospective Randomized Trial. *The Journal of Bone and Joint Surgery-American Volume* 2010; **92**: 792–8.

236 Bonutti PM, Zywiel MG, Ulrich SD, Stroh DA, Seyler TM, Mont MA. A Comparison of Subvastus and Midvastus Approaches in Minimally Invasive Total Knee Arthroplasty: *The Journal of Bone and Joint Surgery-American Volume* 2010; **92**: 575–82.

237 Choi WC, Lee S, Seong SC, Jung JH, Lee MC. Comparison Between Standard and High-Flexion Posterior-Stabilized Rotating-Platform Mobile-Bearing Total Knee Arthroplasties: A Randomized Controlled Study. *The Journal of Bone and Joint Surgery-American Volume* 2010; **92**: 2634–42.

238 Demey G, Servien E, Pinaroli A, Lustig S, Si Selmi TA, Neyret P. The Influence of Femoral Cementing on Perioperative Blood Loss in Total Knee Arthroplasty: A Prospective Randomized Study. *The Journal of Bone and Joint Surgery-American Volume* 2010; **92**: 536–41.

239 Kim Y-H, Choi Y, Kim J-S. Comparison of a Standard and a Gender-Specific Posterior Cruciate-Substituting High-Flexion Knee Prosthesis: A Prospective, Randomized, Short-Term Outcome Study. *The Journal of Bone and Joint Surgery-American Volume* 2010; **92**: 1911–20.

240 Wülker N, Lambermont JP, Sacchetti L, Lazaró JG, Nardi J. A Prospective Randomized Study of Minimally Invasive Total Knee Arthroplasty Compared with Conventional Surgery: *The Journal of Bone and Joint Surgery-American Volume* 2010; **92**: 1584–90.

241 Pospischill M, Kranzl A, Attwenger B, Knahr K. Minimally Invasive Compared with Traditional Transgluteal Approach for Total Hip Arthroplasty: A Comparative Gait Analysis. *The Journal of Bone & Joint Surgery* 2010; **92**: 328–37.

242 Delamarter R, Zigler JE, Balderston RA, Cammisa FP, Goldstein JA, Spivak JM. Prospective, Randomized, Multicenter Food and Drug Administration Investigational Device Exemption Study of the ProDisc-L Total Disc Replacement Compared with Circumferential Arthrodesis for the Treatment of Two-Level Lumbar Degenerative Disc Disease: Results at Twenty-four Months. *The Journal of Bone & Joint Surgery* 2011; **93**: 705–15.

243 Kim JK, Koh YD, Kook SH. Effect of Calcium Phosphate Bone Cement Augmentation on Volar Plate Fixation of Unstable Distal Radial Fractures in the Elderly: *The Journal of Bone and Joint Surgery-American Volume* 2011; **93**: 609–14.

© 2021 Robinson NB et al. *JAMA Network Open*.

244 Kim Y-H, Kim J-S, Park J-W, Joo J-H. Comparison of the Low Contact Stress and Press Fit Condylar Rotating-Platform Mobile-Bearing Prostheses in Total Knee Arthroplasty: A Prospective Randomized Study. *The Journal of Bone and Joint Surgery-American Volume* 2011; **93**: 1001–7.

245 MacDonald P, McRae S, Leiter J, Mascarenhas R, Lapner P. Arthroscopic Rotator Cuff Repair with and without Acromioplasty in the Treatment of Full-Thickness Rotator Cuff Tears: A Multicenter, Randomized Controlled Trial. *The Journal of Bone and Joint Surgery-American Volume* 2011; **93**: 1953–60.

246 Stannard JP, Bankston L, Futch LA, McGwin G, Volgas DA. Functional Outcome Following Intramedullary Nailing of the Femur: A Prospective Randomized Comparison of Piriformis Fossa and Greater Trochanteric Entry Portals. *The Journal of Bone and Joint Surgery-American Volume* 2011; **93**: 1385–91.

247 Zhang G, Chen J, Chai W, Liu M, Wang Y. Comparison Between Computer-Assisted-Navigation and Conventional Total Knee Arthroplasties in Patients Undergoing Simultaneous Bilateral Procedures: A Randomized Clinical Trial. *The Journal of Bone and Joint Surgery-American Volume* 2011; **93**: 1190–6.

248 Zhu Y, Lu Y, Shen J, Zhang J, Jiang C. Locking Intramedullary Nails and Locking Plates in the Treatment of Two-Part Proximal Humeral Surgical Neck Fractures: A Prospective Randomized Trial with a Minimum of Three Years of Follow-up. *The Journal of Bone and Joint Surgery-American Volume* 2011; **93**: 159–68.

249 Chammout GK, Mukka SS, Carlsson T, Neander GF, Helge Stark AW, Sköldenberg OG. Total Hip Replacement Versus Open Reduction and Internal Fixation of Displaced Femoral Neck Fractures: A Randomized Long-Term Follow-up Study. *The Journal of Bone & Joint Surgery* 2012; **94**: 1921–8.

250 Grewal R, Athwal GS, MacDermid JC, *et al.* Single Versus Double-Incision Technique for the Repair of Acute Distal Biceps Tendon Ruptures: A Randomized Clinical Trial. *The Journal of Bone & Joint Surgery* 2012; **94**: 1166–74.

251 Gumina S, Campagna V, Ferrazza G, *et al.* Use of Platelet-Leukocyte Membrane in Arthroscopic Repair of Large Rotator Cuff Tears: A Prospective Randomized Study. *The Journal of Bone and Joint Surgery-American Volume* 2012; **94**: 1345–52.

252 Howie DW, Holubowycz OT, Middleton R. Large Femoral Heads Decrease the Incidence of Dislocation After Total Hip Arthroplasty: A Randomized Controlled Trial. *The Journal of Bone and Joint Surgery-American Volume* 2012; **94**: 1095–102.

253 Kim Y-H, Park J-W, Kim J-S. Computer-Navigated Versus Conventional Total Knee Arthroplasty: A Prospective Randomized Trial. *The Journal of Bone and Joint Surgery-American Volume* 2012; **94**: 2017–24.

254 Lapner PL, Sabri E, Rakhra K, *et al.* A Multicenter Randomized Controlled Trial Comparing Single-Row with Double-Row Fixation in Arthroscopic Rotator Cuff Repair: *The Journal of Bone and Joint Surgery-American Volume* 2012; **94**: 1249–57.

© 2021 Robinson NB et al. *JAMA Network Open*.

255 Taylor F, Wright M, Zhu M. Hemiarthroplasty of the Hip with and without Cement: A Randomized Clinical Trial: *The Journal of Bone and Joint Surgery-American Volume* 2012; **94**: 577–83.

256 Jayasuriya RL, Buckley SC, Hamer AJ, *et al.* Effect of Sliding-Taper Compared with Composite-Beam Cemented Femoral Prosthesis Loading Regime on Proximal Femoral Bone Remodeling: A Randomized Clinical Trial. *The Journal of Bone and Joint Surgery-American Volume* 2013; **95**: 19–27.

257 Mohammadi F, Salavati M, Akhbari B, Mazaheri M, Mohsen Mir S, Etemadi Y. Comparison of Functional Outcome Measures After ACL Reconstruction in Competitive Soccer Players: A Randomized Trial. *The Journal of Bone and Joint Surgery-American Volume* 2013; **95**: 1271–7.

258 Matre K, Vinje T, Havelin LI, *et al.* TRIGEN INTERTAN Intramedullary Nail Versus Sliding Hip Screw: A Prospective, Randomized Multicenter Study on Pain, Function, and Complications in 684 Patients with an Intertrochanteric or Subtrochanteric Fracture and One Year of Follow-up. *The Journal of Bone and Joint Surgery-American Volume* 2013; **95**: 200–8.

259 Bennett DB, Hill JC, Dennison J, *et al.* Metal-Carbon Fiber Composite Femoral Stems in Hip Replacements: A Randomized Controlled Parallel-Group Study with Mean Ten-Year Follow-up. *The Journal of Bone and Joint Surgery-American Volume* 2014; **96**: 2062–9.

260 Duivenvoorden T, Brouwer RW, Baan A, *et al.* Comparison of Closing-Wedge and Opening-Wedge High Tibial Osteotomy for Medial Compartment Osteoarthritis of the Knee: A Randomized Controlled Trial with a Six-Year Follow-up. *The Journal of Bone and Joint Surgery* 2014; **96**: 1425–32.

261 Glazebrook M, Copithorne P, Boyd G, *et al.* Proximal Opening Wedge Osteotomy with Wedge-Plate Fixation Compared with Proximal Chevron Osteotomy for the Treatment of Hallux Valgus: A Prospective, Randomized Study. *The Journal of Bone and Joint Surgery* 2014; **96**: 1585–92.

262 Jenkins D, Rodriguez J, Ranawat A, *et al.* A Randomized, Controlled, Prospective Study Evaluating the Effect of Patellar Eversion on Functional Outcomes in Primary Total Knee Arthroplasty: *The Journal of Bone & Joint Surgery* 2014; **96**: 851–8.

263 Tomek IM, Kantor SR, Cori LA, *et al.* Early Patient Outcomes After Primary Total Knee Arthroplasty with Quadriceps-Sparing Subvastus and Medial Parapatellar Techniques: A Randomized, Double-Blind Clinical Trial. *The Journal of Bone and Joint Surgery-American Volume* 2014; **96**: 907–15.

264 Reid MJ, Booth G, Khan RJK, Janes G. Patellar Eversion During Total Knee Replacement: A Prospective, Randomized Trial. *The Journal of Bone & Joint Surgery* 2014; **96**: 207–13.

265 Woolson ST, Harris AH, Wagner DW, Giori NJ. Component Alignment During Total Knee Arthroplasty with Use of Standard or Custom Instrumentation: A Randomized Clinical Trial Using Computed Tomography for Postoperative Alignment Measurement. *The Journal of Bone and Joint Surgery-American Volume* 2014; **96**: 366–72.

266 Zhang T, Su Y, Chen W, Zhang Q, Wu Z, Zhang Y. Displaced Intra-Articular Calcaneal Fractures Treated in a Minimally Invasive Fashion: Longitudinal Approach Versus Sinus Tarsi Approach. *The Journal of Bone & Joint Surgery* 2014; **96**: 302–9.

© 2021 Robinson NB et al. *JAMA Network Open*.

267 Andrade-Silva FB, Kojima KE, Joeris A, Santos Silva J, Mattar R. Single, Superiorly Placed Reconstruction Plate Compared with Flexible Intramedullary Nailing for Midshaft Clavicular Fractures: A Prospective, Randomized Controlled Trial. *The Journal of Bone and Joint Surgery-American Volume* 2015; **97**: 620–6.

268 Malhotra R, Singla A, Lekha C, *et al.* A Prospective Randomized Study to Compare Systemic Emboli Using the Computer-Assisted and Conventional Techniques of Total Knee Arthroplasty: *The Journal of Bone and Joint Surgery-American Volume* 2015; **97**: 889–94.

269 van der Meijden OA, Houwert RM, Hulsmans M, *et al.* Operative Treatment of Dislocated Midshaft Clavicular Fractures: Plate or Intramedullary Nail Fixation?: A Randomized Controlled Trial. *The Journal of Bone and Joint Surgery-American Volume* 2015; **97**: 613–9.

270 Reindl R, Harvey EJ, Berry GK, Rahme E. Intramedullary Versus Extramedullary Fixation for Unstable Intertrochanteric Fractures: A Prospective Randomized Controlled Trial. *The Journal of Bone and Joint Surgery-American Volume* 2015; **97**: 1905–12.

271 Shareghi B, Johanson P-E, Kärrholm J. Femoral Head Penetration of Vitamin E-Infused Highly Cross-Linked Polyethylene Liners: A Randomized Radiostereometric Study of Seventy Hips Followed for Two Years. *The Journal of Bone and Joint Surgery-American Volume* 2015; **97**: 1366–71.

272 Bell SW, Anthony I, Jones B, MacLean A, Rowe P, Blyth M. Improved Accuracy of Component Positioning with Robotic-Assisted Unicompartmental Knee Arthroplasty: Data from a Prospective, Randomized Controlled Study. *The Journal of Bone and Joint Surgery* 2016; **98**: 627–35.

273 Heesterbeek PJC, Wymenga AB, van Hellemondt GG. No Difference in Implant Micromotion Between Hybrid Fixation and Fully Cemented Revision Total Knee Arthroplasty: A Randomized Controlled Trial with Radiostereometric Analysis of Patients with Mild-to-Moderate Bone Loss. *The Journal of Bone and Joint Surgery* 2016; **98**: 1359–69.

274 Howie DW, Holubowycz OT, Callary SA. The Wear Rate of Highly Cross-Linked Polyethylene in Total Hip Replacement Is Not Increased by Large Articulations: A Randomized Controlled Trial. *The Journal of Bone and Joint Surgery* 2016; **98**: 1786–93.

275 Verburg H, Mathijssen NMC, Niesten D-D, Verhaar JAN, Pilot P. Comparison of Mini-Midvastus and Conventional Total Knee Arthroplasty with Clinical and Radiographic Evaluation: A Prospective Randomized Clinical Trial with 5-Year Follow-up. *The Journal of Bone and Joint Surgery* 2016; **98**: 1014–22.

276 Duckworth AD, Clement ND, White TO, Court-Brown CM, McQueen MM. Plate Versus Tension-Band Wire Fixation for Olecranon Fractures: A Prospective Randomized Trial. *The Journal of Bone and Joint Surgery* 2017; **99**: 1261–73.

277 Andersen MR, Frihagen F, Hellund JC, Madsen JE, Figved W. Randomized Trial Comparing Suture Button with Single Syndesmotic Screw for Syndesmosis Injury: *The Journal of Bone and Joint Surgery* 2018; **100**: 2–12.

© 2021 Robinson NB et al. *JAMA Network Open*.

278 Strickland CD, Kraeutler MJ, Brick MJ, *et al.* MRI Evaluation of Repaired Versus Unrepaired Interportal Capsulotomy in Simultaneous Bilateral Hip Arthroscopy: A Double-Blind, Randomized Controlled Trial. *The Journal of Bone and Joint Surgery* 2018; **100**: 91–8.

279 Dolatowski FC, Frihagen F, Bartels S, *et al.* Screw Fixation Versus Hemiarthroplasty for Nondisplaced Femoral Neck Fractures in Elderly Patients: A Multicenter Randomized Controlled Trial. *The Journal of Bone and Joint Surgery* 2019; **101**: 136–44.

280 King PR, Ikram A, Eken MM, Lamberts RP. The Effectiveness of a Flexible Locked Intramedullary Nail and an Anatomically Contoured Locked Plate to Treat Clavicular Shaft Fractures: A 1-Year Randomized Control Trial. *The Journal of Bone and Joint Surgery* 2019; **101**: 628–34.

281 Nam D, Lawrie CM, Salih R, Nahhas CR, Barrack RL, Nunley RM. Cemented Versus Cementless Total Knee Arthroplasty of the Same Modern Design: A Prospective, Randomized Trial. *The Journal of Bone and Joint Surgery* 2019; **101**: 1185–92.

282 Langford P, Wolfe R, Danks RA. Wound healing after craniotomy: a randomized trial comparing scalp clips to artery forceps for scalp hemostasis: Clinical article. *JNS* 2009; **111**: 1175–8.

283 Xiao B, Wu F-F, Zhang H, Ma Y-B. A randomized study of urgent computed tomography–based hematoma puncture and aspiration in the emergency department and subsequent evacuation using craniectomy versus craniectomy only: Clinical article. *JNS* 2012; **117**: 566–73.

284 Hutter G, von Felten S, Sailer MH, Schulz M, Mariani L. Risk factors for postoperative CSF leakage after elective craniotomy and the efficacy of fleece-bound tissue sealing against dural suturing alone: a randomized controlled trial: Clinical article. *JNS* 2014; **121**: 735–44.

285 Schucht P, Banz V, Trochsler M, *et al.* Laparoscopically assisted ventriculoperitoneal shunt placement: a prospective randomized controlled trial. *JNS* 2015; **122**: 1058–67.

286 Welling LC, Figueiredo EG, Wen HT, *et al.* Prospective randomized study comparing clinical, functional, and aesthetic results of minipterional and classic pterional craniotomies. *JNS* 2015; **122**: 1012–9.

287 Fonoff ET, Azevedo A, Angelos JS dos, *et al.* Simultaneous bilateral stereotactic procedure for deep brain stimulation implants: a significant step for reducing operation time. *JNS* 2016; **125**: 85–9.

288 Chugh AJ, Pace JR, Singer J, *et al.* Use of a surgical rehearsal platform and improvement in aneurysm clipping measures: results of a prospective, randomized trial. *JNS* 2017; **126**: 838–44.

289 Honeybul S, Morrison DA, Ho KM, Lind CRP, Geelhoed E. A randomized controlled trial comparing autologous cranioplasty with custom-made titanium cranioplasty. *JNS* 2017; **126**: 81–90.

290 Lindner D, Schlothofer-Schumann K, Kern B-C, Marx O, Müns A, Meixensberger J. Cranioplasty using custom-made hydroxyapatite versus titanium: a randomized clinical trial. *JNS* 2017; **126**: 175–83.

291 Vieira E, Guimarães TC, Faquini IV, *et al.* Randomized controlled study comparing 2 surgical techniques for decompressive craniectomy: with watertight duraplasty and without watertight duraplasty. *Journal of Neurosurgery* 2018; **129**: 1017–23.

© 2021 Robinson NB et al. *JAMA Network Open*.

292 Heyligers JMM, Lisman T, Verhagen HJM, Weeterings C, de Groot PG, Moll FL. A heparin-bonded vascular graft generates no systemic effect on markers of hemostasis activation or detectable heparin-induced thrombocytopenia–associated antibodies in humans. *Journal of Vascular Surgery* 2008; **47**: 324–9.

293 Keuter XHA, De Smet AAEA, Kessels AGH, van der Sande FM, Welten RJThJ, Tordoir JHM. A randomized multicenter study of the outcome of brachial-basilic arteriovenous fistula and prosthetic brachial-antecubital forearm loop as vascular access for hemodialysis. *Journal of Vascular Surgery* 2008; **47**: 395–401.

294 AbuRahma AF, Stone PA, Hass SM, *et al.* Prospective randomized trial of routine versus selective shunting in carotid endarterectomy based on stump pressure. *Journal of Vascular Surgery* 2010; **51**: 1133–8.

295 Kennealey PT, Elias N, Hertl M, *et al.* A prospective, randomized comparison of bovine carotid artery and expanded polytetrafluoroethylene for permanent hemodialysis vascular access. *Journal of Vascular Surgery* 2011; **53**: 1640–8.

296 Morosetti M, Cipriani S, Dominijanni S, Pisani G, Frattarelli D, Bruno F. Basilic vein transposition versus biosynthetic prosthesis as vascular access for hemodialysis. *Journal of Vascular Surgery* 2011; **54**: 1713–9.

297 Stollwerck PL, Kozlowski B, Sandmann W, Grabitz K, Pfeiffer T. Long-term dilatation of polyester and expanded polytetrafluoroethylene tube grafts after open repair of infrarenal abdominal aortic aneurysms. *Journal of Vascular Surgery* 2011; **53**: 1506–13.

298 Aitken E, Jeans E, Aitken M, Kingsmore D. A randomized controlled trial of interrupted versus continuous suturing techniques for radiocephalic fistulas. *Journal of Vascular Surgery* 2015; **62**: 1575–82.

299 Lumsden AB, Morrissey NJ, Staffa R, *et al.* Randomized controlled trial comparing the safety and efficacy between the FUSION BIOLINE heparin-coated vascular graft and the standard expanded polytetrafluoroethylene graft for femoropopliteal bypass. *Journal of Vascular Surgery* 2015; **61**: 703-712.e1.

300 Shemesh D, Goldin I, Hijazi J, *et al.* A prospective randomized study of heparin-bonded graft (Propaten) versus standard graft in prosthetic arteriovenous access. *J Vasc Surg* 2015; **62**: 115–22.

301 Damgaard S, Lund JT, Lilleør NB, *et al.* Comparable three months' outcome of total arterial revascularization versus conventional coronary surgery: Copenhagen Arterial Revascularization Randomized Patency and Outcome trial. *The Journal of Thoracic and Cardiovascular Surgery* 2008; **135**: 1069–75.

302 Droghetti A, Schiavini A, Muriana P, *et al.* A prospective randomized trial comparing completion technique of fissures for lobectomy: Stapler versus precision dissection and sealant. *The Journal of Thoracic and Cardiovascular Surgery* 2008; **136**: 383–91.

© 2021 Robinson NB et al. *JAMA Network Open*.

303 Falk V, Seeburger J, Czesla M, *et al.* How does the use of polytetrafluoroethylene neochordae for posterior mitral valve prolapse (loop technique) compare with leaflet resection? A prospective randomized trial. *The Journal of Thoracic and Cardiovascular Surgery* 2008; **136**: 1200–6.

304 Glineur D, Hanet C, Poncelet A, *et al.* Comparison of saphenous vein graft versus right gastroepiploic artery to revascularize the right coronary artery: A prospective randomized clinical, functional, and angiographic midterm evaluation. *The Journal of Thoracic and Cardiovascular Surgery* 2008; **136**: 482–8.

305 Guenzinger R, Eichinger WB, Hettich I, *et al.* A prospective randomized comparison of the Medtronic Advantage Supra and St Jude Medical Regent mechanical heart valves in the aortic position: Is there an additional benefit of supra-annular valve positioning? *The Journal of Thoracic and Cardiovascular Surgery* 2008; **136**: 462–71.

306 Kamiya H, Akhyari P, Martens A, Karck M, Haverich A, Lichtenberg A. Sternal microcirculation after skeletonized versus pedicled harvesting of the internal thoracic artery: A randomized study. *The Journal of Thoracic and Cardiovascular Surgery* 2008; **135**: 32–7.

307 Narang S, Satsangi DK, Banerjee A, Geelani MA. Stentless valves versus stented bioprostheses at the aortic position: Midterm results. *The Journal of Thoracic and Cardiovascular Surgery* 2008; **136**: 943–7.

308 Puskas JD, Halkos ME, Balkhy H, *et al.* Evaluation of the PAS-Port Proximal Anastomosis System in coronary artery bypass surgery (the EPIC trial). *The Journal of Thoracic and Cardiovascular Surgery* 2009; **138**: 125–32.

309 Fattouch K, Guccione F, Dioguardi P, *et al.* Off-pump versus on-pump myocardial revascularization in patients with ST-segment elevation myocardial infarction: A randomized trial. *The Journal of Thoracic and Cardiovascular Surgery* 2009; **137**: 650–7.

310 Formica F, Broccolo F, Martino A, *et al.* Myocardial revascularization with miniaturized extracorporeal circulation versus off pump: Evaluation of systemic and myocardial inflammatory response in a prospective randomized study. *The Journal of Thoracic and Cardiovascular Surgery* 2009; **137**: 1206–12.

311 Fattouch K, Guccione F, Sampognaro R, *et al.* POINT: Efficacy of adding mitral valve restrictive annuloplasty to coronary artery bypass grafting in patients with moderate ischemic mitral valve regurgitation: A randomized trial. *The Journal of Thoracic and Cardiovascular Surgery* 2009; **138**: 278–85.

312 Sakwa MP, Emery RW, Shannon FL, *et al.* Coronary artery bypass grafting with a minimized cardiopulmonary bypass circuit: A prospective, randomized trial. *The Journal of Thoracic and Cardiovascular Surgery* 2009; **137**: 481–5.

313 Nasso G, Coppola R, Bonifazi R, Piancone F, Bozzetti G, Speziale G. Arterial revascularization in primary coronary artery bypass grafting: Direct comparison of 4 strategies--results of the Stand-in-Y Mammary Study. *J Thorac Cardiovasc Surg* 2009; **137**: 1093–100.

© 2021 Robinson NB et al. *JAMA Network Open*.

314 Belcher E, Dusmet M, Jordan S, Ladas G, Lim E, Goldstraw P. A prospective, randomized trial comparing BioGlue and Vivostat for the control of alveolar air leak. *The Journal of Thoracic and Cardiovascular Surgery* 2010; **140**: 32–8.

315 Markman PL, Rowland MA, Leong J-Y, *et al.* Skeletonized internal thoracic artery harvesting reduces chest wall dysesthesia after coronary bypass surgery. *The Journal of Thoracic and Cardiovascular Surgery* 2010; **139**: 674–9.

316 Ponce González MA, Serdá GJ, Suarez PR, Perez-Peñate G, Gilart JF, Navarro PC. Long-term cardiopulmonary function after thoracic sympathectomy: Comparison between the conventional and simplified techniques. *The Journal of Thoracic and Cardiovascular Surgery* 2010; **139**: 405–10.

317 Roshanali F, Saidi B, Mandegar MH, Yousefnia MA, Alaeddini F. Echocardiographic approach to the decision-making process for tricuspid valve repair. *The Journal of Thoracic and Cardiovascular Surgery* 2010; **139**: 1483–7.

318 Salehi Omran A, Karimi A, Ahmadi H, Yazdanifard P, Sheikh Fahtollahi M, Tazik M. Prophylactic ventral cardiac denervation: Does it reduce incidence of atrial fibrillation after coronary artery bypass grafting? *The Journal of Thoracic and Cardiovascular Surgery* 2010; **140**: 1036–9.

319 Wiklund L, Setina M, Tsang K, Cusimano R, Yau T. A multicenter prospective randomized trial of a second-generation anastomotic device in coronary artery bypass surgery. *The Journal of Thoracic and Cardiovascular Surgery* 2010; **139**: 741–7.

320 Darling GE, Allen MS, Decker PA, *et al.* Randomized trial of mediastinal lymph node sampling versus complete lymphadenectomy during pulmonary resection in the patient with N0 or N1 (less than hilar) non–small cell carcinoma: Results of the American College of Surgery Oncology Group Z0030 Trial. *The Journal of Thoracic and Cardiovascular Surgery* 2011; **141**: 662–70.

321 Al-Rashidi F, Landenhed M, Blomquist S, *et al.* Comparison of the effectiveness and safety of a new de-airing technique with a standardized carbon dioxide insufflation technique in open left heart surgery: A randomized clinical trial. *The Journal of Thoracic and Cardiovascular Surgery* 2011; **141**: 1128–33.

322 Bonacchi M, Harmelin G, Peris A, Sani G. A novel strategy to improve systemic oxygenation in venovenous extracorporeal membrane oxygenation: The "χ-configuration". *The Journal of Thoracic and Cardiovascular Surgery* 2011; **142**: 1197–204.

323 De Leyn P, Muller M-R, Oosterhuis JWA, *et al.* Prospective European multicenter randomized trial of PleuraSeal for control of air leaks after elective pulmonary resection. *The Journal of Thoracic and Cardiovascular Surgery* 2011; **141**: 881–7.

324 Michaux I, Filipovic M, Skarvan K, *et al.* A randomized comparison of right ventricular function after on-pump versus off-pump coronary artery bypass graft surgery. *The Journal of Thoracic and Cardiovascular Surgery* 2011; **141**: 361–7.

325 Speziale G, Nasso G, Esposito G, *et al.* Results of mitral valve repair for Barlow disease (bileaflet prolapse) via right minithoracotomy versus conventional median sternotomy: A randomized trial. *The Journal of Thoracic and Cardiovascular Surgery* 2011; **142**: 77–83.

© 2021 Robinson NB et al. *JAMA Network Open*.

326 Benedetto U, Melina G, Angeloni E, *et al.* Prophylactic tricuspid annuloplasty in patients with dilated tricuspid annulus undergoing mitral valve surgery. *The Journal of Thoracic and Cardiovascular Surgery* 2012; **143**: 632–8.

327 Chaudhuri K, Storey E, Lee GA, *et al.* Carbon dioxide insufflation in open-chamber cardiac surgery: A double-blind, randomized clinical trial of neurocognitive effects. *The Journal of Thoracic and Cardiovascular Surgery* 2012; **144**: 646-653.e1.

328 Lemma MG, Coscioni E, Tritto FP, *et al.* On-pump versus off-pump coronary artery bypass surgery in high-risk patients: Operative results of a prospective randomized trial (on-off study). *The Journal of Thoracic and Cardiovascular Surgery* 2012; **143**: 625–31.

329 Hwang HY, Kim JS, Oh SJ, Kim K-B. A randomized comparison of the Saphenous Vein Versus Right Internal Thoracic Artery as a Y-Composite Graft (SAVE RITA) trial: Early results. *The Journal of Thoracic and Cardiovascular Surgery* 2012; **144**: 1027–35.

330 Suri RM, Michelena HI, Burkhart HM, *et al.* A prospective, randomized comparison of 3 contemporary bioprosthetic aortic valves: Should hemodynamic performance influence device selection? *The Journal of Thoracic and Cardiovascular Surgery* 2012; **144**: 1387–98.

331 Rogers CA, Pike K, Angelini GD, *et al.* An open randomized controlled trial of median sternotomy versus anterolateral left thoracotomy on morbidity and health care resource use in patients having off-pump coronary artery bypass surgery: The Sternotomy Versus Thoracotomy (STET) trial. *The Journal of Thoracic and Cardiovascular Surgery* 2013; **146**: 306-316.e9.

332 Bouchard D, Jensen H, Carrier M, *et al.* Effect of systematic downsizing rigid ring annuloplasty in patients with moderate ischemic mitral regurgitation. *The Journal of Thoracic and Cardiovascular Surgery* 2014; **147**: 1471–7.

333 Svensson LG, Blackstone EH, Apperson-Hansen C, *et al.* Implications from neurologic assessment of brain protection for total arch replacement from a randomized trial. *J Thorac Cardiovasc Surg* 2015; **150**: 1140-1147.e11.

334 Lee R, Vassallo P, Kruse J, *et al.* A randomized, prospective pilot comparison of 3 atrial appendage elimination techniques: Internal ligation, stapled excision, and surgical excision. *The Journal of Thoracic and Cardiovascular Surgery* 2016; **152**: 1075–80.

335 Allen KB, Thourani VH, Naka Y, *et al.* Randomized, multicenter trial comparing sternotomy closure with rigid plate fixation to wire cerclage. *The Journal of Thoracic and Cardiovascular Surgery* 2017; **153**: 888-896.e1.

336 Halkos ME, Anderson A, Binongo JNG, *et al.* Operative strategies to reduce cerebral embolic events during on- and off-pump coronary artery bypass surgery: A stratified, prospective randomized trial. *The Journal of Thoracic and Cardiovascular Surgery* 2017; **154**: 1278-1285.e1.

337 Ad N, Holmes SD, Massimiano PS, Rongione AJ, Fornaresio LM, Fitzgerald D. The use of del Nido cardioplegia in adult cardiac surgery: A prospective randomized trial. *The Journal of Thoracic and Cardiovascular Surgery* 2018; **155**: 1011–8.

© 2021 Robinson NB et al. *JAMA Network Open*.

338 Marasco SF, Fuller L, Zimmet A, *et al.* Prospective, randomized, controlled trial of polymer cable ties versus standard wire closure of midline sternotomy. *The Journal of Thoracic and Cardiovascular Surgery* 2018; **156**: 1589-1595.e1.

339 Nair SK, Sudarshan CD, Thorpe BS, *et al.* Mini-Stern Trial: A randomized trial comparing mini-sternotomy to full median sternotomy for aortic valve replacement. *The Journal of Thoracic and Cardiovascular Surgery* 2018; **156**: 2124-2132.e31.

340 Wang H, Han J, Wang Z, *et al.* A prospective randomized trial of the cut-and-sew Maze procedure in patients undergoing surgery for rheumatic mitral valve disease. *The Journal of Thoracic and Cardiovascular Surgery* 2018; **155**: 608–17.

341 Braathen B, Husebye T, Lunde IG, Tønnessen T. Trifecta has lower gradient and less prosthesis–patient mismatch than Mosaic Ultra in the aortic position: A prospective randomized study. *The Journal of Thoracic and Cardiovascular Surgery* 2019; **158**: 1032–9.

342 Szwed K, Pawliszak W, Szwed M, Tomaszewska M, Anisimowicz L, Borkowska A. Reducing delirium and cognitive dysfunction after off-pump coronary bypass: A randomized trial. *The Journal of Thoracic and Cardiovascular Surgery* 2019; : S0022522319320914.

343 Jiwnani S, Ranganathan P, Patil V, Agarwal V, Karimundackal G, Pramesh CS. Pain after posterolateral versus nerve-sparing thoracotomy: A randomized trial. *The Journal of Thoracic and Cardiovascular Surgery* 2019; **157**: 380–6.

344 Suzuki K, Saji H, Aokage K, *et al.* Comparison of pulmonary segmentectomy and lobectomy: Safety results of a randomized trial. *The Journal of Thoracic and Cardiovascular Surgery* 2019; **158**: 895–907.

345 Sasako M, Sano T, Yamamoto S, *et al.* D2 lymphadenectomy alone or with para-aortic nodal dissection for gastric cancer. *N Engl J Med* 2008; **359**: 453–62.

346 Slaughter MS, Rogers JG, Milano CA, *et al.* Advanced Heart Failure Treated with Continuous-Flow Left Ventricular Assist Device. *N Engl J Med* 2009; **361**: 2241–51.

347 Shroyer AL, Grover FL, Hattler B, *et al.* On-Pump versus Off-Pump Coronary-Artery Bypass Surgery. *N Engl J Med* 2009; **361**: 1827–37.

348 Jones RH, Velazquez EJ, Michler RE, *et al.* Coronary Bypass Surgery with or without Surgical Ventricular Reconstruction. *N Engl J Med* 2009; **360**: 1705–17.

349 Altman D, Väyrynen T, Engh ME, Axelsen S, Falconer C, Nordic Transvaginal Mesh Group. Anterior colporrhaphy versus transvaginal mesh for pelvic-organ prolapse. *N Engl J Med* 2011; **364**: 1826–36.

350 Lamy A, Devereaux PJ, Prabhakaran D, *et al.* Off-Pump or On-Pump Coronary-Artery Bypass Grafting at 30 Days. *N Engl J Med* 2012; **366**: 1489–97.

351 Sihvonen R, Paavola M, Malmivaara A, *et al.* Arthroscopic Partial Meniscectomy versus Sham Surgery for a Degenerative Meniscal Tear. *N Engl J Med* 2013; **369**: 2515–24.

© 2021 Robinson NB et al. *JAMA Network Open*.

352 Diegeler A, Börgermann J, Kappert U, *et al.* Off-pump versus on-pump coronary-artery bypass grafting in elderly patients. *N Engl J Med* 2013; **368**: 1189–98.

353 Acker MA, Parides MK, Perrault LP, *et al.* Mitral-Valve Repair versus Replacement for Severe Ischemic Mitral Regurgitation. *N Engl J Med* 2014; **370**: 23–32.

354 Smith PK, Puskas JD, Ascheim DD, *et al.* Surgical Treatment of Moderate Ischemic Mitral Regurgitation. *N Engl J Med* 2014; **371**: 2178–88.

355 Chagpar AB, Killelea BK, Tsangaris TN, *et al.* A Randomized, Controlled Trial of Cavity Shave Margins in Breast Cancer. *N Engl J Med* 2015; **373**: 503–10.

356 Bonjer HJ, Deijen CL, Abis GA, *et al.* A Randomized Trial of Laparoscopic versus Open Surgery for Rectal Cancer. *N Engl J Med* 2015; **372**: 1324–32.

357 D'Cruz AK, Vaish R, Kapre N, *et al.* Elective versus Therapeutic Neck Dissection in Node-Negative Oral Cancer. *N Engl J Med* 2015; **373**: 521–9.

358 Gillinov AM, Gelijns AC, Parides MK, *et al.* Surgical Ablation of Atrial Fibrillation during Mitral-Valve Surgery. *N Engl J Med* 2015; **372**: 1399–409.

359 Försth P, Ólafsson G, Carlsson T, *et al.* A Randomized, Controlled Trial of Fusion Surgery for Lumbar Spinal Stenosis. *N Engl J Med* 2016; **374**: 1413–23.

360 Rogers JG, Pagani FD, Tatooles AJ, *et al.* Intrapericardial Left Ventricular Assist Device for Advanced Heart Failure. *N Engl J Med* 2017; **376**: 451–60.

361 Mehra MR, Naka Y, Uriel N, *et al.* A Fully Magnetically Levitated Circulatory Pump for Advanced Heart Failure. *N Engl J Med* 2017; **376**: 440–50.

362 Ramirez PT, Frumovitz M, Pareja R, *et al.* Minimally Invasive versus Abdominal Radical Hysterectomy for Cervical Cancer. *N Engl J Med* 2018; **379**: 1895–904.

363 Mariette C, Markar SR, Dabakuyo-Yonli TS, *et al.* Hybrid Minimally Invasive Esophagectomy for Esophageal Cancer. *N Engl J Med* 2019; **380**: 152–62.

364 The HEALTH Investigators. Total Hip Arthroplasty or Hemiarthroplasty for Hip Fracture. *N Engl J Med* 2019; **381**: 2199–208.

365 Harter P, Sehouli J, Lorusso D, *et al.* A Randomized Trial of Lymphadenectomy in Patients with Advanced Ovarian Neoplasms. *N Engl J Med* 2019; **380**: 822–32.

366 Ryang Y-M, Oertel MF, Mayfrank L, Gilsbach JM, Rohde V. Standard open microdiscectomy versus minimal access trocar microdiscectomy: results of a prospective randomized study. *Neurosurgery* 2008; **62**: 174–81; discussion 181-182.

367 Rosen CL, Steinberg GK, DeMonte F, *et al.* Results of the Prospective, Randomized, Multicenter Clinical Trial Evaluating a Biosynthesized Cellulose Graft for Repair of Dural Defects. *Neurosurgery* 2011; **69**: 1093–104.

© 2021 Robinson NB et al. *JAMA Network Open*.

368 van den Akker ME, Arts MP, van den Hout WB, Brand R, Koes BW, Peul WC. Tubular Diskectomy vs Conventional Microdiskectomy for the Treatment of Lumbar Disk-Related Sciatica: Cost Utility Analysis Alongside a Double-Blind Randomized Controlled Trial. *Neurosurgery* 2011; **69**: 829–36.

369 Villavicencio AT, Babuska JM, Ashton A, *et al.* Prospective, Randomized, Double-Blind Clinical Study Evaluating the Correlation of Clinical Outcomes and Cervical Sagittal Alignment. *Neurosurgery* 2011; **68**: 1309–16.

370 Manzano GR, Casella G, Wang MY, Vanni S, Levi AD. A Prospective, Randomized Trial Comparing Expansile Cervical Laminoplasty and Cervical Laminectomy and Fusion for Multilevel Cervical Myelopathy. *Neurosurgery* 2012; **70**: 264–77.

371 Schmidt S, Kleist Welch-Guerra W, Matthes M, Baldauf J, Schminke U, Schroeder HWS. Endoscopic vs Open Decompression of the Ulnar Nerve in Cubital Tunnel Syndrome. *Neurosurgery* 2015; **77**: 960–71.

372 George B, Matula C, Kihlström L, Ferrer E, Tetens V. Safety and Efficacy of TachoSil (Absorbable Fibrin Sealant Patch) Compared With Current Practice for the Prevention of Cerebrospinal Fluid Leaks in Patients Undergoing Skull Base Surgery: A Randomized Controlled Trial. *Neurosurgery* 2017; **80**: 847–53.

373 Choi W-S, Kim J-S, Hur J-W, Seong J-H. Minimally Invasive Transforaminal Lumbar Interbody Fusion Using Banana-Shaped and Straight Cages: Radiological and Clinical Results from a Prospective Randomized Clinical Trial. *Neurosurgery* 2018; **82**: 289–98.

374 Meyer B, Baranto A, Schils F, *et al.* Percutaneous Interspinous Spacer vs Decompression in Patients with Neurogenic Claudication: An Alternative in Selected Patients? *Neurosurgery* 2018; **82**: 621–9.

375 Soleman J, Lutz K, Schaedelin S, *et al.* Subperiosteal vs Subdural Drain After Burr-Hole Drainage of Chronic Subdural Hematoma: A Randomized Clinical Trial (cSDH-Drain-Trial). *Neurosurgery* 2019; **85**: E825–34.

376 ASTEC study group, Kitchener H, Swart AMC, Qian Q, Amos C, Parmar MKB. Efficacy of systematic pelvic lymphadenectomy in endometrial cancer (MRC ASTEC trial): a randomised study. *Lancet* 2009; **373**: 125–36.

377 Santarius T, Kirkpatrick PJ, Ganesan D, *et al.* Use of drains versus no drains after burr-hole evacuation of chronic subdural haematoma: a randomised controlled trial. *Lancet* 2009; **374**: 1067–73.

378 El-Hamamsy I, Eryigit Z, Stevens L-M, *et al.* Long-term outcomes after autograft versus homograft aortic root replacement in adults with aortic valve disease: a randomised controlled trial. *The Lancet* 2010; **376**: 524–31.

379 Diener MK, Seiler CM, Rossion I, *et al.* Efficacy of stapler versus hand-sewn closure after distal pancreatectomy (DISPACT): a randomised, controlled multicentre trial. *The Lancet* 2011; **377**: 1514–22.

© 2021 Robinson NB et al. *JAMA Network Open*.

380 Gillgren P, Drzewiecki KT, Niin M, *et al.* 2-cm versus 4-cm surgical excision margins for primary cutaneous melanoma thicker than 2 mm: a randomised, multicentre trial. *Lancet* 2011; **378**: 1635–42.

381 Biere SS, van Berge Henegouwen MI, Maas KW, *et al.* Minimally invasive versus open oesophagectomy for patients with oesophageal cancer: a multicentre, open-label, randomised controlled trial. *The Lancet* 2012; **379**: 1887–92.

382 CORONIS Collaborative Group, Abalos E, Addo V, *et al.* Caesarean section surgical techniques (CORONIS): a fractional, factorial, unmasked, randomised controlled trial. *Lancet* 2013; **382**: 234–48.

383 Kølle S-FT, Fischer-Nielsen A, Mathiasen AB, *et al.* Enrichment of autologous fat grafts with ex-vivo expanded adipose tissue-derived stem cells for graft survival: a randomised placebo-controlled trial. *Lancet* 2013; **382**: 1113–20.

384 Tsujinaka T, Yamamoto K, Fujita J, *et al.* Subcuticular sutures versus staples for skin closure after open gastrointestinal surgery: a phase 3, multicentre, open-label, randomised controlled trial. *Lancet* 2013; **382**: 1105–12.

385 Mol F, van Mello NM, Strandell A, *et al.* Salpingotomy versus salpingectomy in women with tubal pregnancy (ESEP study): an open-label, multicentre, randomised controlled trial. *The Lancet* 2014; **383**: 1483–9.

386 Rintoul RC, Ritchie AJ, Edwards JG, *et al.* Efficacy and cost of video-assisted thoracoscopic partial pleurectomy versus talc pleurodesis in patients with malignant pleural mesothelioma (MesoVATS): an open-label, randomised, controlled trial. *The Lancet* 2014; **384**: 1118–27.

387 Slaghekke F, Lopriore E, Lewi L, *et al.* Fetoscopic laser coagulation of the vascular equator versus selective coagulation for twin-to-twin transfusion syndrome: an open-label randomised controlled trial. *The Lancet* 2014; **383**: 2144–51.

388 Vennix S, Musters GD, Mulder IM, *et al.* Laparoscopic peritoneal lavage or sigmoidectomy for perforated diverticulitis with purulent peritonitis: a multicentre, parallel-group, randomised, open-label trial. *The Lancet* 2015; **386**: 1269–77.

389 da Costa DW, Bouwense SA, Schepers NJ, *et al.* Same-admission versus interval cholecystectomy for mild gallstone pancreatitis (PONCHO): a multicentre randomised controlled trial. *The Lancet* 2015; **386**: 1261–8.

390 Deerenberg EB, Harlaar JJ, Steyerberg EW, *et al.* Small bites versus large bites for closure of abdominal midline incisions (STITCH): a double-blind, multicentre, randomised controlled trial. *The Lancet* 2015; **386**: 1254–60.

391 Watson AJM, Hudson J, Wood J, *et al.* Comparison of stapled haemorrhoidopexy with traditional excisional surgery for haemorrhoidal disease (eTHoS): a pragmatic, multicentre, randomised controlled trial. *The Lancet* 2016; **388**: 2375–85.

© 2021 Robinson NB et al. *JAMA Network Open*.

392 Brown SR, Tiernan JP, Watson AJM, *et al.* Haemorrhoidal artery ligation versus rubber band ligation for the management of symptomatic second-degree and third-degree haemorrhoids (HubBLe): a multicentre, open-label, randomised controlled trial. *The Lancet* 2016; **388**: 356–64.

393 Stenberg E, Szabo E, Ågren G, *et al.* Closure of mesenteric defects in laparoscopic gastric bypass: a multicentre, randomised, parallel, open-label trial. *The Lancet* 2016; **387**: 1397–404.

394 Yaxley JW, Coughlin GD, Chambers SK, *et al.* Robot-assisted laparoscopic prostatectomy versus open radical retropubic prostatectomy: early outcomes from a randomised controlled phase 3 study. *The Lancet* 2016; **388**: 1057–66.

395 Diener MK, Hüttner FJ, Kieser M, *et al.* Partial pancreatoduodenectomy versus duodenum-preserving pancreatic head resection in chronic pancreatitis: the multicentre, randomised, controlled, double-blind ChroPac trial. *Lancet* 2017; **390**: 1027–37.

396 Nauth A, Creek AT, Zellar A, *et al.* Fracture fixation in the operative management of hip fractures (FAITH): an international, multicentre, randomised controlled trial. *The Lancet* 2017; **389**: 1519–27.

397 Rullier E, Rouanet P, Tuech J-J, *et al.* Organ preservation for rectal cancer (GRECCAR 2): a prospective, randomised, open-label, multicentre, phase 3 trial. *The Lancet* 2017; **390**: 469–79.

398 Glazener CM, Breeman S, Elders A, *et al.* Mesh, graft, or standard repair for women having primary transvaginal anterior or posterior compartment prolapse surgery: two parallel-group, multicentre, randomised, controlled trials (PROSPECT). *The Lancet* 2017; **389**: 381–92.

399 Kaufmann R, Halm JA, Eker HH, *et al.* Mesh versus suture repair of umbilical hernia in adults: a randomised, double-blind, controlled, multicentre trial. *The Lancet* 2018; **391**: 860–9.

400 Parekh DJ, Reis IM, Castle EP, *et al.* Robot-assisted radical cystectomy versus open radical cystectomy in patients with bladder cancer (RAZOR): an open-label, randomised, phase 3, non-inferiority trial. *Lancet* 2018; **391**: 2525–36.

401 Cooper K, Breeman S, Scott NW, *et al.* Laparoscopic supracervical hysterectomy versus endometrial ablation for women with heavy menstrual bleeding (HEALTH): a parallel-group, open-label, randomised controlled trial. *The Lancet* 2019; **394**: 1425–36.

402 Beard DJ, Davies LJ, Cook JA, *et al.* The clinical and cost-effectiveness of total versus partial knee replacement in patients with medial compartment osteoarthritis (TOPKAT): 5-year outcomes of a randomised controlled trial. *The Lancet* 2019; **394**: 746–56.

403 Robert M, Espalieu P, Pelascini E, *et al.* Efficacy and safety of one anastomosis gastric bypass versus Roux-en-Y gastric bypass for obesity (YOMEGA): a multicentre, randomised, open-label, non-inferiority trial. *The Lancet* 2019; **393**: 1299–309.

© 2021 Robinson NB et al. *JAMA Network Open*.
